# Unprecedented Combination of Polyketide Natural Product Fragments Identifies the New Hedgehog Signaling Pathway Inhibitor Grismonone

**DOI:** 10.1002/chem.202202164

**Published:** 2022-10-13

**Authors:** Michael Grigalunas, Sohan Patil, Adrian Krzyzanowski, Axel Pahl, Jana Flegel, Beate Schölermann, Jianing Xie, Sonja Sievers, Slava Ziegler, Herbert Waldmann

**Affiliations:** ^1^ Max Planck Institute of Molecular Physiology Department of Chemical Biology Dortmund 44227 Germany; ^2^ Technical University Dortmund Faculty of Chemistry Chemical Biology Dortmund 44227 Germany; ^3^ Compound Management and Screening Center Dortmund 44227 Germany

**Keywords:** chemical evolution, griseofulvin, hedgehog signaling inhibitors, polyketide natural product fragments, pseudo-natural products

## Abstract

Pseudo‐natural products (pseudo‐NPs) are de novo combinations of natural product (NP) fragments that define novel bioactive chemotypes. For their discovery, new design principles are being sought. Previously, pseudo‐NPs were synthesized by the combination of fragments originating from biosynthetically unrelated NPs to guarantee structural novelty and novel bioactivity. We report the combination of fragments from biosynthetically related NPs in novel arrangements to yield a novel chemotype with activity not shared by the guiding fragments. We describe the synthesis of the polyketide pseudo‐NP grismonone and identify it as a structurally novel and potent inhibitor of Hedgehog signaling. The insight that the de novo combination of fragments derived from biosynthetically related NPs may also yield new biologically relevant compound classes with unexpected bioactivity may be considered a chemical extension or diversion of existing biosynthetic pathways and greatly expands the opportunities for exploration of biologically relevant chemical space by means of the pseudo‐NP principle.

## Graphical abstract

**The chemical evolution** of polyketide natural products leads to the unprecedented combination of biosynthetically related fragments in arrangements not found in Nature. The pseudo‐natural product grismonone was identified to be a potent inhibitor of Hedgehog signaling – a bioactivity that is not shared by its guiding fragments.

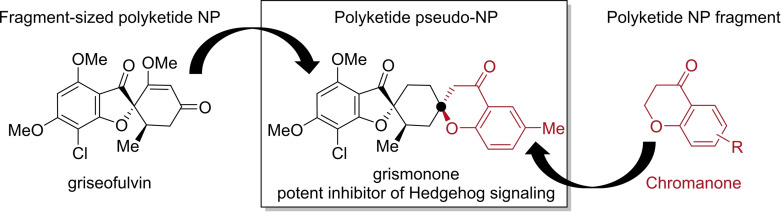

## Introduction

Accessing new chemical entities while retaining biological relevance is a common goal in the design of bioactive compound libraries. Through evolution, Nature has explored biologically relevant chemical space to arrive at natural products (NPs) and has also served as an inspiration for several molecular design concepts,^[1]^ such as complexity to diversity[^2^, ^3^, ^4^] and biology‐oriented synthesis.[^5^, ^6^, ^7^] We have proposed the design principle of pseudo‐natural products[^8^, ^9^, ^10^] (pseudo‐NPs) in which biosynthetically unrelated NP fragments or fragment‐sized NPs are combined to afford scaffolds that resemble NPs but are not accessible through existing biosynthetic pathways. Accordingly, pseudo‐NP collections may explore new regions of biologically relevant chemical space and be enriched with unexpected and/or novel bioactivities.[^11^, ^12^, ^13^, ^14^, ^15^, ^16^, ^17^, ^18^, ^19^, ^20^] This concept may be considered the synthetic analogue of recombination of existing biosynthetic pathways to access novel NP‐like scaffolds, and, thereby, a chemical equivalent to natural evolution of NP structure.^[10]^

Previous pseudo‐NP designs and syntheses were guided by the principle to combine fragments from biosynthetically unrelated NPs to guarantee structural novelty and, in extension, also novel bioactivity. However, the combination of fragments from biosynthetically related natural products in unprecedented arrangements has not been explored. In a sense, this alternative concept may be considered the synthetic extension or diversion of existing biosynthetically related pathways to access novel NP‐like scaffolds. Proof that the synthesis of pseudo‐NPs following this logic may also yield new biologically relevant compound classes with unexpected or novel bioactivity would greatly expand the opportunities for exploration of biologically relevant chemical space by means of the pseudo‐NP principle.

We describe the combination of the biosynthetically related fragment‐sized NP griseofulvin and chromanone fragments in an unprecedented arrangement to arrive at a new class of polyketide pseudo‐NPs. The collection was found to be enriched in bioactivity for the inhibition of the Hedgehog (Hh) signaling pathway. The most active compound, grismonone, is a potent inhibitor of Hh signaling that directly binds to and impedes ciliary entry of Smoothened (SMO) and represents a new SMO antagonist chemotype. The novel bioactivity of grismonone is not observed for either of its NP fragments and is a result of their combination.

## Results and Discussion

**Figure 1:**
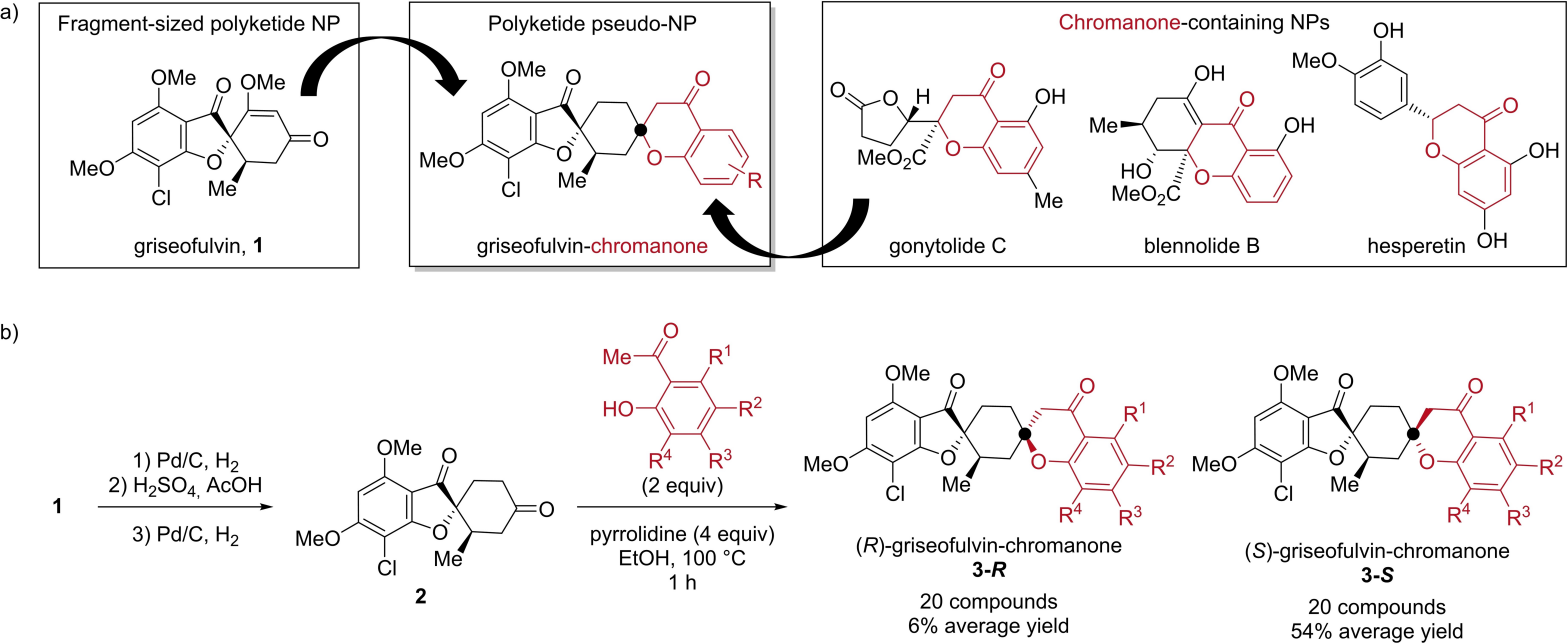
a) Design of griseofulvin‐chromanone (G‐C) pseudo‐natural products through the combination of the biosynthetically related fragment‐sized natural product griseofulvin (**1**) and chromanone fragments. b) Synthesis of the G‐C pseudo‐natural product collection. The configuration of the newly‐formed stereocenter of the G‐Cs were determined by NOESY correlations (see Supporting Information for details).

With the new pseudo‐NP design principle in mind, we aimed to combine the fragment‐sized polyketide NP griseofulvin^[21]^ with biosynthetically related chromanone fragments (Figure 1a). Since the typically employed ‘rule of three’^[22]^ may not be valid for NPs,^[23]^ griseofulvin (**1**) can be considered fragment‐sized.^[24]^ Griseofulvin was first isolated from *Penicillium griseofulvum* in 1939^[25]^ and has been used clinically for the treatment of ringworm since the late 1950’s.^[26]^ In both fungal and mammalian cells, griseofulvin is known to interfere with tubulin polymerization resulting in mitotic arrest.[^27^, ^28^] Chromanone motifs frequently occur in bioactive polyketide NPs^[29]^ and when combined with griseofulvin could provide a new class of griseofulvin‐chromanone (G‐C) polyketide pseudo‐NPs.

In contrast to NP hybridization strategies in which the combination of NPs or NP fragments retain the guiding compounds’ native bioactivities to have polypharmacological effects,^[30]^ the pseudo‐NP concept is designed to explore new areas of biologically relevant chemical space whereby the resulting bioactivities of the pseudo‐NPs are not shared by the guiding NP fragments. Carefully designed modifications of bioactive fragment‐sized NPs or NP fragments may lead to derivatives that do not retain their native bioactivities but are still biologically relevant, that is, have the ability to bind to proteins, and would therefore be suitable combination partners to afford novel bioactivities. This design strategy was applied to griseofulvin which has been reported to lose its native antimitotic activity upon disruption of its enone system.^[31]^ This led us to hypothesize that saturated griseofulvin‐derived fragment **2** (Figure 1b) may be a suitable combination partner with chromanone fragments to explore novel biologically relevant chemical space. The two fragments were designed to be combined via a spirocyclic connection type (Figure 1a). Spirocyclic connectivities can add three‐dimensionality to the resulting scaffolds[^32^, ^33^] and are an underrepresented connectivity in bioactive pseudo‐NPs relative to NPs.^[34]^

Pseudo‐NPs are classified as small molecules that contain at least two NP fragments that are in combinations and/or arrangements that are not found in Nature. Griseofulvin and chromanone motifs are both generated by polyketide biosynthetic pathways;[^35^, ^36^] however, substructure searches in the Dictionary of Natural Products (DNPs) revealed that neither the combination of trimmed griseofulvin and chromanone fragments (Figure S1 in Supporting Information) nor the trimmed scaffold of the G‐C pseudo‐NPs (Figure S2) are found in Nature. Only significantly truncated fragments and scaffolds shared any resemblance to NPs, indicating that the G‐C scaffold represents a novel combination and similarly a novel arrangement of NP fragments not observed in Nature.

The synthesis of the pseudo‐NP collection commenced with the derivatization of griseofulvin (**1**) to saturated derivative **2** over a three‐step sequence (Figure 1b). Compound **2** was a suitable substrate for Kabbe condensation reactions[^37^, ^38^] employing readily available 2‐hydroxyacetophenone derivatives in the presence of a pyrrolidine catalyst. The complexity‐generating reaction proved to be robust and was able to effectively fuse the two fragments with the desired spirocyclic connectivity pattern in a single step. For each reaction, two diastereomers were formed that could be separated by silica chromatography; however, purification was sometimes difficult and resulted in low isolated yields. The minor (**3‐*R***, average of 6 % yield) and major (**3‐*S*** average of 54 % yield) diastereomers were characterized by NOESY correlations and were assigned to have *R* and *S* configurations for the newly generated spirocyclic center, respectively (see the Supporting Information section ‘Stereochemical Determination of Selected G‐Cs’ for representative examples). In total, 21 reactions were attempted resulting in 20 **3‐*R*** and 20 **3‐*S*** griseofulvin‐chromanone (G‐C) pseudo‐NPs. 4‐Nitro‐2‐acetophenone was the only incompatible substrate under the employed conditions.

The scaffold of the G‐C collection is comprised of a novel arrangement of polyketide NP fragments and therefore may lead to unexpected or unprecedented bioactivities. Therefore, to best evaluate these pseudo‐NPs, target‐agnostic cell‐based assays that simultaneously probe multiple targets by monitoring entire biological processes and signaling cascades^[39]^ were employed in a medium throughput manner. Specifically, assays monitoring Hedgehog‐dependent osteoblast differentiation, autophagy, kynurenine production, and natural killer cell‐mediated cancer cell cytolysis rate were employed. Much to our delight, the G‐C collection was enriched with inhibitors of a Hedgehog‐dependent osteoblast differentiation[^40^, ^41^, ^42^, ^43^] while no hits were identified in the other assays. Hedgehog (Hh) signaling is a vital pathway for the regulation of embryonic development and tissue homeostasis and regeneration in adults^[44]^ and is induced by binding of Hh ligands to the membrane receptor Patched1 (PTC1).^[45]^ This relieves PTC1‐mediated inhibition of Smoothened (SMO), a seven‐pass membrane protein, that ultimately leads to activation of transcription of Hh target genes, such as *Gli1* and *Ptch1*, by the transcription factors Glioma‐associated oncogene homolog 2 and 3 (GLI2 and 3).^[46]^ Abnormalities leading to deregulation of the Hh pathway have been linked to cancers such as medulloblastoma and basal cell carcinoma.^[45]^ Therefore, small molecules that modulate the Hh pathway are in high demand as therapeutic options in oncology.^[47]^

Hh signaling can be induced by the Smoothened (SMO) agonist purmorphamine.^[43]^ Activation of Hh signaling induces osteogenesis in C3H10T1/2 cells that, in turn, increases the expression of alkaline phosphatase. Alkaline phosphatase is an early marker of osteoblast differentiation and its activity can be used as an indirect readout of Hh pathway activity upon stimulation with purmorphamine in C3H10T1/2 cells. Upon addition of G‐C pseudo‐NPs to purmorphamine‐stimulated cells, a decreased activity of alkaline phosphatase was observed for several compounds, potentially indicating inhibition of Hh signaling (Table 1). A significant correlation between chromanone configuration and ODA inhibition was observed in which all G‐Cs bearing an (*S*) chromanone configuration (**3‐*S***) had an IC_50_>2 μM while 65 % (13/20) of G‐Cs bearing an (*R*) chromanone configuration (**3‐*R***) had an IC_50_<2 μM. Several compounds had IC_50_ values below 0.2 μM (**3 a‐*R***, **3 c‐*R***, **3 n‐*R***, and **3 p‐*R***). In general, various substitutions patterns at R^2^ and R^3^ retained ODA inhibitory activity. Substitution at R^1^ (**3 b‐*R***), hydrogen bond donors at R^2^ or R^3^ (**3 f‐*R***, **3 k‐*R***, **3 m‐*R***), trifluoromethyl substitution at R^2^ (**3 l‐*R***), and naphthalene derivatives (**3 r‐*R*** and **3 s‐*R***) resulted in significantly decreased inhibitory activities. From these initial medium throughput screenings, **3 c‐*R*** was identified as the most potent compound (ODA IC_50_=0.13±0.05 μM) and was selected for further biological characterization. Most of the G‐Cs did not affect cell viability (36 out of 40 compounds, Table S1), including **3 c‐*R***.

**Table 1:** The activity of G‐C pseudo‐NPs in a Hedgehog‐dependent osteoblast differentiation assay.

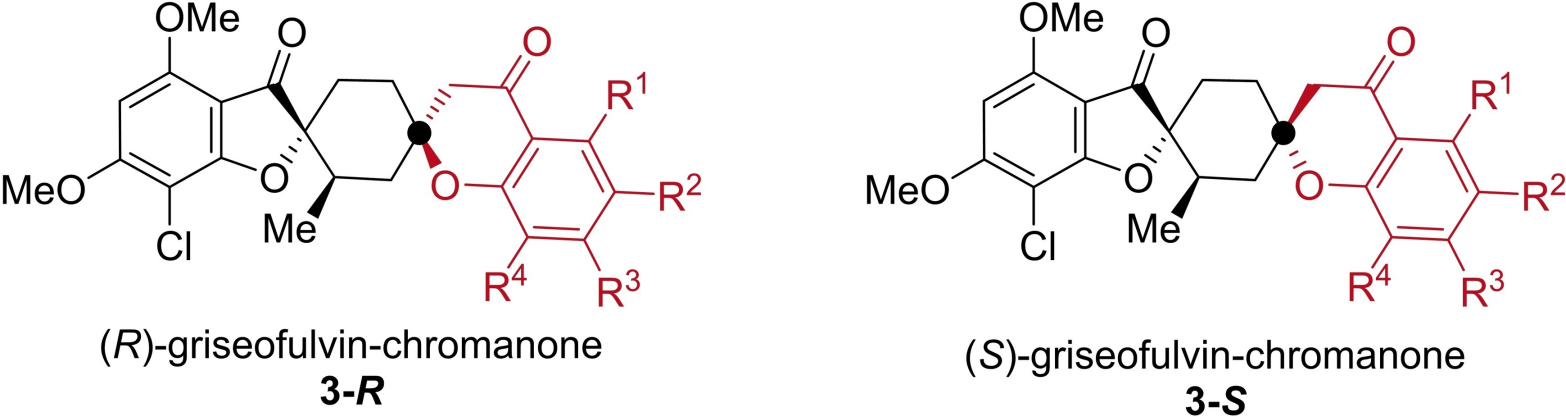									
R^1^	R^2^	R^3^	R^4^		(*R*)‐Config.	ODA IC_50_±SD [μM]^[a]^		(*S*)‐Config.	ODA IC_50_±SD [μM]^[a]^
H	H	H	H		**3 a‐*R***	0.14±0.05		**3 a‐*S***	>10
OMe	H	H	H		**3 b‐*R***	>10		**3 b‐*S***	>10
H	Me	H	H		**3 c‐*R***	0.13±0.05		**3 c‐*S***	9.71±0.26
H	Et	H	H		**3 d‐*R***	0.31±0.08		**3 d‐*S***	5.45±0.85
H	iPr	H	H		**3 e‐*R***	0.52±0.13		**3 e‐*S***	5.66±1.0
H	OH	H	H		**3 f‐*R***	>10		**3 f‐*S***	>10
H	OMe	H	H		**3 g‐*R***	1.59±0.7		**3 g‐*S***	8.67±0.33
H	F	H	H		**3 h‐*R***	0.31±0.13		**3 h‐*S***	>10
H	Cl	H	H		**3 i‐*R***	0.47±0.13		**3 i‐*S***	9.55±0.16
H	Br	H	H		**3 j‐*R***	0.34±0.07		**3 j‐*S***	8.63±1.4
H	NHAc	H	H		**3 k‐*R***	>10		**3 k‐*S***	>10
H	CF_3_	H	H		**3 l‐*R***	8.30±0.63		**3 l‐*S***	6.63±1.2
H	H	OH	H		**3 m‐*R***	2.23±0.1		**3 m‐*S***	>10
H	H	OMe	H		**3 n‐*R***	0.17±0.08		**3 n‐*S***	>10
H	H	NC_4_H_8_	H		**3 o‐*R***	0.96±0.51		**3 o‐*S***	>10
H	Me	Me	H		**3 p‐*R***	0.13±0.05		**3 p‐*S***	>10
H	Cl	Me	H		**3 q‐*R***	0.29±0.06		**3 q‐*S***	>10
C_4_H_4_		H	H		**3 r‐*R***	5.52±2.2		**3 r‐*S***	9.35±0.7
H	H	C_4_H_4_			**3 s‐*R***	5.46±1.5		**3 s‐*S***	3.11±0.8
H	H	OMe	OMe		**3 t‐*R***	1.06±0.4		**3 t‐*S***	2.26±0.5

[a] Half maximum inhibitory concentration in a Hedgehog‐dependent osteoblast differentiation assay (ODA) monitoring the reduction of alkaline phosphatase in a medium throughput manner. Data are mean values±standard deviation (*n*≥3).

**Figure 2:**
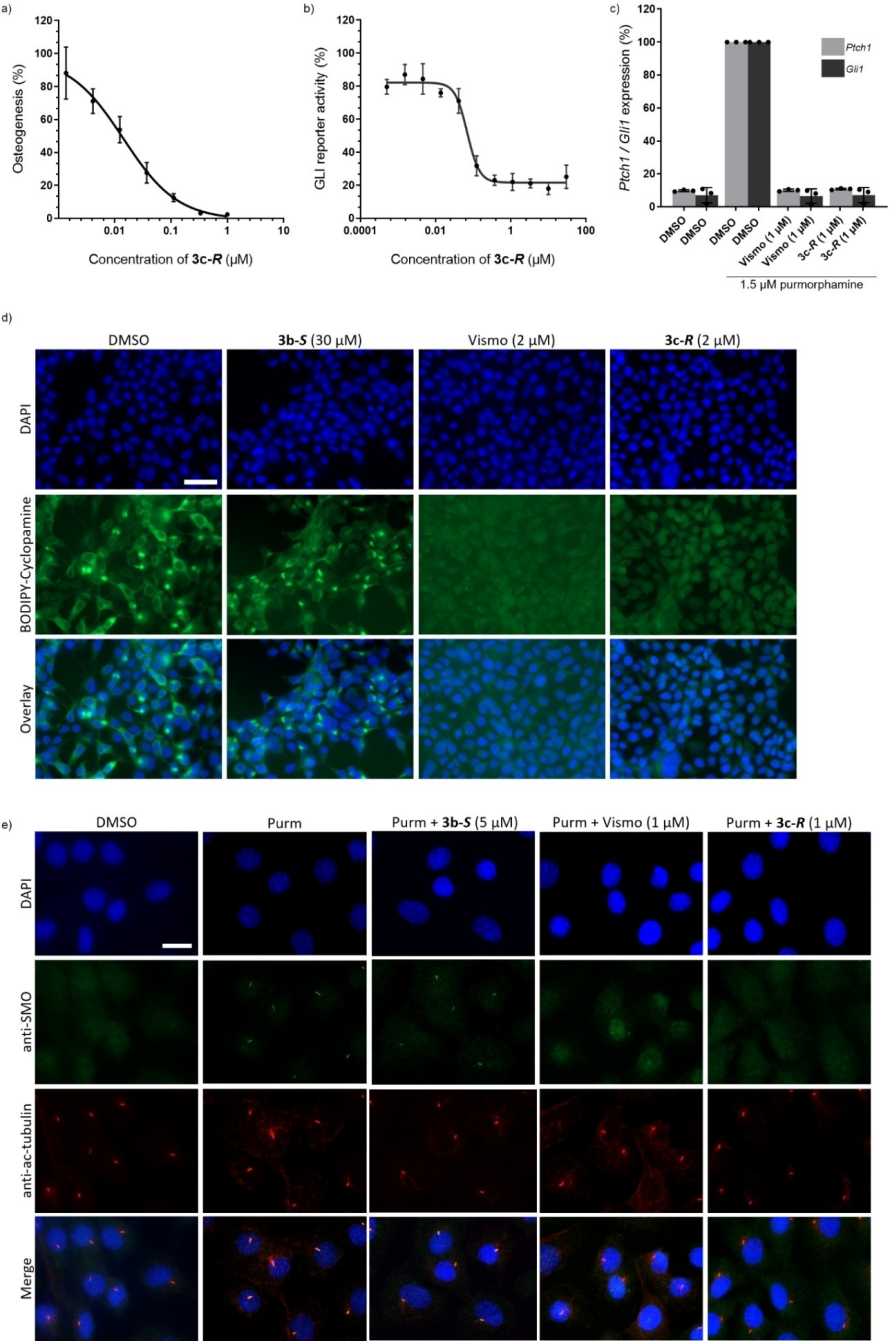
Characterization of compound **3 c‐*R*** for Hh‐signaling inhibition. a) Osteoblast differentiation assay. C3H10T1/2 cells were treated with 1.5 μM purmorphamine together with DMSO as a control or **3 c‐*R*** for 96 h. The activity of alkaline phosphatase was quantified as a measure of Hh pathway activity. Values for cells treated with purmorphamine and DMSO were set to 100 %. Data are mean values±SD of three biological replicates (*n*=3). b) GLI‐responsive reporter‐gene assay. Shh‐LIGHT2 cells were treated with 2 μM purmorphamine together with DMSO as a control or compound **3 c‐*R*** for 48 h. The GLI‐responsive firefly luciferase signal was divided by the control signal of *Renilla* luciferase and the purmorphamine/DMSO control was set to 100 %. Data are mean values±SD and are representative of three biological replicates (*n*=3). c) Expression of the Hh target genes *Ptch1* and *Gli1*. C3H10T1/2 cells were incubated with 1.5 μM purmorphamine and DMSO, 1 μM vismodegib (Vismo), or 1 μM **3 c‐*R*** for 96 h prior to RT‐qPCR. Data are mean values of three biological replicates (*n*=3, N=3). d) Smoothened binding assay. HEK293T cells were transfected with a SMO expressing plasmid and 48 h later cells were fixed and incubated with 5 nM BODIPY‐cyclopamine (green) and treated with either DMSO (negative control), **3 b‐*S*** (30 μM, negative control), vismodegib (2 μM, positive control), or **3 c‐*R*** (2 μM) for 4 h. The cells were stained with 4’,6‐diamidino‐2‐phenylindole (DAPI, blue) to visualize the nuclei. Images are representative of three biological replicates (*n*=3). Scale bar: 40 μm. e) Smoothened ciliary trafficking. NIH/3T3 cells were serum starved for 24 h to induce ciliation before incubating for 24 h with DMSO, purmorphamine (Purm, 2 μM), or co‐incubation with purmorphamine (2 μM) and **3 b‐*S*** (5 μM), vismodegib (Vismo, 1 μM), or **3 c‐*R*** (1 μM). Cilia were then stained with an anti‐acetylated tubulin antibody (anti‐ac‐tubulin, red), SMO with an anti‐SMO antibody (green), and the nucleus with DAPI (blue). Images are representative of three biological replicates (*n*=3). Scale bar: 20 μm.

The activity of compound **3 c‐*R*** was confirmed in a manual osteoblast differentiation assay (ODA) (IC_50_=12±5 nM, Figure 2a). Further experiments were conducted to validate that **3 c‐*R*** affects the Hh signaling pathway. In an orthogonal GLI‐dependent reporter gene assay (GLI RGA) employing Shh‐LIGHT2 cells^[48]^ with a GLI‐responsive firefly luciferase construct,^[49]^ **3 c‐*R*** suppressed the reporter activity with a nanomolar IC_50_ of 48±18 nM (Figure 2b) while not impairing cell viability. Hh pathway modulation was further confirmed by reverse‐transcription quantitative PCR^[41]^ in which **3 c‐*R*** reduced the expression of the Hh target genes *Ptch1* and *Gli1* by 93 % and 89 %, respectively (Figure 2c). These results confirm **3 c‐*R*** is a potent inhibitor of Hh signaling and has comparable cellular activity to the clinically‐approved Hh‐pathway inhibitor and smoothened antagonist vismodegib (ODA IC_50_=11±10 nM, GLI RGA IC_50_=100±12 nM, Figure S3).

Smoothened (SMO) is a validated therapeutic target for affecting Hh signaling.^[50]^ To determine whether **3 c‐*R*** directly interacts with SMO, a displacement assay employing a BODIPY‐labelled derivative of the steroidal alkaloid cyclopamine was used.^[51]^ Cyclopamine is an antagonist of SMO and binds to its heptahelical bundle resulting in the inhibition of Hh signaling.^[52]^ Treatment of HEK293T cells that express SMO with the BODIPY‐labelled cyclopamine derivative and DMSO or with 30 μM of the ODA inactive G‐C **3 b‐*S*** (ODA IC_50_ >10 μM, Figure S4) led to the retention of BODIPY‐related fluorescence (Figure 2d). Conversely, treatment with the cyclopamine derivative and vismodegib (SMO antagonist, 2 μM) or **3 c‐*R*** (2 μM) led to the depletion of BODIPY‐related fluorescence. This indicates the displacement of the bodipy‐cyclopamine probe from SMO and suggests that **3 c‐*R*** directly binds to SMO. Accordingly, the pseudo‐NP **3 c‐*R*** was termed “grismonone”.

The mode of action of grismonone was further investigated by monitoring the localization of SMO in ciliated NIH/3T3 cells (Figure 2e). Upon purmorphamine‐induced activation of the Hh pathway, SMO localized to the primary cilium of the cells. Similar results were obtained with cotreatment of purmorphamine and **3 b‐*S*** (negative control, 5 μM). Conversely, Hh pathway activation and treatment with either vismodegib (positive control, 1 μM) or grismonone (1 μM) led to a decrease in ciliary localization of SMO. Together with the cyclopamine‐displacement assay, these findings demonstrate that grismonone inhibits Hh signaling by directly binding and impeding SMO's ciliary entry.

**Figure 3:**
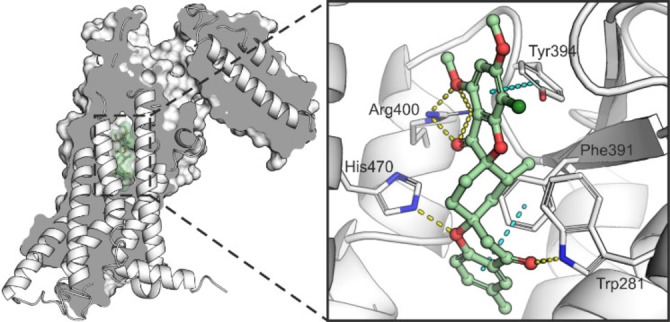
Proposed binding mode of grismonone in complex with SMO. The model is based on induced‐fit molecular docking results with the PDB structure 4 N4 W. Possible H‐bonds and π‐π interactions between grismonone and SMO are represented as yellow and blue dashed lines, respectively. For further comparisons and calculated binding energies of grismonone, SANT‐1 (positive control), and **3 b‐*S*** (negative control) to SMO, see Figure S5.

In order to determine a plausible binding mode of grismonone to SMO, molecular docking techniques were implemented. Several different crystal structures of human SMO (PBD ID: 4JKV, 4N4W, 4O9R, 4QIM, 4QIN, 5L7I and 5V56), most in complex with various antagonists located in the long and narrow binding site of the transmembrane domain, were used for the modelling. The standard docking approach using the rigid structure of the receptor for the calculations but allowing for the flexibility of the ligand did not afford any reasonable binding poses for grismonone with the tested protein structures. Therefore, the computationally demanding yet typically more accurate induced‐fit docking (IFD) approach was employed which allows receptor flexibility in close proximity to the ligand being docked into the protein.[^53^, ^54^, ^55^] The afforded IFD results appeared to be probable, and the obtained poses were re‐scored with a MM‐GBSA protocol estimating the ligand‐receptor binding energy. The ligand poses obtained using the PDB structure of 4N4W gave the best results in which the occupancy of grismonone in the cavity is comparable to the location of the antagonist SANT‐1. In the obtained model, grismonone forms an extensive H‐bond network with SMO, binding to Trp281, His470 and Arg400. The ligand appears to also form possible π‐π interactions with Phe391 and Tyr394 (Figure 3). Docking of the inactive derivative **3 b‐*S*** to SMO using the obtained grismonone pose as a reference and subsequent re‐scoring with MM‐GBSA afforded low binding energy estimate and indicates that **3 b‐*S*** is markedly disadvantaged for binding to the receptor.

Grismonone may represent a new chemotype for SMO inhibition. Retroactively, the web‐based tools SwissTargetPrediction,^[56]^ Similarity Ensemble Approach,^[57]^ and Polypharmacology Browser^[58]^ were used to predict potential targets of grismonone based on chemical similarities to small molecules with known biological targets; however, none of the three tools predicted SMO to be the target of grismonone. In order to acquire a broader overview of the chemical space occupied by known SMO antagonists, a curated set of 615 SMO antagonists was downloaded from the ChEMBL database (see Supporting Information for details) and compared to grismonone by Tanimoto similarity of their Morgan fingerprints as employed in the RDKit (Figure S6a).^[59]^ The range of similarities of ChEMBL SMO antagonists (CSAs) to grismonone is 0.09–0.26 while the median similarity to grismonone is 0.14. These values are comparable to the similarity of a random set 100 compounds from the Enamine Advanced Screening Collection that were compared to themselves (range=0.01–0.39, median similarity=0.15).^[60]^ This demonstrates there is no meaningful chemical similarity between grismonone and CSAs and that grismonone is a novel chemotype for SMO inhibition. Similar conclusions were also obtained when the analysis was conducted with Murcko scaffolds (292 unique scaffolds, Figure S6b) and different fingerprints of a different design (Figure S6a and Figure S6b). The pseudo‐NP could be further differentiated from CSAs by a NP‐likeness score^[61]^ in which grismonone is more NP‐like than 99 % of the compounds in the CSA dataset (Figure S7).

**Figure 4:**
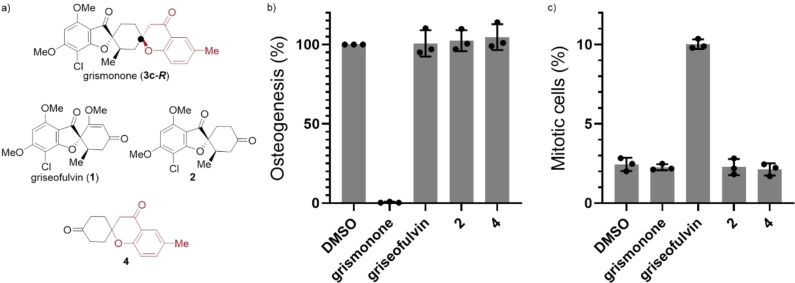
a) Depiction of grismonone (**3 c‐*R***) and its fragments (griseofulvin (**1**), **2**, and **4**). b) Hedgehog‐dependent osteoblast differentiation assay. C3H10T1/2 cells were treated with 1.5 μM purmorphamine together with DMSO as a control or grismonone (10 μM), griseofulvin (10 μM), **2** (10 μM), or **4** (10 μM) for 96 h. The activity of alkaline phosphatase was quantified as a measure of Hh pathway activity. Values for cells treated with purmorphamine and DMSO were set to 100 %. Data are mean values±SD (n=3). c) Quantification of phospho‐histone H3 as a marker of mitotic cells upon treatment of U2OS cells with compounds from Figure 4a for 24 h. Grismonone, griseofulvin, **2**, and **4** were all employed at a concentration of 30 μM. Data are mean values±SD (n=3).

The biological contribution of fragments was assessed by employing grismonone (**3 c‐*R***), the NP griseofulvin (**1**), saturated griseofulvin derivative **2**, and the chromanone fragment of grismonone (**4**) in various biological assays (Figure 4a). The combination of griseofulvin with a chromanone fragment results in grismonone which is a potent inhibitor of Hh‐dependent osteogenesis; however, grismonone's individual fragments (**1**, **2**, and **4**) do not affect Hh‐dependent osteogenesis (Figure 4b). Griseofulvin (**1**) interacts with tubulin which can lead to mitotic arrest.^[28]^ Quantification of mitotic arrest utilizing phospho‐histone H3 as a marker confirmed this hypothesis in which only griseofulvin increased the percentage of mitotic cells (10 % as compared to 2 % for the DMSO control, Figure 4c and Figure S8). Moreover, grismonone, **2**, and **4** did not affect the microtubule cytoskeleton (Figure S8), further validating that they do not target tubulin.

These results indicate that it is not the individual NP fragments but rather the combination of fragments that generates the novel biological activity of grismonone. Furthermore, the strategic modification of the NP griseofulvin lead to derivative **2** that neither retains the NP's native antimitotic activity by itself nor in combination with another NP fragment, that is, grismonone. However, compound **2** still retains biological relevance as its combination with chromanone fragment **4** results in a potent inhibitor of Hh‐dependent osteoblast differentiation. This fragment modification strategy may be a useful approach for reprogramming biologically active chemical matter in future compound collections. Overall, these biological outcomes differ from other hybridization strategies in which chimeric molecules are intended to be polypharmacological by retaining the native bioactivity of their fragments.^[30]^

## Conclusion

In conclusion, a new design principle of combining biosynthetically related NP‐fragments in arrangements not found in Nature was explored. Combination of the fragment‐sized NP griseofulvin and chromanone fragments resulted in a collection of polyketide pseudo‐NPs that is enriched in inhibitors of Hh‐dependent osteoblast differentiation. Further biological and cheminformatic investigations of the most potent compound, grismonone, revealed it is a potent inhibitor of Hedgehog signaling by targeting SMO and represents a new chemotype for SMO inhibition. The individual fragments of grismonone do not inhibit Hedgehog signaling and, therefore, the combination of fragments results in grismonone's bioactivity. These results suggest that combining fragments derived from biosynthetically related NPs in arrangements not found in Nature may expand the repertoire of pseudo‐NP design and facilitate the exploration of biologically relevant chemical space.

## Experimental Section

**Synthesis of the griseofulvin‐chromanone compound collection**: To an oven‐dried microwave vial equipped with a stir bar was added the griseofulvin‐based ketone **2** ^[60]^ (100 mg, 0.31 mmol, 1 equiv). Anhydrous EtOH (1 ml) was added followed by a 2‐hydroxyacetophenone derivative (0.62 mmol, 2 equiv) and pyrrolidine (101 μl, 1.23 mmol, 4 equiv). The vial was flushed with Ar and sealed with the proper cap. The reaction was then heated to 100–130 °C in a microwave for 15–120 min. After cooling to room temperature, the reaction was diluted with 10 ml of DCM and washed with 5 ml of 1 M HCl (aq). The aqueous layer was washed twice more with 5 ml of DCM. The organic layers were combined, dried over Na_2_SO_4_, filtered, and concentrated. Purification could be achieved by silica chromatography (either 15–60 % EtOAc in CyHex or 40–100 % DCM in CyHex) to afford the pure isomers (**3‐*R*** or **3‐*S***).

**Screening of the griseofulvin‐chromanones in a Hedgehog‐dependent osteoblast differentiation assay**: The screening for small molecule inhibitors of the Hh pathway was performed by the Compound Management and Screening Center (COMAS) in Dortmund, Germany in 384 well format. Shortly, 800 cells per well were seeded in 25 μl medium (high glucose DMEM, 10 % heat inactivated fetal calf serum, 1 mM sodium pyruvate, 6 mM L‐glutamine, 100 U/ml penicillin and 0.1 mg/ml streptomycin) and allowed to grow overnight. The next day, compounds were added to a final concentration of 10 μM using the acoustic nanoliter dispenser ECHO 520 (Beckman). After one hour, 10 μl of Purmorphamine in medium were added to a final concentration of 1.5 μM using Multidrop Combi (Thermofisher Scientific); control cells did not receive Purmorphamine. After four days, the cell culture medium was aspirated using the aspiration function of the Elx405 cell washer (Biotek) and 25 μl of a commercial luminogenic ALK substrate (CDP‐Star, Roche) were added. After one hour, luminescence was read. To identify and exclude toxic compounds that also lead to a reduction in the luminescent signal, cell viability measurements were carried out in parallel. The cell viability assay followed the same workflow as the Hh assay, except that only 200 cells per well were seeded. Cell culture medium alone served as control for the cell viability assay. For the measurement of cell viability, 15 μl of CellTiterGlo reagent (Promega) which determines the cellular ATP content were added after aspiration of the medium. Hits were scored as showing at least a 50 % reduction in the luminescent signal in the Hh assay, and a minimum of 80 % cell viability. Dose‐response analysis for hit compounds was done using a three‐fold dilution curve starting from 10 μM. IC_50_ values were calculated using the Quattro software suite (Quattro Research GmbH).

## Conflict of interest

The authors declare no conflict of interest.

1

## Supporting information

As a service to our authors and readers, this journal provides supporting information supplied by the authors. Such materials are peer reviewed and may be re‐organized for online delivery, but are not copy‐edited or typeset. Technical support issues arising from supporting information (other than missing files) should be addressed to the authors.

Supporting Information

## Data Availability

The data that support the findings of this study are available in the manuscript or Supporting Information of this article. The data sets generated and computer code used to analyze the data during the current study are available in the github repository https://github.com/mpimp‐comas/2022_grigalunas_smo_anta.
