# Supplementary material for: Unprecedented Combination of Polyketide Natural Product Fragments Identifies the New Hedgehog Signaling Pathway Inhibitor Grismonone
Source: Chemistry. 2022 Oct 13;28(67):e202202164. doi: 10.1002/chem.202202164 (PMC10091983; doi:10.1002/chem.202202164)
Supplement: Supplementary file 1 — Supporting Information [file CHEM-28-0-s001.pdf]

Chemistry–A European Journal

Supporting Information

Unprecedented Combination of Polyketide Natural Product Fragments Identifies the New Hedgehog Signaling Pathway Inhibitor Grismonone

Michael Grigalunas, Sohan Patil, Adrian Krzyzanowski, Axel Pahl, Jana Flegel, Beate Schölermann, Jianing Xie, Sonja Sievers, Slava Ziegler, and Herbert Waldmann*

Contents

Supporting Information Figures and Tables 2
Biological Experimental Details 11
General Biological Experimental Details 11
Hedgehog-Dependent Osteoblast Differentiation Assay 12
GLI-Responsive Reporter-Gene Assay 14
Reverse Transcription Quantitative PCR (RT-qPCR) 15
Smoothened Binding Assay 16
Smoothened Trafficking 17
Immunocytochemistry 18
Cheminformatic Studies of 3c-*R* and ChEMBL SMO Antagonsits 19
Curation Workflow Details for ChEMBL SMO Antagonists 19
Molecular Modelling 34
Synthetic Experimental Details 35
Synthesis of Griseofulvin-Based Ketone 2 36
General Procedure for Kabbe Condensation 37
Structural Determination of Grismonone (3c-*R*) 58
Stereochemical Determination of Selected G-Cs 65
References 69
NMR Spectra 71
Purity Spectra of Griseofulvin-Chromanones 115

Supporting Information Figures and Tables

The fragment combinations below were investigated via a substructure search in the Dictionary of Natural Products with tautomers included (http://dnp.chemnetbase.com, accessed 06/04/2022).

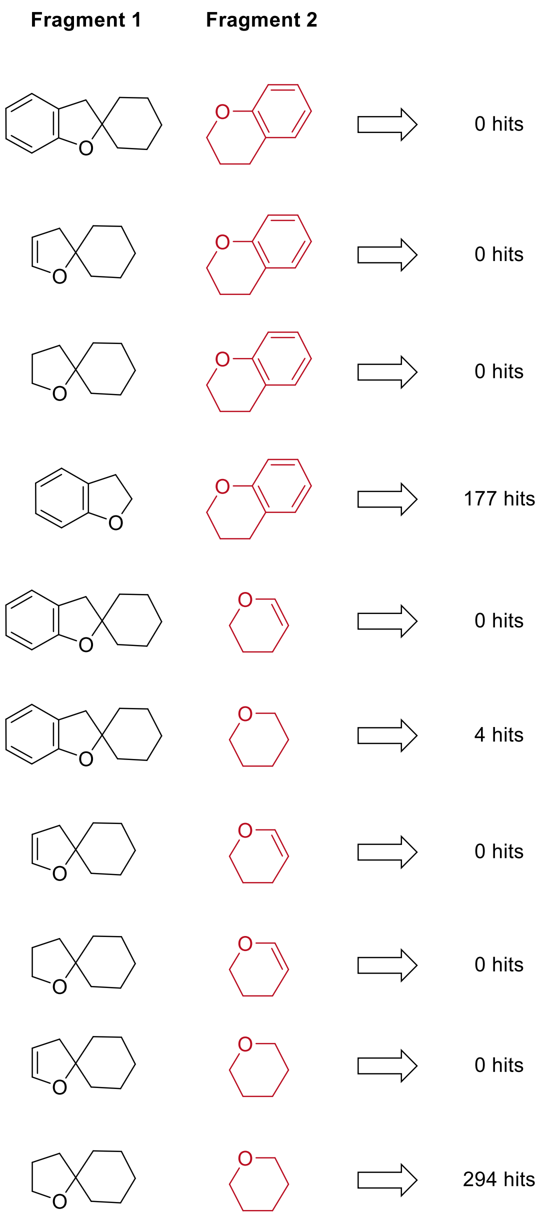

Figure S1. Fragment combination searches within the Dictionary of Natural Products.

The substructures below were investigated in the Dictionary of Natural Products (http://dnp.chemnetbase.com, accessed 06/04/2022) with tautomers included. A second substructure search was conducted in the Collection of Open Natural Products (COCONUT) database (http://coconut.naturalproducts.net, accessed 06/04/2022) using the default substructure search (Ullmann algorithm). For the COCONUT database, each tautomer was manually entered and searched. The number of hits reflects the combination of all tautomers found.

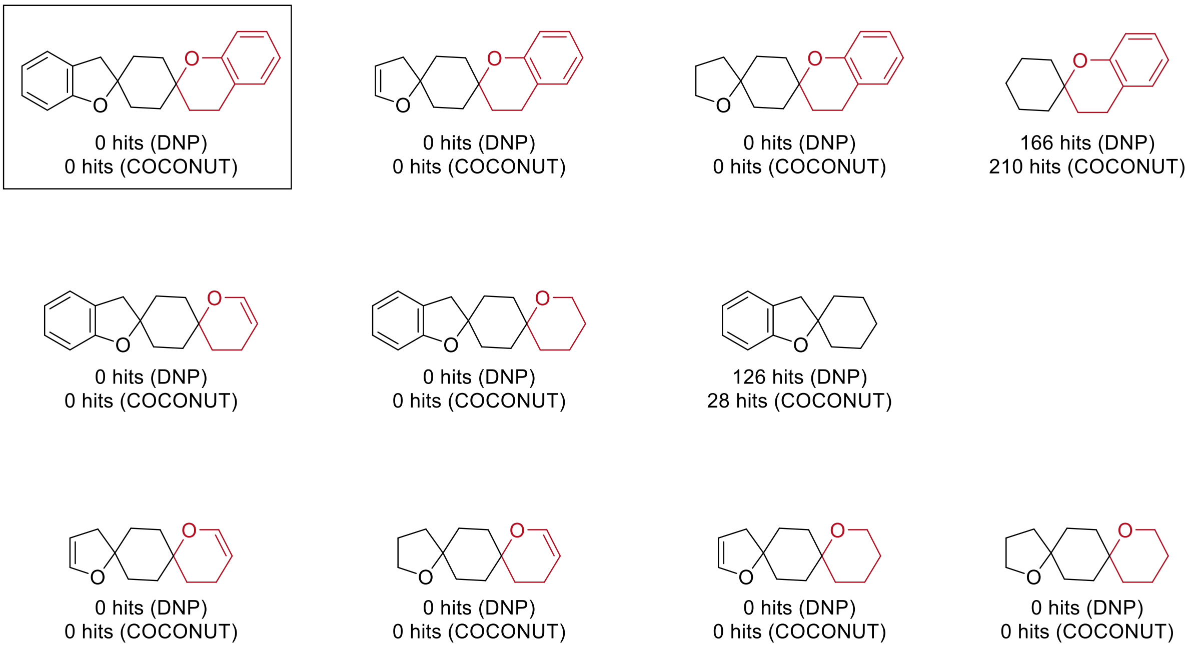

Figure S2. Substructure searches within the Dictionary of Natural Products and the COCONUT database.

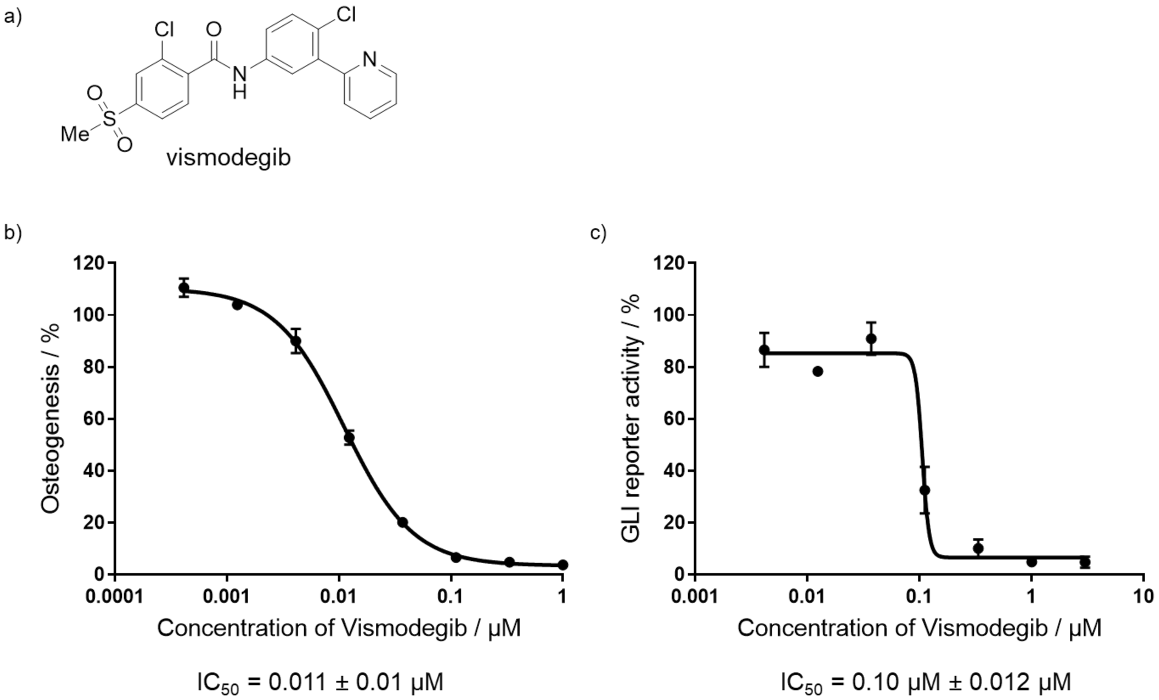

Figure S3. Vismodegib as a control for osteoblast differentiation and GLI-responsive reporter-gene assays. a) Chemical structure of vismodegib. b) Osteoblast differentiation assay. C3H10T1/2 cells were treated with 1.5 µM purmorphamine and vismodegib or DMSO as a control for 96 hours. Alkaline phosphatase was quantified to assess cell differentiation. The alkaline phosphatase levels of cells treated with purmorphamine along with DMSO were set to 100%. Data are mean values ± SD of three biological replicates (*n* = 3, N = 3). c) GLI-responsive reporter-gene assay. Shh-LIGHT2 cells were treated with 2 µM purmorphamine and vismodegib or DMSO as a control for 48 hours. The GLI-responsive firefly luciferase signal was divided by the control signal of the *Renilla* and the DMSO control was set to 100%. Data are representative of three biological replicates (*n* = 3, N = 3).

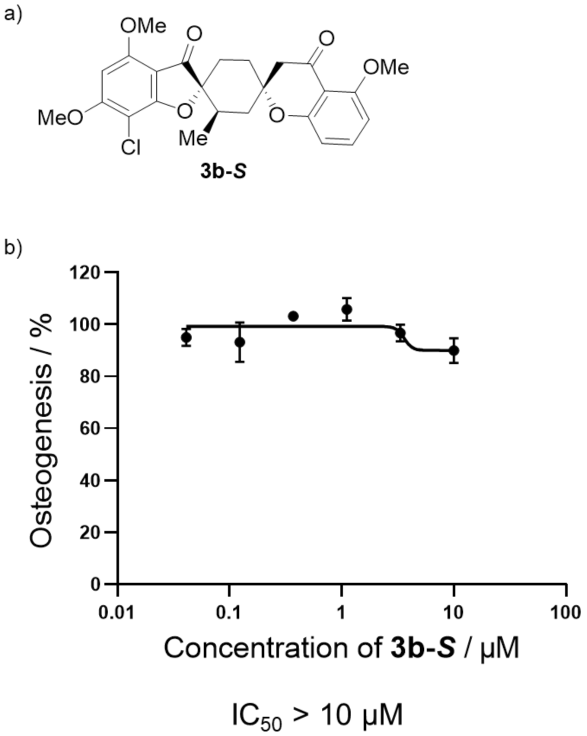

Figure S4. Osteoblast differentiation assay for the negative control **3b-*S***. a) Structure of **3b-*S***. b) C3H10T1/2 cells were treated with 1.5 µM purmorphamine and **3b-*S*** or DMSO as a control for 96 hours. Alkaline phosphatase was quantified to assess cell differentiation. The alkaline phosphatase levels of cells treated with purmorphamine along with DMSO were set to 100%. Data are mean values ± SD of three biological replicates (*n* = 3, N = 3).

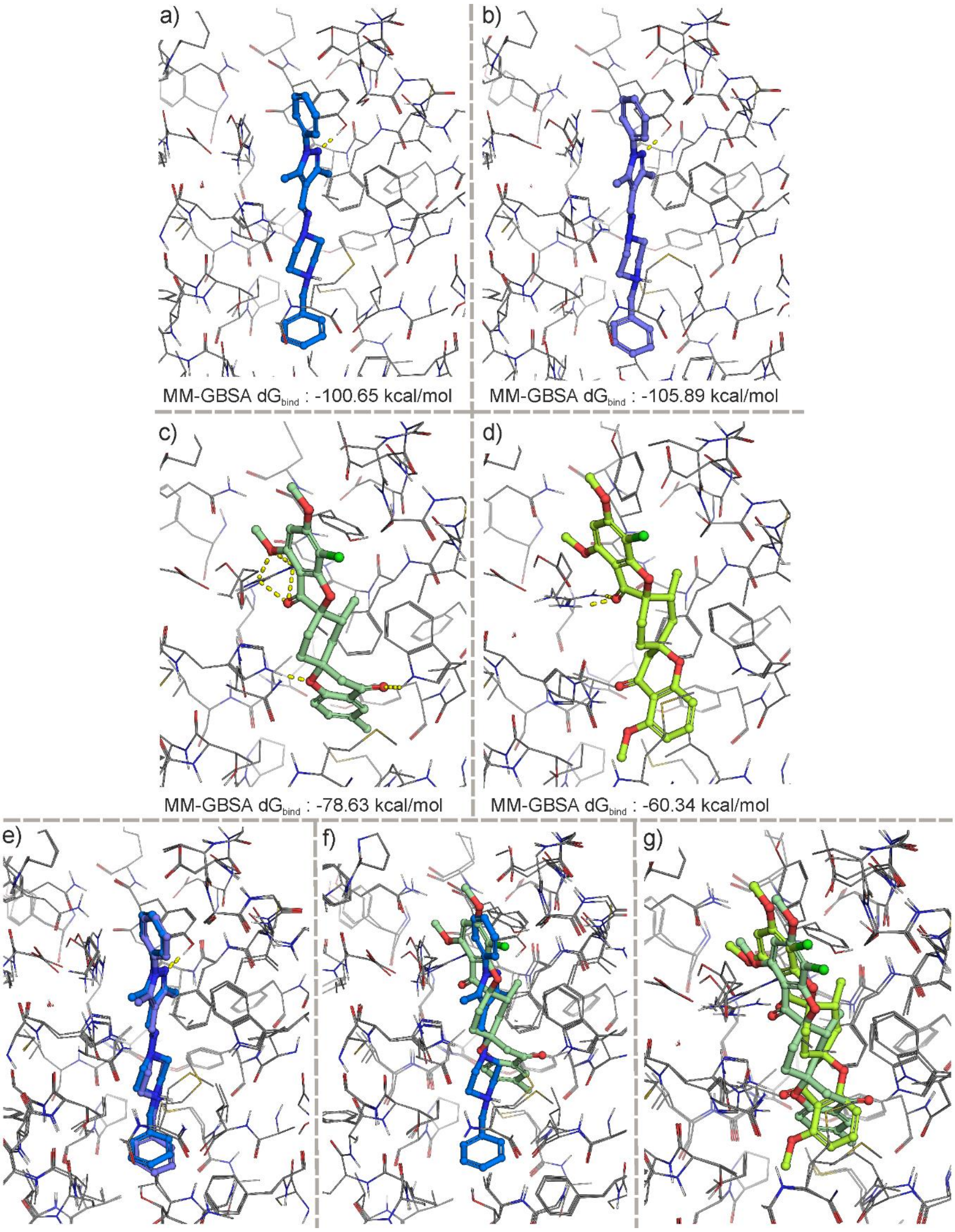

Figure S5. a) Crystal strucutre of SANT-1 in the binding pocket of SMO (PDB ID: 4N4W). b) Redocked SANT-1 in the binding pocket of SMO. c) Proposed binding mode of grismonone (**3c-*R***) in complex with SMO. d) Predicted complex of **3b-*S*** with SMO. e) Superposition of the experimentally optained crystal strucutre of SANT-1 (blue; PDB ID: 4N4W) and the redocked structure (purple). f) Superposition of the experimentally optained crystal strucutre of SANT-1 (blue; PDB ID: 4N4W) and the predicted position of grismonone (pale green). g) Superposition of the predicted models of grismonone (pale green) and **3b-*S*** (bright green) with SMO.

a)

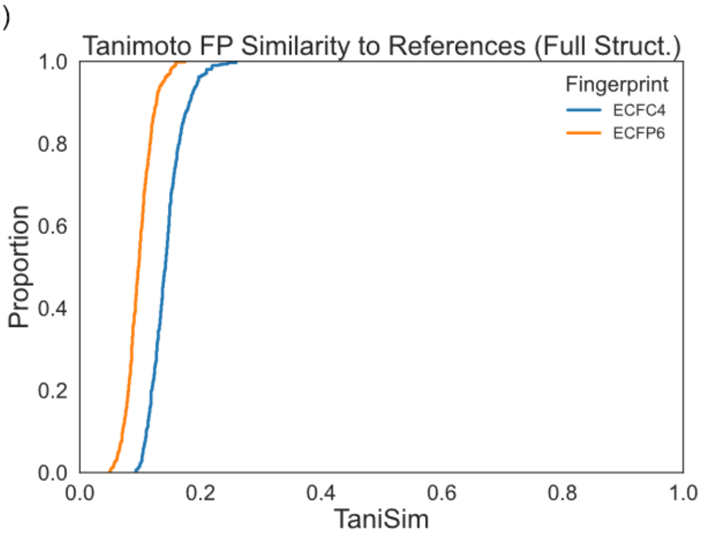

b)

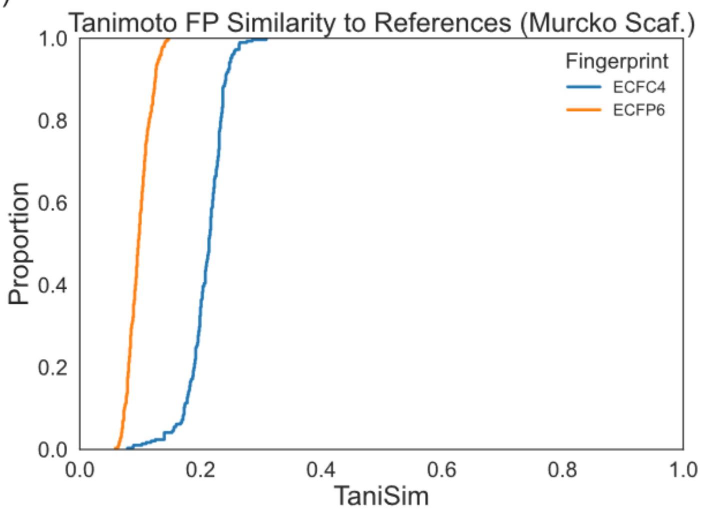

Figure S6. a) Empirical Cumulative Distribution Function plot of the Tanimoto similarities of the Morgan fingerprints of ChEMBL SMO antagonists (615 compounds, as described in the Cheminformatic Studies of **3c-*R*** and ChEMBL SMO Antagonsits section) to **3c-*R*** employing two different fingerprints of different designs (ECFC4, count fingerprint, radius 2 (blue; range = 0.09-0.26; median = 0.14) and ECFP6, bit fingerprint of length 1024, radius 3 (orange; range = 0.05-0.17; median = 0.10)).b) Empirical Cumulative Distribution Function plot of the Tanimoto similarities of the Morgan fingerprints of ChEMBL SMO antagonist Murcko scaffolds (292 unique scaffolds) to the Murcko scaffold of **3c-*R*** employing two different fingerprints of different designs (ECFC4, count fingerprint, radius 2 (blue; range = 0.08-0.31; median = 0.21) and ECFP6, bit fingerprint of length 1024, radius 3 (orange; range = 0.06-0.15; median = 0.10)).

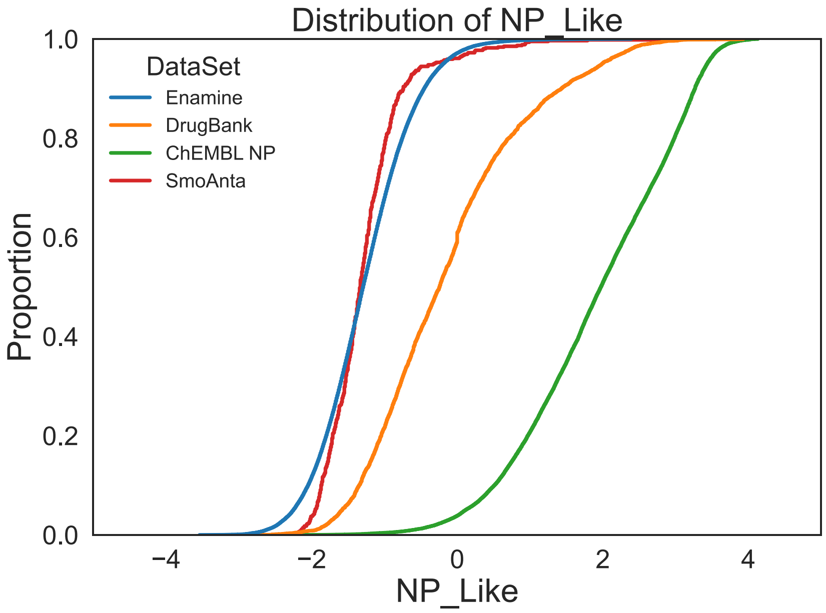

Figure S7. Natural product-likeness score of Enamine Advanced Screening Collection (blue), DrugBank compounds (orange), ChEMBL NPs (green), and ChEMBL SMO antagonists (red). Grismonone (**3c-*R***) has NP-likeness score of +0.94 and is not depicted on the plot.

The atom connectivity of grismonone (**3c-*R***) was compared to ChEMBL SMO antagonists (615 compounds), Enamine Advanced Screening Collection (representing synthetic compounds commonly employed in screening campaigns), DrugBank compounds (representing approved and investigational drugs), and ChEMBL NPs (representing biologically active NPs) via a NP-likeness score.[1] Scores that have a more positive value are considered to be more NP-like. The vast majority of ChEMBL SMO antagonists have a NP-likeness score of < 0 (96%, median = -1.33) and heavily overlap with the Enamine dataset. On the other hand, grismonone is more NP-like (+0.94, which is 7.83 median absolute deviations from the ChEMBL SMO antagonists' median) and lies at the intersection of the DrugBank and ChEMBL NP datasets. Of the ChEMBL SMO antagonists, only 4 compounds (0.7%) have a NP-likeness score greater than grismonone. Therefore, relative to the majority of other SMO antagonists, grismonone is more NP-like. The SMO inhibitors that have a higher NP-likeness score than grismonone are a hybrid molecule combining artemisinin with the warhead of vismodegib[2] (CHEMBL3924243), a NP derivative of a flavanone[3] (CHEMBL4291101), a derivative of the NP cyclopamine (patidegib, CHEMBL538867), and the NP cyclopamine (CHEMBL254129).

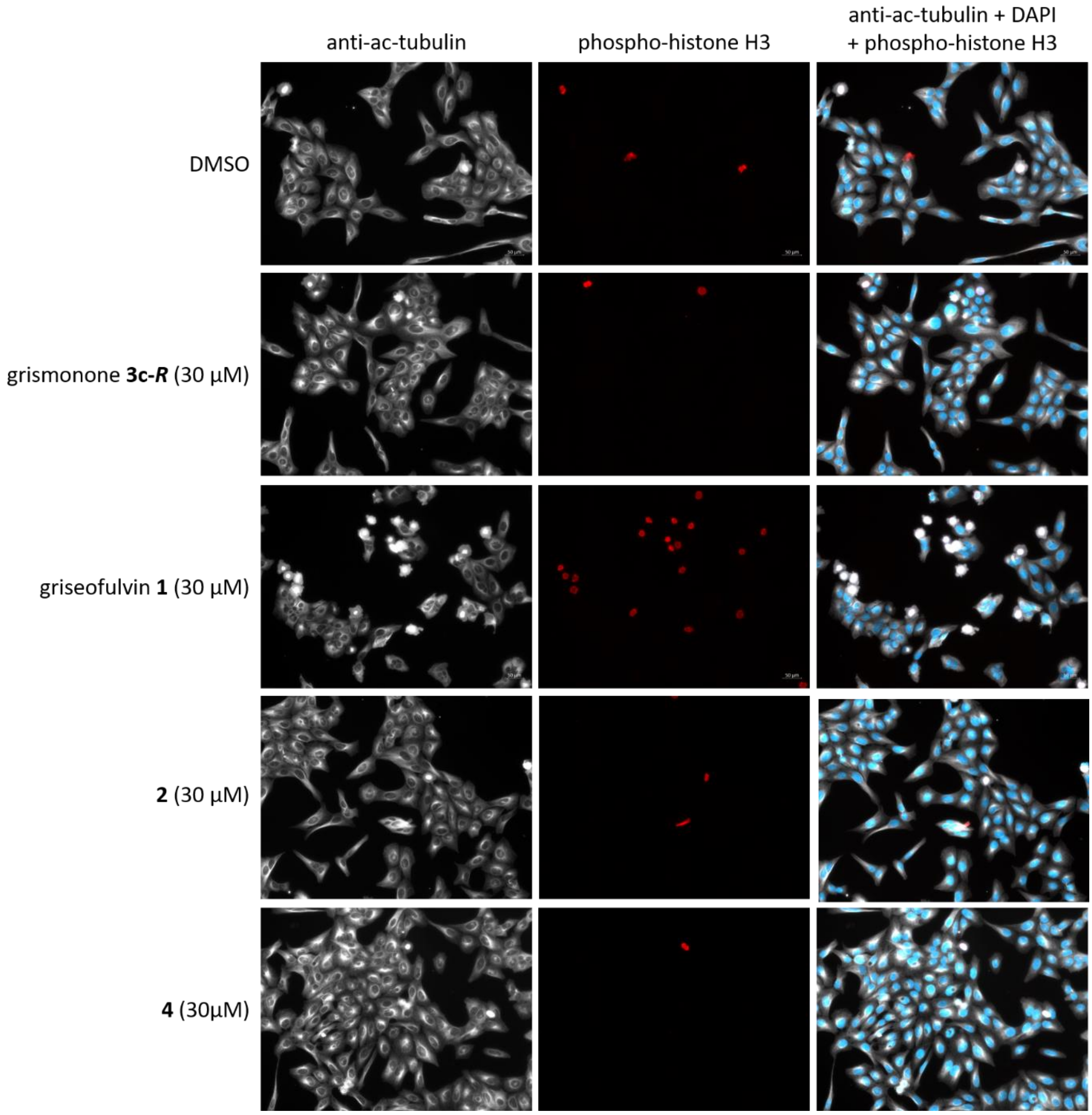

Figure S8. Detection of phospho histone H3 as a marker of mitotic cells. U2OS cells were treated for 24 h with DMSO (negative control), griseofulvin (**1**), grismonone (**3c-*R***), **2**, or **4**. Cells were then fixed, stained with DAPI (blue), anti-tubulin-FITC antibody (white), and phospho-Histone H3 (Ser10) (D2C8) XP® Rabbit mAb Alexa Fluor® 594 Conjugate (red). Images are representative of three biological replicates (*n* = 3). Scale bar: 50 µm.

Table S1. Influence of G-Cs on cell viability in C3H10T1/2 cells. Cell viability was determined at 10 µM and 96 h treatment using CellTiter-Glo Luminescent Cell Viability Assay.

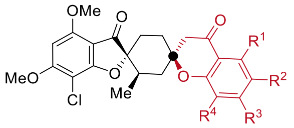

(*R*)-griseofulvin-chromanone
3-R

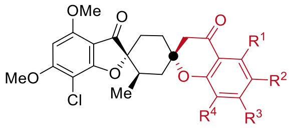

(*S*)-griseofulvin-chromanone
3-S

R^1	R^2	R^3	R^4		(*R*)-Config.	Cell Count (%)[a]		(*S*)-Config.	Cell Count (%)[a]
H	H	H	H		**3a-*R***	103		**3a-*S***	89
OMe	H	H	H		**3b-*R***	92		**3b-*S***	79
H	Me	H	H		**3c-*R***	95		**3c-*S***	113
H	Et	H	H		**3d-*R***	94		**3d-*S***	131
H	iPr	H	H		**3e-*R***	71		**3e-*S***	112
H	OH	H	H		**3f-*R***	59		**3f-*S***	74
H	OMe	H	H		**3g-*R***	96		**3g-*S***	113
H	F	H	H		**3h-*R***	113		**3h-*S***	107
H	Cl	H	H		**3i-*R***	97		**3i-*S***	128
H	Br	H	H		**3j-*R***	94		**3j-*S***	121
H	NHAc	H	H		**3k-*R***	86		**3k-*S***	74
H	CF_3	H	H		**3l-*R***	88		**3l-*S***	135
H	H	OH	H		**3m-*R***	90		**3m-*S***	90
H	H	OMe	H		**3n-*R***	101		**3n-*S***	81
H	H	NC_4H_8	H		**3o-*R***	98		**3o-*S***	92
H	Me	Me	H		**3p-*R***	123		**3p-*S***	110
H	Cl	Me	H		**3q-*R***	104		**3q-*S***	125
C_4H_4		H	H		**3r-*R***	83		**3r-*S***	100
H	H	C_4H_4			**3s-*R***	104		**3s-*S***	94
H	H	OMe	OMe		**3t-*R***	104		**3t-*S***	74

[a] Cell viability of cells that were treated with DMSO was set to 100%. Compounds that reduce cell viability below 80% relative to the DMSO control are considered to impair cell viability.

Biological Experimental Details

General Biological Experimental Details

The compounds were synthesized as described below. DMSO was obtained from Sigma Aldrich (#67685) and all other reagents and their sources are listed in the protocols that follow.

n = x indicates the number of biological replicates. N = x indicates the number of technical replicates.

Cell lines

The C3H10T1/2 cell line (ATCC, CCL-226) is a murine mesenchymal stem cell line. The cells were cultured in Dulbecco's Modified Eagle's medium (DMEM with 4.5 g/L glucose, L-glutamine and 3.7 g/L sodium bicarbonate; PAN Biotech, #P04-03550) supplemented with 10% of Fetal Bovine Serum Australian Origin (FBS, CellSera Australia #AU-FBS/PG, heat inactivated), 1 mM sodium pyruvate (PAN, #P04-43100). Shh-LIGHT2[4] cells are murine fibroblast cells (NIH/3T3 cells) which are stably transfected with a GLI-responsive firefly luciferase reporter plasmid[5] and a pRL-TK constitutive *Renilla* luciferase expression vector (Promega). The cells were cultured in Dulbecco's Modified Eagle's medium (DMEM with 4.5 g/L Glucose, L-glutamine and 3.7 g/L sodium bicarbonate; PAN Biotech, #P04-03550) supplemented with 10% of Fetal Bovine Serum Australian Origin (FBS, CellSera Australia #AU-FBS/PG, heat inactivated), 1 mM sodium pyruvate (PAN, #P04-43100). Additionally, 400 µg/ml Geneticin (Sigma Aldrich, #A1720) and 150 µg/ml Zeocin (InvitroGen, #R25001) were added to the medium as selecting agents. The human kidney cell line HEK293T (ATCC, CRL-11268) was cultured in Dulbecco's Modified Eagle's medium (DMEM with 4.5 g/L Glucose, L-glutamine and 3.7 g/L sodium bicarbonate; PAN Biotech, #P04-03550) supplemented with 10% of Fetal Bovine Serum (FBS, Fisher Scientific, #10270106), 1 mM sodium pyruvate (PAN, #P04-43100) and 1% MEM-non-essential amino acids (PAN, #P08-32100). U2OS cells (Cat#300364, Cell Line Service, Germany) were cultured in DMEM supplemented with 10% FBS, 2 mM L-glutamine, 1 mM sodium pyruvate and non-essential amino acids. All cells were maintained at 37 °C and 5% CO_2 in a humidified atmosphere. Mycoplasma contaminations were checked on a regular basis, and cells were found to be free of contaminations at all times.

Hedgehog-Dependent Osteoblast Differentiation Assay

For assaying signal transduction through the Hedgehog (Hh) pathway, mouse embryonic mesoderm fibroblast C3H10T1/2 cells were used. These multipotent mesenchymal progenitor cells differentiate into osteoblasts upon treatment with the SMO agonist Purmorphamine.[6] During differentiation osteoblast specific genes such as alkaline phosphatase (ALK), which plays an essential role in bone formation, are highly expressed. Activity of ALK can directly be monitored by following substrate hydrolysis yielding a highly luminescent product. Inhibition of the pathway results in reduction of luminescence.

The screening for small molecule inhibitors of the Hh pathway was performed by the Compound Management and Screening Center (COMAS) in Dortmund, Germany in 384 well format. Shortly, 800 cells per well were seeded in 25 µl medium (high glucose DMEM, 10% heat inactivated fetal calf serum, 1 mM sodium pyruvate, 6 mM L-glutamine, 100 U/ml penicillin and 0.1 mg/ml streptomycin) and allowed to grow overnight. The next day, compounds were added to a final concentration of 10 µM using the acoustic nanoliter dispenser ECHO 520 (Beckman). After one hour, 10 µl of Purmorphamine in medium were added to a final concentration of 1.5 µM using Multidrop Combi (Thermofisher Scientific); control cells did not receive Purmorphamine. After four days, the cell culture medium was aspirated using the aspiration function of the Elx405 cell washer (Biotek) and 25 µl of a commercial luminogenic ALK substrate (CDP-Star, Roche) were added. After one hour, luminescence was read. To identify and exclude toxic compounds that also lead to a reduction in the luminescent signal, cell viability measurements were carried out in parallel. The cell viability assay followed the same workflow as the Hh assay, except that only 200 cells per well were seeded. Cell culture medium alone served as control for the cell viability assay. For the measurement of cell viability, 15 µl of CellTiterGlo reagent (Promega) which determines the cellular ATP content were added after aspiration of the medium. Hits were scored as showing at least a 50% reduction in the luminescent signal in the Hh assay, and a minimum of 80% cell viability. Dose-response analysis for hit compounds was done using a three-fold dilution curve starting from 10 µM. IC_{50} values were calculated using the Quattro software suite (Quattro Research GmbH).

In a low-throughput manner, the Hh-dependent osteoblast differentiation activity was determined by adopting the previously described procedure reported by Wu et al.[6] Briefly, 6000 C3H10T1/2 cells per well were seeded in white 96-well plates with a clear flat bottom

(Greiner Bio-One, # 655098) and incubated in 5% CO_2 at 37 °C for 16 h. Next, the cells were treated with 1.5 μM purmorphamine (Cayman Chemical #10009634) and different concentration of the compounds or DMSO (<0.5%) as a control. The plate was sealed with gas permeable membrane and incubated in 5% CO_2 at 37 °C. After 96 h, the cell culture medium was aspirated and 50 μL per well lysis buffer (100mM Tris pH 9.5, 250 mM NaCl, 25 mM $MgCl_2$ and 1% Triton X-100) containing luminogenic the luminogenic alkaline phosphatase (ALP) substrate CDP-Star (Roche, #11685627001) 1:100 dilution was added and incubated for 1 h at room temperature with gentle shaking in dark. Finally, the luminescence signal was measured using the Spark® plate reader (Tecan). The alkaline phosphatase activity of cells that were treated with DMSO and purmorphamine was set to 100%. Calculations of the IC_{50} values were conducted using the GraphPad Prism 9 (GraphPad Software, USA).

GLI-Responsive Reporter-Gene Assay

The GLI-dependent reporter gene assay was performed by utilizing Shh-LIGHT2 cells.[4] Briefly, $2.5x10^4$ Shh-LIGHT2 cells per well were seeded in 96-well plates (Sarstedt, #83.3924) and incubated in 5% CO_2 at 37 °C overnight. The cells were incubated for 48 h with 2 μM purmorphamine to activate Hh signaling and different concentrations of the compounds or DMSO (<0.5%) as a control, in serum-reduced medium (0.5% FBS). Then, the Dual-Luciferase Reporter Assay System (Promega, #E1960), was used to detect the expression and activity of firefly and *Renilla* luciferases using the Spark® plate reader (Tecan). The *Renilla* luciferase signal was used to normalize the firefly luciferase signal. The normalized activity ratio of the cell treated with DMSO and purmorphamine was set to 100%. Calculations of the IC_{50} values were conducted using the GraphPad Prism 9 (GraphPad Software, USA).

Reverse Transcription Quantitative PCR (RT-qPCR)

Sixty thousand C3H10T1/2 cells were seeded into 12-well plates and incubated at 37 °C in 5% CO_2 for 48 hours to achieve 80% cell confluency. The cells were then treated with 1.5 µM purmorphamine to activate Hh signaling, and different concentrations of the compounds or DMSO as control for 96 h. Afterwards, the total RNA was isolated using the RNAeasy Kit (Qiagen, #74104) including DNase digestion step. The RNA concentration was measured using NanoDrop 2000 (Thermo Scientific). The QuantiTect Reverse Transcription Kit (Qiagen #205313) was used to generate cDNA. The relative mRNA amount of the Hedgehog target genes *Ptch1*[7] and *Gli1*[8] and the reference genes *Gapdh* and *Ap3d1* was assessed using SsoAdvanced™ Universal SYBR® Green Supermix, template cDNA, and primers. The SYBR Green signal was detected using the CFX96 Real-Time PCR Detection System (Bio-Rad, Germany) and relative gene expression levels were calculated using the $^{\Delta\Delta}$Ct method[9] with *Gapdh* and *Ap3dl* as reference genes. Gene expression levels in DMSO and purmorphamine-treated samples were set to 100%, whereas *Ptch1 and Gli1* expression levels in compound-treated samples were related to the respective positive control.

Used primers:

Primer	Forward (5’-3’)	Reverse (5’-3’)
Ptch1	CTCTGGAGCAGATTTCCAAGG	TGCCGCAGTTCTTTTGAATG
Gli1	CACCGTGGGAGTAAACAGGCCTTCC	CCAGAGCGTTACACACCTGCCCTTC
Gapdh	CAGTGCCAGCCTCGTC	CAATCTCCACTTTGCCACTG
Ap3dl	CAGAGGGCTCATCGGTACAC	GCCGGAAGTCCAACTTCTCA

Smoothened Binding Assay

The Smoothened binding assay was adopted by previously described procedure.[10] 6 x10^4 HEK293T cells per well were seeded on poly-D-lysine-coated coverslips (Neuvitro, 12 mm, #GG-12-1.5-PDL) placed in a 24-well plate and incubated at 37 °C in 5% CO_2 for 24 h. The cells were transfected with the SMO-expressing plasmid pGEN-mSMO (pGEN-mSmo was a gift from Philip Beachy (Addgene plasmid # 37673; http://n2t.net/addgene:37673 ; RRID:Addgene_37673))[4] in OptiMEM medium using FuGENE® HD transfection reagent (Promega, # E2311) according to the manufacturer's protocol. Cells were incubated at 37 °C in 5% CO_2 for 48h. Then, the cells were washed once with PBS, fixed with 3.7 % paraformaldehyde in PBS for 10 min at room temperature and subsequently treated with PBS containing 10 mM glycine and 0.2% sodium azide for 5 min. The fixed cells were then washed three times with PBS and treated with the compounds, Vismodegib (Selleckchem #1082) and DMSO in DMEM containing 0.5% FBS (assay medium) and 5 nM BODIPY-Cyclopamine S26 (Carbosynth Limited, FB18988) for four hours at room temperature in the dark. After that, cover slips were washed with PBS and incubated for 10 min at room temperature with 1 g/ml 4',6 diamidino-2-phenylindole (DAPI, Sigma Aldrich, Roche, #10236276001) in PBS. Cover slips were then washed again and mounted onto glass slides using Aqua Polymount (Polysciences). Zeiss Observer Z1 microscope (Carl Zeiss, Germany) was used to acquire the images using a Plan-Apochromat 63x/1.40 Oil DIC M27 objective.

Smoothened Trafficking

In 24-well plates, NIH/3T3 cells (2x 10^4 per well) were seeded on cover slips (Carl Roth, 12 mm, #P231.1) and cultured for 24 h at 37°C and 5% CO_2. Cells were then incubated in DMEM containing 0.5 % FBS for 24 h to induce ciliation. Cells were then treated for 24 h in assay medium (DMEM + 0.5 % FBS) with **3c-*R*** (1 µM), **3b-*S*** (5 µM), vismodegib (1 µM) or DMSO as a control. The following day, cells were incubated with purmorphamine (2 µM) in fresh assay medium along with **3c-*R*** (1 µM), **3b-*S*** (5 µM) vismodegib (1 µM), or DMSO for 24 h. Cells were washed with PBS before being fixed in 3.7 % paraformaldehyde for 10 min, permeabilized for 5 min in PBS with 0.3 % Triton-X100, and blocked for 30 min in PBS with 10% FBS. Cells were then incubated overnight at 4 °C with a rabbit anti-N-acetylated tubulin antibody (Cell Signaling Technology # 5335, dilution 1:1000) as a cilia marker and a mouse anti-SMO antibody (Santa Cruz Biotechnology #sc-166685, dilution 1:500). The following day, cells were washed three times with 0.1% Triton X-100 in PBS prior to incubation for 45 min with Alexa Fluor 594-conjugated goat anti-rabbit and Alexa Fluor 488-conjugated donkey anti-mouse antibodies (1:1000 dilutions) and DAPI (0.1 µg/mL). The cover slips were then washed twice with 0.1% Triton X-100 in PBS, once with PBS and mounted onto glass slides using Aqua Polymount (Polysciences). The images were captured using a Plan-Apochromat 63x/1.40 Oil DIC M27 objective on a Zeiss Observer Z1 (Carl Zeiss, Germany).

Immunocytochemistry

5,000 U2OS cells were seeded per well in a black 96-well plate with cover glass bottom (Cellvis, USA (P96-1-N)) and incubated overnight. Cells were treated with compounds or DMSO as a control for 24 h. Cells were then fixed using 3.7% paraformaldehyde in phosphate-buffered saline (PBS) and permeabilized with 0.1% Triton X-100 (in PBS) prior to blocking unspecific binding with 2% bovine serum albumin (BSA) in PBS. Staining with DAPI (#10236276001 from Sigma Aldrich, Germany) to visualize DNA, anti-tubulin-FITC antibody (#F2168 from Sigma Aldrich, Germany), and phospho-Histone H3 (Ser10) (D2C8) XP® Rabbit mAb Alexa Fluor® 594 Conjugate (#8481 from Cell Signaling Technology Europe, Germany) was performed in blocking buffer overnight at 4 C°. Images were acquired using Observer Z1 (Carl Zeiss, Germany) using 40x objective (LD Plan-Neofluar). Images were used to detect phospho-histone H3-positive cells using the Cell Profiler software (version 4.2.1).[11] For automated image analysis, the percentage of phospho-histone H3-positive cells was calculated by considering the total cell count as determined by the DNA stain.

Cheminformatic Studies of 3c-*R* and ChEMBL SMO Antagonsits

The data sets generated during and/or analyzed during the current study are available in the github repository https://github.com/mpimp-comas/2022_grigalunas_smo_anta.

Curation Workflow Details for ChEMBL SMO Antagonists

The ChEMBL wbesite was accessed on 23/03/2022 using version ChEMBL 30. Assays that evaluated either function or binding of the SMO homolog from homosapiens (CHEMBL5971) and the SMO homolog from mus musculus (CHEMBL6080) were selected. For high confidence, only entries that had "confidence label 9" (direct single protein target assigned) were selected. To exclude SMO agonists, only compounds that had IC_{50} values were used. Furthermore, to exclude compounds that do not have significant potentcy, entries that had IC_{50} values ≥ 10 µM were excluded. From the resulting entries, duplicate ChEMBL IDs were deleted resulting in 615 unique ChEMBL IDs. Morgan fingerprints of ChEMBL SMO antagonists were calculated and compared to **3c-*R*** by employing two different fingerprints of different designs (ECFC4, count fingerprint, radius 2 and ECFP6, bit fingerprint of length 1024, radius 3). Additionally, NP-likeness scores were calculated for SMO antagonists and **3c-*R*** (+0.94).

Number	Cpd_Id	ECFC4	ECFP6	NP_Like
1	CHEMBL1084738	0.118	0.086	-1.37
2	CHEMBL4632769	0.143	0.103	-1.73
3	CHEMBL4637222	0.148	0.109	-1.67
4	CHEMBL2160078	0.148	0.12	-1.55
5	CHEMBL2160068	0.159	0.125	-1.42
6	CHEMBL1083915	0.152	0.106	-1.29
7	CHEMBL2160071	0.166	0.117	-1.23
8	CHEMBL2160079	0.195	0.111	-1.12
9	CHEMBL1209455	0.118	0.082	-1.41
10	CHEMBL1086467	0.138	0.096	-1.38
11	CHEMBL4288592	0.134	0.11	-1.44
12	CHEMBL497266	0.114	0.073	-1.2
13	CHEMBL538867	0.127	0.112	1.79
14	CHEMBL2160067	0.161	0.128	-1.6
15	CHEMBL2160074	0.168	0.121	-1.34
16	CHEMBL1084835	0.118	0.084	-1.22
17	CHEMBL1084730	0.126	0.078	-1.12
18	CHEMBL2059865	0.115	0.095	-1.81
19	CHEMBL2059863	0.116	0.097	-1.67
20	CHEMBL2142592	0.135	0.095	-1.75
21	CHEMBL2059864	0.128	0.086	-1.72

22	CHEMBL2160077	0.156	0.105	-1.35
23	CHEMBL2160208	0.109	0.091	-1.2
24	CHEMBL485870	0.114	0.065	-1.08
25	CHEMBL1084734	0.121	0.086	-1.01
26	CHEMBL1209454	0.118	0.08	-1.27
27	CHEMBL4539839	0.152	0.145	-1.04
28	CHEMBL516246	0.218	0.11	-1.52
29	CHEMBL2160072	0.166	0.117	-1.18
30	CHEMBL1084736	0.121	0.086	-1.46
31	CHEMBL1083284	0.152	0.106	-1.33
32	CHEMBL1209189	0.138	0.101	-0.99
33	CHEMBL4278388	0.142	0.11	-1.55
34	CHEMBL1083914	0.118	0.086	-1.4
35	CHEMBL474507	0.148	0.085	-1.45
36	CHEMBL1813106	0.149	0.112	-1.14
37	CHEMBL1085503	0.139	0.102	-1.36
38	CHEMBL1209190	0.124	0.092	-1.21
39	CHEMBL2043435	0.108	0.099	-1.54
40	CHEMBL2043430	0.154	0.094	-1.46
41	CHEMBL2043437	0.111	0.099	-1.66
42	CHEMBL561533	0.142	0.119	-1.21
43	CHEMBL2059859	0.12	0.088	-1.72
44	CHEMBL2031290	0.21	0.1	-0.82
45	CHEMBL2059866	0.121	0.094	-1.74
46	CHEMBL2160070	0.159	0.132	-1.41
47	CHEMBL1084737	0.133	0.086	-1.35
48	CHEMBL1084837	0.125	0.084	-1.45
49	CHEMBL1086045	0.118	0.084	-1.26
50	CHEMBL1086048	0.118	0.084	-1.22
51	CHEMBL471672	0.155	0.087	-1.16
52	CHEMBL2059862	0.134	0.102	-1.76
53	CHEMBL1824915	0.11	0.076	-1.18
54	CHEMBL1083285	0.138	0.096	-1.42
55	CHEMBL2059867	0.128	0.086	-1.79
56	CHEMBL473417	0.147	0.11	-2.12
57	CHEMBL4441529	0.137	0.159	-1.87
58	CHEMBL4441310	0.137	0.159	-1.87
59	CHEMBL1824916	0.11	0.07	-1.44
60	CHEMBL1084731	0.133	0.086	-1.19
61	CHEMBL473896	0.134	0.081	-0.98
62	CHEMBL1209314	0.118	0.094	-1.11
63	CHEMBL4567678	0.137	0.159	-1.87
64	CHEMBL1209251	0.113	0.085	-1.29
65	CHEMBL474503	0.136	0.087	-1.44

66	CHEMBL4472296	0.155	0.116	-0.8
67	CHEMBL1813111	0.131	0.101	-1.59
68	CHEMBL2059871	0.123	0.083	-1.93
69	CHEMBL2059868	0.128	0.101	-1.63
70	CHEMBL1083104	0.108	0.075	-1.5
71	CHEMBL3965352	0.186	0.113	-0.78
72	CHEMBL519219	0.12	0.09	-0.84
73	CHEMBL474893	0.136	0.092	-1.38
74	CHEMBL4159710	0.19	0.098	-0.63
75	CHEMBL1084733	0.139	0.087	-1.28
76	CHEMBL1083739	0.111	0.068	-1.53
77	CHEMBL1083605	0.123	0.08	-1.49
78	CHEMBL1813105	0.124	0.089	-0.9
79	CHEMBL2059870	0.154	0.124	-1.89
80	CHEMBL1209074	0.123	0.081	-1.45
81	CHEMBL1209381	0.118	0.08	-1.26
82	CHEMBL2160076	0.149	0.106	-1.22
83	CHEMBL1083103	0.115	0.069	-1.51
84	CHEMBL1086284	0.118	0.084	-1.26
85	CHEMBL2031277	0.21	0.101	-1.11
86	CHEMBL1824904	0.103	0.053	-1.33
87	CHEMBL495877	0.114	0.066	-1.44
88	CHEMBL514764	0.143	0.094	-1.17
89	CHEMBL2031288	0.207	0.1	-0.97
90	CHEMBL1824905	0.095	0.066	-1.01
91	CHEMBL1813109	0.119	0.106	-1.27
92	CHEMBL1083738	0.137	0.098	-1.24
93	CHEMBL1082387	0.132	0.101	-1.28
94	CHEMBL2059861	0.121	0.095	-1.92
95	CHEMBL1209380	0.118	0.08	-1.29
96	CHEMBL520858	0.106	0.071	-0.91
97	CHEMBL1084836	0.138	0.098	-1.52
98	CHEMBL4517976	0.105	0.131	-1.85
99	CHEMBL3818447	0.168	0.118	-1.81
100	CHEMBL3741651	0.133	0.084	-1.81
101	CHEMBL1082791	0.106	0.071	-1.29
102	CHEMBL2057336	0.119	0.092	-1.72
103	CHEMBL1209132	0.127	0.08	-1.27
104	CHEMBL495608	0.107	0.051	-1.55
105	CHEMBL2043436	0.128	0.109	-1.57
106	CHEMBL1083740	0.144	0.104	-1.33
107	CHEMBL1082396	0.132	0.092	-1.4
108	CHEMBL1813096	0.13	0.099	-0.84
109	CHEMBL2059875	0.113	0.098	-1.71

110	CHEMBL2059869	0.134	0.1	-1.92
111	CHEMBL1209133	0.125	0.082	-1.47
112	CHEMBL1084301	0.108	0.061	-1.78
113	CHEMBL1813107	0.133	0.096	-1.21
114	CHEMBL1813114	0.131	0.11	-0.94
115	CHEMBL497238	0.116	0.077	-1.58
116	CHEMBL1209248	0.118	0.088	-1.32
117	CHEMBL1824917	0.103	0.07	-1.71
118	CHEMBL1824908	0.105	0.062	-1.96
119	CHEMBL1824918	0.11	0.091	-1.36
120	CHEMBL474301	0.139	0.087	-0.98
121	CHEMBL497437	0.116	0.07	-1.53
122	CHEMBL4562943	0.135	0.141	-1.1
123	CHEMBL1824909	0.113	0.067	-1.53
124	CHEMBL2160073	0.159	0.103	-1.37
125	CHEMBL1813110	0.145	0.116	-1.53
126	CHEMBL1824919	0.103	0.056	-1.38
127	CHEMBL2031287	0.195	0.098	-0.81
128	CHEMBL4632598	0.124	0.092	-1.62
129	CHEMBL523255	0.112	0.07	-1.4
130	CHEMBL2043434	0.141	0.116	-1.33
131	CHEMBL2160069	0.159	0.117	-1.26
132	CHEMBL1824920	0.103	0.07	-1.71
133	CHEMBL563928	0.127	0.106	-1.2
134	CHEMBL2059860	0.115	0.086	-1.62
135	CHEMBL1824910	0.113	0.07	-1.54
136	CHEMBL3604612	0.145	0.094	-2.03
137	CHEMBL4444651	0.144	0.158	-1.92
138	CHEMBL1813108	0.126	0.101	-1.37
139	CHEMBL3740447	0.133	0.086	-1.7
140	CHEMBL2059872	0.14	0.103	-2.19
141	CHEMBL2043432	0.16	0.136	-1.1
142	CHEMBL2059873	0.115	0.119	-2.12
143	CHEMBL1824911	0.133	0.078	-1.56
144	CHEMBL2043433	0.157	0.124	-1.41
145	CHEMBL3960082	0.181	0.121	-0.78
146	CHEMBL495875	0.109	0.066	-1.38
147	CHEMBL4532846	0.137	0.155	-1.76
148	CHEMBL4167751	0.137	0.129	-1.78
149	CHEMBL1813095	0.13	0.099	-0.84
150	CHEMBL1824921	0.11	0.073	-1.44
151	CHEMBL1824906	0.097	0.06	-1.1
152	CHEMBL4558043	0.124	0.139	-1.92
153	CHEMBL3978393	0.194	0.119	-0.81

154	CHEMBL3819559	0.158	0.108	-1.91
155	CHEMBL4437383	0.137	0.151	-1.67
156	CHEMBL142972	0.134	0.093	-1.31
157	CHEMBL451194	0.127	0.087	-0.9
158	CHEMBL1209250	0.118	0.088	-1.34
159	CHEMBL4160906	0.137	0.134	-1.9
160	CHEMBL4441183	0.129	0.127	-0.87
161	CHEMBL4586057	0.153	0.156	-1.13
162	CHEMBL1813113	0.142	0.104	-0.53
163	CHEMBL485869	0.109	0.058	-1.06
164	CHEMBL2057337	0.12	0.088	-1.56
165	CHEMBL2031268	0.21	0.101	-1.27
166	CHEMBL1824912	0.113	0.062	-1.53
167	CHEMBL3604610	0.139	0.088	-1.86
168	CHEMBL1209379	0.118	0.079	-1.25
169	CHEMBL1813104	0.17	0.113	-1.18
170	CHEMBL4282915	0.13	0.09	-1.77
171	CHEMBL497534	0.104	0.051	-1.62
172	CHEMBL4593367	0.105	0.13	-1.87
173	CHEMBL562270	0.137	0.096	-1.5
174	CHEMBL2031283	0.204	0.086	-0.71
175	CHEMBL2031289	0.21	0.123	-0.89
176	CHEMBL2043438	0.118	0.096	-1.79
177	CHEMBL1813103	0.174	0.129	-1.21
178	CHEMBL1209249	0.118	0.086	-1.41
179	CHEMBL1824907	0.103	0.049	-1.25
180	CHEMBL4447291	0.124	0.14	-1.88
181	CHEMBL474302	0.136	0.084	-1
182	CHEMBL3979327	0.194	0.128	-0.87
183	CHEMBL3262641	0.185	0.105	-1.85
184	CHEMBL2043431	0.154	0.115	-1.09
185	CHEMBL4450586	0.169	0.115	-0.75
186	CHEMBL1824913	0.113	0.074	-1.5
187	CHEMBL495876	0.114	0.073	-1.54
188	CHEMBL2031098	0.19	0.099	-1.09
189	CHEMBL1082790	0.117	0.076	-1.31
190	CHEMBL4441914	0.149	0.139	-1.01
191	CHEMBL3818034	0.151	0.099	-1.8
192	CHEMBL471881	0.163	0.099	-0.94
193	CHEMBL1824922	0.11	0.07	-1.44
194	CHEMBL1824914	0.113	0.062	-1.53
195	CHEMBL1082967	0.118	0.088	-1.36
196	CHEMBL3909078	0.161	0.104	-0.75
197	CHEMBL3966631	0.144	0.087	-1.38

198	CHEMBL3903125	0.145	0.096	-1.33
199	CHEMBL3944996	0.108	0.056	-1.67
200	CHEMBL3983372	0.151	0.086	-1.43
201	CHEMBL3974732	0.168	0.086	-0.97
202	CHEMBL3939322	0.166	0.109	-1.44
203	CHEMBL3934045	0.146	0.092	-0.85
204	CHEMBL3927351	0.146	0.079	-2.01
205	CHEMBL3898393	0.163	0.089	-1.55
206	CHEMBL3978073	0.145	0.091	-0.71
207	CHEMBL3922084	0.15	0.104	-1.08
208	CHEMBL3955346	0.122	0.071	-1.66
209	CHEMBL3902706	0.137	0.09	-1.17
210	CHEMBL3926840	0.146	0.079	-1.1
211	CHEMBL3986419	0.169	0.115	-1.33
212	CHEMBL3953985	0.13	0.079	-1.48
213	CHEMBL3950917	0.122	0.062	-1.28
214	CHEMBL3954626	0.149	0.096	-1.3
215	CHEMBL3930632	0.127	0.066	-1.24
216	CHEMBL3959845	0.154	0.109	-1.31
217	CHEMBL3927630	0.146	0.1	-0.72
218	CHEMBL3927084	0.164	0.086	-1.17
219	CHEMBL3975414	0.146	0.074	-0.86
220	CHEMBL3944267	0.14	0.114	-1.14
221	CHEMBL3903726	0.155	0.097	-1.37
222	CHEMBL3948829	0.147	0.078	-1.24
223	CHEMBL3975985	0.142	0.071	-0.87
224	CHEMBL3923355	0.172	0.111	-1.21
225	CHEMBL3980924	0.107	0.057	-1.62
226	CHEMBL3894764	0.135	0.084	-0.94
227	CHEMBL3979837	0.126	0.076	-1.24
228	CHEMBL3906492	0.147	0.109	-1.13
229	CHEMBL3970317	0.178	0.1	-1.41
230	CHEMBL3967114	0.158	0.098	-1.92
231	CHEMBL3978301	0.151	0.087	-1.46
232	CHEMBL3925179	0.157	0.086	-1.08
233	CHEMBL3965479	0.161	0.09	-1.34
234	CHEMBL3950710	0.189	0.116	-1.25
235	CHEMBL3942022	0.15	0.099	-1.64
236	CHEMBL3952417	0.155	0.103	-1.13
237	CHEMBL3936743	0.147	0.071	-1
238	CHEMBL3957247	0.147	0.079	-1.32
239	CHEMBL3899187	0.103	0.057	-1.55
240	CHEMBL3933708	0.124	0.082	-1.41
241	CHEMBL3930109	0.145	0.09	-0.74

242	CHEMBL3953389	0.185	0.128	-1.54
243	CHEMBL3969486	0.155	0.109	-1.04
244	CHEMBL3930063	0.13	0.085	-1.75
245	CHEMBL3961874	0.162	0.099	-0.33
246	CHEMBL3910022	0.12	0.069	-1.85
247	CHEMBL3956883	0.156	0.095	-1.22
248	CHEMBL3927593	0.149	0.09	-1.72
249	CHEMBL3965473	0.115	0.062	-1.78
250	CHEMBL3982279	0.128	0.084	-1.31
251	CHEMBL3937323	0.146	0.08	-1.19
252	CHEMBL3948598	0.127	0.075	-1.51
253	CHEMBL3965793	0.169	0.106	-1.12
254	CHEMBL3959946	0.117	0.072	-0.97
255	CHEMBL3986550	0.149	0.093	-1.43
256	CHEMBL3912449	0.147	0.094	-0.87
257	CHEMBL3957159	0.166	0.103	-1.24
258	CHEMBL3983242	0.148	0.096	-1.07
259	CHEMBL3948278	0.147	0.074	-1.24
260	CHEMBL3899393	0.142	0.084	-0.82
261	CHEMBL3959587	0.149	0.101	-1.15
262	CHEMBL3972256	0.142	0.089	-1.5
263	CHEMBL3889599	0.141	0.087	-1.43
264	CHEMBL3976089	0.147	0.101	-0.92
265	CHEMBL3902667	0.138	0.092	-0.81
266	CHEMBL3927397	0.145	0.099	-0.74
267	CHEMBL3896685	0.168	0.105	-0.74
268	CHEMBL3971766	0.169	0.099	-1.07
269	CHEMBL3920191	0.149	0.086	-1.11
270	CHEMBL3942299	0.133	0.075	-1.65
271	CHEMBL3905821	0.161	0.111	-1.24
272	CHEMBL3893223	0.14	0.09	-0.98
273	CHEMBL3986696	0.12	0.063	-1.24
274	CHEMBL3939157	0.142	0.08	-1.28
275	CHEMBL3912445	0.148	0.088	-1.35
276	CHEMBL3909290	0.14	0.121	-1.47
277	CHEMBL3903694	0.174	0.106	-1.09
278	CHEMBL3891390	0.139	0.081	-1.12
279	CHEMBL3940076	0.162	0.1	-1.58
280	CHEMBL3949191	0.162	0.106	-0.52
281	CHEMBL3946402	0.148	0.094	-1.2
282	CHEMBL3133037	0.147	0.094	-0.87
283	CHEMBL3910786	0.14	0.063	-1.03
284	CHEMBL3931348	0.149	0.078	-0.83
285	CHEMBL3889851	0.137	0.081	-0.99

286	CHEMBL3922446	0.146	0.084	-1.01
287	CHEMBL3934557	0.146	0.088	-1.3
288	CHEMBL3958192	0.149	0.078	-0.92
289	CHEMBL3919707	0.152	0.095	-1.3
290	CHEMBL3953149	0.153	0.109	-0.91
291	CHEMBL3942727	0.135	0.078	-1.81
292	CHEMBL3919524	0.149	0.092	-1.29
293	CHEMBL3973520	0.144	0.092	-1.16
294	CHEMBL3922623	0.145	0.079	-1.17
295	CHEMBL3958040	0.135	0.09	-1.41
296	CHEMBL3973612	0.122	0.07	-1.83
297	CHEMBL3971495	0.142	0.083	-0.92
298	CHEMBL3604621	0.129	0.083	-1.89
299	CHEMBL3950687	0.138	0.079	-1.48
300	CHEMBL3929514	0.136	0.077	-1.03
301	CHEMBL3895940	0.161	0.119	-1.34
302	CHEMBL3929057	0.135	0.063	-0.9
303	CHEMBL3921763	0.157	0.106	-1.17
304	CHEMBL3905798	0.137	0.076	-1.44
305	CHEMBL3983876	0.172	0.114	-1.25
306	CHEMBL3962661	0.167	0.105	-0.88
307	CHEMBL3915100	0.138	0.085	-1.08
308	CHEMBL3962643	0.136	0.082	-0.92
309	CHEMBL3915663	0.197	0.129	-1.64
310	CHEMBL3967151	0.143	0.1	-1.82
311	CHEMBL3986486	0.141	0.082	-1.53
312	CHEMBL3967092	0.135	0.082	-1.43
313	CHEMBL3955058	0.16	0.102	-1.26
314	CHEMBL3891481	0.137	0.079	-1
315	CHEMBL3911804	0.149	0.098	-0.82
316	CHEMBL561735	0.134	0.095	-1.38
317	CHEMBL2031245	0.195	0.098	-1.26
318	CHEMBL2043429	0.107	0.104	-1.38
319	CHEMBL2031273	0.197	0.089	-1.03
320	CHEMBL1580265	0.105	0.07	-1.96
321	CHEMBL4440172	0.155	0.116	-0.8
322	CHEMBL474281	0.134	0.093	-1.31
323	CHEMBL1082713	0.118	0.088	-1.37
324	CHEMBL2031291	0.21	0.099	-0.88
325	CHEMBL1615189	0.143	0.092	-2.27
326	CHEMBL474508	0.148	0.085	-1.33
327	CHEMBL551065	0.128	0.095	-1.32
328	CHEMBL4644288	0.132	0.103	-1.97
329	CHEMBL561069	0.099	0.082	-1.52

330	CHEMBL550190	0.127	0.098	-1.52
331	CHEMBL2031267	0.21	0.125	-1.34
332	CHEMBL3819191	0.166	0.105	-1.68
333	CHEMBL4588930	0.144	0.15	-2.22
334	CHEMBL4562342	0.165	0.141	-0.57
335	CHEMBL480889	0.18	0.127	-1.05
336	CHEMBL3604624	0.143	0.104	-1.72
337	CHEMBL3604623	0.151	0.113	-2.03
338	CHEMBL557114	0.128	0.09	-1.04
339	CHEMBL495620	0.102	0.064	-1.66
340	CHEMBL3986017	0.163	0.12	-1.15
341	CHEMBL2160075	0.122	0.094	-1.09
342	CHEMBL4572598	0.161	0.116	-0.63
343	CHEMBL2031295	0.219	0.132	-1
344	CHEMBL3604616	0.145	0.094	-2.07
345	CHEMBL3951963	0.185	0.127	0.89
346	CHEMBL4473275	0.124	0.147	-1.77
347	CHEMBL1813101	0.17	0.117	-1.19
348	CHEMBL3933195	0.181	0.152	0.82
349	CHEMBL4472335	0.151	0.115	-0.88
350	CHEMBL473897	0.134	0.095	-1.06
351	CHEMBL4447164	0.161	0.116	-0.63
352	CHEMBL3604625	0.148	0.11	-1.89
353	CHEMBL2031294	0.219	0.106	-0.88
354	CHEMBL2043439	0.107	0.101	-1.63
355	CHEMBL3819380	0.166	0.118	-1.47
356	CHEMBL564222	0.093	0.092	-1.29
357	CHEMBL561330	0.114	0.089	-1.35
358	CHEMBL3262645	0.172	0.113	-1.74
359	CHEMBL3604622	0.146	0.108	-1.85
360	CHEMBL4473575	0.165	0.141	-0.57
361	CHEMBL3967752	0.176	0.127	-0.84
362	CHEMBL1082386	0.116	0.072	-1.62
363	CHEMBL3817897	0.148	0.103	-1.54
364	CHEMBL1084241	0.118	0.086	-1.45
365	CHEMBL3819639	0.166	0.108	-1.89
366	CHEMBL3968327	0.201	0.116	-1.09
367	CHEMBL4525451	0.144	0.15	-2.14
368	CHEMBL2031284	0.22	0.119	-1.03
369	CHEMBL4276902	0.132	0.092	-1.73
370	CHEMBL3818767	0.127	0.067	-1.84
371	CHEMBL4168846	0.124	0.113	-1.94
372	CHEMBL3924243	0.178	0.142	0.99
373	CHEMBL3980443	0.188	0.114	-0.9

374	CHEMBL4562519	0.161	0.122	-0.68
375	CHEMBL3818148	0.132	0.076	-1.96
376	CHEMBL3941556	0.182	0.118	-1.08
377	CHEMBL4281406	0.128	0.097	-1.37
378	CHEMBL3949509	0.174	0.137	-1.24
379	CHEMBL3262647	0.188	0.124	-1.21
380	CHEMBL3262644	0.172	0.107	-1.69
381	CHEMBL3262632	0.171	0.114	-1.49
382	CHEMBL4527095	0.144	0.15	-2.14
383	CHEMBL3932585	0.181	0.106	-1.14
384	CHEMBL4635021	0.168	0.137	-1.48
385	CHEMBL3971787	0.176	0.129	-0.31
386	CHEMBL4518172	0.148	0.119	-1.71
387	CHEMBL2031282	0.197	0.087	-0.67
388	CHEMBL3817987	0.141	0.084	-1.86
389	CHEMBL2031084	0.197	0.087	-1.22
390	CHEMBL3262639	0.179	0.124	-1.58
391	CHEMBL3126692	0.134	0.096	-2.16
392	CHEMBL4639888	0.121	0.099	-1.98
393	CHEMBL4462158	0.161	0.122	-0.68
394	CHEMBL560932	0.099	0.074	-1.95
395	CHEMBL3818511	0.164	0.106	-1.69
396	CHEMBL3817945	0.139	0.072	-1.72
397	CHEMBL3890559	0.15	0.116	-0.91
398	CHEMBL562811	0.11	0.083	-1.49
399	CHEMBL563112	0.127	0.1	-1.34
400	CHEMBL454984	0.137	0.087	-1.06
401	CHEMBL3262640	0.185	0.12	-1.81
402	CHEMBL3819236	0.137	0.072	-1.73
403	CHEMBL500172	0.145	0.08	-0.99
404	CHEMBL552208	0.103	0.066	-1.59
405	CHEMBL2031292	0.219	0.114	-0.97
406	CHEMBL3818879	0.14	0.076	-1.93
407	CHEMBL3262646	0.193	0.121	-1.3
408	CHEMBL4553897	0.18	0.15	-0.95
409	CHEMBL4583477	0.159	0.118	-0.4
410	CHEMBL2059876	0.113	0.106	-1.64
411	CHEMBL4513409	0.117	0.145	-1.86
412	CHEMBL496013	0.1	0.056	-0.98
413	CHEMBL3262643	0.182	0.106	-1.93
414	CHEMBL3818648	0.139	0.084	-1.5
415	CHEMBL3290331	0.142	0.103	-1.64
416	CHEMBL3262642	0.185	0.107	-1.77
417	CHEMBL184712	0.148	0.105	-2.01

418	CHEMBL1813102	0.167	0.114	-1.21
419	CHEMBL2059874	0.113	0.103	-1.76
420	CHEMBL184721	0.147	0.112	-1.95
421	CHEMBL3818296	0.143	0.094	-1.75
422	CHEMBL4452315	0.144	0.159	-2.02
423	CHEMBL3977846	0.168	0.115	-0.5
424	CHEMBL473892	0.116	0.081	-1.37
425	CHEMBL496604	0.123	0.065	-1.76
426	CHEMBL3262630	0.18	0.116	-1.42
427	CHEMBL4163487	0.127	0.127	-1.91
428	CHEMBL4539606	0.156	0.128	-1.13
429	CHEMBL4470299	0.144	0.149	-2.07
430	CHEMBL3262636	0.192	0.142	-1.28
431	CHEMBL4632537	0.151	0.125	-1.74
432	CHEMBL3976847	0.19	0.119	-1.17
433	CHEMBL2152380	0.151	0.107	0.08
434	CHEMBL2031285	0.197	0.101	-1.44
435	CHEMBL1813099	0.157	0.108	-1.19
436	CHEMBL3262654	0.192	0.136	-1.65
437	CHEMBL4168100	0.125	0.133	-1.87
438	CHEMBL1813098	0.157	0.108	-1.19
439	CHEMBL556427	0.107	0.072	-1.67
440	CHEMBL1813097	0.157	0.108	-1.19
441	CHEMBL3262627	0.188	0.123	-1.35
442	CHEMBL521900	0.13	0.078	-1.87
443	CHEMBL550254	0.103	0.076	-1.68
444	CHEMBL497436	0.116	0.07	-1.53
445	CHEMBL497639	0.116	0.077	-1.58
446	CHEMBL3262648	0.183	0.124	-1
447	CHEMBL4645447	0.154	0.11	-1.57
448	CHEMBL496014	0.1	0.054	-1.35
449	CHEMBL3262633	0.175	0.128	-1.63
450	CHEMBL3604617	0.176	0.119	-1.9
451	CHEMBL4063037	0.123	0.065	-0.29
452	CHEMBL4284509	0.125	0.092	-1.38
453	CHEMBL3262653	0.179	0.123	-1.58
454	CHEMBL550661	0.099	0.081	-1.55
455	CHEMBL563992	0.129	0.108	-1.33
456	CHEMBL2031286	0.196	0.093	-0.93
457	CHEMBL3262651	0.17	0.105	-1.77
458	CHEMBL4471156	0.159	0.118	-0.4
459	CHEMBL3604620	0.133	0.086	-1.88
460	CHEMBL561995	0.128	0.09	-1.21
461	CHEMBL4171049	0.156	0.121	-1.79

462	CHEMBL3262649	0.187	0.124	-1.26
463	CHEMBL496603	0.109	0.05	-1.83
464	CHEMBL3948161	0.188	0.131	-0.99
465	CHEMBL3906154	0.201	0.121	-1.04
466	CHEMBL3966039	0.151	0.093	-1.27
467	CHEMBL3915704	0.125	0.102	-1.01
468	CHEMBL3960935	0.158	0.088	-1.34
469	CHEMBL3977460	0.187	0.101	-1.63
470	CHEMBL3971008	0.139	0.084	-0.64
471	CHEMBL3938334	0.14	0.076	-1.45
472	CHEMBL3951798	0.132	0.074	-0.64
473	CHEMBL3897743	0.149	0.087	-0.99
474	CHEMBL3941852	0.143	0.093	-1.4
475	CHEMBL3969729	0.146	0.083	-1.57
476	CHEMBL3910002	0.148	0.097	-1.07
477	CHEMBL3916441	0.13	0.078	-1.69
478	CHEMBL3973278	0.197	0.107	-1.15
479	CHEMBL3950902	0.168	0.119	-0.22
480	CHEMBL3913172	0.184	0.159	-0.06
481	CHEMBL3979853	0.152	0.113	-0.18
482	CHEMBL3906243	0.135	0.086	-0.69
483	CHEMBL3934007	0.143	0.081	-2.02
484	CHEMBL3892588	0.159	0.101	-0.65
485	CHEMBL3959511	0.156	0.099	-1.19
486	CHEMBL3981990	0.207	0.126	-1.63
487	CHEMBL3944445	0.169	0.099	-1.07
488	CHEMBL3958236	0.142	0.071	-1.04
489	CHEMBL3919359	0.175	0.117	-0.91
490	CHEMBL3906764	0.163	0.103	-1.02
491	CHEMBL3890551	0.172	0.11	-1.11
492	CHEMBL3955601	0.162	0.086	-0.61
493	CHEMBL3967640	0.17	0.105	-1.33
494	CHEMBL3896503	0.16	0.092	-0.89
495	CHEMBL3938550	0.149	0.099	-1.11
496	CHEMBL3959631	0.181	0.119	-0.92
497	CHEMBL3909304	0.163	0.088	-0.51
498	CHEMBL1209315	0.127	0.086	-1.15
499	CHEMBL1209317	0.111	0.098	-0.92
500	CHEMBL1209134	0.127	0.081	-1.07
501	CHEMBL1209316	0.113	0.09	-1.49
502	CHEMBL1209378	0.118	0.104	-1.33
503	CHEMBL4293519	0.123	0.096	-1.54
504	CHEMBL366255	0.136	0.097	-1.85
505	CHEMBL4161601	0.146	0.128	-1.89

506	CHEMBL474892	0.136	0.092	-1.38
507	CHEMBL1813112	0.142	0.104	-0.53
508	CHEMBL568916	0.097	0.067	-1.71
509	CHEMBL559001	0.114	0.107	-1.44
510	CHEMBL3262637	0.173	0.127	-1.27
511	CHEMBL4175623	0.136	0.074	-1.9
512	CHEMBL1600636	0.148	0.122	-2.01
513	CHEMBL4215992	0.13	0.082	-1.02
514	CHEMBL522241	0.105	0.056	-1.72
515	CHEMBL551932	0.105	0.083	-1.8
516	CHEMBL3259848	0.179	0.106	-1.55
517	CHEMBL521558	0.101	0.058	-1.52
518	CHEMBL4641704	0.12	0.108	-1.9
519	CHEMBL551873	0.11	0.082	-1.21
520	CHEMBL4290843	0.139	0.094	-1.59
521	CHEMBL515245	0.115	0.077	-1.24
522	CHEMBL3262638	0.194	0.132	-1.19
523	CHEMBL498017	0.106	0.088	-1.71
524	CHEMBL4450803	0.161	0.145	-0.65
525	CHEMBL4284830	0.129	0.094	-1.77
526	CHEMBL4173721	0.13	0.127	-1.99
527	CHEMBL1813100	0.157	0.108	-1.19
528	CHEMBL497209	0.11	0.07	-1.3
529	CHEMBL4294637	0.126	0.093	-1.4
530	CHEMBL4474558	0.156	0.132	-1.88
531	CHEMBL559874	0.117	0.085	-1.06
532	CHEMBL3262652	0.167	0.113	-1.47
533	CHEMBL2031079	0.219	0.107	-1.41
534	CHEMBL557448	0.093	0.074	-1.41
535	CHEMBL254129	0.137	0.097	2.99
536	CHEMBL4646599	0.128	0.11	-1.69
537	CHEMBL4286296	0.148	0.112	-1.62
538	CHEMBL4279209	0.129	0.094	-1.5
539	CHEMBL4455409	0.155	0.117	-0.66
540	CHEMBL1086141	0.118	0.105	-0.91
541	CHEMBL4164165	0.102	0.063	-1.85
542	CHEMBL498457	0.104	0.052	-1.04
543	CHEMBL423915	0.165	0.1	-1.03
544	CHEMBL497819	0.104	0.051	-1.41
545	CHEMBL497412	0.11	0.078	-1.31
546	CHEMBL4290044	0.23	0.118	0.06
547	CHEMBL523431	0.109	0.067	-1.21
548	CHEMBL4282560	0.13	0.093	-1.5
549	CHEMBL3262629	0.183	0.109	-1.42

550	CHEMBL1084495	0.127	0.087	-1.19
551	CHEMBL473073	0.134	0.09	-1.39
552	CHEMBL4285647	0.259	0.105	0.94
553	CHEMBL4282987	0.133	0.101	-1.37
554	CHEMBL473703	0.165	0.1	-1.03
555	CHEMBL4543531	0.155	0.118	-0.59
556	CHEMBL564987	0.105	0.081	-1.56
557	CHEMBL183630	0.149	0.096	-2.1
558	CHEMBL4172388	0.124	0.119	-1.76
559	CHEMBL4289354	0.132	0.102	-1.55
560	CHEMBL365472	0.148	0.081	-1.66
561	CHEMBL4289775	0.13	0.091	-1.74
562	CHEMBL3604619	0.136	0.093	-1.88
563	CHEMBL4291101	0.235	0.106	1.25
564	CHEMBL4215124	0.174	0.112	-0.74
565	CHEMBL497003	0.1	0.061	-1.42
566	CHEMBL4562011	0.096	0.118	-1.61
567	CHEMBL4286606	0.245	0.105	0.36
568	CHEMBL4467625	0.135	0.152	-1.2
569	CHEMBL4473720	0.151	0.117	-0.94
570	CHEMBL2152364	0.151	0.113	0.24
571	CHEMBL2152370	0.121	0.069	0.39
572	CHEMBL3604611	0.145	0.086	-2.04
573	CHEMBL3262650	0.173	0.104	-1.28
574	CHEMBL3262631	0.173	0.11	-1.5
575	CHEMBL4171870	0.133	0.13	-2.06
576	CHEMBL4176279	0.13	0.129	-1.95
577	CHEMBL2152381	0.151	0.118	-0.14
578	CHEMBL4571341	0.151	0.115	-0.88
579	CHEMBL4169989	0.144	0.121	-1.68
580	CHEMBL4644469	0.123	0.1	-2.02
581	CHEMBL557518	0.093	0.101	-1.35
582	CHEMBL4551454	0.137	0.153	-1.22
583	CHEMBL4168517	0.154	0.118	-1.81
584	CHEMBL4647112	0.124	0.094	-1.45
585	CHEMBL495579	0.11	0.067	-1.03
586	CHEMBL497218	0.112	0.063	-1.46
587	CHEMBL3604615	0.145	0.092	-2.18
588	CHEMBL2152379	0.151	0.111	-0.19
589	CHEMBL2152373	0.153	0.088	0.15
590	CHEMBL497217	0.13	0.075	-1.48
591	CHEMBL497820	0.104	0.058	-1.42
592	CHEMBL516370	0.151	0.12	-1.26
593	CHEMBL2152375	0.138	0.096	0.14

594	CHEMBL2152357	0.146	0.104	0.03
595	CHEMBL142450	0.165	0.1	-1.03
596	CHEMBL4171838	0.143	0.122	-2.1
597	CHEMBL4456292	0.184	0.128	-0.81
598	CHEMBL2152362	0.128	0.088	0.1
599	CHEMBL550191	0.093	0.075	-1.47
600	CHEMBL4632758	0.127	0.088	-1.82
601	CHEMBL4289866	0.245	0.104	0.35
602	CHEMBL4279181	0.259	0.105	0.93
603	CHEMBL2152371	0.16	0.112	0.64
604	CHEMBL4649551	0.124	0.104	-1.75
605	CHEMBL4562945	0.184	0.174	-0.93
606	CHEMBL4159684	0.128	0.126	-1.86
607	CHEMBL2152361	0.151	0.113	0.24
608	CHEMBL2152359	0.156	0.104	0.25
609	CHEMBL2152372	0.162	0.15	0.56
610	CHEMBL4537455	0.155	0.134	-0.59
611	CHEMBL4582942	0.149	0.124	-0.96
612	CHEMBL2152376	0.143	0.087	0.04
613	CHEMBL474894	0.161	0.114	-1.37
614	CHEMBL356310	0.134	0.093	-1.31
615	CHEMBL4633504	0.127	0.096	-1.73

Molecular Modelling

Experimental Details

Computational docking was performed using the Maestro environment, version 12.7, with the Schrödinger suite of software, release 2021-1 (Schrödinger Inc., USA). The protein structures (PBD ID: 4JKV, 4N4W, 4O9R, 4QIM, 4QIN, 5L7I and 5V56) were prepared for docking using the Protein Preparation Wizard (Schrödinger). The protonation states of amino acids were refined with PROPKA at pH set to 7.0, and applying restrained minimization with an OPLS4 force field, setting the heavy atom convergence to 0.3 Å RMSD.[12] Grismonone was docked to the receptor structures using the standard Glide protocol, centering the docking grids around the co-crystallized ligands when possible.[13,14] The afforded results were assessed visually and with the obtained GScore values. As no reasonable poses were found, the ligand was modelled using the *ab initio* induced-fit docking (IFD; Schrödinger) strategy, allowing for the residue refinement with Prime (v6.3) 5 Å within the ligand poses.[15–18] Redocking into structures was performed within 30 kcal/mol of the best structure and within the top 20 structures overall. All afforded ligand poses were re-scored with Prime MM-GBSA, where the solvation model was set to VSGB,[19] the force field was set to OPLS4, protein residue flexibility was allowed within 5 Å of the ligand and the sampling method was set to minimization. Docking of the inactive compound **3b-*S*** into SMO was done through IFD using the best grismonone pose as a restricting reference position and was followed by the re-scoring with Prime MM-GBSA. In order to validate the method, the SMO agonist SANT-1 was redocked into the protein (PDB ID: 4N4W) using the same procedure as was applied for grismonone.

Synthetic Experimental Details

General Experimental Details

All reactions were conducted open to air without precautions to exclude water unless specifically stated. Dry solvents were purchased from Fisher Scientific and/or Acros and used without further treatment. Thin layer chromatography (TLC) was performed on silica coated aluminium plates (Merck 60 F254) and visualization was achieved under UV irradiation (254 nm). Purification of crude products was achieved through automated medium pressure liquid chromatography (MPLC, Grace Reveleris X2) using the indicated solvents. NMR spectra were recorded on Bruker AV 400 Avance III HD (NanoBay), Agilent Technologies DD2, Bruker AV 500 Avance III HD (Prodigy), Bruker AV 600 Avance III HD (CryoProbe) or Bruker AV 700 Avance III HD (CryoProbe) spectrometers. Data is reported in ppm with reference to the used deuterated solvent.[20] Isolated products as well as diastereomers could be assigned based on 2D NMR correlations (^{1}H/^{1}H COSY, ^{1}H/^{1}H NOESY, ^{1}H/^{13}C HSQC, ^{1}H/^{13}C HMBC). High-resolution mass spectrometry (HRMS) was performed on an LTQ Orbitrap mass spectrometer coupled to an Accela HPLC-System (HPLC column: Hypersyl GOLD, 50 mm x 1 mm, particle size 1.9 µm, ionization method: electron spray ionization (ESI)). Microwave reactions were carried out in a CEM Discover SP Activent machine. Compound **4** was synthesized according to Grigalunas et al.[21]

Synthesis of Griseofulvin-Based Ketone 2

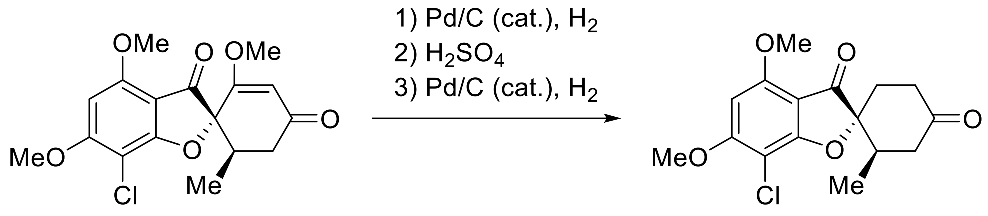

Ketone **2** was synthesized according to Grigalunas et. al.[21]

To an oven-dried three-neck flask was added griseofulvin (10.583 g, 30 mmol), 10% by weight Pd/C (500mg) and anhydrous ethyl acetate (100 ml). The reaction was evacuated and backfilled with H_2 (3x). A balloon of H_2 was attached and the reaction was stirred at 22 °C. After 36 h, the reaction was evacuated and backfilled with Ar (3x). The mixture was filtered through Celite and washed with dichloromethane. The eluent was concentrated and used in the next step without further purification.

To 500 ml flask containing the crude hydrogenated product was added AcOH (200 ml) and an aqueous solution of 2*M* H_2SO_4 (60 ml). The reaction was heated to 80 °C for 16 h. After this time, the reaction was cooled to room temperature and diluted with EtOAc (200 ml) and poured into ice water (200 ml). The organic layer was washed three times with water, dried over Na_2SO_4 and concentrated. The crude product was used in the next step without further purification.

To an oven-dried three-neck flask was added the above crude enone, 10% by weight Pd/C (600mg) and anhydrous ethyl acetate (80 ml) under an atmosphere of Ar. The reaction was evacuated and backfilled with H_2 (3x). A balloon of H_2 was attached and the reaction was stirred at 22 °C. After 6 h, the reaction was evacuated and backfilled with Ar (3x). The mixture was filtered through Celite, washed with dichloromethane, and concentrated. Purification by MPLC (silica gel, 10-36% EtOAc in CyHex) afforded 4.7g (48% over three steps) of **2** as a white solid. ^{1}H NMR (400 MHz, Chloroform-*d*) δ 6.10 (s, 1H), 4.00 (s, 3H), 3.97 (s, 3H), 2.98 (dt, *J* = 15.2, 8.9 Hz, 1H), 2.87 (dd, *J* = 16.0, 12.5 Hz, 1H), 2.54 – 2.42 (m, 3H), 2.20 (dd, *J* = 8.8, 5.2 Hz, 2H), 0.92 (d, *J* = 6.5 Hz, 3H); ^{13}C NMR (101 MHz, $CDCl_3$) δ 209.12, 197.49, 168.07, 164.44, 157.84, 105.51, 97.43, 90.55, 89.24, 57.03, 56.42, 43.96, 38.44, 36.22, 31.18, 15.19; HRMS (ESI) calcd for $C_{16}H_{16}ClO_5$ [M + H] 325.08373 and 327.08078, found 325.08375 and 327.08073; $[\alpha]_D^{20}$ +30.1 (*c* 0.3 , $CHCl_3$).

General Procedure for Kabbe Condensation

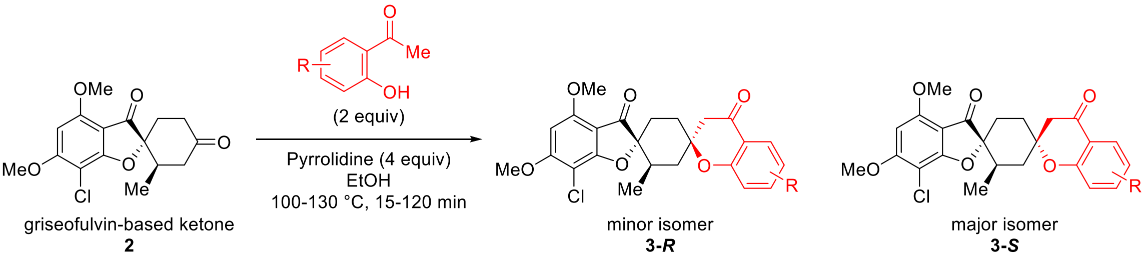

To an oven-dried microwave vial equipped with a stir bar was added the griseofulvin-based ketone **2** (100 mg, 0.31 mmol, 1 equiv). Anhydrous EtOH (1 ml) was added followed by a 2-hydroxyacetophenone derivative (0.62 mmol, 2 equiv) and pyrrolidine (101 ul, 1.23 mmol, 4 equiv). The vial was flushed with Ar and sealed with the proper cap. The reaction was then heated to 100-130 °C in a microwave for 15-120 min. After cooling to room temperature, the reaction was diluted with 10 ml of DCM and washed wash 5 ml of 1 M HCl (aq). The aqueous layer was washed twice more with 5 ml of DCM. The organic layers were combined, dried over Na_2SO_4, filtered, and concentrated. Purification could be achieved by silica chromatography (either 15-60% EtOAc in CyHex or 40-100% DCM in CyHex) to afford the pure isomers. All of the reactions produced two diastereomers. The configuration of the diastereomers could be identified by 2D NOESY NMR.

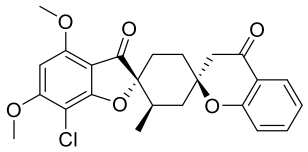

3a-*R*

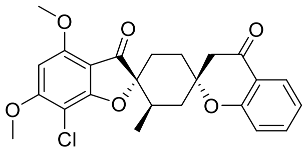

3a-*S*

Following the representative procedure for grisofulvin-chromanones at 100 ºC for 1 h (10:1 dr), 7 mg (5%) of **3a-*R*** and 60 mg (44%) of **3a-*S*** were isolated.

Minor diastereomer **3a-*R***:

^{1}**H NMR** (600 MHz, Chloroform-*d*) δ 7.84 (dd, *J* = 7.8, 1.6 Hz, 1H), 7.48 (ddd, *J* = 8.4, 7.1, 1.8 Hz, 1H), 7.03 – 6.96 (m, 2H), 6.08 (s, 1H), 4.00 (s, 3H), 3.96 (s, 3H), 2.97 (s, 2H), 2.54 – 2.47 (m, 1H), 2.39 (dd, *J* = 13.6, 11.0 Hz, 1H), 2.24 – 2.17 (m, 1H), 2.10 – 1.96 (m, 3H), 1.96 – 1.88 (m, 1H), 0.92 (d, *J* = 6.9 Hz, 3H).

^{13}**C NMR** (151 MHz, $CDCl_3$) δ 197.60, 192.04, 168.06, 164.21, 159.94, 157.87, 136.47, 126.60, 121.03, 120.60, 118.70, 105.61, 97.43, 91.50, 89.11, 80.28, 57.02, 56.43, 45.34, 37.37, 35.01, 30.26, 28.13, 15.32.

HRMS (ESI) calcd for $C_{24}H_{23}ClO_6$ $[M + H]^+$ 443.12559 and 445.12264, found 443.12530 and 445.12238, respectively.

$[\alpha]_D^{20}$ +29.1 (*c* 0.11, $CHCl_3$).

Major diastereomer **3a-*S***:

^{1}**H NMR** (600 MHz, Chloroform-*d*) δ 7.86 (dd, *J* = 7.7, 1.5 Hz, 1H), 7.49 (ddd, *J* = 8.3, 7.1, 1.8 Hz, 1H), 7.04 – 6.96 (m, 2H), 6.07 (s, 1H), 4.00 (s, 3H), 3.96 (s, 3H), 2.80 – 2.71 (m, 2H), 2.70 – 2.62 (m, 1H), 2.42 – 2.35 (m, 1H), 2.28 (td, *J* = 14.1, 4.0 Hz, 1H), 2.21 (dd, *J* = 14.3, 12.9 Hz, 1H), 2.13 – 2.07 (m, 1H), 2.04 (ddd, *J* = 14.3, 4.3, 2.8 Hz, 1H), 1.72 (ddd, *J* = 12.9, 4.0, 2.7 Hz, 1H), 0.77 (d, *J* = 6.8 Hz, 3H).

^{13}**C NMR** (151 MHz, $CDCl_3$) δ 198.42, 192.20, 168.36, 164.19, 159.24, 157.73, 136.39, 126.75, 121.18, 120.85, 118.48, 105.83, 97.18, 91.92, 89.01, 79.35, 57.00, 56.37, 48.25, 37.60, 33.36, 28.96, 28.22, 14.57.

HRMS (ESI) calcd for $C_{24}H_{23}ClO_6$ $[M + H]^+$ 443.12559 and 445.12264, found 443.12532 and 445.12239, respectively.

$[\alpha]_D^{20}$ +34.1 (*c* 0.27, $CHCl_3$).

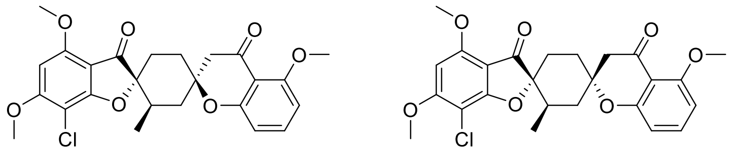

3b-*R* **3b-*S***

Following the representative procedure for grisofulvin-chromanones 100 ºC for 1 h (10:1 dr), 8 mg (6%) of **3b-*R*** and 88 mg (60%) of **3b-*S*** were isolated.

Minor diastereomer **3b-*R***:

^{1}H NMR (500 MHz, Chloroform-*d*) δ 7.37 (t, *J* = 8.3 Hz, 1H), 6.60 (dd, *J* = 8.4, 0.9 Hz, 1H), 6.48 (dd, *J* = 8.5, 0.9 Hz, 1H), 6.07 (s, 1H), 3.99 (s, 3H), 3.96 (s, 3H), 3.90 (s, 3H), 2.94 (d, *J* = 1.7 Hz, 2H), 2.54 – 2.43 (m, 1H), 2.36 (dd, *J* = 13.5, 11.4 Hz, 1H), 2.26 – 2.18 (m, 1H), 2.05 – 1.89 (m, 4H), 0.88 (d, *J* = 6.8 Hz, 3H).

^{13}C NMR (126 MHz, $CDCl_3$) δ 197.70, 190.87, 168.08, 164.16, 161.58, 160.41, 157.81, 136.27, 110.84 (two carbons are present at this peak), 105.58, 103.37, 97.37, 91.51, 89.03, 79.72, 57.01, 56.41, 56.25, 46.58, 37.09, 34.97, 30.12, 28.27, 15.26.

HRMS (ESI) calcd for $C_{25}H_{25}ClO_7$ $[M + H]^+$ 473.13616 and 475.13321, found 473.13572 and 475.13276, respectively.

$[\alpha]_D^{20}$ +35.2 (*c* 0.13, $CHCl_3$).

Major diastereomer **3b-*S***:

^{1}H NMR (500 MHz, Chloroform-*d*) δ 7.35 (t, *J* = 8.3 Hz, 1H), 6.57 (dd, *J* = 8.3, 0.9 Hz, 1H), 6.46 (dd, *J* = 8.4, 0.9 Hz, 1H), 6.05 (s, 1H), 3.96 (s, 3H), 3.92 (s, 3H), 3.86 (s, 3H), 2.74 – 2.64 (m, 2H), 2.64 – 2.55 (m, 1H), 2.37 – 2.28 (m, 1H), 2.23 (td, *J* = 14.0, 3.8 Hz, 1H), 2.15 (dd, *J* = 14.3, 13.0 Hz, 1H), 2.09 – 2.02 (m, 1H), 2.02 – 1.95 (m, 1H), 1.72 – 1.64 (m, 1H), 0.72 (d, *J* = 6.9 Hz, 3H).

^{13}C NMR (126 MHz, $CDCl_3$) δ 198.27, 190.87, 168.17, 164.04, 160.80, 160.39, 157.59, 136.13, 110.91, 110.52, 105.66, 103.57, 96.93, 91.84, 88.89, 78.57, 56.91, 56.27, 56.13, 49.51, 37.29, 33.25, 28.66, 28.11, 14.46.

HRMS (ESI) calcd for $C_{25}H_{25}ClO_7$ $[M + H]^+$ 473.13616 and 475.13321, found 473.13574 and 475.13278, respectively.

$[\alpha]_D^{20}$ +32.3 (*c* 0.17, $CHCl_3$).

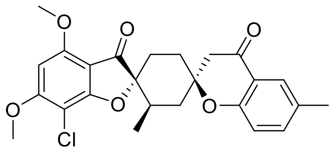

3c-*R*

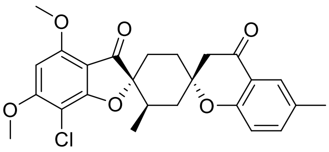

3c-*S*

Following the representative procedure for grisofulvin-chromanones at 130 °C for 15 min (7.2:1 dr), 8 mg (6%) of **3c-*R*** and 93 mg (66%) of **3c-*S*** were isolated.

Minor diastereomer **3c-*R***:

^{1}H NMR (500 MHz, Chloroform-*d*) δ 7.65 - 7.61 (m, 1H), 7.30 (dd, *J* = 8.5, 2.2 Hz, 1H), 6.90 (d, *J* = 8.4 Hz, 1H), 6.07 (s, 1H), 4.00 (s, 3H), 3.96 (s, 3H), 2.94 (s, 2H), 2.52 - 2.44 (m, 1H), 2.37 (dd, *J* = 13.5, 10.9 Hz, 1H), 2.29 (s, 3H), 2.24 - 2.15 (m, 1H), 2.08 - 1.95 (m, 3H), 1.95 - 1.87 (m, 1H), 0.91 (d, *J* = 6.9 Hz, 3H).

^{13}C NMR (126 MHz, $CDCl_3$) δ 197.64, 192.28, 168.04, 164.17, 157.93, 157.84, 137.55, 130.43, 126.17, 120.17, 118.50, 105.58, 97.37, 91.56, 89.04, 80.06, 57.01, 56.42, 45.38, 37.29, 35.00, 30.24, 28.10, 20.52, 15.31.

HRMS (ESI) calcd for $C_{25}H_{25}ClO_6$ $[M + H]^+$ 457.14124 and 459.13829, found 457.14117 and 459.13815, respectively.

$[\alpha]_D^{20}$ +34.6 (*c* 0.15, $CHCl_3$).

Major diastereomer **3c-*S***:

^{1}H NMR (600 MHz, Chloroform-*d*) δ 7.63 (d, *J* = 1.6 Hz, 1H), 7.28 (dd, *J* = 8.5, 2.0 Hz, 1H), 6.89 (d, *J* = 8.4 Hz, 1H), 6.06 (s, 1H), 3.98 (s, 3H), 3.94 (s, 3H), 2.75 - 2.67 (m, 2H), 2.67 - 2.58 (m, 1H), 2.39 - 2.32 (m, 1H), 2.29 - 2.22 (m, 4H), 2.17 (dd, *J* = 14.3, 12.9 Hz, 1H), 2.10 - 2.04 (m, 1H), 2.04 - 1.98 (m, 1H), 1.72 - 1.66 (m, 1H), 0.74 (d, *J* = 6.8 Hz, 3H).

^{13}C NMR (151 MHz, $CDCl_3$) δ 198.34, 192.36, 168.29, 164.12, 157.66, 157.19, 137.38, 130.54, 126.29, 120.39, 118.23, 105.76, 97.09, 91.90, 88.97, 79.07, 56.95, 56.32, 48.24, 37.49, 33.29, 28.85, 28.15, 20.45, 14.52.

HRMS (ESI) calcd for $C_{25}H_{25}ClO_6$ $[M + H]^+$ 457.14124 and 459.13829, found 457.14112 and 459.13815, respectively.

$[\alpha]_D^{20}$ +35.5 (*c* 0.54, $CHCl_3$).

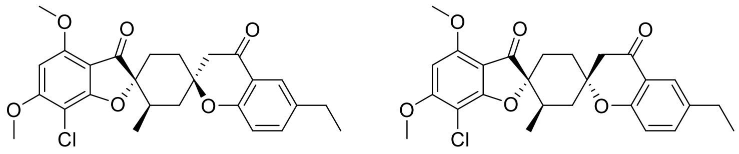

3d-*R* **3d-*S***

Following the representative procedure for grisofulvin-chromanones at 130 °C for 15 min (7.3:1 dr), 10 mg (7%) of **3d-*R*** and 110 mg (76%) of **3d-*S*** were isolated.

Minor diastereomer **3d-*R***:

^{1}H NMR (500 MHz, Chloroform-*d*) δ 7.66 (d, *J* = 2.3 Hz, 1H), 7.33 (dd, *J* = 8.5, 2.4 Hz, 1H), 6.92 (d, *J* = 8.4 Hz, 1H), 6.07 (s, 1H), 4.00 (s, 3H), 3.96 (s, 3H), 2.94 (s, 2H), 2.60 (q, *J* = 7.6 Hz, 2H), 2.53 - 2.44 (m, 1H), 2.37 (dd, *J* = 13.5, 11.0 Hz, 1H), 2.24 - 2.15 (m, 1H), 2.09 - 1.96 (m, 3H), 1.95 - 1.87 (m, 1H), 1.21 (t, *J* = 7.6 Hz, 3H), 0.91 (d, *J* = 6.8 Hz, 3H).

^{13}C NMR (126 MHz, $CDCl_3$) δ 197.64, 192.33, 168.03, 164.16, 158.10, 157.83, 136.84, 136.52, 124.96, 120.21, 118.53, 105.58, 97.37, 91.55, 89.04, 80.06, 57.01, 56.41, 45.36, 37.32, 35.00, 30.25, 28.11, 27.99, 15.64, 15.31.

HRMS (ESI) calcd for $C_{26}H_{27}ClO_6$ $[M + H]^+$ 471.15673 and 473.15394, found 471.15673 and 473.15380, respectively.

$[\alpha]_D^{20}$ +74.8 (*c* 0.13, $CHCl_3$).

Major diastereomer **3d-*S***:

^{1}H NMR (600 MHz, Chloroform-*d*) δ 7.65 (s, 1H), 7.31 (d, *J* = 8.4 Hz, 1H), 6.90 (d, *J* = 8.4 Hz, 1H), 6.06 (s, 1H), 3.97 (s, 3H), 3.93 (s, 3H), 2.76 - 2.66 (m, 2H), 2.67 - 2.53 (m, 3H), 2.35 (td, *J* = 13.4, 3.9 Hz, 1H), 2.24 (td, *J* = 14.1, 3.8 Hz, 1H), 2.16 (t, *J* = 13.6 Hz, 1H), 2.10 - 2.04 (m, 1H), 2.00 (dt, *J* = 14.1, 3.3 Hz, 1H), 1.69 (dt, *J* = 13.2, 3.4 Hz, 1H), 1.19 (t, *J* = 7.6 Hz, 3H), 0.74 (d, *J* = 6.9 Hz, 3H).

^{13}C NMR (151 MHz, $CDCl_3$) δ 198.31, 192.37, 168.25, 164.09, 157.63, 157.33, 136.91, 136.32, 125.04, 120.39, 118.25, 105.73, 97.05, 91.87, 88.95, 79.04, 56.92, 56.29, 48.22, 37.51, 33.27, 28.81, 28.13, 27.90, 15.50, 14.48.

HRMS (ESI) calcd for $C_{26}H_{27}ClO_6$ $[M + H]^+$ 471.15673 and 473.15394, found 471.15666 and 473.15367, respectively.

$[\alpha]_D^{20}$ +38.1 (*c* 0.31, $CHCl_3$).

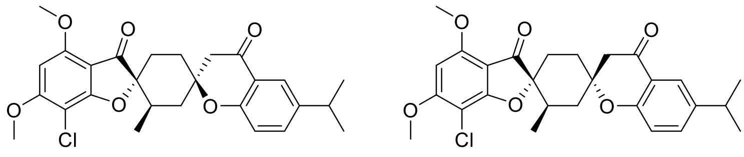

3e-*R* **3e-*S***

Following the representative procedure for grisofulvin-chromanones 100 ºC for 1 h (10:1 dr), 12 mg (8%) of **3e-*R*** and 88 mg (59%) of **3e-*S*** were isolated.

Minor diastereomer **3e-*R***:

^{1}H NMR (600 MHz, Chloroform-*d*) δ 7.69 (d, *J* = 2.4 Hz, 1H), 7.36 (dd, *J* = 8.6, 2.4 Hz, 1H), 6.92 (d, *J* = 8.5 Hz, 1H), 6.07 (s, 1H), 4.00 (s, 3H), 3.96 (s, 3H), 2.94 (s, 2H), 2.87 (hept, *J* = 7.1 Hz, 1H), 2.52 – 2.44 (m, 1H), 2.38 (dd, *J* = 13.6, 11.0 Hz, 1H), 2.24 – 2.17 (m, 1H), 2.07 – 1.97 (m, 3H), 1.95 – 1.88 (m, 1H), 1.23 (d, *J* = 6.9 Hz, 6H), 0.91 (d, *J* = 6.8 Hz, 3H).

^{13}C NMR (151 MHz, $CDCl_3$) δ 197.63, 192.37, 168.06, 164.18, 158.17, 157.85, 141.52, 135.23, 123.55, 120.17, 118.51, 105.61, 97.42, 91.56, 89.08, 80.07, 57.01, 56.42, 45.37, 37.38, 35.03, 33.40, 30.28, 24.06, 24.05, 15.32.

HRMS (ESI) calcd for $C_{27}H_{29}ClO_6$ $[M + H]^+$ 485.17254 and 487.16959, found 485.17205 and 487.16918, respectively.

$[\alpha]_D^{20}$ +54.4 (*c* 0.15, $CHCl_3$).

Major diastereomer **3e-*S***:

^{1}H NMR (600 MHz, Chloroform-*d*) δ 7.69 (d, *J* = 2.4 Hz, 1H), 7.35 (dd, *J* = 8.5, 2.4 Hz, 1H), 6.91 (d, *J* = 8.5 Hz, 1H), 6.07 (s, 1H), 3.98 (s, 3H), 3.94 (s, 3H), 2.85 (hept, *J* = 6.9 Hz, 1H), 2.77 – 2.68 (m, 2H), 2.68 – 2.60 (m, 1H), 2.36 (td, *J* = 13.4, 4.1 Hz, 1H), 2.25 (td, *J* = 14.1, 4.0 Hz, 1H), 2.17 (dd, *J* = 14.3, 12.9 Hz, 1H), 2.10 – 2.05 (m, 1H), 2.01 (ddd, *J* = 14.3, 4.3, 2.8 Hz, 1H), 1.69 (ddd, *J* = 12.9, 3.9, 2.7 Hz, 1H), 1.21 (d, *J* = 7.0 Hz, 6H), 0.75 (d, *J* = 6.8 Hz, 3H).

^{13}C NMR (151 MHz, $CDCl_3$) δ 198.37, 192.46, 168.29, 164.12, 157.66, 157.41, 141.60, 135.07, 123.65, 120.35, 118.24, 105.77, 97.09, 91.93, 88.96, 79.06, 56.94, 56.32, 48.25, 37.60, 33.32, 33.30, 28.82, 28.18, 23.98, 23.97, 14.52.

HRMS (ESI) calcd for $C_{27}H_{29}ClO_6$ $[M + H]^+$ 485.17254 and 487.16959, found 485.17204 and 487.16908, respectively.

$[\alpha]_D^{20}$ +32.3 (*c* 0.53, $CHCl_3$).

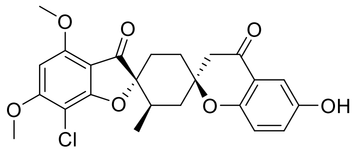

3f-*R*

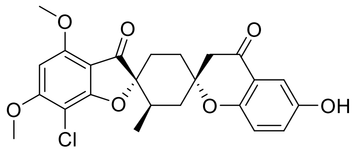

3f-*S*

Following the representative procedure for grisofulvin-chromanones at 130 ºC for 15 min (7:1 dr), 12 mg (9%) of **3f-*R*** and 100 mg (71%) of **3f-*S*** were isolated.

Minor diastereomer **3f-*R***:

^{1}H NMR (500 MHz, Chloroform-*d*) δ 7.30 (d, *J* = 3.1 Hz, 1H), 7.09 (dd, *J* = 8.9, 3.2 Hz, 1H), 6.91 (d, *J* = 8.9 Hz, 1H), 6.07 (s, 1H), 5.54 (s, 1H), 4.00 (s, 3H), 3.95 (s, 3H), 2.93 (s, 2H), 2.51 - 2.42 (m, 1H), 2.36 (dd, *J* = 13.6, 10.8 Hz, 1H), 2.23 - 2.13 (m, 1H), 2.09 - 1.94 (m, 3H), 1.94 - 1.85 (m, 1H), 0.91 (d, *J* = 6.8 Hz, 3H).

^{13}C NMR (126 MHz, $CDCl_3$) δ 197.80, 192.40, 168.04, 164.26, 157.89, 154.23, 149.98, 125.13, 120.55, 119.97, 110.83, 105.54, 97.36, 91.62, 89.06, 80.03, 57.03, 56.39, 45.33, 37.28, 35.01, 30.17, 28.05, 15.33.

HRMS (ESI) calcd for $C_{24}H_{23}ClO_7$ $[M + H]^+$ 459.12051 and 461.11756, found 459.12038 and 461.11738, respectively.

$[\alpha]_D^{20}$ +33.6 (*c* 0.12, $CHCl_3$).

Major diastereomer **3f-*S***:

^{1}H NMR (600 MHz, Chloroform-*d*) δ 7.38 (d, *J* = 3.1 Hz, 1H), 7.09 (dd, *J* = 8.9, 3.1 Hz, 1H), 6.90 (d, *J* = 8.9 Hz, 1H), 6.63 (s, 1H), 6.06 (s, 1H), 3.98 (s, 3H), 3.93 (s, 3H), 2.79 - 2.70 (m, 2H), 2.68 - 2.58 (m, 1H), 2.36 (td, *J* = 13.5, 4.2 Hz, 1H), 2.24 (td, *J* = 14.1, 4.0 Hz, 1H), 2.16 (dd, *J* = 14.3, 12.9 Hz, 1H), 2.12 - 2.07 (m, 1H), 2.05 - 2.00 (m, 1H), 1.71 (ddd, *J* = 13.0, 3.9, 2.7 Hz, 1H), 0.75 (d, *J* = 6.8 Hz, 3H).

^{13}C NMR (151 MHz, $CDCl_3$) δ 198.77, 193.00, 168.37, 164.30, 157.77, 153.41, 150.52, 125.21, 120.79, 119.66, 111.06, 105.77, 97.10, 92.10, 88.99, 79.06, 57.00, 56.29, 48.13, 37.44, 33.31, 28.84, 28.16, 14.55.

HRMS (ESI) calcd for $C_{24}H_{23}ClO_7$ $[M + H]^+$ 459.12051 and 461.11756, found 459.12051 and 461.11756, respectively.

$[\alpha]_D^{20}$ +34.7 (*c* 0.35, $CHCl_3$).

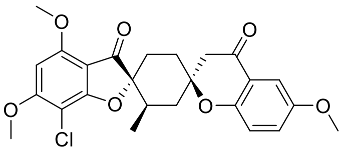

3g-*R*

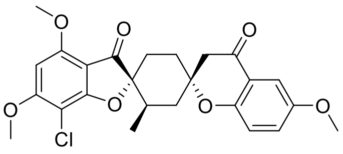

3g-*S*

Following the representative procedure for grisofulvin-chromanones at 130 °C for 15 min (7:1 dr), 9 mg (6%) of **3g-*R*** and 89 mg (61%) of **3g-*S*** were isolated.

Minor diastereomer **3g-*R***:

^{1}H NMR (700 MHz, Chloroform-*d*) δ 7.28 (d, *J* = 3.2 Hz, 1H), 7.10 (dd, *J* = 9.0, 3.2 Hz, 1H), 6.93 (d, *J* = 9.0 Hz, 1H), 6.07 (s, 1H), 3.99 (s, 3H), 3.96 (s, 3H), 3.79 (s, 3H), 2.94 (s, 2H), 2.51 - 2.44 (m, 1H), 2.37 (dd, *J* = 13.7, 10.9 Hz, 1H), 2.23 - 2.16 (m, 1H), 2.09 - 1.96 (m, 3H), 1.93 - 1.87 (m, 1H), 0.92 (d, *J* = 6.8 Hz, 3H).

^{13}C NMR (176 MHz, $CDCl_3$) δ 197.59, 192.07, 168.05, 164.19, 157.87, 154.53, 153.90, 125.57, 120.33, 120.00, 107.16, 105.62, 97.43, 91.56, 89.11, 80.11, 57.01, 56.42, 55.93, 45.31, 37.28, 35.04, 30.21, 28.11, 15.33.

HRMS (ESI) calcd for $C_{25}H_{25}ClO_7$ $[M + H]^+$ 473.13616 and 475.13321, found 473.13592 and 475.13301, respectively.

$[\alpha]_D^{20}$ +46.5 (*c* 0.16, $CHCl_3$).

Major diastereomer **3g-*S***:

^{1}H NMR (700 MHz, Chloroform-*d*) δ 7.28 (d, *J* = 2.7 Hz, 1H), 7.09 (dd, *J* = 9.0, 3.2 Hz, 1H), 6.93 (d, *J* = 9.0 Hz, 1H), 6.07 (s, 1H), 3.99 (s, 3H), 3.95 (s, 3H), 3.77 (s, 3H), 2.77 - 2.68 (m, 2H), 2.66 - 2.59 (m, 1H), 2.39 - 2.31 (m, 1H), 2.29 - 2.21 (m, 1H), 2.20 - 2.13 (m, 1H), 2.12 - 2.06 (m, 1H), 2.05 - 1.98 (m, 1H), 1.73 - 1.66 (m, 1H), 0.75 (d, *J* = 6.9 Hz, 3H).

^{13}C NMR (176 MHz, $CDCl_3$) δ 198.29, 192.10, 168.23, 164.08, 157.61, 153.89, 153.67, 125.26, 120.53, 119.64, 107.29, 105.70, 97.04, 91.82, 88.95, 79.08, 56.90, 56.28, 55.79, 48.06, 37.35, 33.25, 28.71, 28.09, 14.47.

HRMS (ESI) calcd for $C_{25}H_{25}ClO_7$ $[M + H]^+$ 473.13616 and 475.13321, found 473.13596 and 475.13300, respectively.

$[\alpha]_D^{20}$ +57.7 (*c* 0.18, $CHCl_3$).

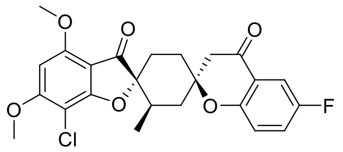

3h-*R*

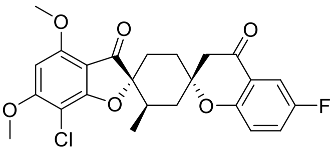

3h-*S*

Following the representative procedure for grisofulvin-chromanones 100 ºC for 1 h (10:1 dr), 7 mg (5%) of **3h-*R*** and 87 mg (61%) of **3h-*S*** were isolated.

Minor diastereomer **3h-*R***:

^{1}H NMR (600 MHz, Chloroform-*d*) δ 7.49 (dd, J = 8.2, 3.2 Hz, 1H), 7.21 (ddd, J = 9.1, 7.8, 3.2 Hz, 1H), 6.97 (dd, J = 9.1, 4.2 Hz, 1H), 6.08 (s, 1H), 4.00 (s, 3H), 3.96 (s, 3H), 2.95 (s, 2H), 2.52 – 2.45 (m, 1H), 2.38 (dd, J = 13.6, 11.0 Hz, 1H), 2.23 – 2.15 (m, 1H), 2.09 – 1.87 (m, 4H), 0.91 (d, J = 6.9 Hz, 3H).

^{13}C NMR (151 MHz, $CDCl_3$) δ 197.55, 191.28 (d, J = 2.0 Hz), 168.05, 164.25, 157.89, 157.12 (d, J = 241.5 Hz), 156.09 (d, J = 1.3 Hz), 123.94 (d, J = 24.3 Hz), 120.89 (d, J = 6.3 Hz), 120.31 (d, J = 7.2 Hz), 111.63 (d, J = 23.2 Hz), 105.57, 97.44, 91.37, 89.13, 80.56, 57.03, 56.43, 45.07, 37.26, 35.00, 30.15, 28.11, 15.30.

^{19}F NMR (565 MHz, Chloroform-*d*) δ -122.17 (td, J = 8.1, 4.3 Hz).

HRMS (ESI) calcd for $C_{24}H_{22}ClO_6F$ $[M + H]^+$ 461.11617 and 463.11322, found 461.11578 and 463.11287, respectively.

$[\alpha]^{20}_{D}$ +32.1 (*c* 0.24, $CHCl_3$).

Major diastereomer **3h-*S***:

^{1}H NMR (600 MHz, Chloroform-*d*) δ 7.49 (dd, J = 8.2, 3.2 Hz, 1H), 7.20 (ddd, J = 9.0, 7.7, 3.2 Hz, 1H), 6.97 (dd, J = 9.1, 4.1 Hz, 1H), 6.07 (s, 1H), 3.98 (s, 3H), 3.94 (s, 3H), 2.74 (d, J = 3.4 Hz, 2H), 2.65 – 2.58 (m, 1H), 2.39 – 2.23 (m, 2H), 2.18 (dd, J = 14.3, 12.9 Hz, 1H), 2.11 – 2.04 (m, 1H), 2.04 – 1.97 (m, 1H), 1.75 – 1.68 (m, 1H), 0.75 (d, J = 6.8 Hz, 3H).

^{13}C NMR (151 MHz, $CDCl_3$) δ 198.41, 191.48, 168.40, 164.31, 157.81, 157.25 (d, J = 241.6 Hz), 155.45 (d, J = 2.0 Hz), 123.90 (d, J = 24.3 Hz), 121.28 (d, J = 6.2 Hz), 120.13 (d, J = 7.2 Hz), 111.90 (d, J = 23.5 Hz), 105.84, 97.23, 91.81, 89.12, 79.72, 57.07, 56.45, 48.02, 37.47, 33.41, 28.92, 28.23, 14.61.

^{19}F NMR (565 MHz, Chloroform-*d*) δ -121.85 (td, J = 8.1, 4.2 Hz).

HRMS (ESI) calcd for $C_{24}H_{22}ClO_6F$ $[M + H]^+$ 461.11617 and 463.11322, found 461.11581 and 463.11288, respectively.

$[\alpha]^{20}_{D}$ +29.7 (*c* 0.26, $CHCl_3$).

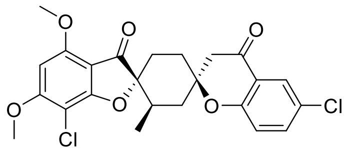

3i-*R*

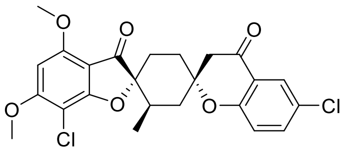

3i-*S*

Following the representative procedure for grisofulvin-chromanones at 130 °C for 15 min (5.8:1 dr), 9 mg (6%) of **3i-*R*** and 95 mg (65%) of **3i-*S*** were isolated.

Minor diastereomer **3i-*R***:

^{1}H NMR (500 MHz, Chloroform-*d*) δ 7.80 (d, J = 2.7 Hz, 1H), 7.42 (dd, J = 8.8, 2.7 Hz, 1H), 6.96 (d, J = 8.8 Hz, 1H), 6.08 (s, 1H), 4.00 (s, 3H), 3.97 (s, 3H), 2.96 (s, 2H), 2.54 - 2.46 (m, 1H), 2.38 (dd, J = 13.6, 11.0 Hz, 1H), 2.24 - 2.15 (m, 1H), 2.09 - 1.86 (m, 4H), 0.91 (d, J = 6.8 Hz, 3H).

^{13}C NMR (126 MHz, $CDCl_3$) δ 197.55, 190.91, 168.03, 164.25, 158.34, 157.87, 136.28, 126.55, 125.97, 121.31, 120.43, 105.53, 97.39, 91.29, 89.10, 80.78, 57.04, 56.44, 45.01, 37.23, 34.97, 30.14, 28.08, 15.29.

HRMS (ESI) calcd for $C_{24}H_{22}Cl_2O_6$ $[M + H]^+$ 477.08662 and 479.08367, found 477.08631 and 479.08330, respectively.

$[\alpha]_D^{20}$ +29.0 (*c* 0.12, $CHCl_3$).

Major diastereomer **3i-*S***:

^{1}H NMR (600 MHz, Chloroform-*d*) δ 7.79 (d, J = 2.7 Hz, 1H), 7.41 (dd, J = 8.8, 2.7 Hz, 1H), 6.95 (d, J = 8.8 Hz, 1H), 6.06 (s, 1H), 3.98 (s, 3H), 3.94 (s, 3H), 2.78 - 2.69 (m, 2H), 2.64 - 2.57 (m, 1H), 2.36 - 2.23 (m, 2H), 2.18 (dd, J = 14.3, 12.9 Hz, 1H), 2.06 (dq, J = 14.0, 3.2 Hz, 1H), 2.01 - 1.97 (m, 1H), 1.75 - 1.69 (m, 1H), 0.75 (d, J = 6.8 Hz, 3H).

^{13}C NMR (151 MHz, $CDCl_3$) δ 198.23, 190.94, 168.27, 164.19, 157.68, 157.60, 136.13, 126.64, 126.07, 121.55, 120.14, 105.69, 97.10, 91.59, 89.02, 79.83, 56.96, 56.33, 47.87, 37.35, 33.28, 28.89, 28.08, 14.48.

HRMS (ESI) calcd for $C_{24}H_{22}Cl_2O_6$ $[M + H]^+$ 477.08662 and 479.08367, found 477.08644 and 479.08344, respectively.

$[\alpha]_D^{20}$ +27.3 (*c* 0.16, $CHCl_3$).

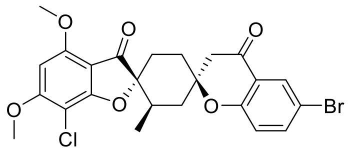

3j-*R*

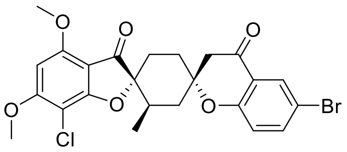

3j-*S*

Following the representative procedure for grisofulvin-chromanones 100 ºC for 1 h (10:1 dr), 8 mg (5%) of **3j-*R*** and 91 mg (57%) of **3j-S** were isolated.

Minor diastereomer **3j-*R***:

^{1}H NMR (600 MHz, Chloroform-*d*) δ 7.95 (d, *J* = 2.5 Hz, 1H), 7.55 (dd, *J* = 8.8, 2.5 Hz, 1H), 6.91 (d, *J* = 8.8 Hz, 1H), 6.08 (s, 1H), 4.00 (s, 3H), 3.96 (s, 3H), 2.95 (s, 2H), 2.53 – 2.46 (m, 1H), 2.38 (dd, *J* = 13.6, 11.0 Hz, 1H), 2.19 (ddd, *J* = 11.1, 7.0, 4.4 Hz, 1H), 2.08 – 1.88 (m, 4H), 0.91 (d, *J* = 6.9 Hz, 3H).

^{13}C NMR (151 MHz, $CDCl_3$) δ 197.52, 190.76, 168.04, 164.27, 158.81, 157.89, 139.05, 129.09, 121.84, 120.80, 113.68, 105.55, 97.43, 91.28, 89.14, 80.80, 57.03, 56.43, 45.01, 37.25, 34.98, 30.16, 28.09, 15.29.

HRMS (ESI) calcd for $C_{24}H_{22}ClO_6Br$ $[M + H]^+$ 523.03316 and 525.03111, found 523.03329 and 525.03059, respectively.

$[\alpha]_D^{20}$ +27.7 (*c* 0.22, $CHCl_3$).

Major diastereomer **3j-*S***:

^{1}H NMR (500 MHz, Chloroform-*d*) δ 7.95 (d, *J* = 2.6 Hz, 1H), 7.55 (dd, *J* = 8.8, 2.6 Hz, 1H), 6.90 (d, *J* = 8.8 Hz, 1H), 6.07 (s, 1H), 3.99 (s, 3H), 3.94 (s, 3H), 2.74 (d, *J* = 1.7 Hz, 2H), 2.66 – 2.56 (m, 1H), 2.38 – 2.24 (m, 2H), 2.19 (dd, *J* = 14.3, 12.9 Hz, 1H), 2.10 – 1.96 (m, 2H), 1.74 – 1.70 (m, 1H), 0.75 (d, *J* = 6.9 Hz, 3H).

^{13}C NMR (126 MHz, $CDCl_3$) δ 198.28, 190.90, 168.26, 164.20, 158.07, 157.69, 138.95, 129.20, 122.03, 120.53, 113.79, 105.68, 97.08, 91.61, 88.99, 79.85, 56.99, 56.35, 47.83, 37.34, 33.28, 28.89, 28.08, 14.49.

HRMS (ESI) calcd for $C_{24}H_{22}ClO_6Br$ $[M + H]^+$ 521.03611, 523.03316, and 525.03111, found 521.03561, 523.0326 and 525.03055, respectively.

$[\alpha]_D^{20}$ +25.8 (*c* 0.16, $CHCl_3$).

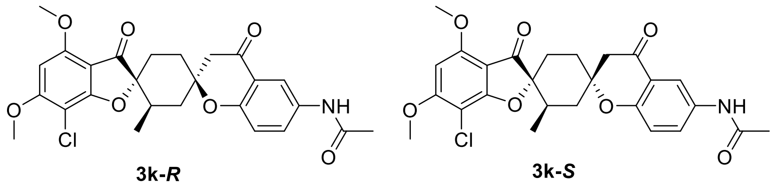

Following the representative procedure for grisofulvin-chromanones at 130 °C for 15 min (7:1 dr), 1.5 mg (1%) of **3k-*R*** and 20 mg (17%) of **3k-*S*** were isolated. The difficult separation is what led to the low isolated yields.

Minor diastereomer **3k-*R***:

^{1}H NMR (600 MHz, Chloroform-*d*) δ 7.86 (d, *J* = 7.9 Hz, 1H), 7.67 (s, 1H), 7.11 (s, 1H), 6.99 (d, *J* = 8.7 Hz, 1H), 6.08 (s, 1H), 4.00 (s, 3H), 3.96 (s, 3H), 2.95 (s, 2H), 2.53 - 2.45 (m, 1H), 2.38 (dd, *J* = 13.6, 10.9 Hz, 1H), 2.24 - 2.14 (m, 4H), 2.07 - 1.96 (m, 3H), 1.95 - 1.86 (m, 1H), 0.91 (d, *J* = 6.8 Hz, 3H).

^{13}C NMR (151 MHz, $CDCl_3$) δ 197.65, 191.67, 168.52, 168.14, 164.31, 157.98, 156.89, 131.49, 130.00, 120.44, 119.48, 117.69, 105.70, 97.52, 91.54, 89.22, 80.53, 57.14, 56.55, 45.37, 37.43, 35.12, 30.32, 24.64, 15.41.

HRMS (ESI) calcd for $C_{26}H_{26}ClO_7N$ $[M + H]^+$ 500.14706 and 502.14411, found 500.14672 and 502.14374, respectively.

$[\alpha]_D^{20}$ +30.0 (*c* 0.11, $CHCl_3$).

Major diastereomer **3k-*S***:

^{1}H NMR (600 MHz, Chloroform-*d*) δ 8.00 (dd, *J* = 9.0, 2.7 Hz, 1H), 7.77 - 7.70 (m, 2H), 7.02 (d, *J* = 8.9 Hz, 1H), 6.09 (s, 1H), 4.02 (s, 3H), 3.97 (s, 3H), 2.77 (d, *J* = 2.8 Hz, 2H), 2.70 - 2.62 (m, 1H), 2.39 (td, *J* = 13.4, 4.0 Hz, 1H), 2.29 (td, *J* = 14.1, 3.9 Hz, 1H), 2.24 - 2.17 (m, 4H), 2.11 (dd, *J* = 14.0, 3.3 Hz, 1H), 2.08 - 2.01 (m, 1H), 1.76 - 1.73 (m, 1H), 0.78 (d, *J* = 6.8 Hz, 3H).

^{13}C NMR (151 MHz, $CDCl_3$) δ 198.48, 192.10, 168.38, 164.24, 157.72, 156.00, 131.90, 129.85, 120.52, 119.12, 117.58, 105.80, 97.22, 91.85, 89.02, 79.49, 57.01, 56.37, 48.21, 37.45, 33.34, 28.99, 28.17, 24.41, 14.56.

HRMS (ESI) calcd for $C_{26}H_{26}ClO_7N$ $[M + H]^+$ 500.14706 and 502.14411, found 500.14677 and 502.14384, respectively.

$[\alpha]_D^{20}$ +44.0 (*c* 0.15, $CHCl_3$).

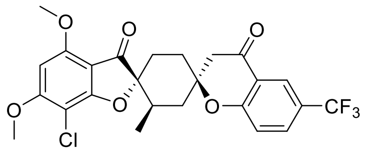

3l-*R*

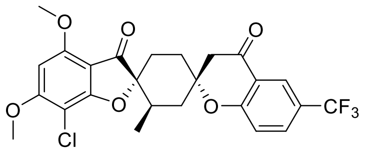

3l-*S*

Following the representative procedure for grisofulvin-chromanones at 130 °C for 15 min (6.5:1 dr), 8 mg (5%) of **3l-*R*** and 57 mg (36%) of **3l-*S*** were isolated.

Minor diastereomer **3l-*R***:

^{1}H NMR (600 MHz, Chloroform-*d*) δ 8.14 (d, *J* = 2.4 Hz, 1H), 7.71 (dd, *J* = 8.8, 2.4 Hz, 1H), 7.11 (d, *J* = 8.7 Hz, 1H), 6.08 (s, 1H), 4.00 (s, 3H), 3.97 (s, 3H), 3.01 (s, 2H), 2.57 - 2.51 (m, 1H), 2.43 (dd, *J* = 13.6, 11.1 Hz, 1H), 2.26 - 2.19 (m, 1H), 2.09 - 1.90 (m, 4H), 0.92 (d, *J* = 6.8 Hz, 3H).

^{13}C NMR (151 MHz, $CDCl_3$) δ 197.58, 190.85, 168.16, 164.41, 162.12, 158.01, 132.85 (q, *J* = 3.3 Hz), 124.64 (q, *J* = 4.0 Hz), 124.07 (q, *J* = 271.7 Hz), 123.71 (q, *J* = 33.5 Hz), 120.25, 119.68, 105.64, 97.55, 91.23, 89.27, 81.49, 57.14, 56.55, 45.09, 37.40, 35.08, 30.28, 28.23, 15.38.

^{19}F NMR (470 MHz, $CDCl_3$) δ -62.10.

HRMS (ESI) calcd for $C_{25}H_{22}ClO_6F_3$ $[M + H]^+$ 511.11298 and 513.11003, found 511.11258 and 513.10958, respectively.

$[\alpha]_D^{20}$ +40.7 (*c* 0.12, $CHCl_3$).

Major diastereomer **3l-*S***:

^{1}H NMR (600 MHz, Chloroform-*d*) δ 8.15 (d, *J* = 2.4 Hz, 1H), 7.71 (dd, *J* = 8.8, 2.4 Hz, 1H), 7.12 (d, *J* = 8.7 Hz, 1H), 6.08 (s, 1H), 3.99 (s, 3H), 3.95 (s, 3H), 2.84 - 2.76 (m, 2H), 2.68 - 2.59 (m, 1H), 2.40 - 2.29 (m, 2H), 2.24 (dd, *J* = 14.4, 13.0 Hz, 1H), 2.12 - 2.06 (m, 1H), 2.06 - 1.99 (m, 1H), 1.78 - 1.71 (m, 1H), 0.77 (d, *J* = 6.8 Hz, 3H).

^{13}C NMR (151 MHz, $CDCl_3$) δ 198.25, 190.85, 168.33, 164.29, 161.32, 157.76, 132.69 (q, *J* = 3.3 Hz), 124.67 (q, *J* = 3.9 Hz), 123.92 (q, *J* = 271.6 Hz), 123.73 (q, *J* = 33.5 Hz), 120.42, 119.36, 105.73, 97.19, 91.51, 89.08, 80.49, 57.00, 56.38, 47.92, 37.49, 33.36, 29.04, 28.14, 14.50.

^{19}F NMR (470 MHz, $CDCl_3$) δ -62.08.

HRMS (ESI) calcd for $C_{25}H_{22}ClO_6F_3$ $[M + H]^+$ 511.11298 and 513.11003, found 511.11262 and 513.10966, respectively.

$[\alpha]_D^{20}$ +26.9 (*c* 0.12, $CHCl_3$).

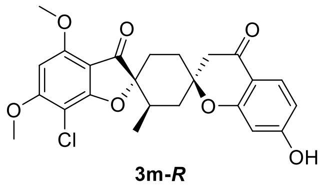

3m-*R*

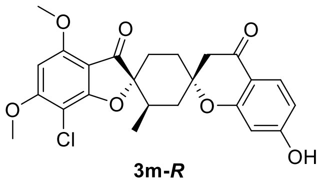

3m-*R*

Following the representative procedure for grisofulvin-chromanones at 130 °C for 15 min (6.5:1 dr), 8 mg (6%) of **3m-*R*** and 89 mg (63%) of **3m-*S*** were isolated.

Minor diastereomer **3m-*R***:

^{1}H NMR (500 MHz, Chloroform-*d*) δ 7.79 (d, *J* = 8.5 Hz, 2H), 6.63 (d, *J* = 2.2 Hz, 1H), 6.55 (dd, *J* = 8.6, 2.2 Hz, 1H), 6.07 (s, 1H), 4.01 (s, 3H), 3.97 (s, 3H), 2.91 (s, 2H), 2.55 - 2.45 (m, 1H), 2.39 (dd, *J* = 13.6, 10.6 Hz, 1H), 2.27 - 2.18 (m, 1H), 2.13 - 2.01 (m, 3H), 1.98 - 1.89 (m, 1H), 0.94 (d, *J* = 6.8 Hz, 3H).

^{13}C NMR (126 MHz, $CDCl_3$) δ 198.83, 190.75, 168.19, 164.74, 164.37, 162.02, 158.11, 128.88, 114.15, 110.57, 105.37, 103.85, 97.29, 92.03, 88.97, 80.42, 57.10, 56.22, 45.10, 37.55, 35.04, 30.37, 29.25, 27.97, 15.37.

HRMS (ESI) calcd for $C_{24}H_{23}ClO_7$ $[M + H]^+$ 459.12051 and 461.11756, found 459.12042 and 461.11746, respectively.

$[\alpha]_D^{20}$ +30.5 (*c* 0.18, $CHCl_3$).

Major diastereomer **3m-*S***:

^{1}H NMR This spectra was run in a mixture of 3 MeOD: 1 $CDCl_3$ and locked to MeOD (600 MHz, MeOD) δ 7.68 (d, *J* = 8.7 Hz, 1H), 6.46 (dd, *J* = 8.7, 2.3 Hz, 1H), 6.40 (d, *J* = 2.3 Hz, 1H), 6.33 - 6.29 (m, 1H), 4.01 (s, 3H), 3.94 (s, 3H), 2.68 (d, *J* = 4.1 Hz, 2H), 2.62 - 2.54 (m, 1H), 2.32 (td, *J* = 13.5, 4.2 Hz, 1H), 2.21 (td, *J* = 14.1, 4.0 Hz, 1H), 2.15 - 2.08 (m, 2H), 2.06 - 2.02 (m, 1H), 1.76 - 1.70 (m, 1H), 0.73 (d, *J* = 6.8 Hz, 3H).

^{13}C NMR This spectra was run in a mixture of 3 MeOD: 1 $CDCl_3$ and locked to MeOD (151 MHz, MeOD) δ 199.96, 192.72, 169.34, 166.84, 166.03, 162.70, 159.16, 129.41, 114.57, 111.39, 106.34, 104.20, 97.65, 92.61, 92.60, 90.58, 80.27, 57.59, 56.74, 38.48, 34.43, 30.06, 29.03, 14.84.

HRMS (ESI) calcd for $C_{24}H_{23}ClO_7$ $[M + H]^+$ 459.12051 and 461.11756, found 459.12041 and 461.11736, respectively.

$[\alpha]_D^{20}$ +26.0 (*c* 0.27, $CHCl_3$).

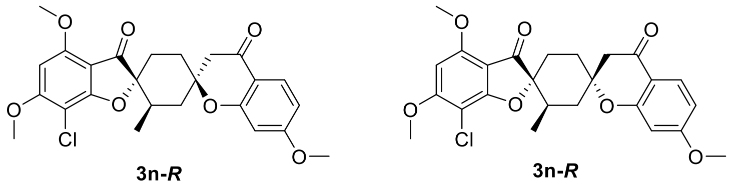

Following the representative procedure for grisofulvin-chromanones 100 ºC for 1 h (10:1 dr), 6 mg (4%) of **3n-*R*** and 90 mg (62%) of **3n-*S*** were isolated.

Minor diastereomer **3n-*R***:

^{1}H NMR (500 MHz, Chloroform-*d*) δ 7.78 (d, *J* = 8.7 Hz, 1H), 6.54 (dd, *J* = 8.7, 2.4 Hz, 1H), 6.44 (d, *J* = 2.4 Hz, 1H), 6.08 (s, 1H), 4.00 (s, 3H), 3.97 (s, 3H), 3.84 (s, 3H), 2.91 (s, 2H), 2.53 – 2.45 (m, 1H), 2.39 (dd, *J* = 13.5, 11.1 Hz, 1H), 2.27 – 2.18 (m, 1H), 2.08 – 1.86 (m, 4H), 0.91 (d, *J* = 6.8 Hz, 3H).

^{13}C NMR (126 MHz, $CDCl_3$) δ 197.60, 190.41, 167.96, 166.39, 164.08, 161.88, 157.72, 128.21, 114.28, 109.69, 105.47, 101.23, 97.28, 91.40, 88.93, 80.58, 56.90, 56.30, 55.68, 44.81, 37.36, 34.91, 30.18, 28.13, 15.22.

HRMS (ESI) calcd for $C_{25}H_{25}ClO_7$ $[M + H]^+$ 473.13616 and 475.13321, found 473.13571 and 475.13275, respectively.

$[\alpha]_D^{20}$ +31.1 (*c* 0.12, $CHCl_3$).

Major diastereomer **3n-*S***:

^{1}H NMR (500 MHz, Chloroform-*d*) δ 7.75 (d, *J* = 8.8 Hz, 1H), 6.51 (dd, *J* = 8.7, 2.4 Hz, 1H), 6.42 (d, *J* = 2.4 Hz, 1H), 6.06 (s, 1H), 3.97 (s, 3H), 3.93 (s, 3H), 3.82 (s, 3H), 2.67 (d, *J* = 2.1 Hz, 2H), 2.64 – 2.58 (m, 1H), 2.40 – 2.30 (m, 1H), 2.24 (td, *J* = 14.0, 3.9 Hz, 1H), 2.15 (dd, *J* = 14.3, 12.8 Hz, 1H), 2.10 – 2.03 (m, 1H), 2.03 – 1.97 (m, 1H), 1.70 (ddd, *J* = 12.8, 3.9, 2.6 Hz, 1H), 0.74 (d, *J* = 6.9 Hz, 3H).

^{13}C NMR (126 MHz, $CDCl_3$) δ 198.24, 190.63, 168.18, 166.35, 164.08, 161.19, 157.63, 128.30, 114.48, 109.78, 105.66, 101.13, 96.94, 91.84, 88.93, 79.64, 56.93, 56.29, 55.68, 47.81, 37.52, 33.29, 29.04, 28.14, 14.47.

HRMS (ESI) calcd for $C_{25}H_{25}ClO_7$ $[M + H]^+$ 473.13616 and 475.13321, found 473.13574 and 475.13277, respectively.

$[\alpha]_D^{20}$ +28.4 (*c* 0.2, $CHCl_3$).

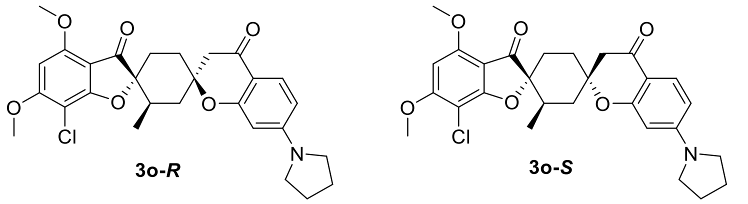

Following the representative procedure for grisofulvin-chromanones employing 4-fluoro-2-hydroxyacetophenone at 110 ºC for 2 h (10:1 dr), 6 mg (4%) of **3o-*R*** and 80 mg (51%) of **3o-*S*** were isolated as the result of an S_NAr reaction.

Minor diastereomer **3o-*R***:

^{1}H NMR (600 MHz, Chloroform-*d*) δ 7.72 (d, *J* = 8.8 Hz, 1H), 6.21 (dd, *J* = 8.8, 2.3 Hz, 1H), 6.07 (s, 1H), 5.99 (d, *J* = 2.3 Hz, 1H), 3.99 (s, 3H), 3.96 (s, 3H), 3.38 – 3.33 (m, 4H), 2.86 (d, *J* = 1.7 Hz, 2H), 2.53 – 2.44 (m, 1H), 2.37 (dd, *J* = 13.5, 11.3 Hz, 1H), 2.26 – 2.17 (m, 1H), 2.06 – 1.98 (m, 7H), 1.97 – 1.89 (m, 1H), 0.90 (d, *J* = 6.9 Hz, 3H).

^{13}C NMR (151 MHz, $CDCl_3$) δ 197.85, 189.58, 168.14, 164.15, 161.63, 157.81, 153.78, 128.40, 110.08, 106.60, 105.69, 98.17, 97.44, 91.80, 89.03, 79.95, 57.00, 56.41, 47.84, 44.87, 37.70, 35.12, 30.52, 28.44, 25.55, 15.37.

HRMS (ESI) calcd for $C_{28}H_{30}ClO_6N$ $[M + H]^+$ 512.18344 and 514.18049, found 512.18278 and 514.17997, respectively.

$[\alpha]_D^{20}$ +18.4 (*c* 0.13, $CHCl_3$).

Major diastereomer **3o-*S***:

^{1}H NMR (500 MHz, Chloroform-*d*) δ 7.72 (d, *J* = 8.8 Hz, 1H), 6.21 (dd, *J* = 8.8, 2.3 Hz, 1H), 6.07 (s, 1H), 5.98 (d, *J* = 2.3 Hz, 1H), 3.99 (s, 3H), 3.94 (s, 3H), 3.40 – 3.27 (m, 4H), 2.72 – 2.59 (m, 3H), 2.45 – 2.35 (m, 1H), 2.24 (td, *J* = 14.1, 4.0 Hz, 1H), 2.16 (dd, *J* = 14.2, 12.8 Hz, 1H), 2.12 – 2.06 (m, 1H), 2.06 – 1.99 (m, 5H), 1.70 (ddd, *J* = 13.0, 4.0, 2.7 Hz, 1H), 0.75 (d, *J* = 6.9 Hz, 3H).

^{13}C NMR (126 MHz, $CDCl_3$) δ 198.39, 189.93, 168.25, 164.06, 160.97, 157.69, 153.70, 128.44, 110.11, 106.63, 105.82, 98.03, 96.97, 92.29, 88.94, 78.91, 56.98, 56.34, 47.84, 47.79, 37.85, 33.43, 29.26, 28.34, 25.50, 14.57.

HRMS (ESI) calcd for $C_{28}H_{30}ClO_6N$ $[M + H]^+$ 512.18344 and 514.18049, found 512.18287 and 514.18004, respectively.

$[\alpha]_D^{20}$ +21.8 (*c* 0.49, $CHCl_3$).

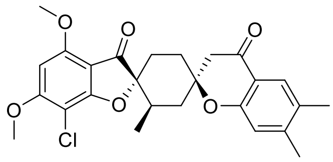

3p-*R*

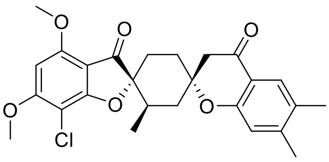

3p-*S*

Following the representative procedure for grisofulvin-chromanones 100 ºC for 1 h (10:1 dr), 10 mg (7%) of **3p-*R*** and 57 mg (39%) of **3p-*S*** were isolated.

Minor diastereomer **3p-*R***:

^{1}H NMR (500 MHz, Chloroform-*d*) δ 7.57 (s, 1H), 6.79 (s, 1H), 6.07 (s, 1H), 3.99 (s, 3H), 3.96 (s, 3H), 2.90 (s, 2H), 2.51 – 2.42 (m, 1H), 2.35 (dd, J = 13.6, 10.8 Hz, 1H), 2.26 (s, 3H), 2.22 – 2.17 (m, 4H), 2.06 – 1.95 (m, 3H), 1.94 – 1.87 (m, 1H), 0.91 (d, J = 6.8 Hz, 3H).

^{13}C NMR (126 MHz, $CDCl_3$) δ 197.67, 191.94, 168.03, 164.15, 158.20, 157.82, 146.98, 129.65, 126.53, 119.18, 118.33, 105.57, 97.36, 91.60, 89.02, 80.00, 57.00, 56.97, 56.40, 45.31, 37.36, 35.00, 30.24, 28.10, 20.66, 18.89, 15.33.

HRMS (ESI) calcd for $C_{26}H_{27}ClO_6$ $[M + H]^+$ 471.15689 and 473.15394, found 471.15647 and 473.15356, respectively.

$[\alpha]_D^{20}$ 34.8 (*c* 0.11, $CHCl_3$).

Major diastereomer **3p-*S***:

^{1}H NMR (500 MHz, Chloroform-*d*) δ 7.59 (s, 1H), 6.80 (s, 1H), 6.07 (s, 1H), 3.99 (s, 3H), 3.95 (s, 3H), 2.75 – 2.60 (m, 3H), 2.37 (td, J = 13.4, 4.1 Hz, 1H), 2.29 – 2.23 (m, 4H), 2.21 – 2.13 (m, 4H), 2.11 – 2.05 (m, 1H), 2.02 (ddd, J = 14.3, 4.3, 2.7 Hz, 1H), 1.73 – 1.69 (m, 1H), 0.75 (d, J = 6.8 Hz, 3H).

^{13}C NMR (126 MHz, $CDCl_3$) δ 198.46, 192.17, 168.33, 164.13, 157.69, 157.51, 146.85, 129.80, 126.70, 119.06, 118.59, 105.80, 97.10, 92.02, 88.93, 79.06, 56.99, 56.35, 48.23, 37.54, 33.32, 28.93, 28.20, 20.60, 18.88, 14.57.

HRMS (ESI) calcd for $C_{26}H_{27}ClO_6$ $[M + H]^+$ 471.15689 and 473.15394, found 471.15652 and 473.15357, respectively.

$[\alpha]_D^{20}$ +34.2 (*c* 0.11, $CHCl_3$).

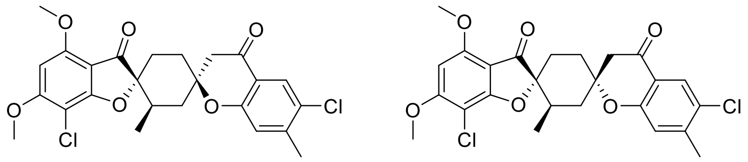

3q-*R*   **3q-*S***

Following the representative procedure for grisofulvin-chromanones at 130 °C for 15 min (5.8:1 dr), 7 mg (5%) of **3q-*R*** and 101 mg (67%) of **3q-*S*** were isolated.

Minor diastereomer **3q-*R***:

^{1}H NMR (500 MHz, Chloroform-*d*) δ 7.78 (s, 1H), 6.90 (s, 1H), 6.08 (s, 1H), 4.00 (s, 3H), 3.96 (s, 3H), 2.92 (s, 2H), 2.52 - 2.42 (m, 1H), 2.41 - 2.28 (m, 4H), 2.23 - 2.15 (m, 1H), 2.08 - 1.86 (m, 4H), 0.91 (d, *J* = 6.9 Hz, 3H).

^{13}C NMR (126 MHz, $CDCl_3$) δ 197.58, 190.74, 168.02, 164.22, 158.18, 157.85, 145.48, 127.25, 126.31, 120.76, 119.54, 105.53, 97.38, 91.36, 89.07, 80.63, 57.02, 56.42, 45.02, 37.32, 34.97, 30.16, 28.08, 21.00, 15.31.

HRMS (ESI) calcd for $C_{25}H_{24}Cl_2O_6$ $[M + H]^+$ 491.10227 and 493.09932, found 491.10197 and 493.09895, respectively.

$[\alpha]_D^{20}$ +33.3 (*c* 0.12, $CHCl_3$).

Major diastereomer **3q-*S***:

^{1}H NMR (600 MHz, Chloroform-*d*) δ 7.78 (s, 1H), 6.89 (s, 1H), 6.06 (s, 1H), 3.98 (s, 3H), 3.94 (s, 3H), 2.70 (d, *J* = 1.5 Hz, 2H), 2.65 - 2.55 (m, 1H), 2.37 - 2.30 (m, 4H), 2.26 (td, *J* = 14.0, 3.7 Hz, 1H), 2.17 (dd, *J* = 14.3, 12.9 Hz, 1H), 2.08 - 2.02 (m, 1H), 2.02 - 1.95 (m, 1H), 1.73 - 1.69 (m, 1H), 0.74 (d, *J* = 6.8 Hz, 3H).

^{13}C NMR (151 MHz, $CDCl_3$) δ 198.26, 190.81, 168.27, 164.17, 157.68, 157.44, 145.30, 127.33, 126.41, 120.57, 119.80, 105.71, 97.10, 91.66, 89.00, 79.69, 56.96, 56.33, 47.89, 37.37, 33.29, 28.97, 28.10, 20.87, 14.49.

HRMS (ESI) calcd for $C_{25}H_{24}Cl_2O_6$ $[M + H]^+$ 491.10227 and 493.09932, found 491.10204 and 493.09932, respectively.

$[\alpha]_D^{20}$ +22.4 (*c* 0.19, $CHCl_3$).

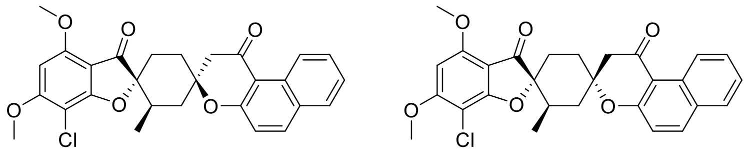

3r-*R* **3r-*S***

Following the representative procedure for grisofulvin-chromanones 100 ºC for 1 h (8:1 dr), 8 mg (5%) of **3r-*R*** and 84 mg (55%) of **3r-*S*** were isolated.

Minor diastereomer **3r-*R***:

^{1}H NMR (500 MHz, Chloroform-*d*) δ 9.41 (d, *J* = 8.5 Hz, 1H), 7.93 (d, *J* = 9.0 Hz, 1H), 7.74 (d, *J* = 8.3 Hz, 1H), 7.62 (ddd, *J* = 8.5, 6.9, 1.5 Hz, 1H), 7.41 (ddd, *J* = 8.0, 6.9, 1.2 Hz, 1H), 7.13 (d, *J* = 9.0 Hz, 1H), 6.08 (s, 1H), 4.00 (s, 3H), 3.97 (s, 3H), 3.08 (s, 2H), 2.55 (td, *J* = 13.1, 4.3 Hz, 1H), 2.46 (dd, *J* = 13.3, 11.3 Hz, 1H), 2.32 – 2.23 (m, 1H), 2.17 – 2.04 (m, 3H), 1.98 (td, *J* = 14.0, 13.0, 4.1 Hz, 1H), 0.92 (d, *J* = 6.8 Hz, 3H).

^{13}C NMR (126 MHz, $CDCl_3$) δ 197.70, 193.10, 168.09, 164.21, 162.06, 157.85, 137.70, 131.37, 129.70, 129.03, 128.48, 125.60, 124.75, 119.62, 111.79, 105.60, 97.39, 91.51, 89.07, 80.55, 57.02, 56.42, 46.52, 37.05, 35.02, 29.88, 28.26, 15.33.

HRMS (ESI) calcd for $C_{28}H_{25}ClO_6$ $[M + H]^+$ 493.14124 and 495.13829, found 493.14068 and 495.13780, respectively.

$[\alpha]_D^{20}$ +48.7 (*c* 0.11, $CHCl_3$).

Major diastereomer **3r-*S***:

^{1}H NMR (500 MHz, Chloroform-*d*) δ 9.43 (d, *J* = 8.5 Hz, 1H), 7.93 (d, *J* = 8.9 Hz, 1H), 7.73 (d, *J* = 9.1 Hz, 1H), 7.61 (ddd, *J* = 8.5, 6.9, 1.4 Hz, 1H), 7.40 (ddd, *J* = 8.0, 6.9, 1.2 Hz, 1H), 7.13 (d, *J* = 9.0 Hz, 1H), 6.07 (s, 1H), 3.98 (s, 3H), 3.95 (s, 3H), 2.87 (s, 2H), 2.73 – 2.62 (m, 1H), 2.46 – 2.29 (m, 2H), 2.29 – 2.11 (m, 3H), 1.80 – 1.71 (m, 1H), 0.77 (d, *J* = 6.8 Hz, 3H).

^{13}C NMR (126 MHz, $CDCl_3$) δ 198.31, 193.26, 168.27, 164.14, 161.27, 157.68, 137.66, 131.39, 129.67, 129.06, 128.41, 125.67, 124.74, 119.34, 112.02, 105.74, 97.06, 91.85, 88.95, 79.50, 56.96, 56.34, 49.37, 37.08, 33.40, 28.75, 28.20, 14.53.

HRMS (ESI) calcd for $C_{28}H_{25}ClO_6$ $[M + H]^+$ 493.14124 and 495.13829, found 493.14068 and 495.13776, respectively.

$[\alpha]_D^{20}$ +28.9 (*c* 0.1, $CHCl_3$).

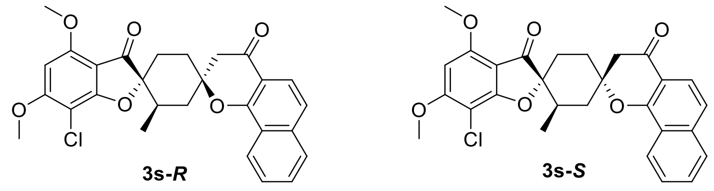

Following the representative procedure for grisofulvin-chromanones 100 ºC for 1 h (10:1 dr), 11 mg (7%) of **3s-*R*** and 85 mg (56%) of **3s-*S*** were isolated.

Minor diastereomer **3s-*R***:

^{1}H NMR (600 MHz, Chloroform-*d*) δ 8.38 (d, *J* = 8.3 Hz, 1H), 7.83 (d, *J* = 8.6 Hz, 1H), 7.78 (d, *J* = 8.1 Hz, 1H), 7.62 (ddd, *J* = 8.1, 6.9, 1.3 Hz, 1H), 7.55 (ddd, *J* = 8.2, 6.9, 1.2 Hz, 1H), 7.38 (d, *J* = 8.6 Hz, 1H), 6.08 (s, 1H), 4.00 (s, 3H), 3.97 (s, 3H), 3.08 (d, *J* = 2.4 Hz, 2H), 2.69 (td, *J* = 13.2, 4.6 Hz, 1H), 2.58 (dd, *J* = 13.6, 11.6 Hz, 1H), 2.34 – 2.26 (m, 1H), 2.20 – 2.11 (m, 2H), 2.10 – 1.97 (m, 2H), 0.92 (d, *J* = 6.8 Hz, 3H).

^{13}C NMR (151 MHz, $CDCl_3$) δ 197.61, 191.54, 168.13, 164.26, 158.23, 157.89, 137.91, 129.75, 127.91, 126.36, 125.43, 124.05, 121.49, 120.69, 114.80, 105.66, 97.43, 91.47, 89.13, 81.52, 57.03, 56.40, 44.73, 37.58, 35.04, 30.08, 28.54, 15.38.

HRMS (ESI) calcd for $C_{28}H_{25}ClO_6$ $[M + H]^+$ 493.14124 and 495.13829, found 493.14067 and 495.13829, respectively.

$[\alpha]_D^{20}$ +16.6 (*c* 0.16, $CHCl_3$).

Major diastereomer **3s-*S***:

^{1}H NMR (500 MHz, Chloroform-*d*) δ 8.40 (d, *J* = 8.8 Hz, 1H), 7.84 (d, *J* = 8.6 Hz, 1H), 7.79 (d, *J* = 7.5 Hz, 1H), 7.66 – 7.56 (m, 2H), 7.38 (d, *J* = 8.6 Hz, 1H), 6.08 (s, 1H), 3.99 (s, 3H), 3.96 (s, 3H), 2.87 (d, *J* = 3.9 Hz, 2H), 2.80 – 2.70 (m, 1H), 2.52 – 2.17 (m, 5H), 1.82 – 1.76 (m, 1H), 0.79 (d, *J* = 6.9 Hz, 3H).

^{13}C NMR (126 MHz, $CDCl_3$) δ 198.31, 191.70, 168.34, 164.19, 157.71, 157.23, 137.84, 129.67, 128.04, 126.55, 125.44, 123.53, 121.58, 120.72, 114.97, 105.82, 97.12, 91.84, 88.97, 80.60, 56.99, 56.36, 47.72, 37.70, 33.96, 29.12, 28.75, 14.63.

HRMS (ESI) calcd for $C_{28}H_{25}ClO_6$ $[M + H]^+$ 493.14124 and 495.13829, found 493.14072 and 495.13780, respectively.

$[\alpha]_D^{20}$ +15.7 (*c* 0.26, $CHCl_3$).

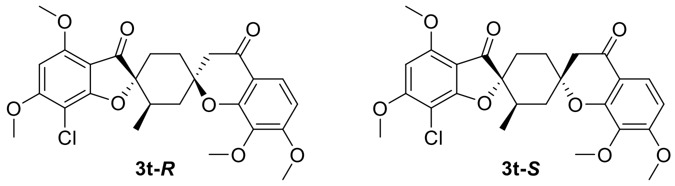

Following the representative procedure for grisofulvin-chromanones 100 ºC for 1 h (10:1 dr), 7 mg (5%) of **3t-*R*** and 68 mg (44%) of **3t-*S*** were isolated.

Minor diastereomer **3t-*R***:

^{1}H NMR (700 MHz, Chloroform-*d*) δ 7.63 (d, *J* = 8.8 Hz, 1H), 6.60 (d, *J* = 8.9 Hz, 1H), 6.08 (s, 1H), 4.00 (s, 3H), 3.96 (s, 3H), 3.92 (s, 3H), 3.91 (s, 3H), 3.00 – 2.92 (m, 2H), 2.61 (td, *J* = 13.0, 5.3 Hz, 1H), 2.50 (t, *J* = 12.8 Hz, 1H), 2.29 – 2.22 (m, 1H), 2.10 – 2.02 (m, 2H), 2.02 – 1.94 (m, 2H), 0.89 (d, *J* = 6.8 Hz, 3H).

^{13}C NMR (176 MHz, $CDCl_3$) δ 197.62, 190.83, 168.17, 164.24, 159.13, 157.84, 153.83, 137.58, 122.57, 115.96, 105.68, 105.28, 97.42, 91.43, 89.10, 81.12, 61.19, 57.03, 56.41, 56.35, 44.70, 37.88, 35.20, 30.05, 28.84, 15.18.

HRMS (ESI) calcd for $C_{26}H_{27}ClO_8$ $[M + H]^+$ 503.14672 and 505.14377, found 503.14639 and 505.14345, respectively.

$[\alpha]_D^{20}$ +32.9 (*c* 0.14, $CHCl_3$).

Major diastereomer **3t-*S***:

^{1}H NMR (600 MHz, Chloroform-*d*) δ 7.64 (d, *J* = 8.8 Hz, 1H), 6.60 (d, *J* = 8.9 Hz, 1H), 6.06 (s, 1H), 3.99 (s, 3H), 3.95 (s, 3H), 3.93 (s, 3H), 3.91 (s, 3H), 2.76 – 2.64 (m, 3H), 2.40 (td, *J* = 13.5, 4.0 Hz, 1H), 2.30 (td, *J* = 14.1, 4.0 Hz, 1H), 2.21 (dd, *J* = 14.4, 12.9 Hz, 1H), 2.14 – 2.08 (m, 1H), 2.05 (ddd, *J* = 14.4, 4.3, 2.8 Hz, 1H), 1.74 – 1.70 (m, 1H), 0.76 (d, *J* = 6.8 Hz, 3H).

^{13}C NMR (151 MHz, $CDCl_3$) δ 198.54, 191.10, 168.44, 164.17, 159.14, 157.66, 153.22, 137.59, 122.59, 115.98, 105.87, 105.22, 97.24, 91.86, 88.92, 80.06, 61.27, 56.97, 56.34, 56.28, 48.07, 37.76, 33.55, 29.09, 28.40, 14.63.

HRMS (ESI) calcd for $C_{26}H_{27}ClO_8$ $[M + H]^+$ 503.14672 and 505.14377, found 503.14620 and 505.14335, respectively.

$[\alpha]_D^{20}$ +26.5 (*c* 0.15, $CHCl_3$).

Structural Determination of Grismonone (3c-*R*)

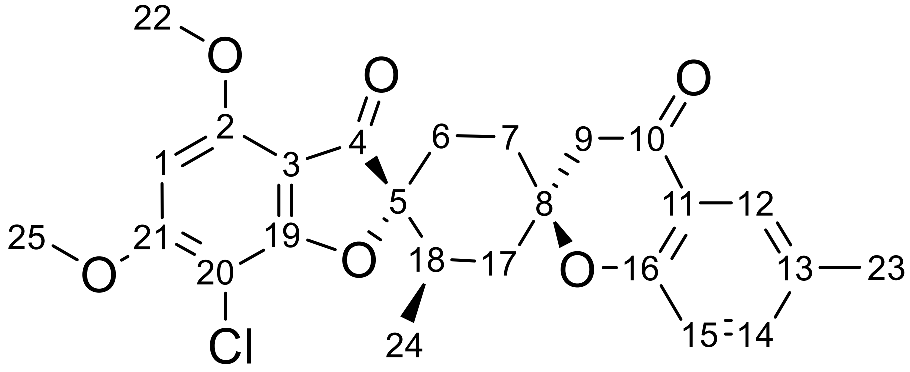

Carbon	^{13}C ppm	^{1}H ppm
24	15.11	0.84 (3*H*)
23	20.31	2.23 (3*H*)
6	27.90	1.80-2.00 (2*H*)
7	30.03	2.40, 1.95 (2*H*)
18	34.80	2.13 (1*H*)
17	37.08	2.30, 1.91 (2*H*)
9	45.17	2.87 (2*H*)
22 or 25	56.21	3.90 or 3.93 (3*H*)
22 or 25	56.80	3.90 or 3.93 (3*H*)
8	79.85	
1	88.84	6.01 (1*H*)
5	91.35	
3 or 20	97.17	
3 or 20	105.37	
15	118.29	6.83 (1*H*)
11	119.96	
12	125.97	7.57 (1*H*)
13	130.22	
14	137.34	7.23 (1*H*)
2 or 21	157.63	
16	157.72	
2 or 21	163.96	
19	167.83	
10	192.08	
4	197.44	

All assignments could be made with high confidence through analysis of 1H, 13C, COSY, HSQC, HMBC, and NOESY-NMRs. However, three sets of carbons could not be unambiguously determined (22 and 25; 3 and 20; 2 and 21). An NOE correlation was found between the hydrogens on carbon 9 and 18 and indicates the stereocenter of the newly formed spirocyclic carbon to be *R* (highlighted in yellow).

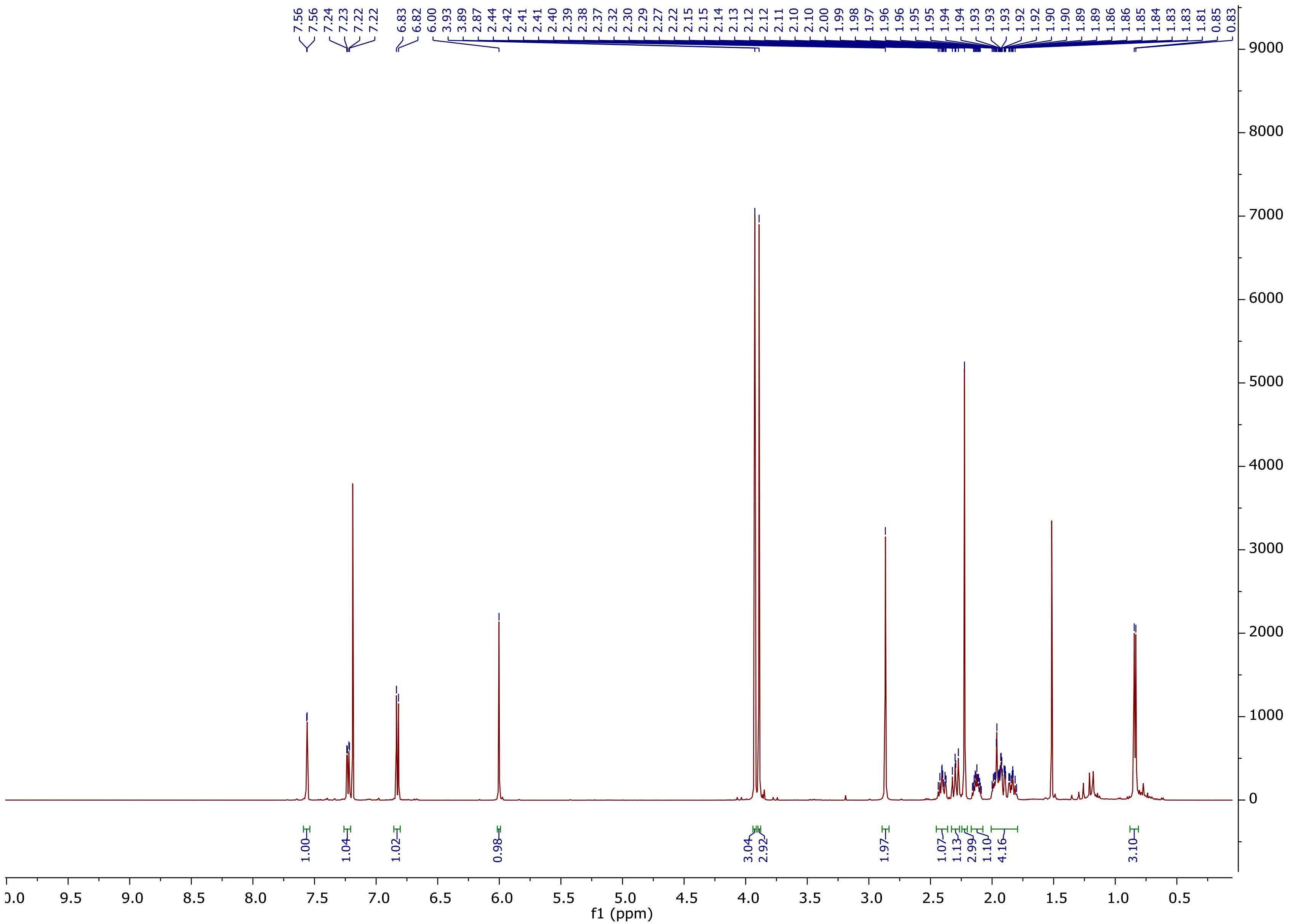

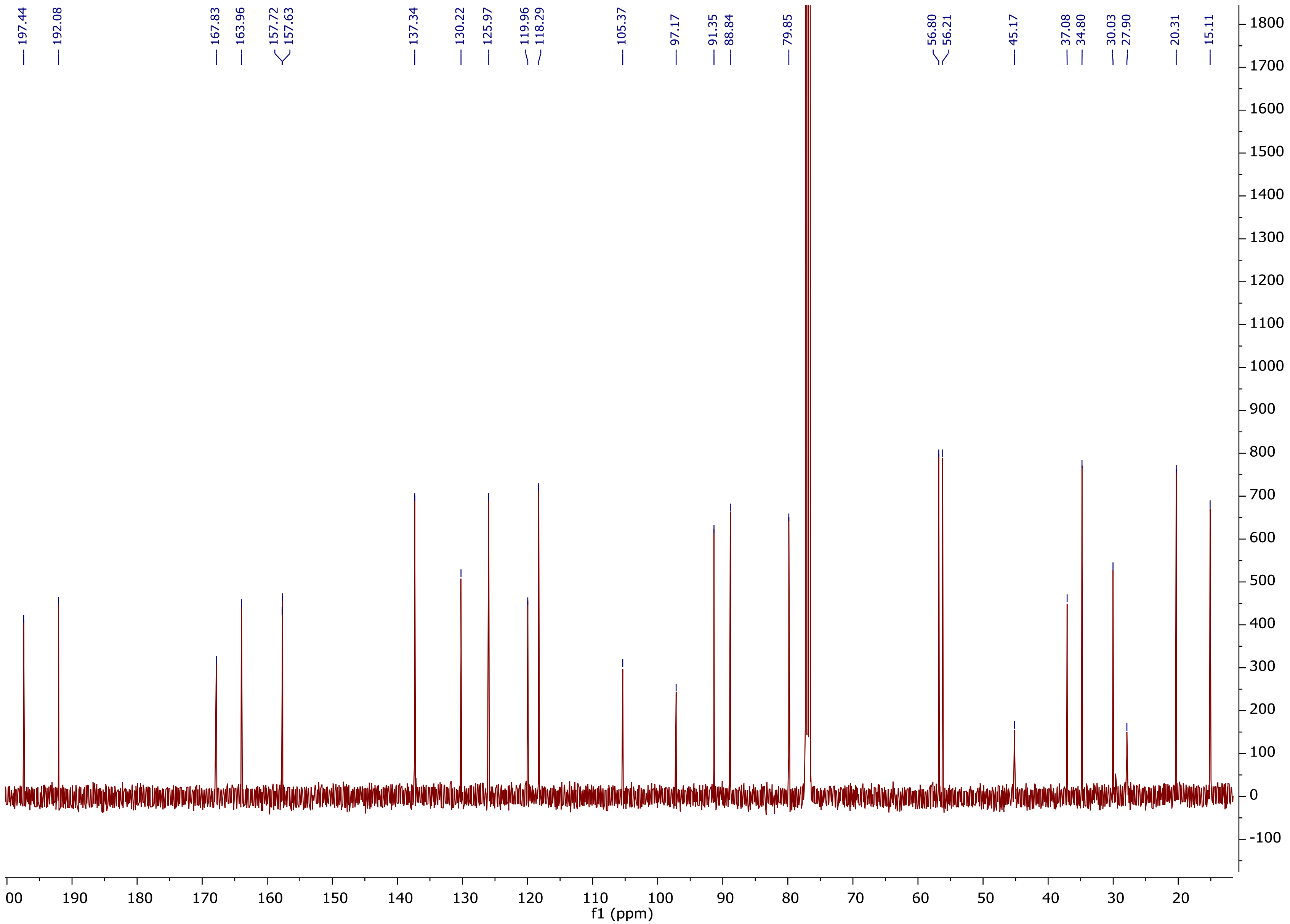

197.44
192.08
167.83
163.96
157.72
157.63
137.34
130.22
125.97
119.96
118.29
105.37
97.17
91.35
88.84
79.85
56.80
56.21
45.17
37.08
34.80
30.03
27.90
20.31
15.11
f1 (ppm)

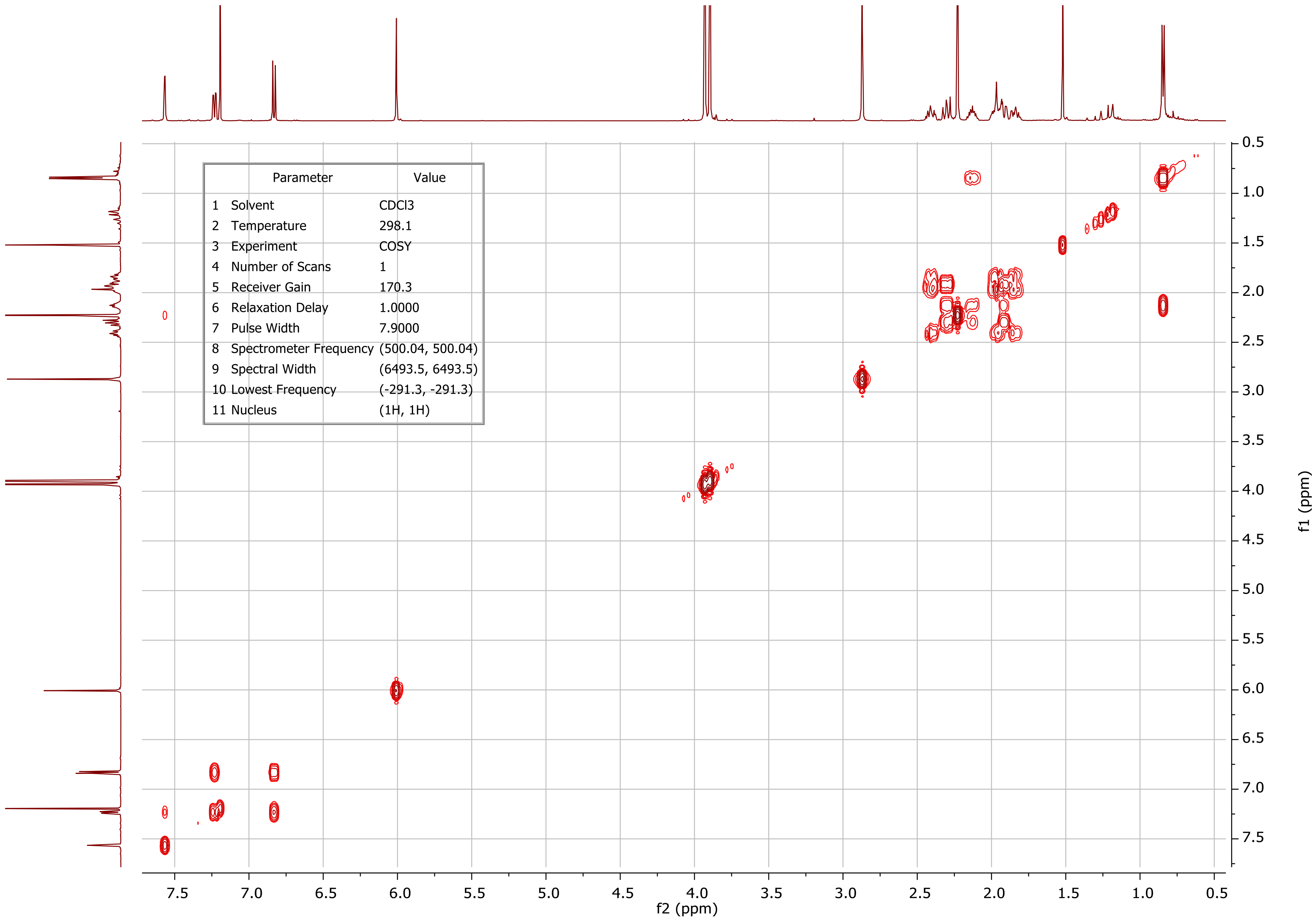

	Parameter	Value
1	Solvent	CDCl3
2	Temperature	298.1
3	Experiment	COSY
4	Number of Scans	1
5	Receiver Gain	170.3
6	Relaxation Delay	1.0000
7	Pulse Width	7.9000
8	Spectrometer Frequency	(500.04, 500.04)
9	Spectral Width	(6493.5, 6493.5)
10	Lowest Frequency	(-291.3, -291.3)
11	Nucleus	(1H, 1H)

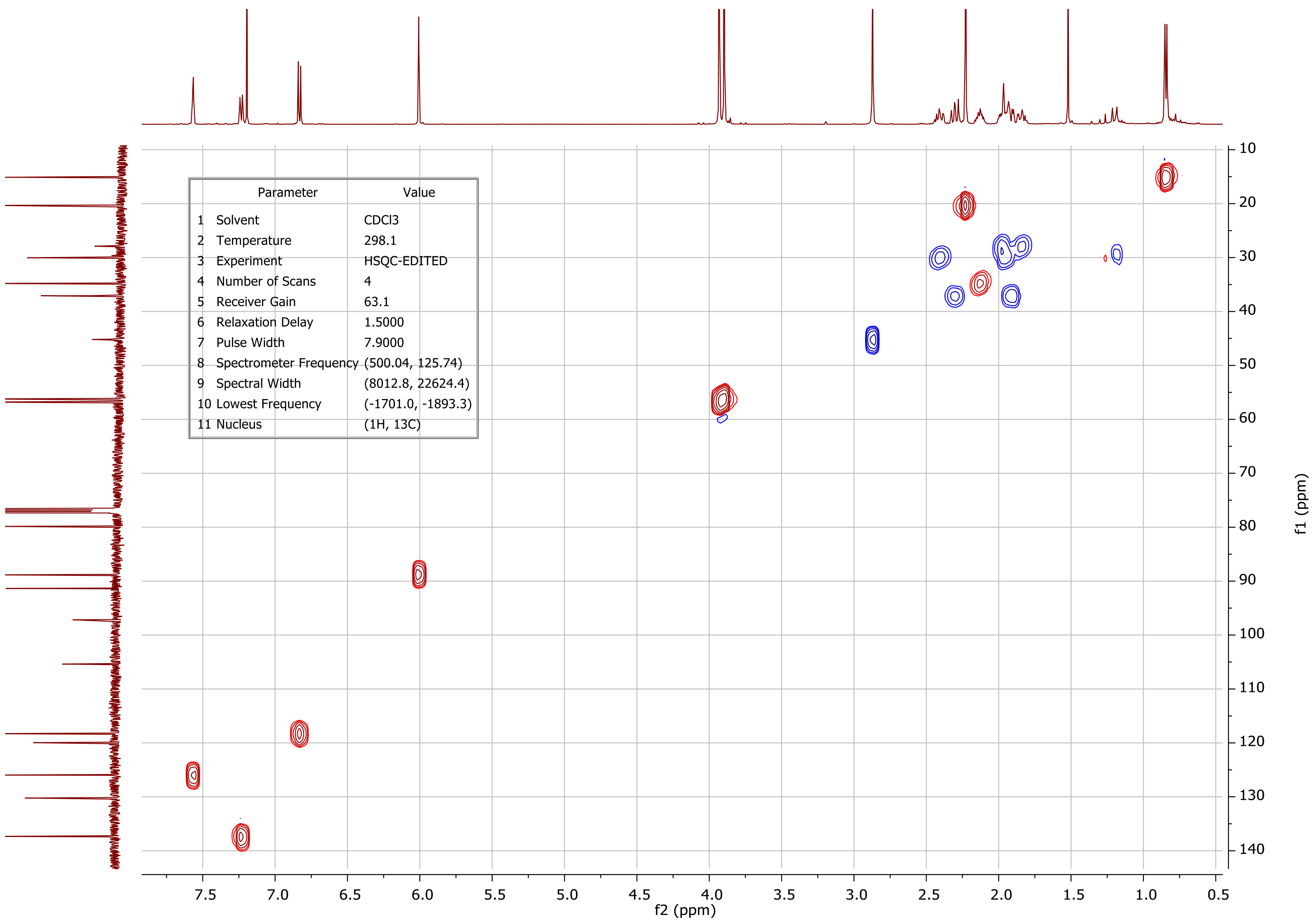

	Parameter	Value
1	Solvent	CDCl3
2	Temperature	298.1
3	Experiment	HSQC-EDITED
4	Number of Scans	4
5	Receiver Gain	63.1
6	Relaxation Delay	1.5000
7	Pulse Width	7.9000
8	Spectrometer Frequency	(500.04, 125.74)
9	Spectral Width	(8012.8, 22624.4)
10	Lowest Frequency	(-1701.0, -1893.3)
11	Nucleus	(1H, 13C)

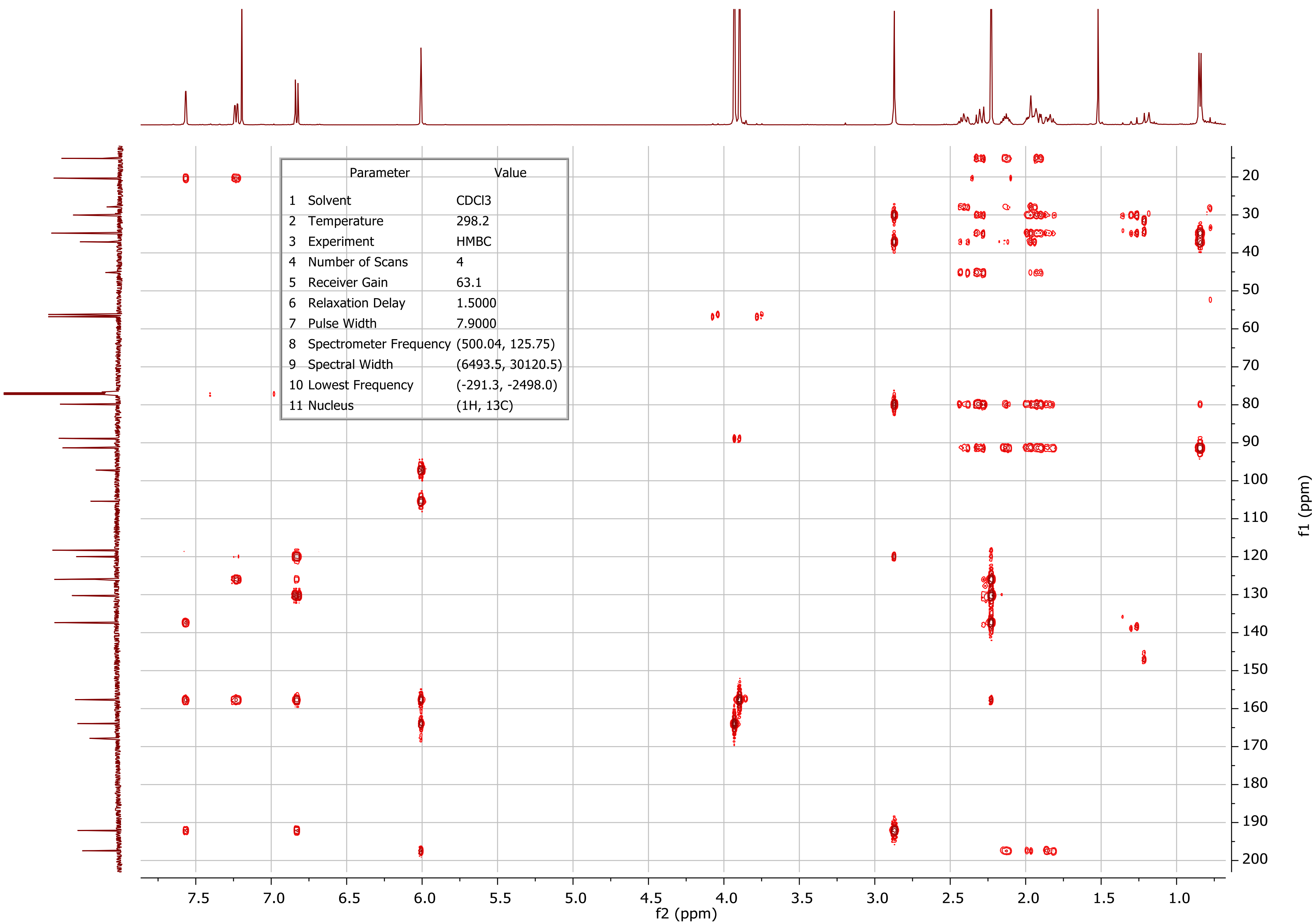

	Parameter	Value
1	Solvent	CDCl3
2	Temperature	298.2
3	Experiment	HMBC
4	Number of Scans	4
5	Receiver Gain	63.1
6	Relaxation Delay	1.5000
7	Pulse Width	7.9000
8	Spectrometer Frequency	(500.04, 125.75)
9	Spectral Width	(6493.5, 30120.5)
10	Lowest Frequency	(-291.3, -2498.0)
11	Nucleus	(1H, 13C)

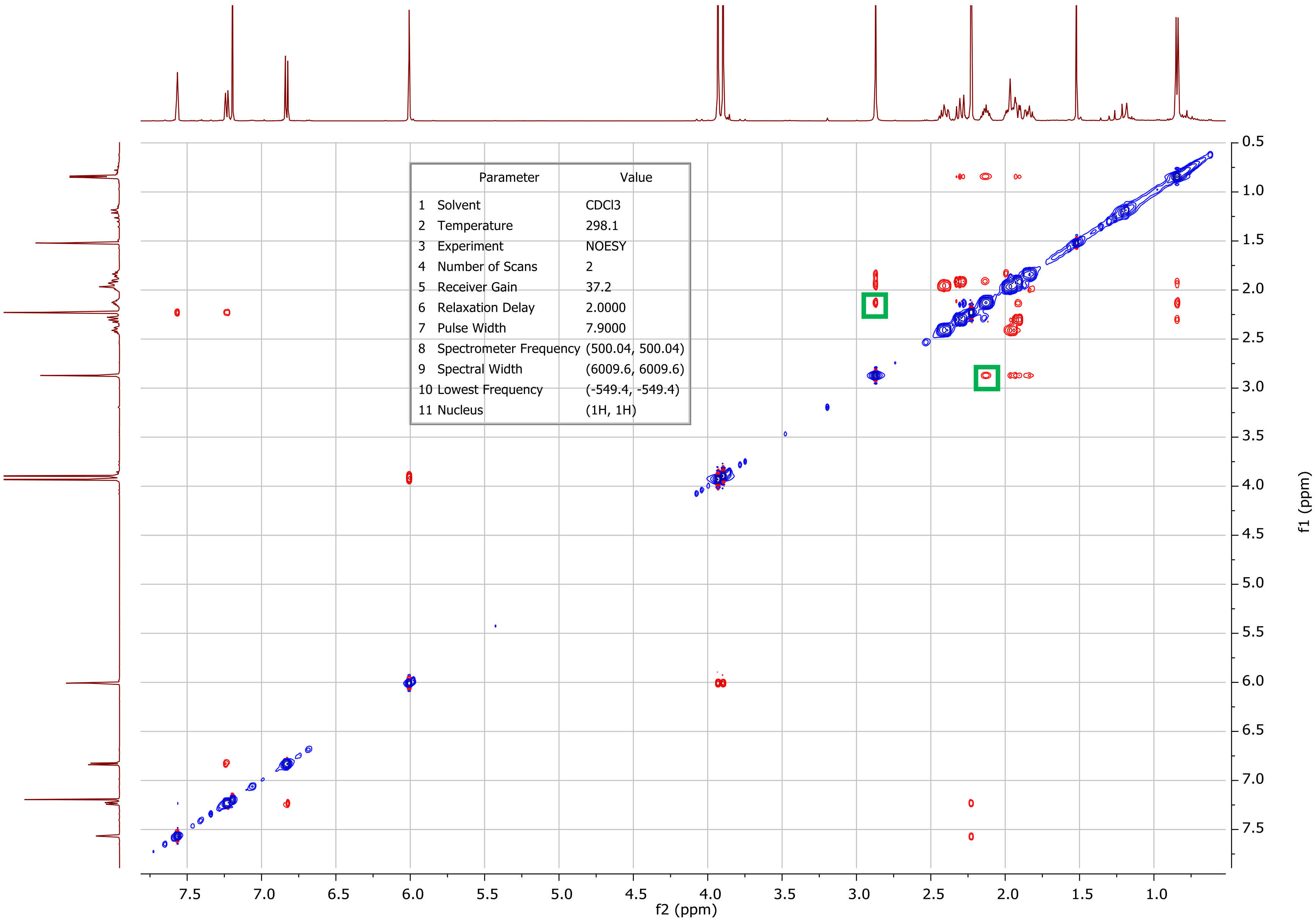

	Parameter	Value
1	Solvent	CDCl3
2	Temperature	298.1
3	Experiment	NOESY
4	Number of Scans	2
5	Receiver Gain	37.2
6	Relaxation Delay	2.0000
7	Pulse Width	7.9000
8	Spectrometer Frequency	(500.04, 500.04)
9	Spectral Width	(6009.6, 6009.6)
10	Lowest Frequency	(-549.4, -549.4)
11	Nucleus	(1H, 1H)

Stereochemical Determination of Selected G-Cs

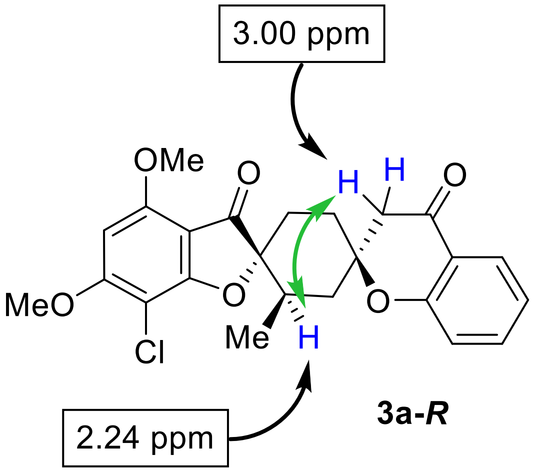

3a-*R*

A NOE correlation was found between the hydrogens at 3.00 ppm and 2.24 ppm and indicates the stereocenter of the newly formed spirocyclic carbon to be *R*.

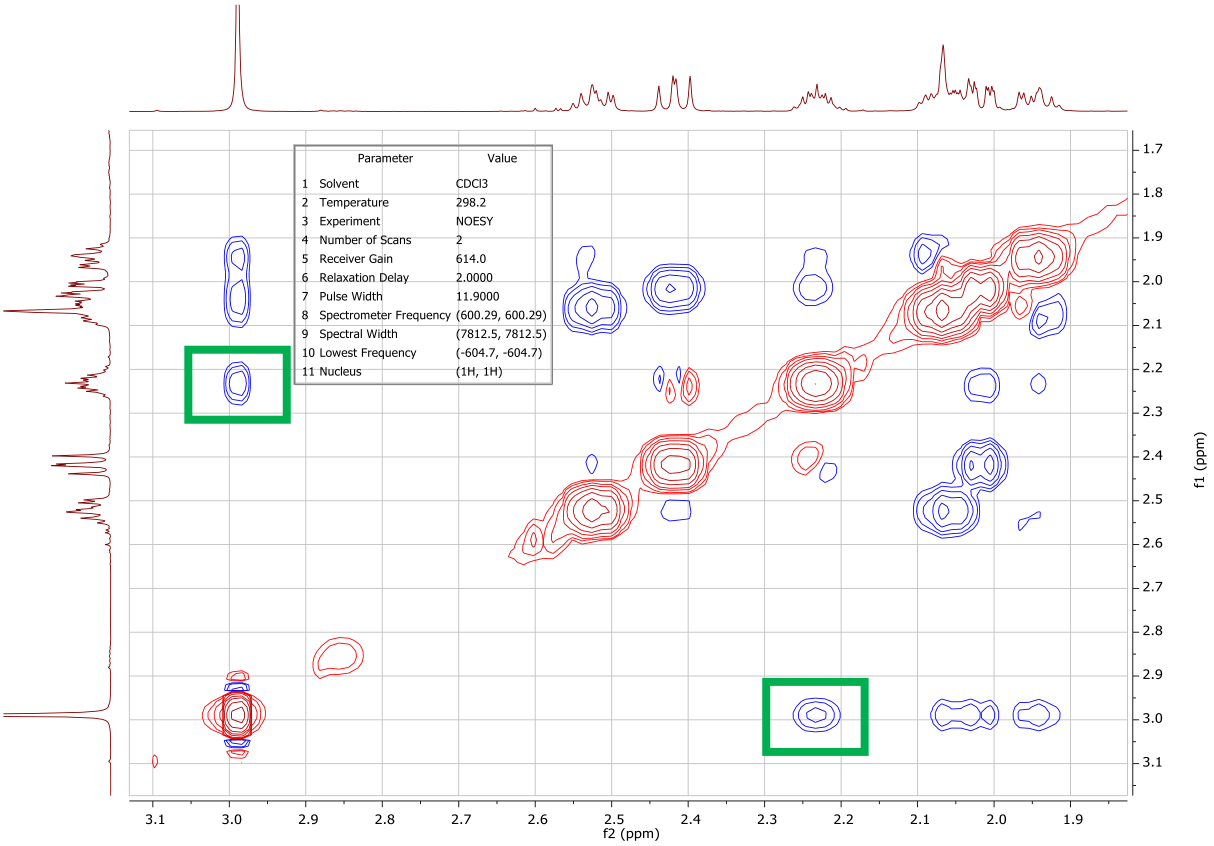

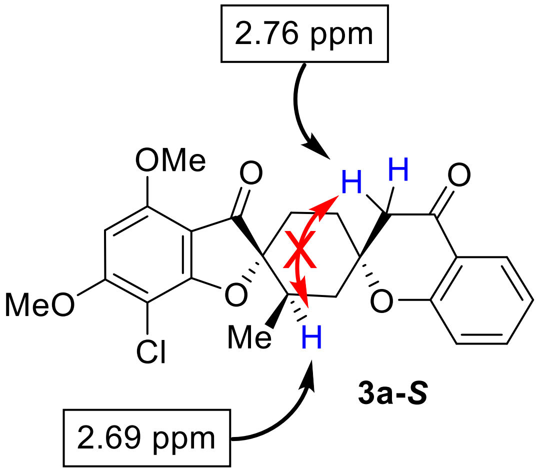

3a-*S*

No NOE correlation was found between the hydrogens at 2.76 ppm and 2.69 ppm and indicates the stereocenter of the newly formed spirocyclic carbon to be *S*.

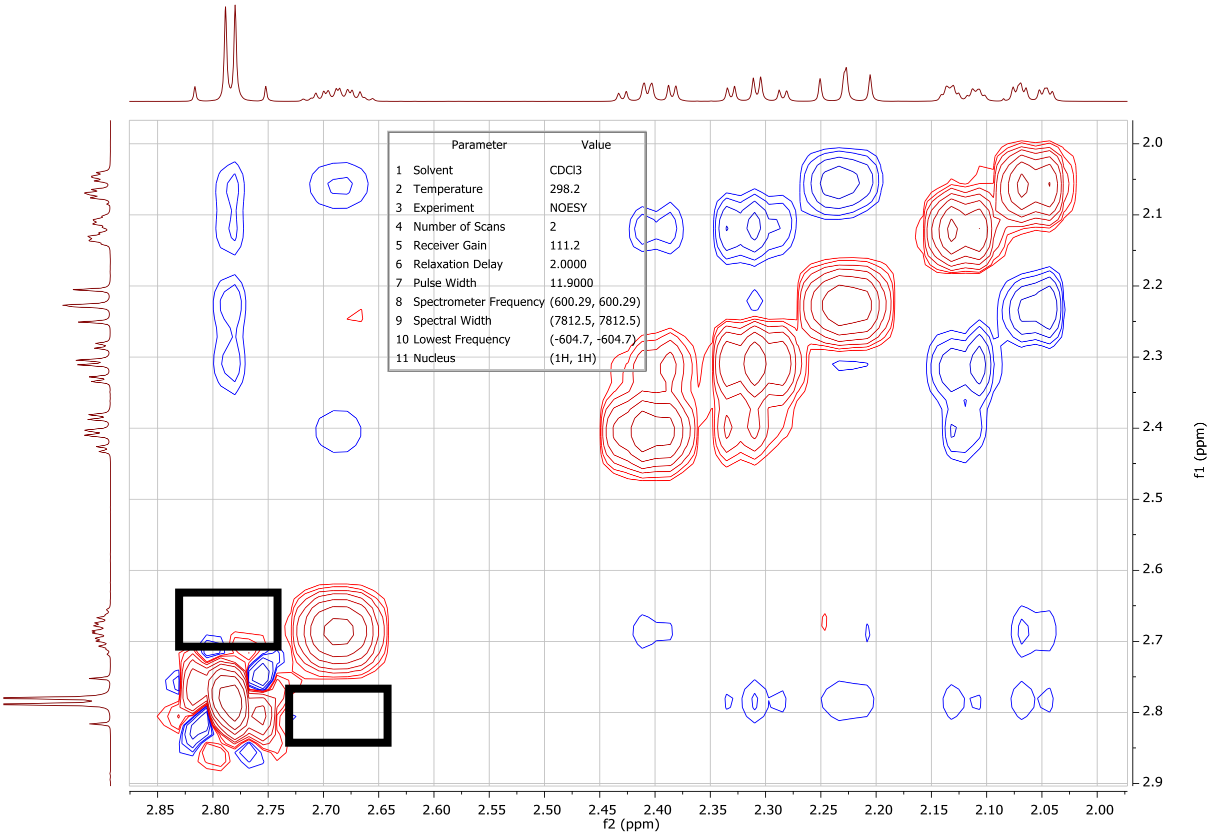

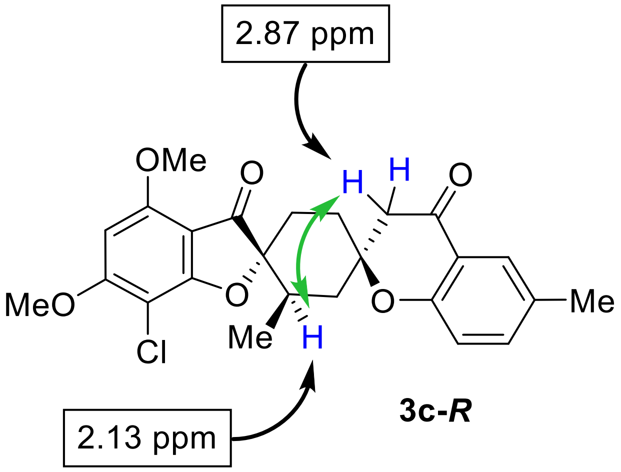

3c-*R*

A NOE correlation was found between the hydrogens at 2.87 ppm and 2.13 ppm and indicates the stereocenter of the newly formed spirocyclic carbon to be *R*.

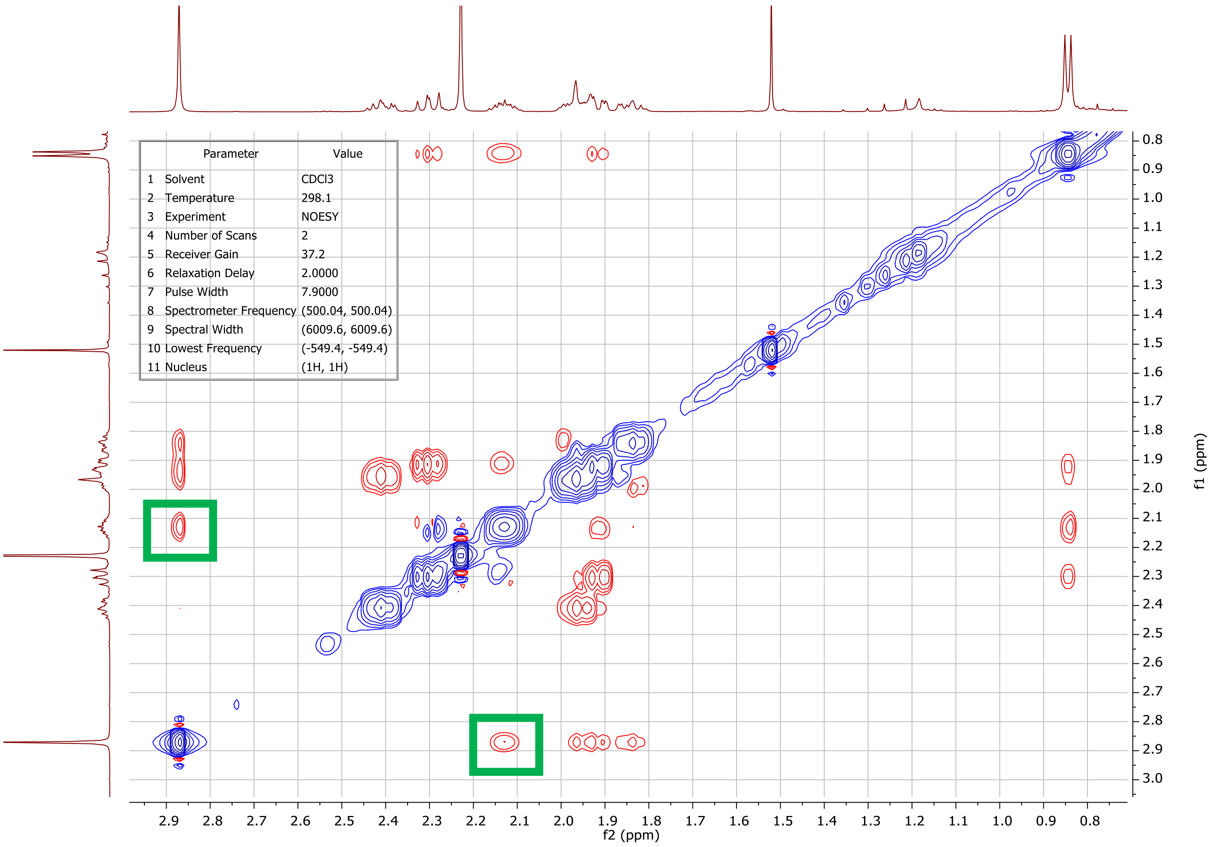

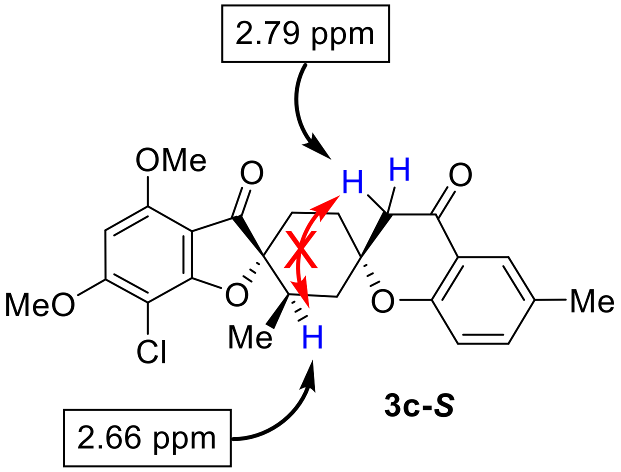

3c-*S*

No NOE correlation was found between the hydrogens at 2.79 ppm and 2.66 ppm and indicates the stereocenter of the newly formed spirocyclic carbon to be *S*.

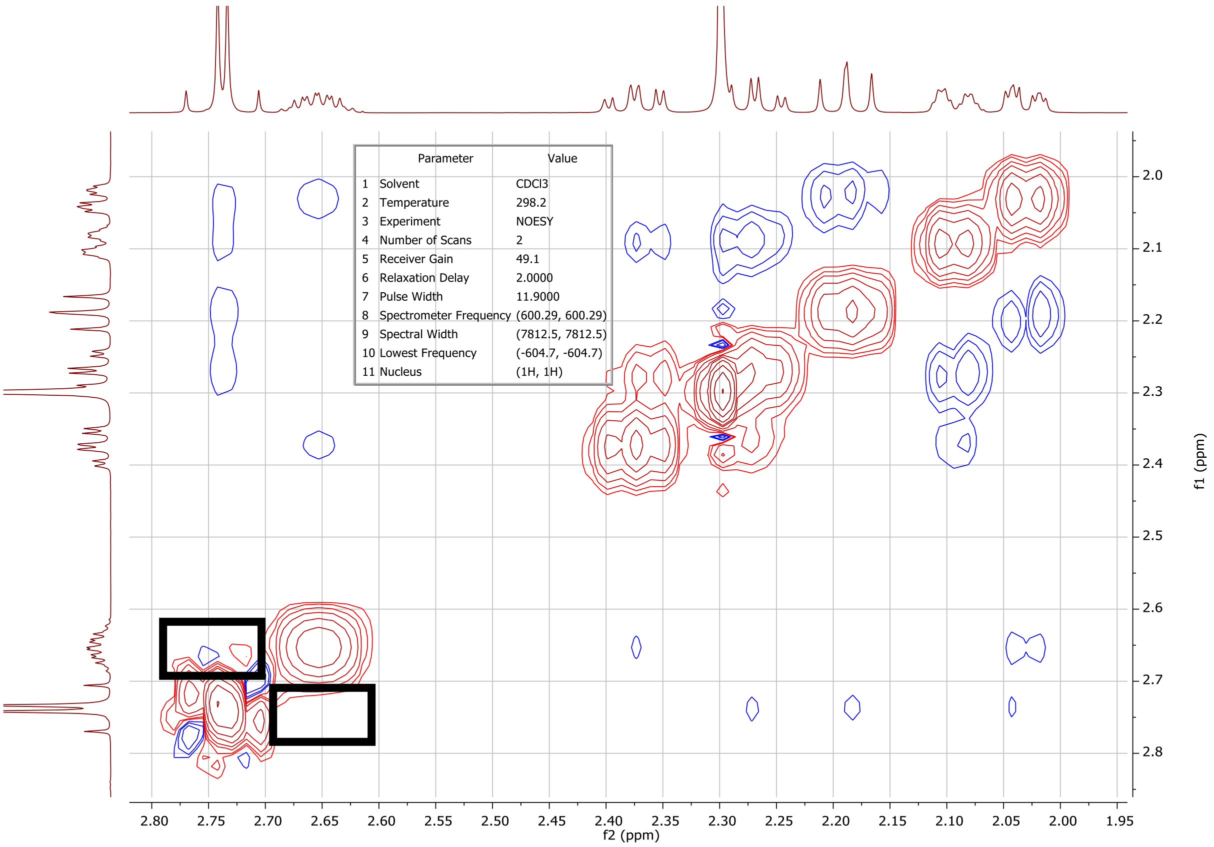

References

[1] P. Ertl, S. Roggo, A. Schuffenhauer, *J. Chem. Inf. Model.* **2008**, *48*, 68.

[2] G. Liu, D. Xue, J. Yang, J. Wang, X. Liu, W. Huang, J. Li, Y. Q. Long, W. Tan, A. Zhang, *J. Med. Chem.* **2016**, *59*, 11050.

[3] S. Berardozzi, F. Bernardi, P. Infante, C. Ingallina, S. Toscano, E. De Paolis, R. Alfonsi, M. Caimano, B. Botta, M. Mori, et al., *Eur. J. Med. Chem.* **2018**, *156*, 554.

[4] J. Talpale, J. K. Chen, M. K. Cooper, B. Wang, R. K. Mann, L. Milenkovic, M. P. Scott, P. A. Beachy, *Nature* **2000**, *406*, 1005.

[5] H. Sasaki, C. Hui, M. Nakafuku, H. Kondoh, *Development* **1997**, *1322*, 1313.

[6] X. Wu, S. Ding, Q. Ding, N. S. Gray, P. G. Schultz, *J. Am. Chem. Soc.* **2002**, *124*, 14520.

[7] R. J. Lipinski, J. J. Gipp, J. Zhang, J. D. Doles, W. Bushman, *Exp. Cell Res* **2006**, *312*, 1925.

[8] G. Regl, G. W. Neill, T. Eichberger, M. Kasper, M. S. Ikram, J. Koller, H. Hintner, A. G. Quinn, A. M. Frischauf, F. Aberger, *Oncogene* **2002**, *21*, 5529.

[9] M. W. Pfaffl, *Nucleic Acids Res.* **2001**, *29*, e45.

[10] S. Sinha, J. K. Chen, *Nat. Chem. Biol.* **2006**, *2*, 29.

[11] D. R. Stirling, M. J. Swain-Bowden, A. M. Lucas, A. E. Carpenter, B. A. Cimini, A. Goodman, *BMC Bioinformatics* **2021**, *22*, 443.

[12] G. Madhavi Sastry, M. Adzhigirey, T. Day, R. Annabhimoju, W. Sherman, *J. Comput. Aided Mol. Des.* **2013**, *27*, 221.

[13] R. A. Friesner, J. L. Banks, R. B. Murphy, T. A. Halgren, J. J. Klicic, D. T. Mainz, M. P. Repasky, E. H. Knoll, M. Shelley, J. K. Perry, et al., *J. Med. Chem.* **2004**, *47*, 1750.

[14] T. A. Halgren, R. B. Murphy, R. A. Friesner, H. S. Beard, L. L. Frye, W. T. Pollard, J. L. Banks, *J. Med. Chem.* **2004**, *47*, 1750.

[15] W. Sherman, T. Day, M. P. Jacobson, R. A. Friesner, R. Farid, *J. Med. Chem.* **2006**, *49*, 534.

[16] W. Sherman, H. S. Beard, R. Farid, *Chem. Biol. Drug Des.* **2006**, *67*, 83.

[17] M. P. Jacobson, R. A. Friesner, Z. Xiang, B. Honig, *J. Mol. Bio.* **2002**, *320*, 597.

[18] M. P. Jacobson, D. L. Pincus, C. S. Rapp, T. J. F. Day, B. Honig, D. E. Shaw, R. A. Friesner, *Proteins Struct. Funct. Bioinforma.* **2004**, *55*, 351.

[19] J. Li, R. Abel, K. Zhu, Y. Cao, S. Zhao, R. A. Friesner, *Proteins Struct. Funct. Bioinforma.* **2011**, *79*, 2794.

[20] G. R. Fulmer, A. J. M. Miller, N. H. Sherden, H. E. Gottlieb, A. Nudelman, B. M. Stoltz, J. E. Bercaw, K. I. Goldberg, *Organometallics* **2010**, *29*, 2176.

[21] M. Grigalunas, A. Burhop, S. Zinken, A. Pahl, J.-M. Gally, N. Wild, Y. Mantel, S.

Sievers, D. J. Foley, R. Scheel, et al., *Nat. Commun.* **2021**, *12*, 1883.

NMR Spectra

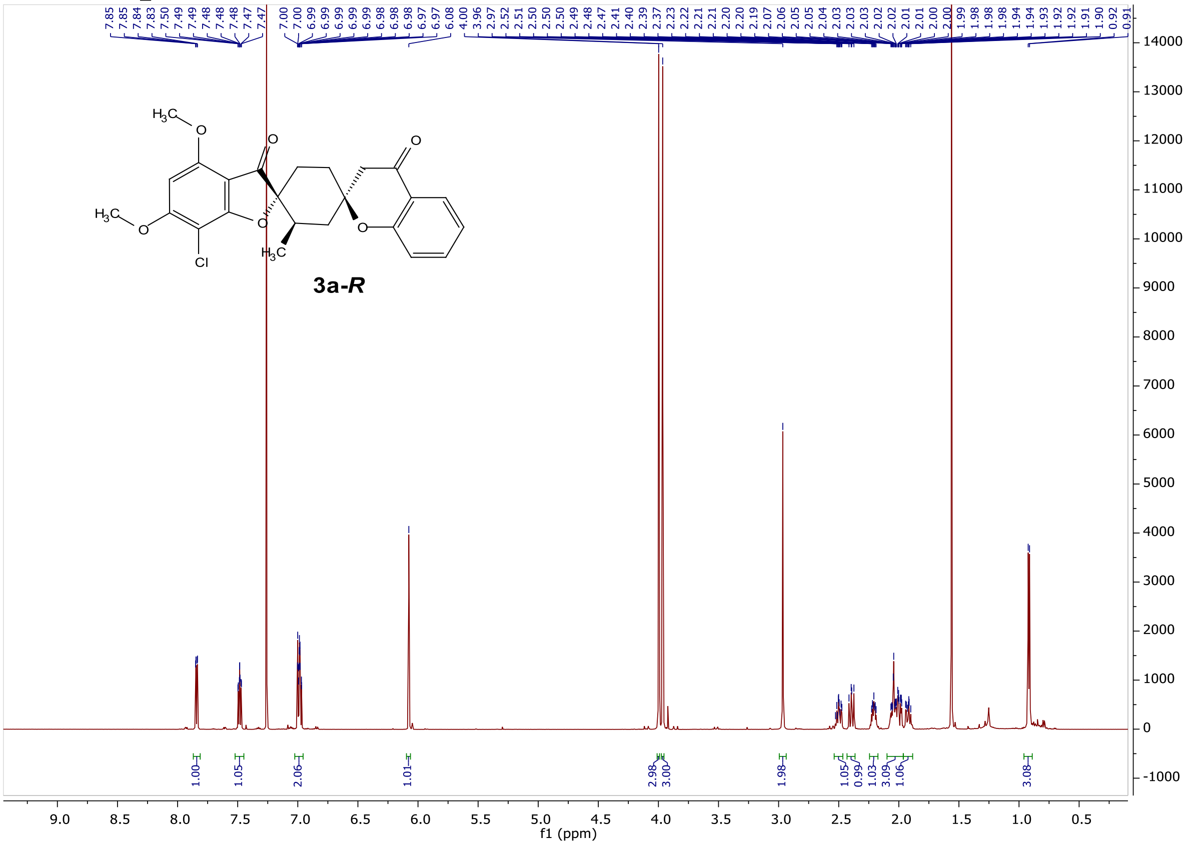
7.85 7.85 7.84 7.83 7.50 7.49 7.49 7.48 7.48 7.47 7.47 7.00 7.00 6.99 6.99 6.99 6.99 6.98 6.98 6.98 6.97 6.97 6.08 4.00 3.96 2.97 2.52 2.51 2.50 2.50 2.50 2.49 2.48 2.47 2.41 2.40 2.39 2.37 2.23 2.22 2.21 2.21 2.20 2.20 2.19 2.07 2.06 2.05 2.05 2.04 2.03 2.03 2.03 2.02 2.02 2.01 2.01 2.00 2.00 1.99 1.98 1.98 1.98 1.94 1.94 1.93 1.92 1.92 1.91 1.90 0.92 0.91
3a-R
1.00 1.05 2.06 1.01 2.98 3.00 1.98 1.05 0.99 1.03 3.09 1.06 3.08
f1 (ppm)

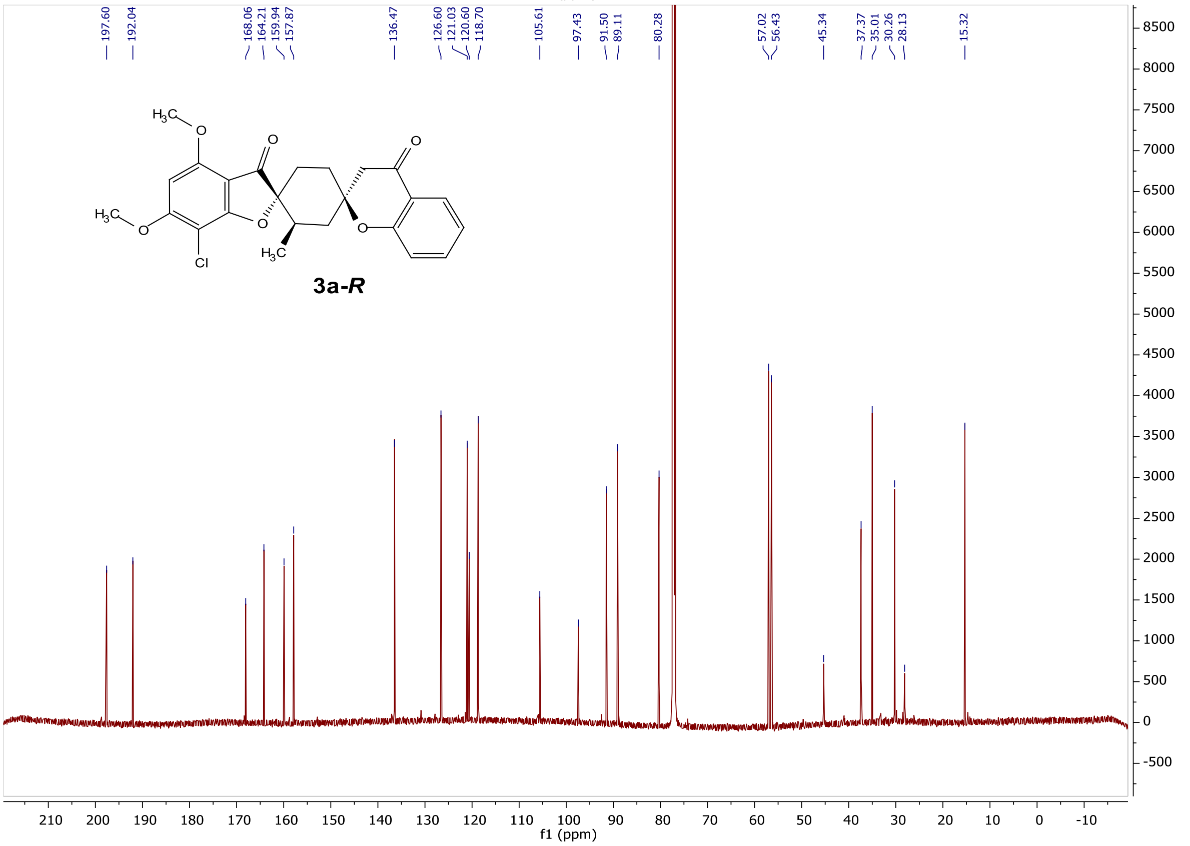
197.60 192.04 168.06 164.21 159.94 157.87 136.47 126.60 121.03 120.60 118.70 105.61 97.43 91.50 89.11 80.28 57.02 56.43 45.34 37.37 35.01 30.26 28.13 15.32
3a-R
f1 (ppm)

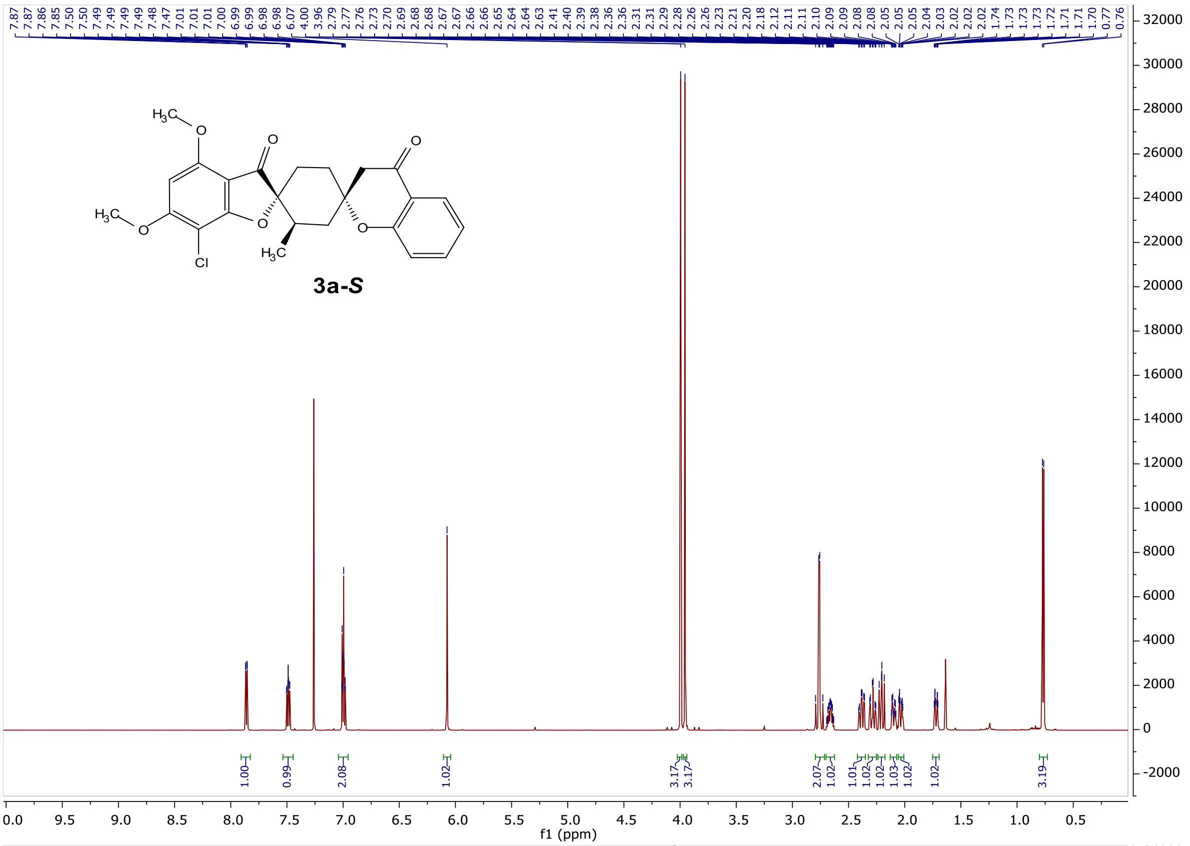
7.87 7.87 7.86 7.85 7.50 7.50 7.49 7.49 7.49 7.49 7.48 7.47 7.01 7.01 7.01 7.01 7.00 6.99 6.99 6.98 6.98 6.07 4.00 3.96 2.79 2.77 2.76 2.73 2.70 2.69 2.68 2.68 2.67 2.67 2.66 2.66 2.65 2.64 2.64 2.63 2.41 2.40 2.39 2.38 2.36 2.36 2.31 2.31 2.29 2.28 2.26 2.26 2.23 2.21 2.20 2.18 2.12 2.11 2.11 2.10 2.09 2.09 2.08 2.08 2.05 2.05 2.05 2.04 2.03 2.02 2.02 2.02 1.74 1.73 1.73 1.72 1.72 1.71 1.71 1.70 0.77 0.76
3a-*S*
1.00 0.99 2.08 1.02 3.17 3.17 2.07 1.02 1.01 1.02 1.02 1.02 1.03 1.02 1.02 3.19
f1 (ppm)

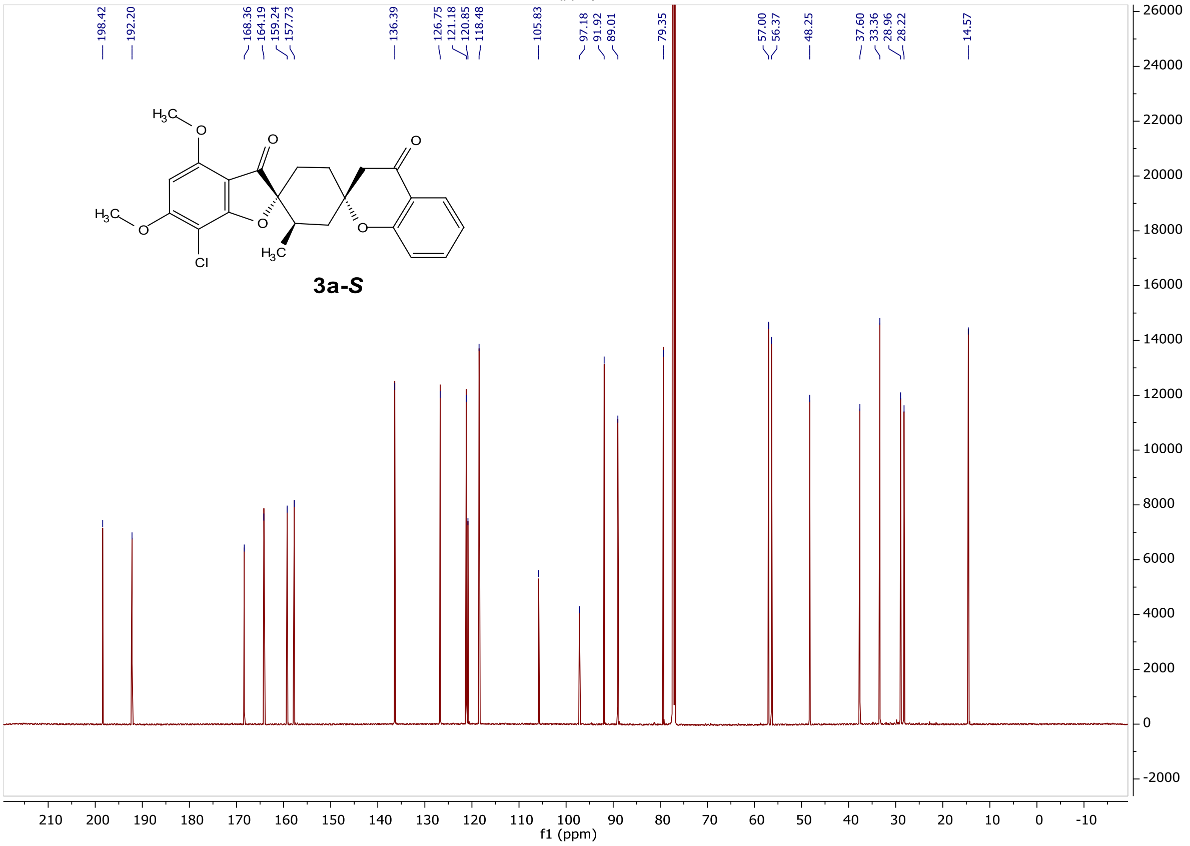
198.42 192.20 168.36 164.19 159.24 157.73 136.39 126.75 121.18 120.85 118.48 105.83 97.18 91.92 89.01 79.35 57.00 56.37 48.25 37.60 33.36 28.96 28.22 14.57
3a-*S*
f1 (ppm)

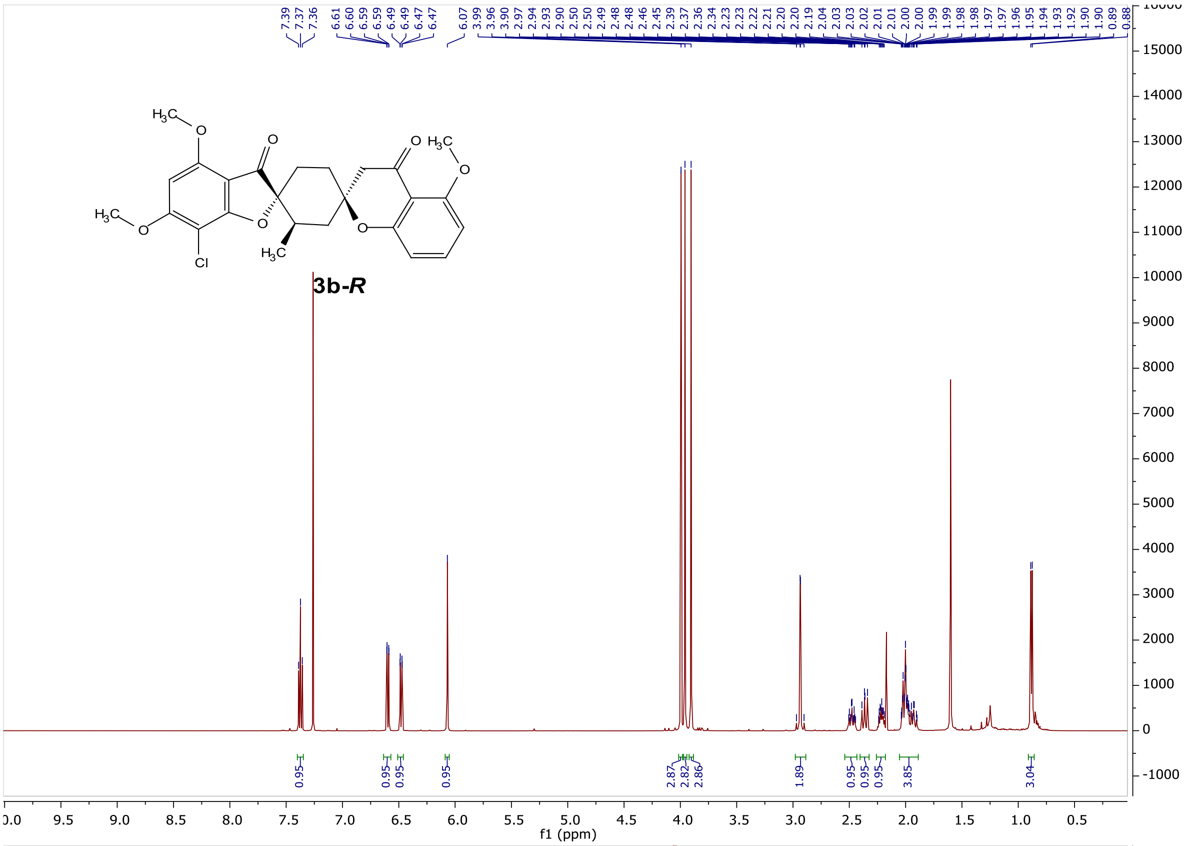
7.39 7.37 7.36 6.61 6.60 6.59 6.59 6.49 6.49 6.47 6.47 6.07 3.99 3.96 3.90 2.97 2.94 2.93 2.90 2.50 2.50 2.49 2.48 2.46 2.45 2.39 2.37 2.36 2.34 2.23 2.23 2.22 2.21 2.20 2.19 2.04 2.03 2.03 2.02 2.01 2.01 2.00 2.00 1.99 1.99 1.98 1.98 1.97 1.97 1.96 1.95 1.94 1.93 1.92 1.90 1.90 0.89 0.88
3b-*R*
0.95 0.95 0.95 0.95 2.87 2.82 2.86 1.89 0.95 0.95 0.95 3.85 3.04
f1 (ppm)

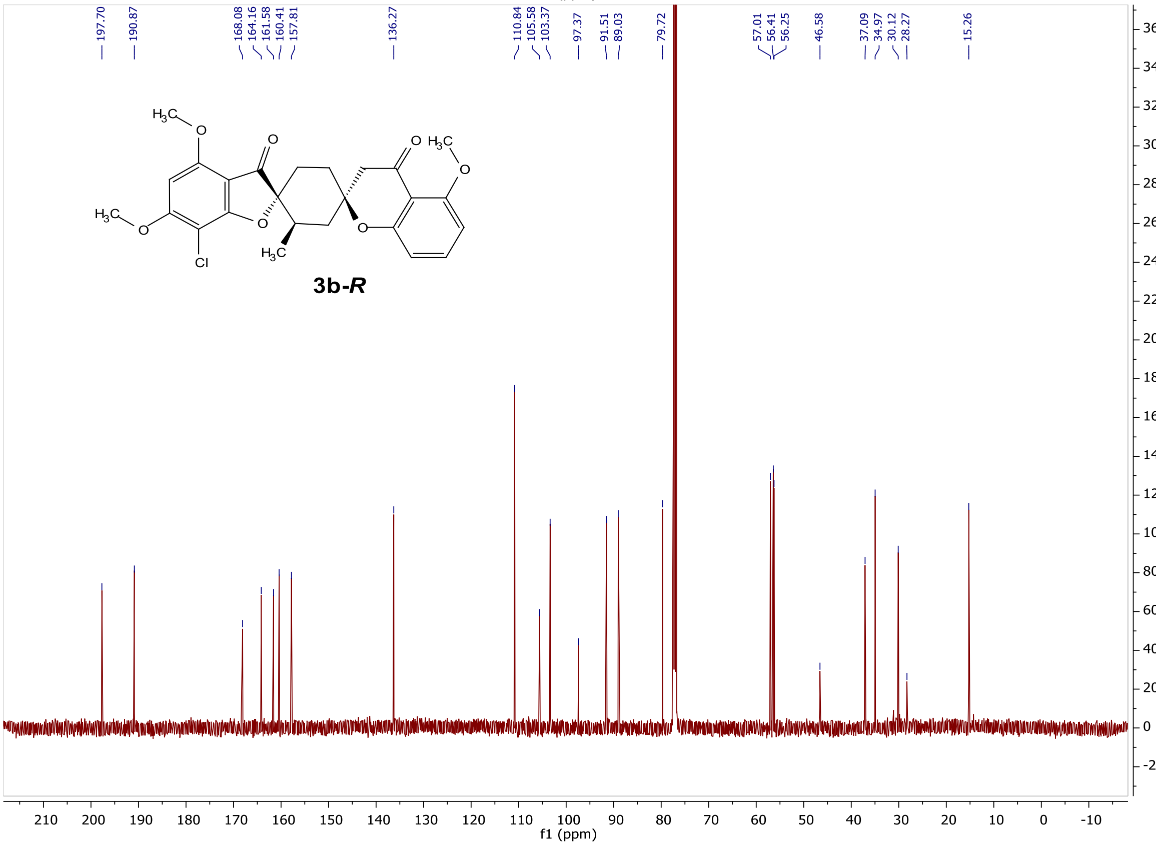
197.70 190.87 168.08 164.16 161.58 160.41 157.81 136.27 110.84 105.58 103.37 97.37 91.51 89.03 79.72 57.01 56.41 56.25 46.58 37.09 34.97 30.12 28.27 15.26
3b-*R*
f1 (ppm)

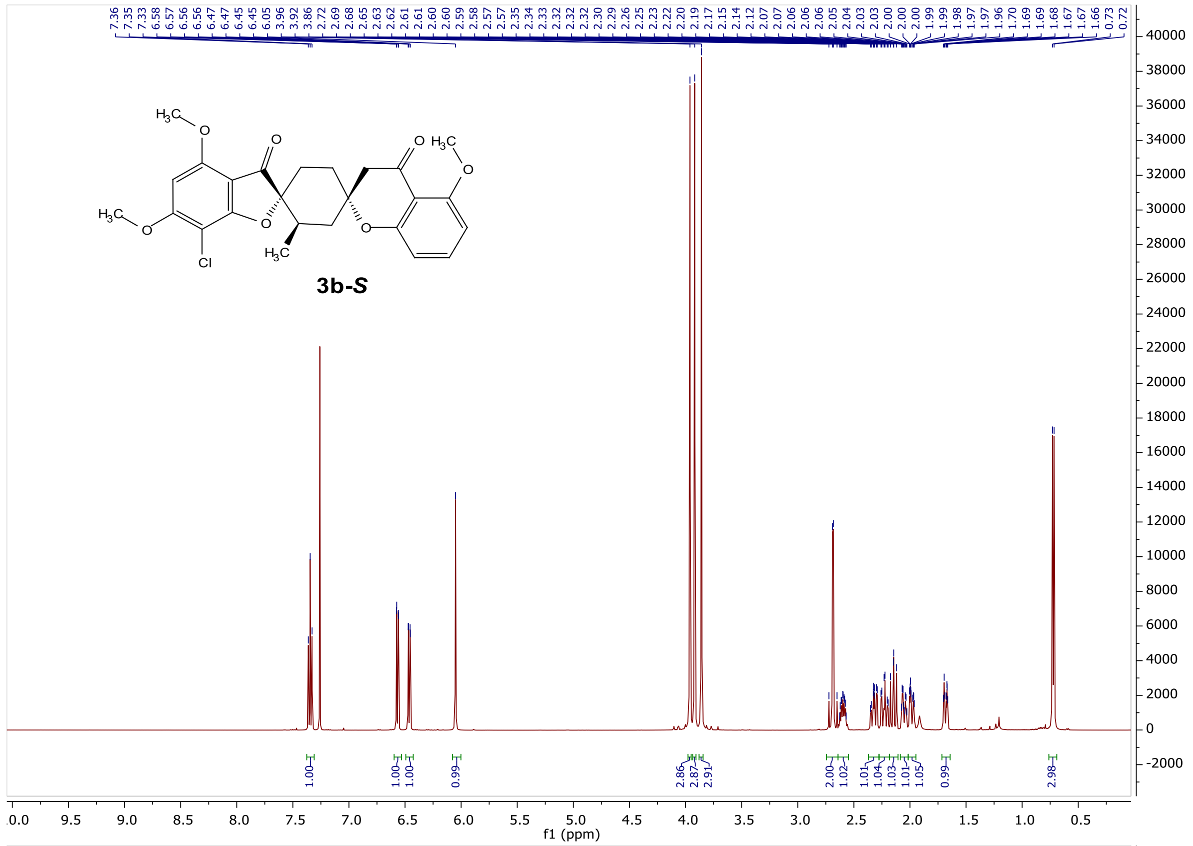
3b-S
f1 (ppm)

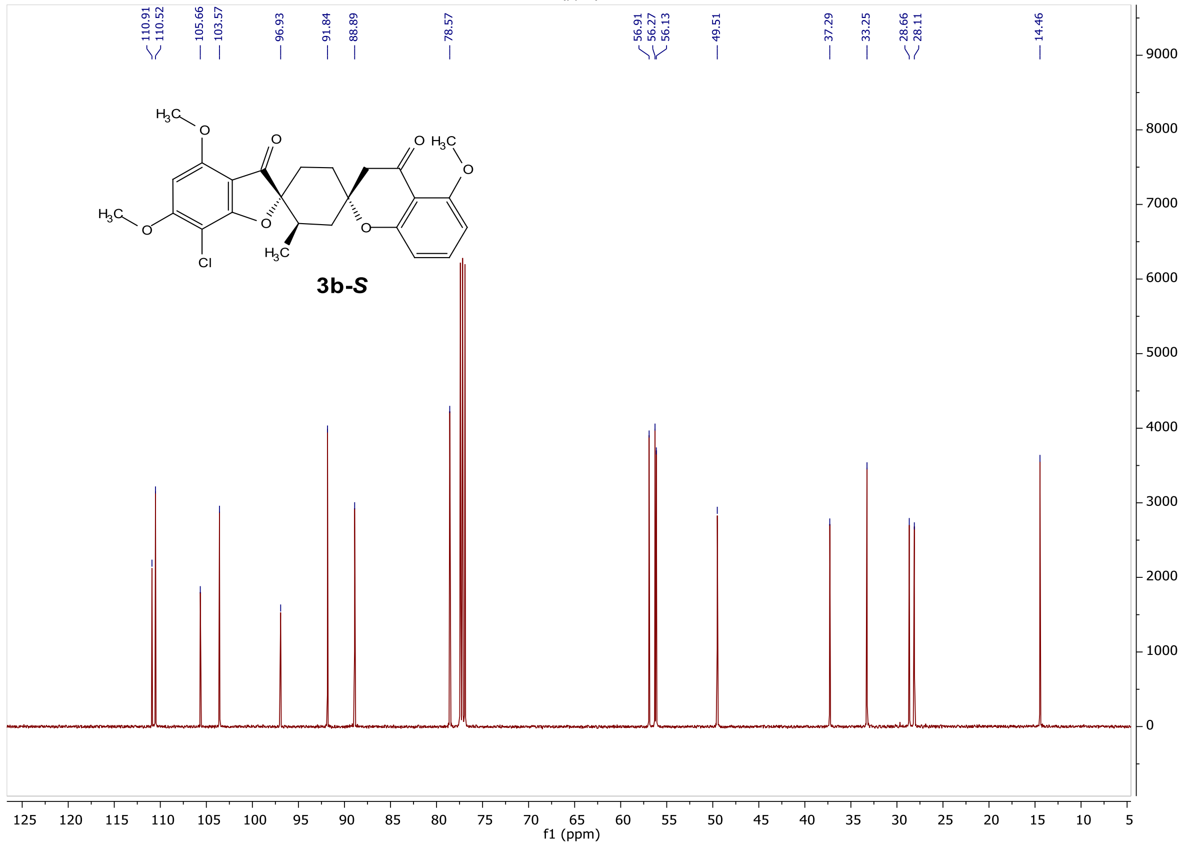
110.91
110.52
105.66
103.57
96.93
91.84
88.89
78.57
56.91
56.27
56.13
49.51
37.29
33.25
28.66
28.11
14.46
3b-S
f1 (ppm)

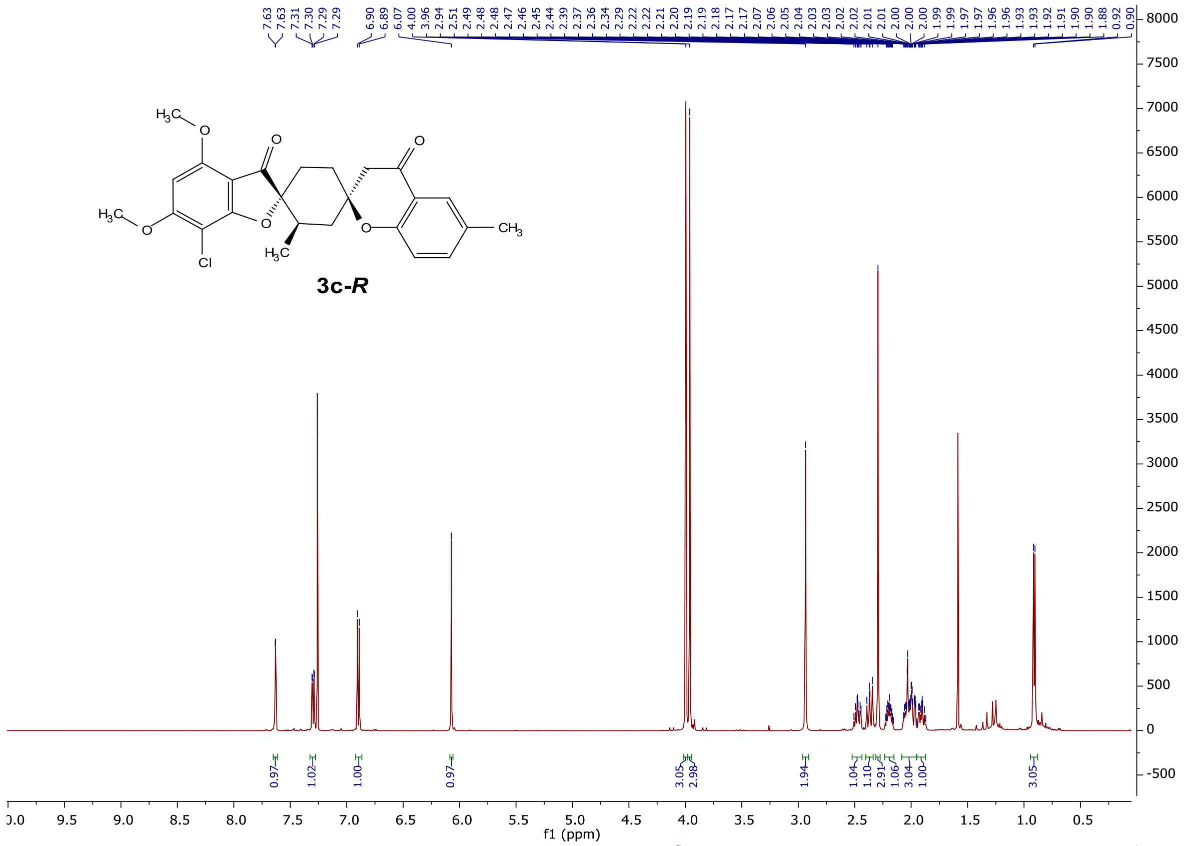

3c-*R*

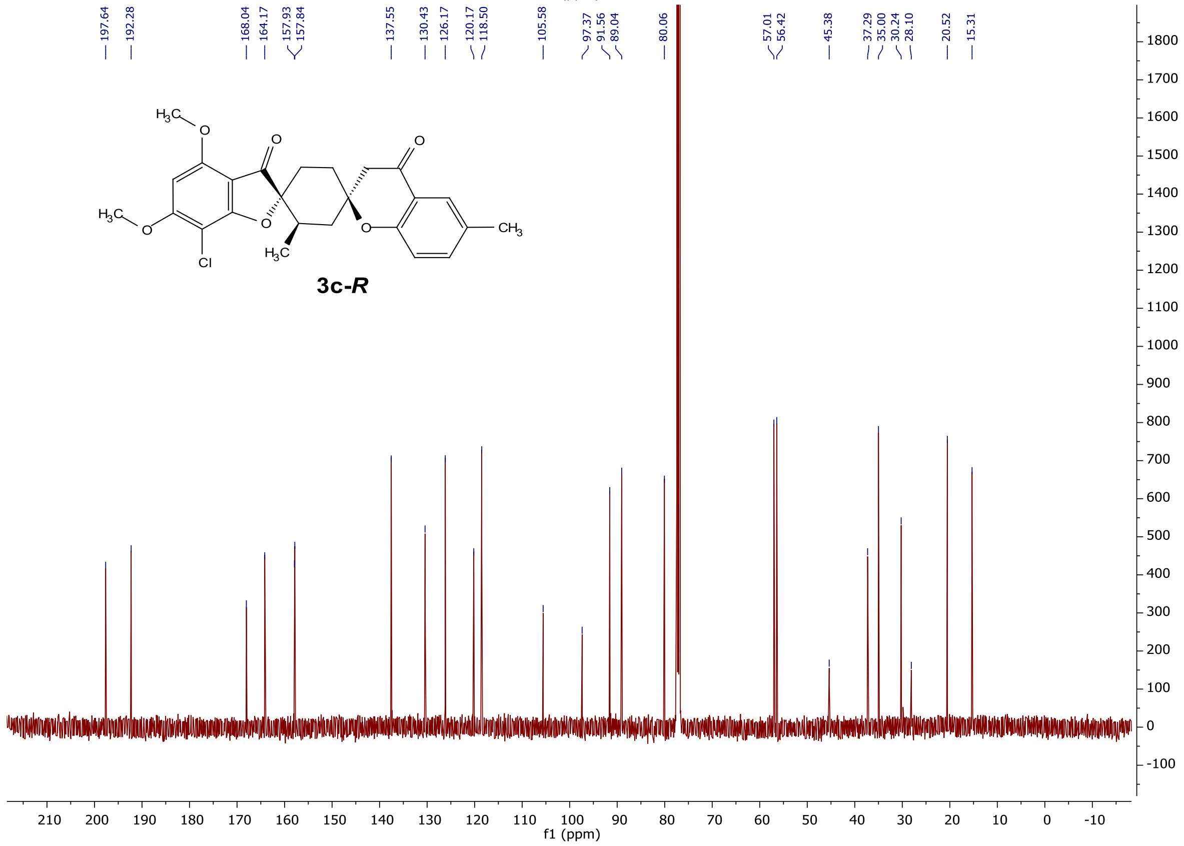

3c-*R*

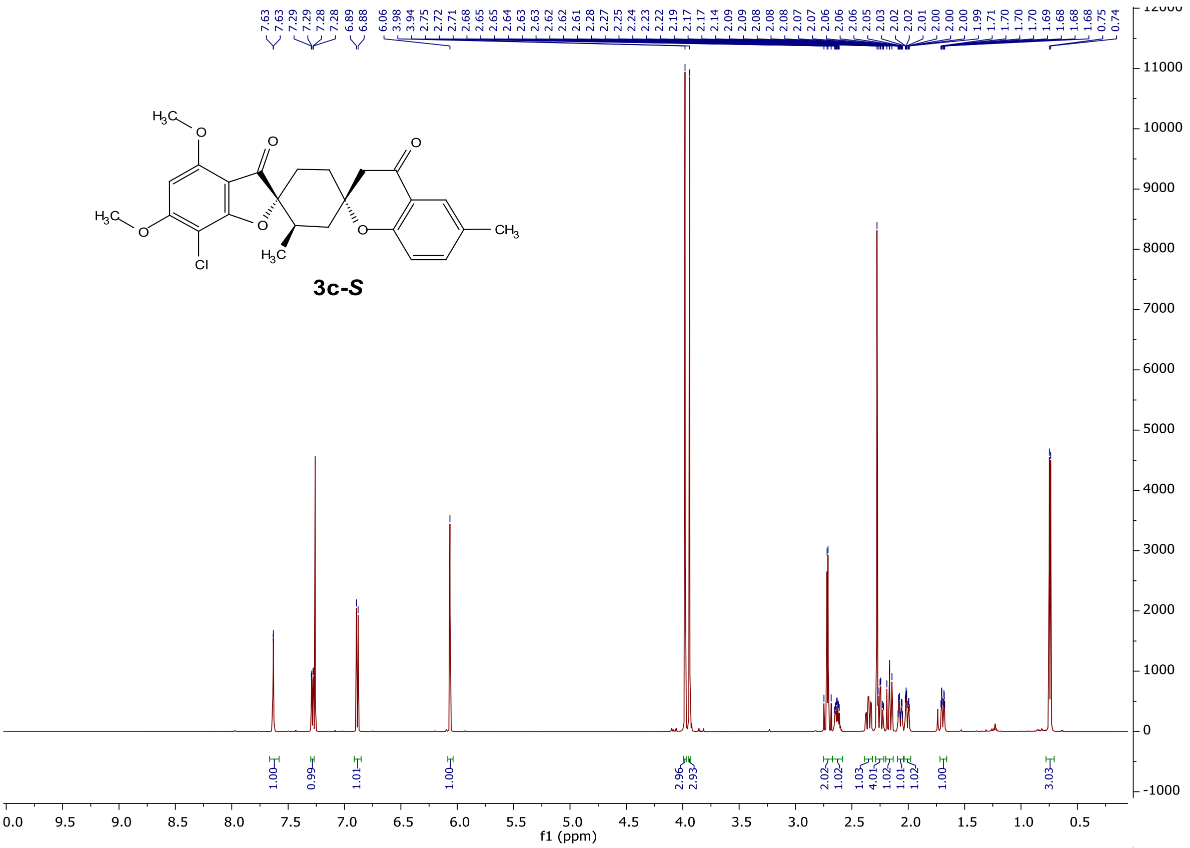
7.63
7.63
7.29
7.29
7.28
7.28
6.89
6.88
6.06
3.98
3.94
0.75
0.74
1.00
0.99
1.01
1.00
2.96
2.93
2.02
1.02
1.03
4.01
1.02
1.01
1.02
1.00
3.03
3c-S
f1 (ppm)

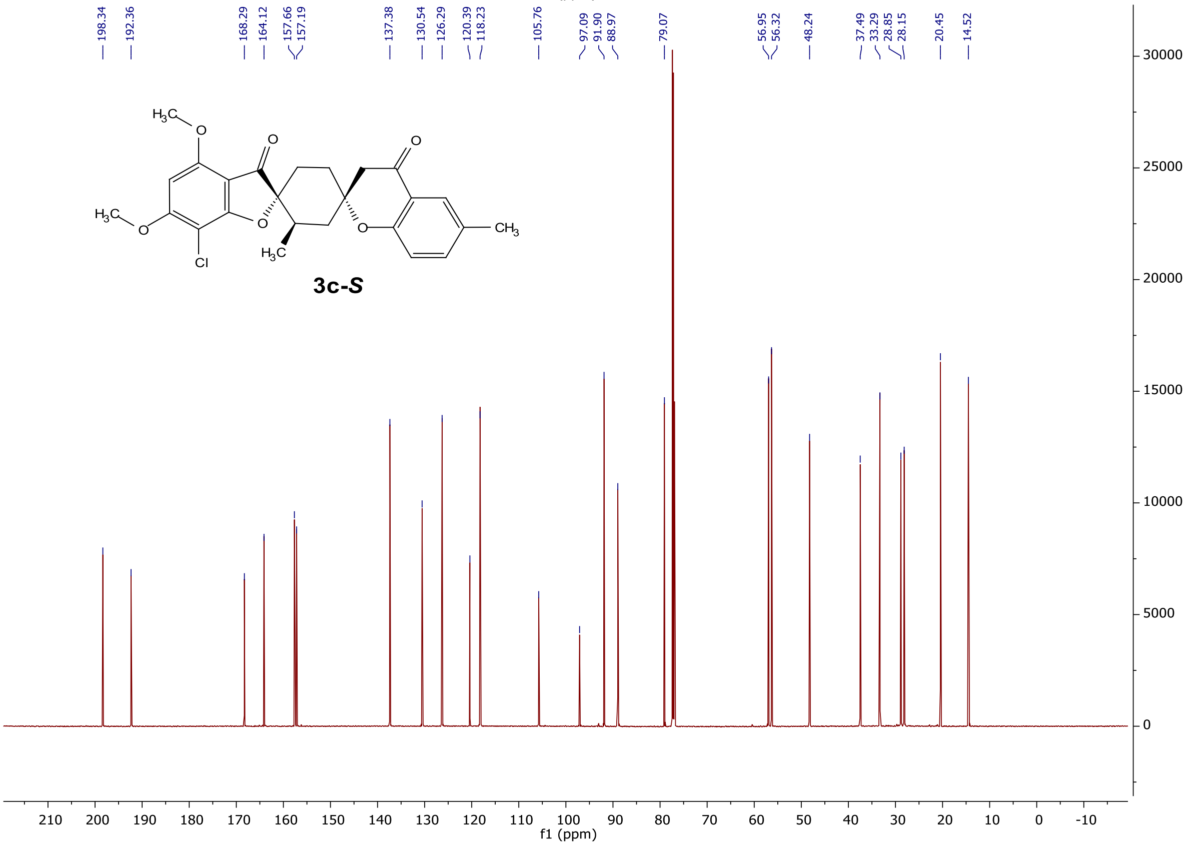
198.34
192.36
168.29
164.12
157.66
157.19
137.38
130.54
126.29
120.39
118.23
105.76
97.09
91.90
88.97
79.07
56.95
56.32
48.24
37.49
33.29
28.85
28.15
20.45
14.52
3c-S
f1 (ppm)

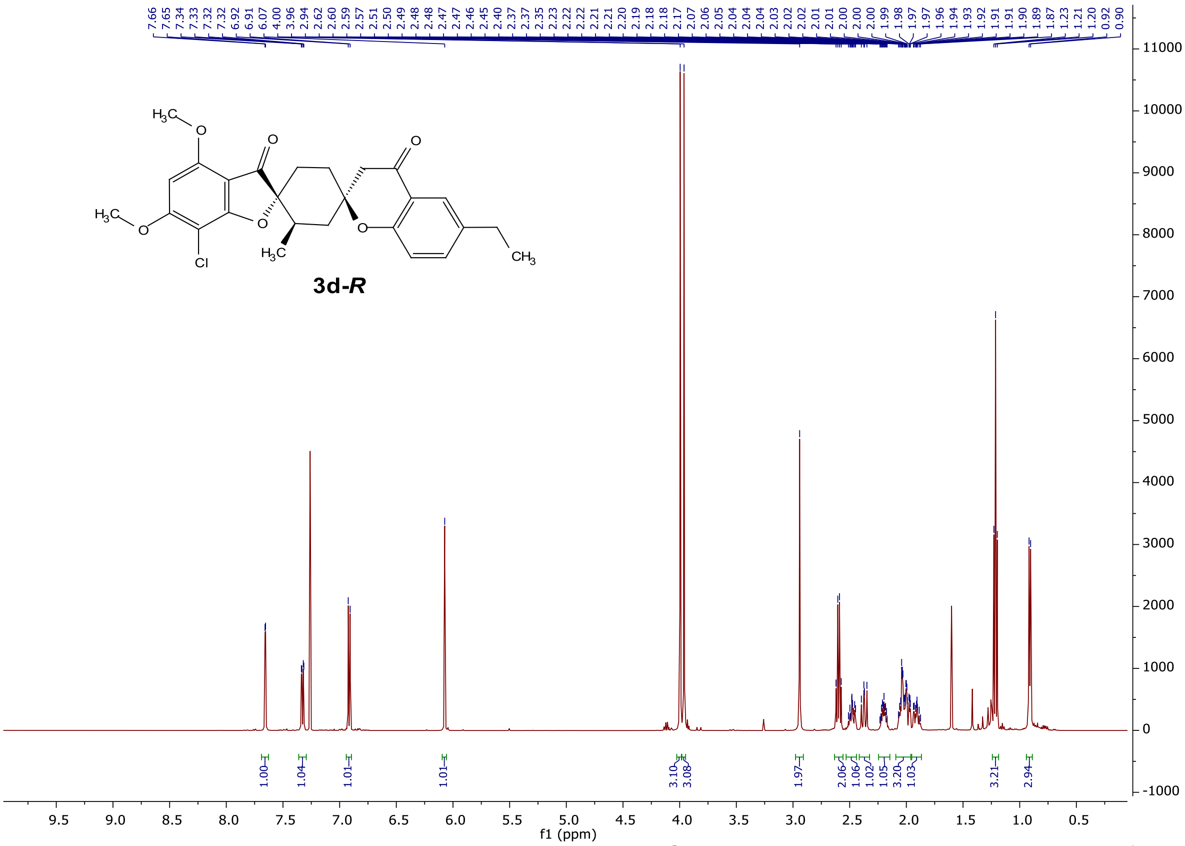
7.66 7.65 7.34 7.33 7.32 7.32 6.92 6.91 6.07 4.00 3.96 2.94 2.62 2.60 2.59 2.57 2.51 2.50 2.49 2.48 2.48 2.47 2.47 2.46 2.45 2.40 2.37 2.37 2.35 2.23 2.22 2.22 2.21 2.21 2.20 2.19 2.18 2.18 2.17 2.07 2.06 2.05 2.04 2.04 2.04 2.03 2.02 2.02 2.01 2.01 2.00 2.00 2.00 1.99 1.98 1.97 1.97 1.96 1.94 1.93 1.92 1.91 1.91 1.90 1.89 1.87 1.23 1.21 1.20 0.92 0.90
H3C O O O H3C O O Cl H3C O CH3
3d-R
1.00 1.04 1.01 1.01 3.10 3.08 1.97 2.06 1.06 1.02 1.05 3.20 1.03 3.21 2.94
f1 (ppm)

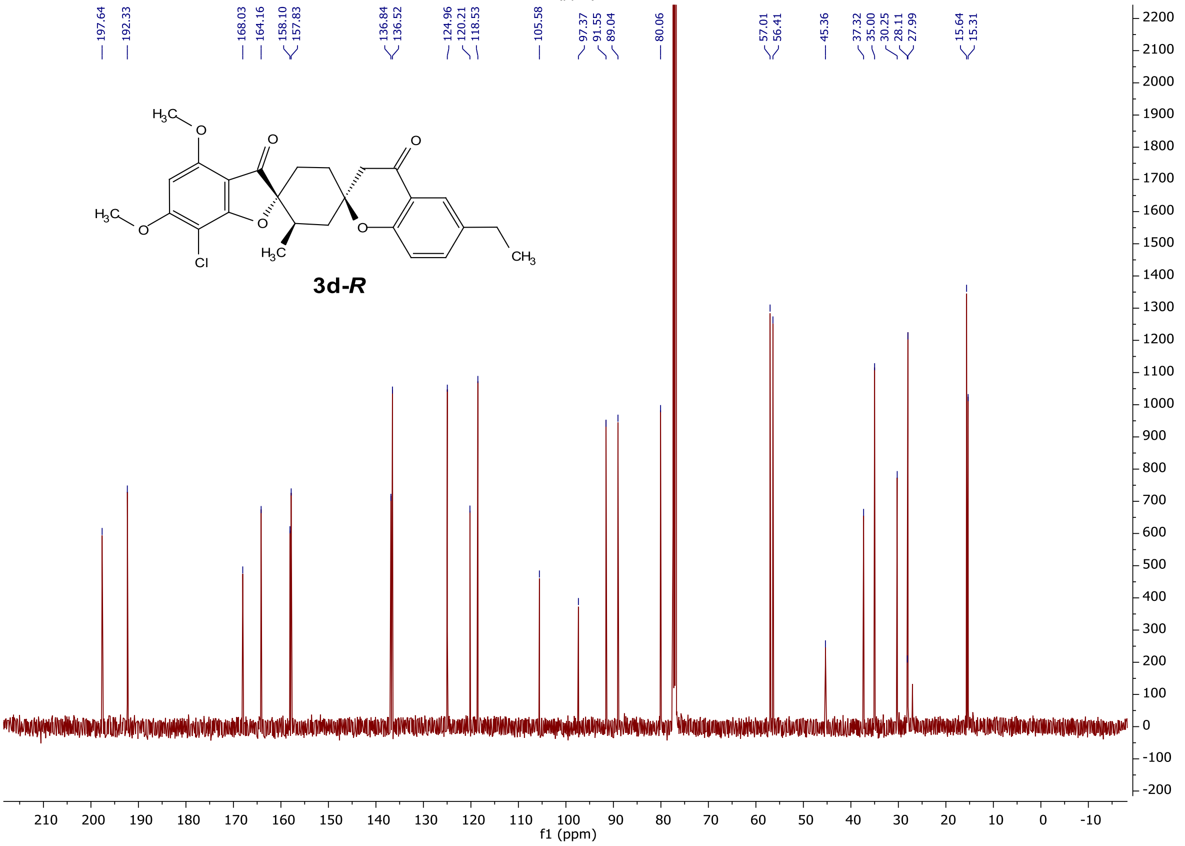
197.64 192.33 168.03 164.16 158.10 157.83 136.84 136.52 124.96 120.21 118.53 105.58 97.37 91.55 89.04 80.06 57.01 56.41 45.36 37.32 35.00 30.25 28.11 27.99 15.64 15.31
H3C O O O H3C O O Cl H3C O CH3
3d-R
f1 (ppm)

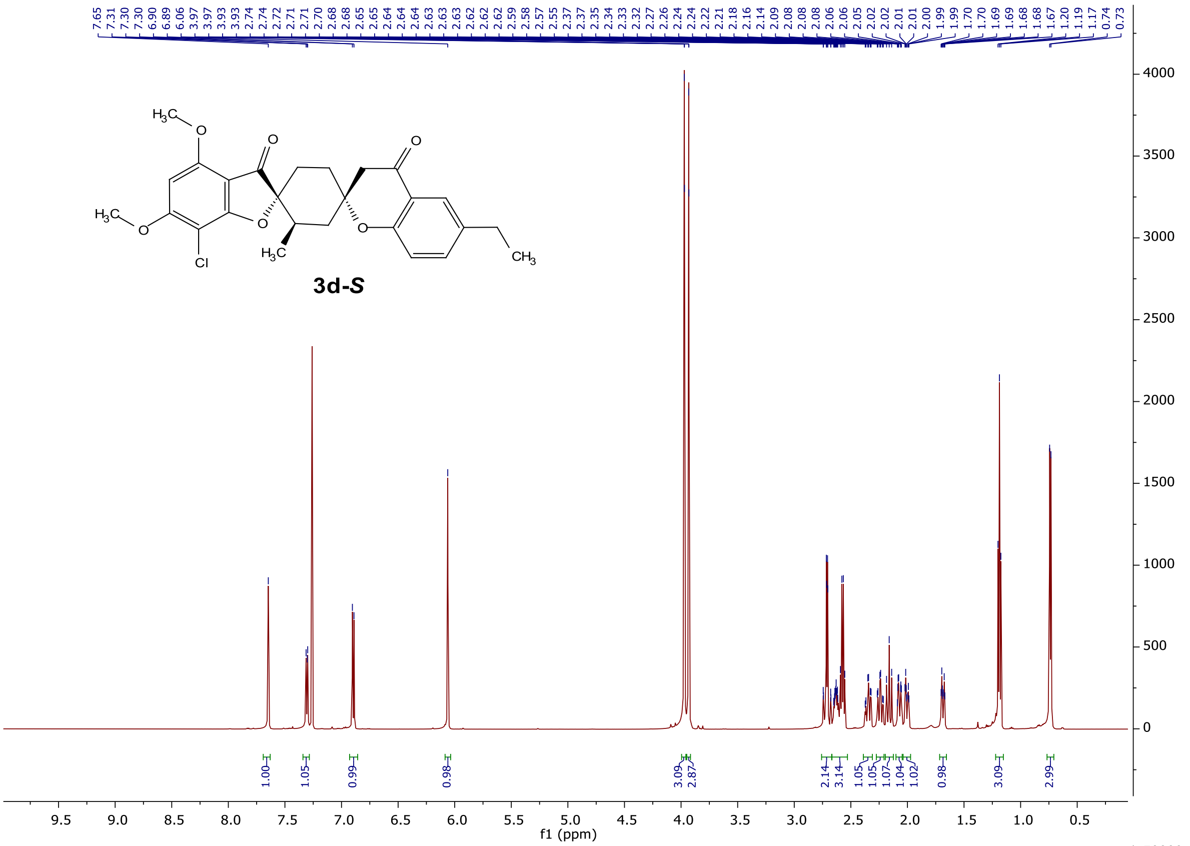
7.65 7.31 7.30 7.30 6.90 6.89 6.06 3.97 3.97 3.93 3.93 2.74 2.72 2.71 2.71 2.70 2.68 2.68 2.65 2.65 2.64 2.64 2.63 2.63 2.63 2.62 2.62 2.62 2.59 2.58 2.57 2.55 2.37 2.37 2.35 2.34 2.33 2.32 2.27 2.26 2.24 2.24 2.22 2.21 2.18 2.16 2.14 2.09 2.08 2.08 2.08 2.06 2.06 2.05 2.02 2.02 2.01 2.01 2.00 1.99 1.99 1.70 1.70 1.69 1.69 1.68 1.68 1.67 1.20 1.19 1.17 0.74 0.73
H3C
O
O
O
H3C
O
O
Cl
H3C
O
CH3
3d-S
1.00 1.05 0.99 0.98 3.09 2.87 2.14 3.14 1.05 1.05 1.07 1.04 1.02 0.98 3.09 2.99
f1 (ppm)

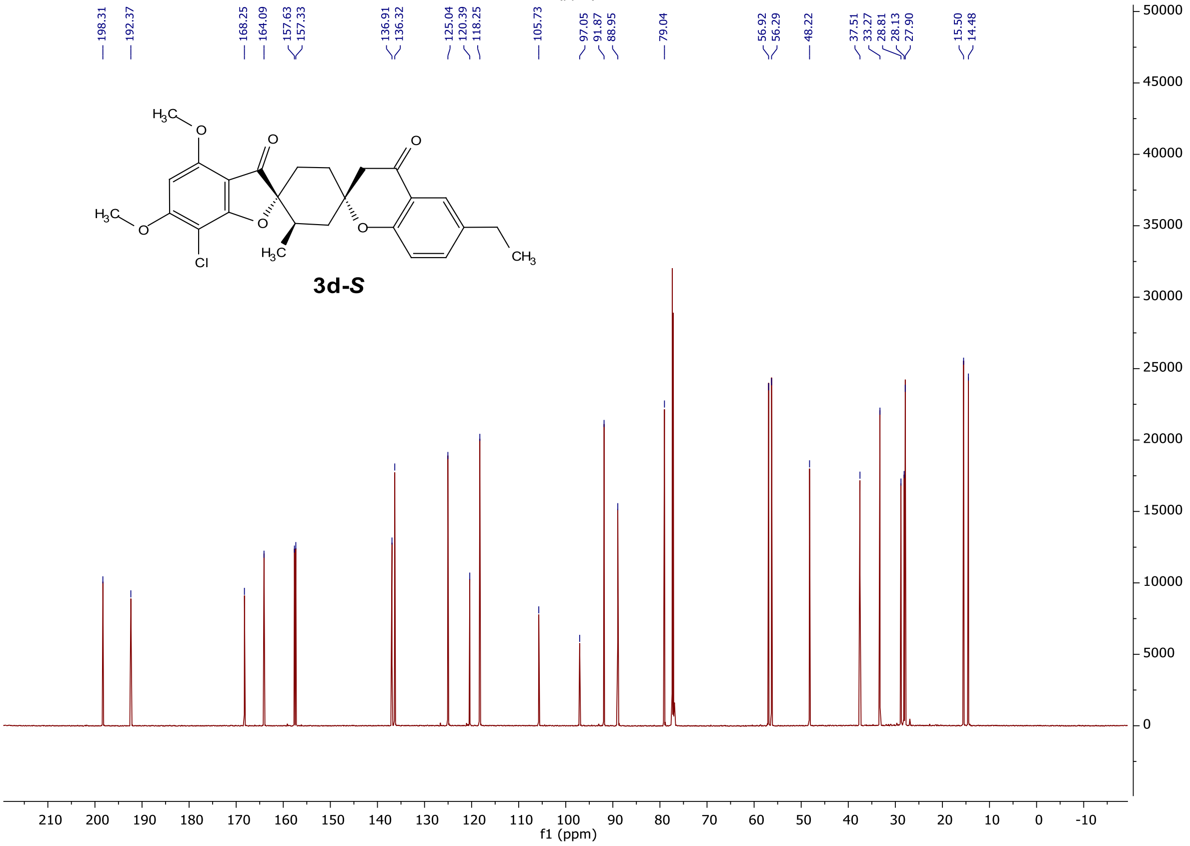
198.31 192.37 168.25 164.09 157.63 157.33 136.91 136.32 125.04 120.39 118.25 105.73 97.05 91.87 88.95 79.04 56.92 56.29 48.22 37.51 33.27 28.81 28.13 27.90 15.50 14.48
3d-S
f1 (ppm)

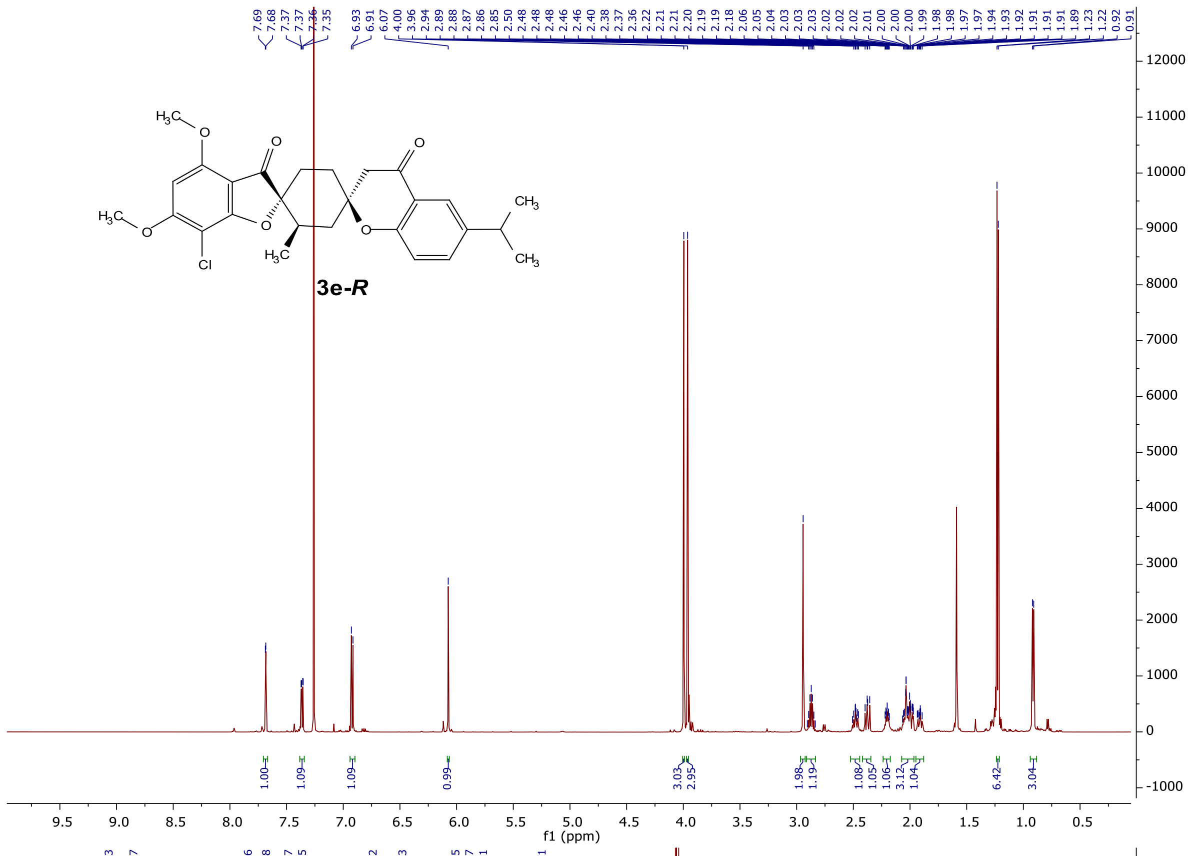

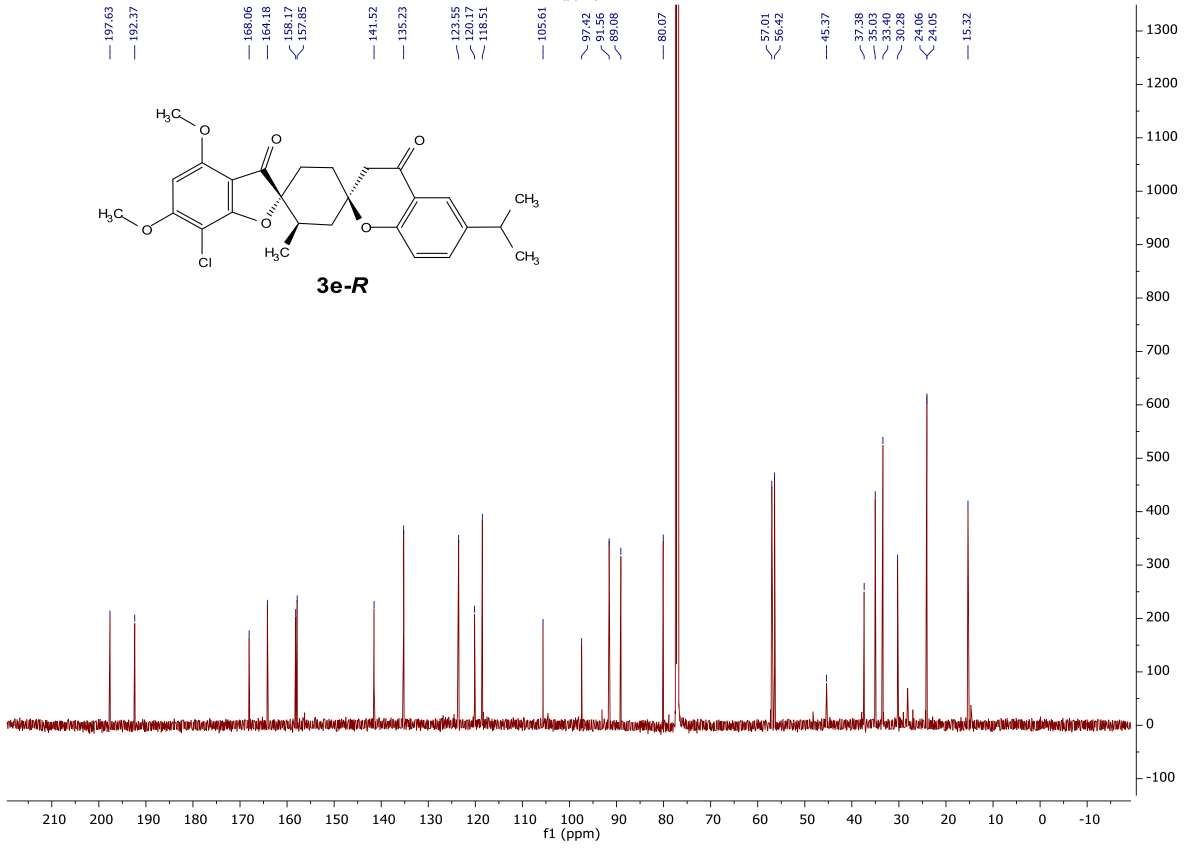

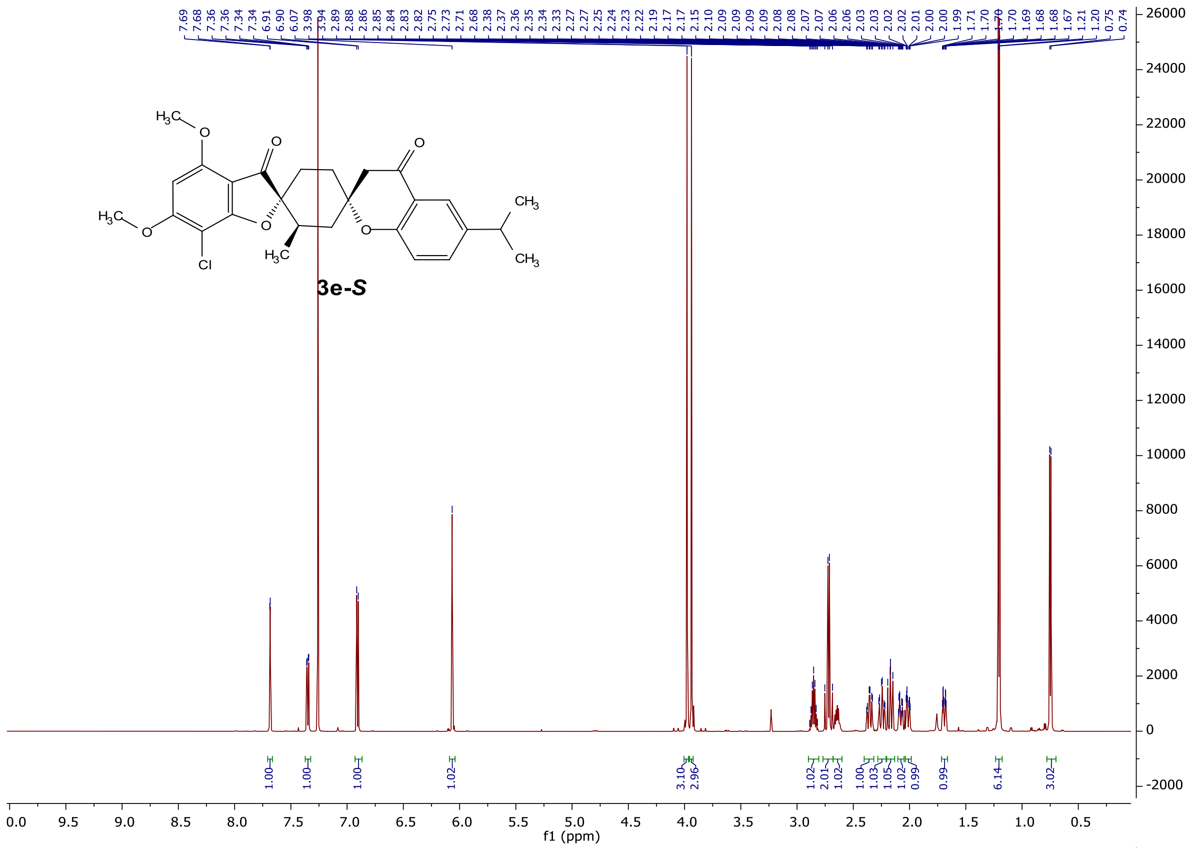
7.69 7.68 7.36 7.36 7.34 7.34 6.91 6.90 6.07 3.98 3.94 2.89 2.88 2.86 2.85 2.84 2.83 2.82 2.75 2.73 2.71 2.68 2.38 2.37 2.36 2.35 2.34 2.33 2.27 2.27 2.25 2.24 2.23 2.22 2.19 2.17 2.17 2.15 2.10 2.09 2.09 2.09 2.09 2.08 2.08 2.07 2.07 2.06 2.03 2.03 2.02 2.02 2.01 2.00 2.00 1.99 1.71 1.70 1.70 1.70 1.69 1.68 1.68 1.67 1.21 1.20 0.75 0.74
1.00 1.00 1.00 1.02 3.10 2.96 1.02 2.01 1.02 1.00 1.03 1.05 1.02 0.99 0.99 6.14 3.02
3e-S
f1 (ppm)

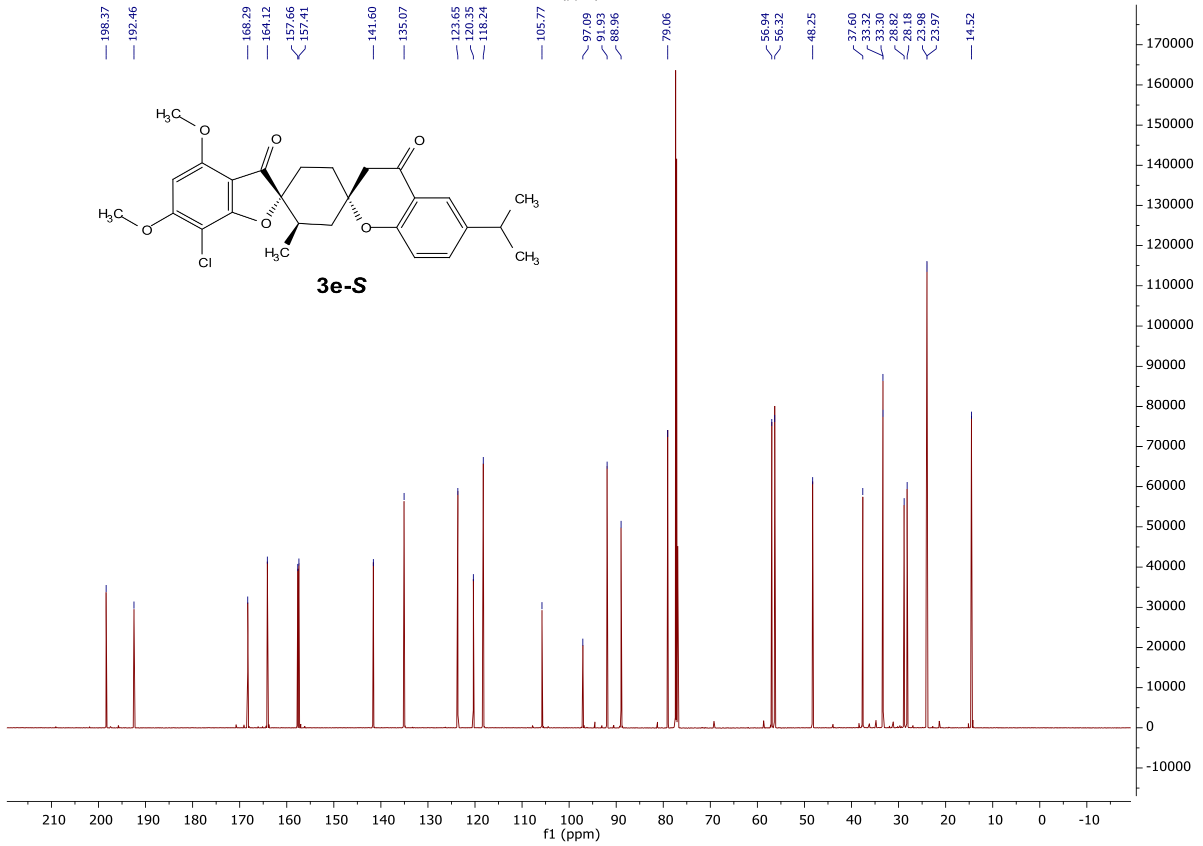
198.37 192.46 168.29 164.12 157.66 157.41 141.60 135.07 123.65 120.35 118.24 105.77 97.09 91.93 88.96 79.06 56.94 56.32 48.25 37.60 33.32 33.30 28.82 28.18 23.98 23.97 14.52
3e-S
f1 (ppm)

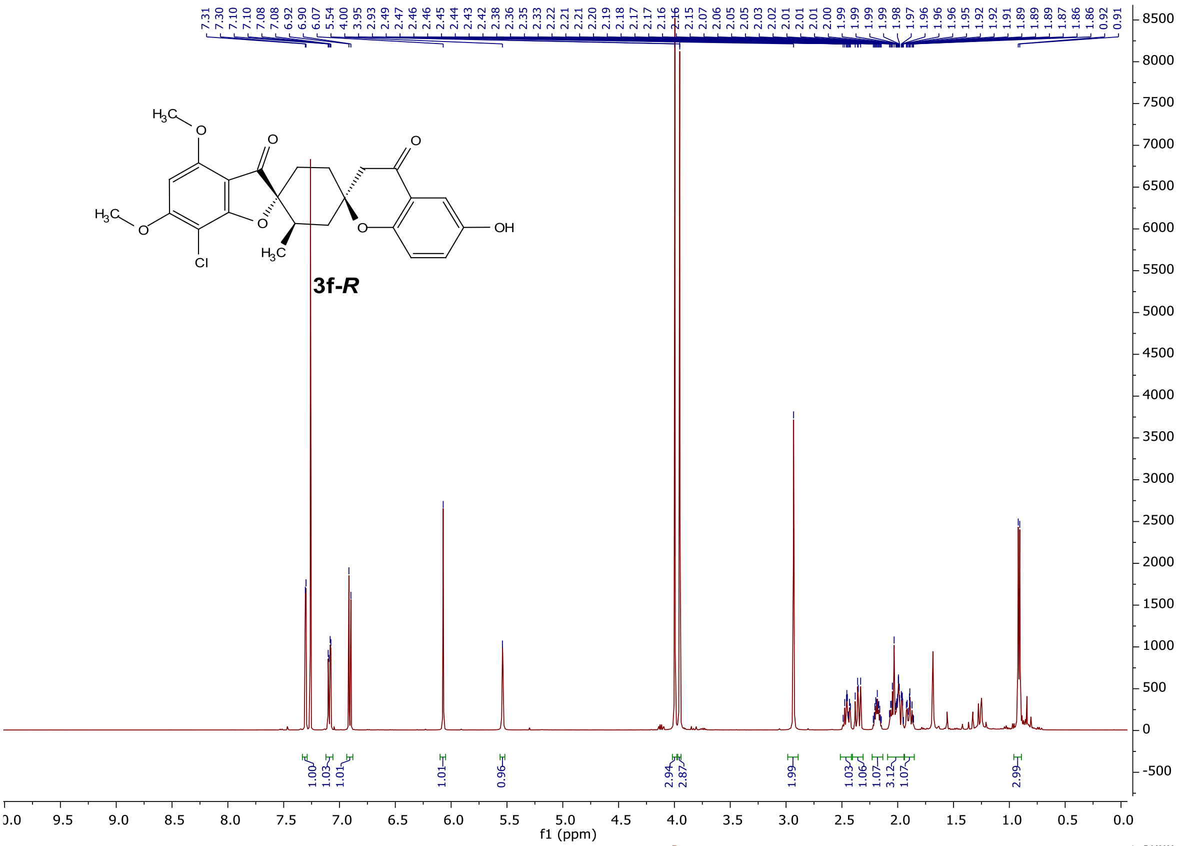
7.31 7.30 7.10 7.08 7.08 6.92 6.90 6.07 5.54 4.00 3.95 2.93 2.49 2.47 2.46 2.46 2.45 2.44 2.43 2.42 2.38 2.36 2.35 2.33 2.22 2.21 2.21 2.20 2.19 2.18 2.17 2.17 2.16 2.16 2.15 2.07 2.06 2.05 2.05 2.03 2.02 2.01 2.01 2.01 2.00 1.99 1.99 1.99 1.99 1.98 1.97 1.96 1.96 1.96 1.95 1.92 1.92 1.91 1.89 1.89 1.89 1.87 1.86 0.92 0.91
1.00 1.03 1.01 1.01 0.96 2.94 2.87 1.99 1.03 1.06 1.07 3.12 1.07 2.99
3f-R
f1 (ppm)

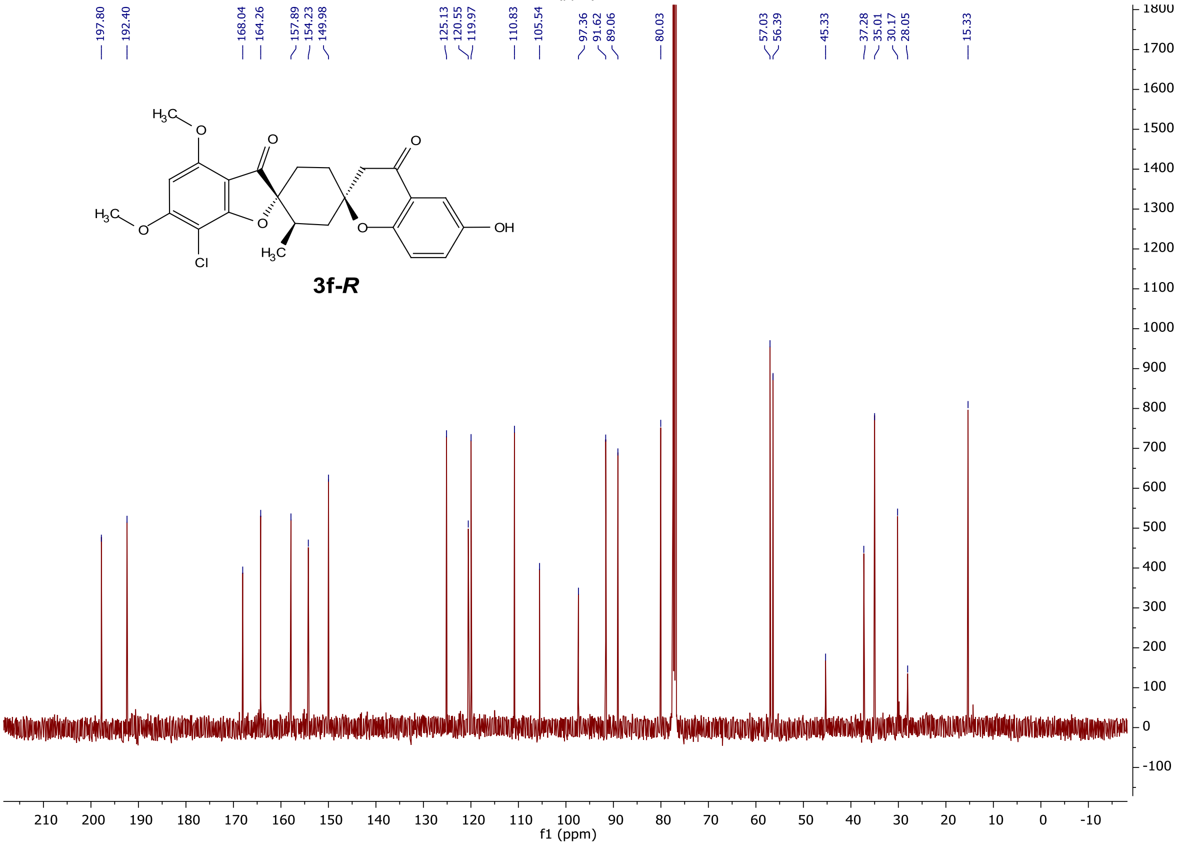
197.80 192.40 168.04 164.26 157.89 154.23 149.98 125.13 120.55 119.97 110.83 105.54 97.36 91.62 89.06 80.03 57.03 56.39 45.33 37.28 35.01 30.17 28.05 15.33
3f-R
f1 (ppm)

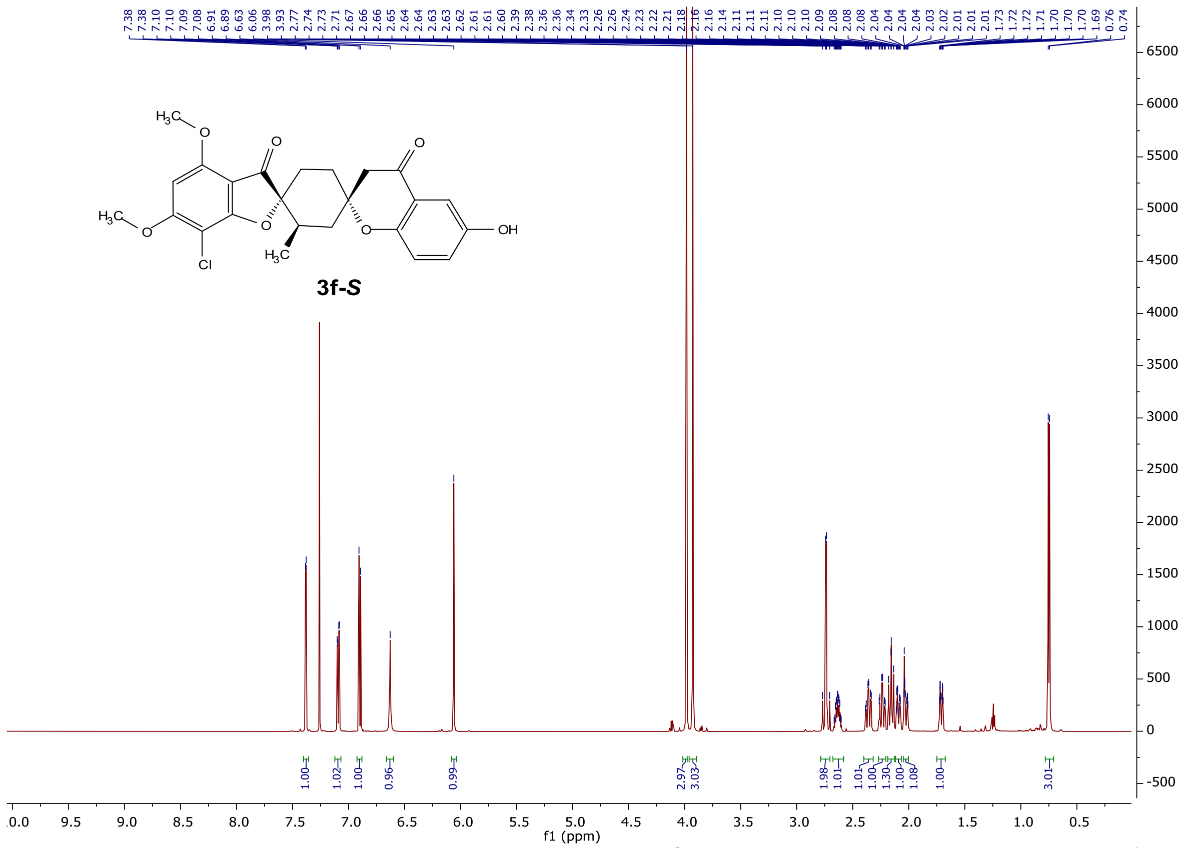
3f-*S*
f1 (ppm)

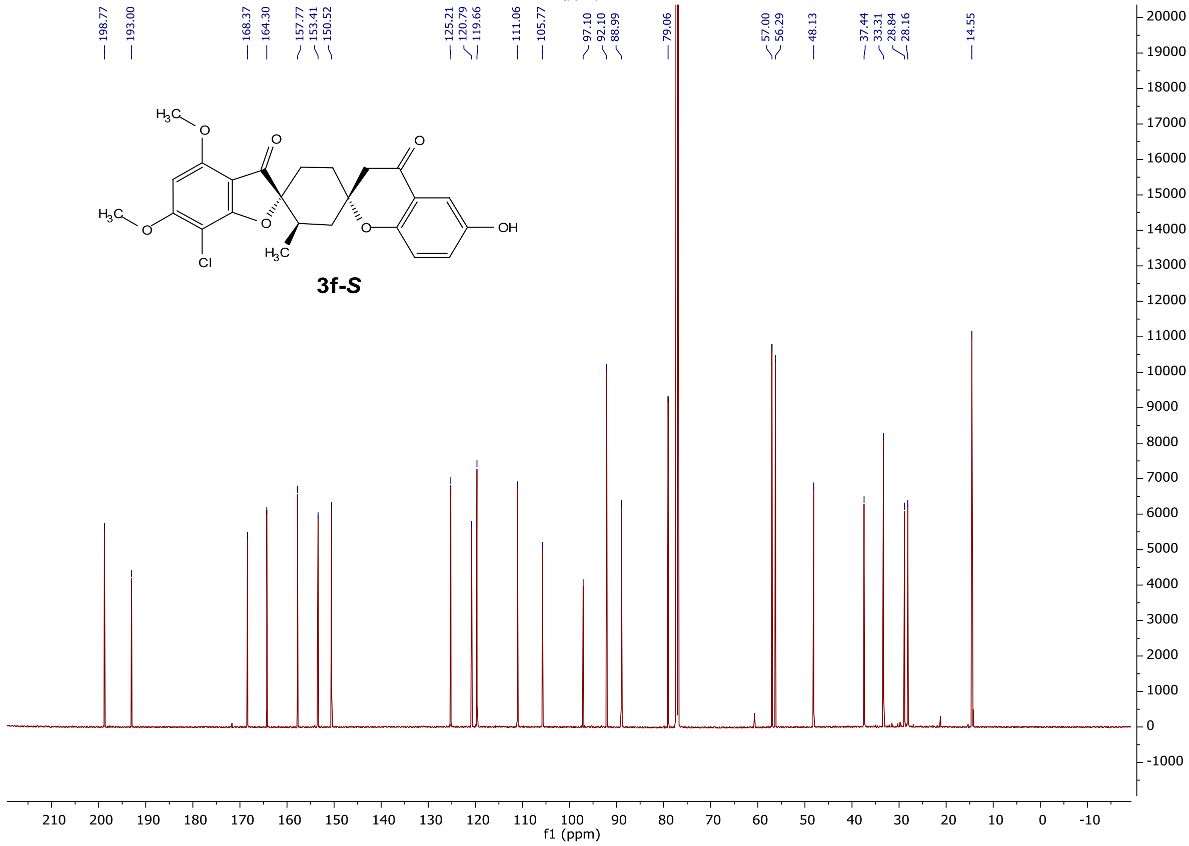
3f-*S*
f1 (ppm)

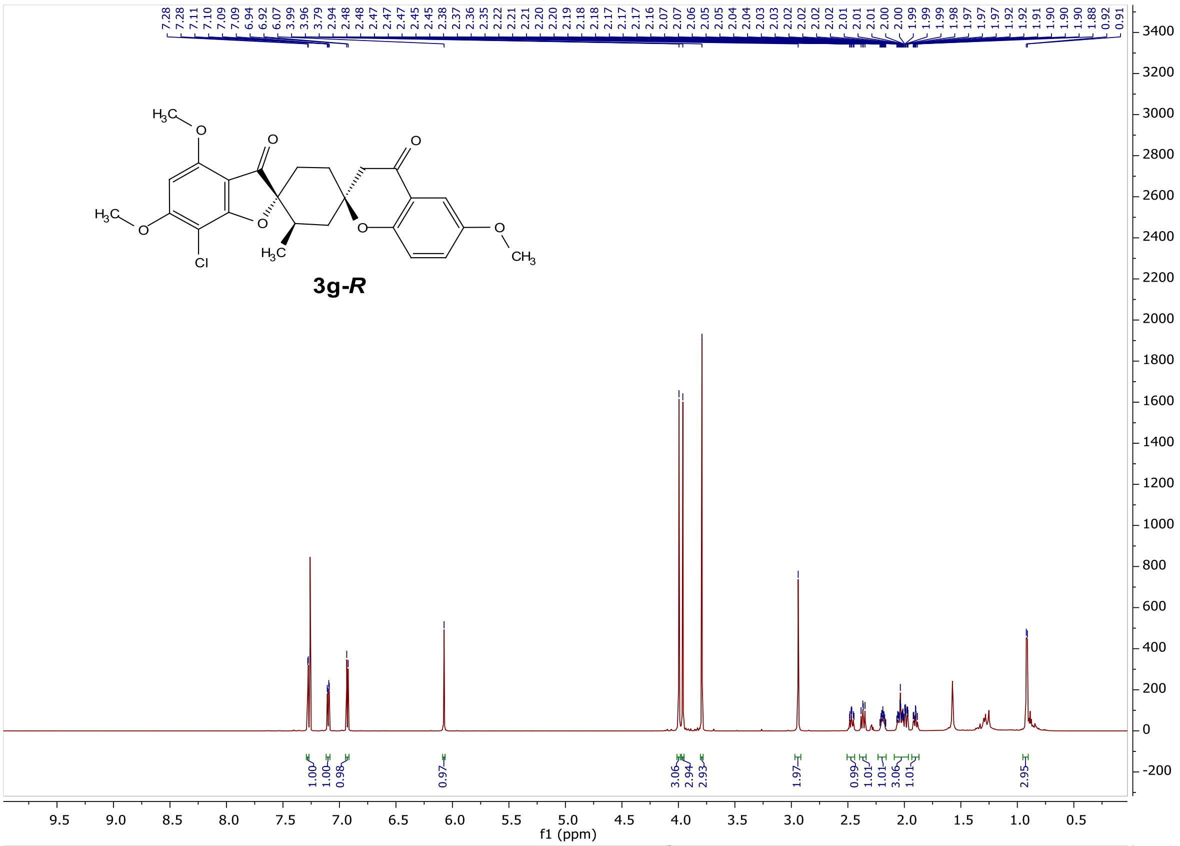
7.28 7.28 7.11 7.10 7.09 7.09 6.94 6.92 6.07 3.99 3.96 3.79 2.94 2.48 2.48 2.47 2.47 2.45 2.45 2.38 2.37 2.36 2.35 2.22 2.21 2.21 2.20 2.20 2.19 2.18 2.18 2.17 2.17 2.16 2.07 2.07 2.06 2.05 2.05 2.04 2.04 2.03 2.03 2.02 2.02 2.02 2.01 2.01 2.01 2.00 2.00 1.99 1.99 1.99 1.98 1.97 1.97 1.97 1.92 1.92 1.91 1.90 1.90 1.90 1.88 0.92 0.91
3g-*R*
1.00 1.00 0.98 0.97 3.06 2.94 2.93 1.97 0.99 1.01 1.01 3.06 1.01 2.95
f1 (ppm)

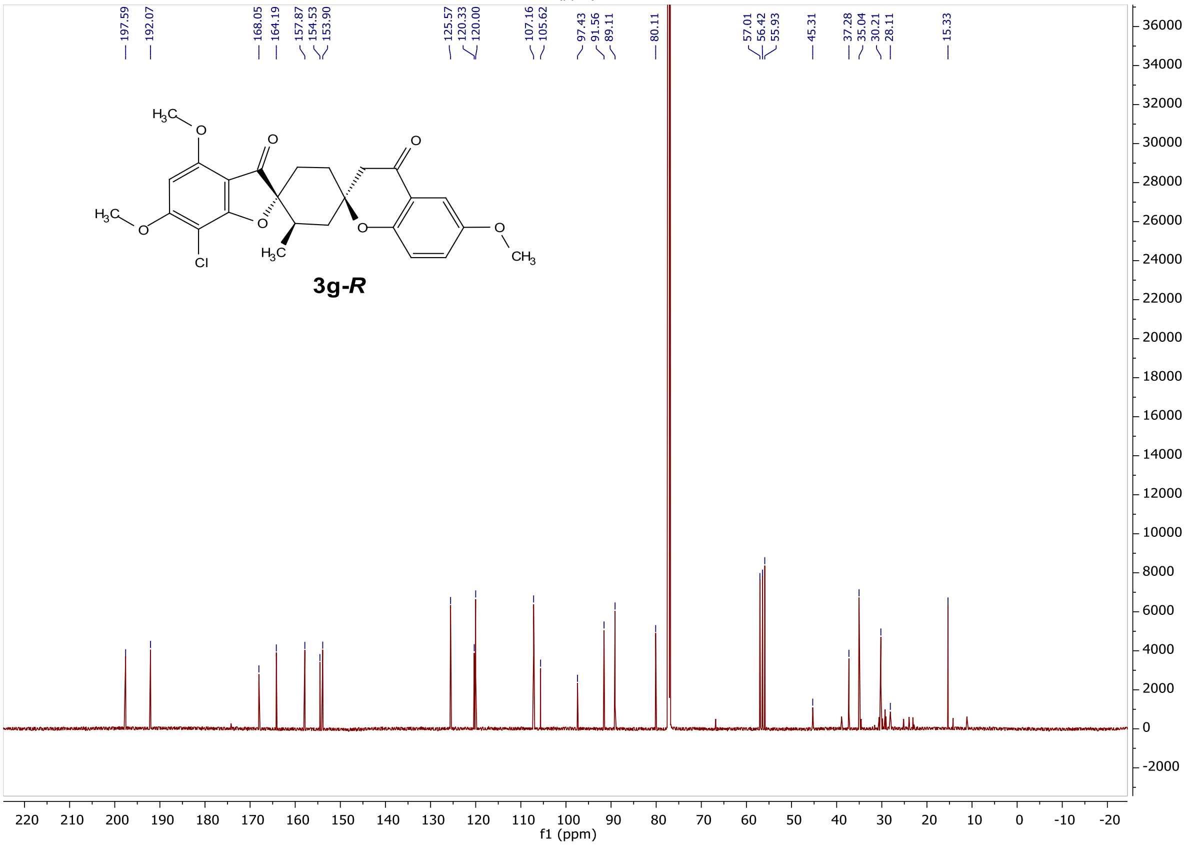
197.59 192.07 168.05 164.19 157.87 154.53 153.90 125.57 120.33 120.00 107.16 105.62 97.43 91.56 89.11 80.11 57.01 56.42 55.93 45.31 37.28 35.04 30.21 28.11 15.33
3g-*R*
f1 (ppm)

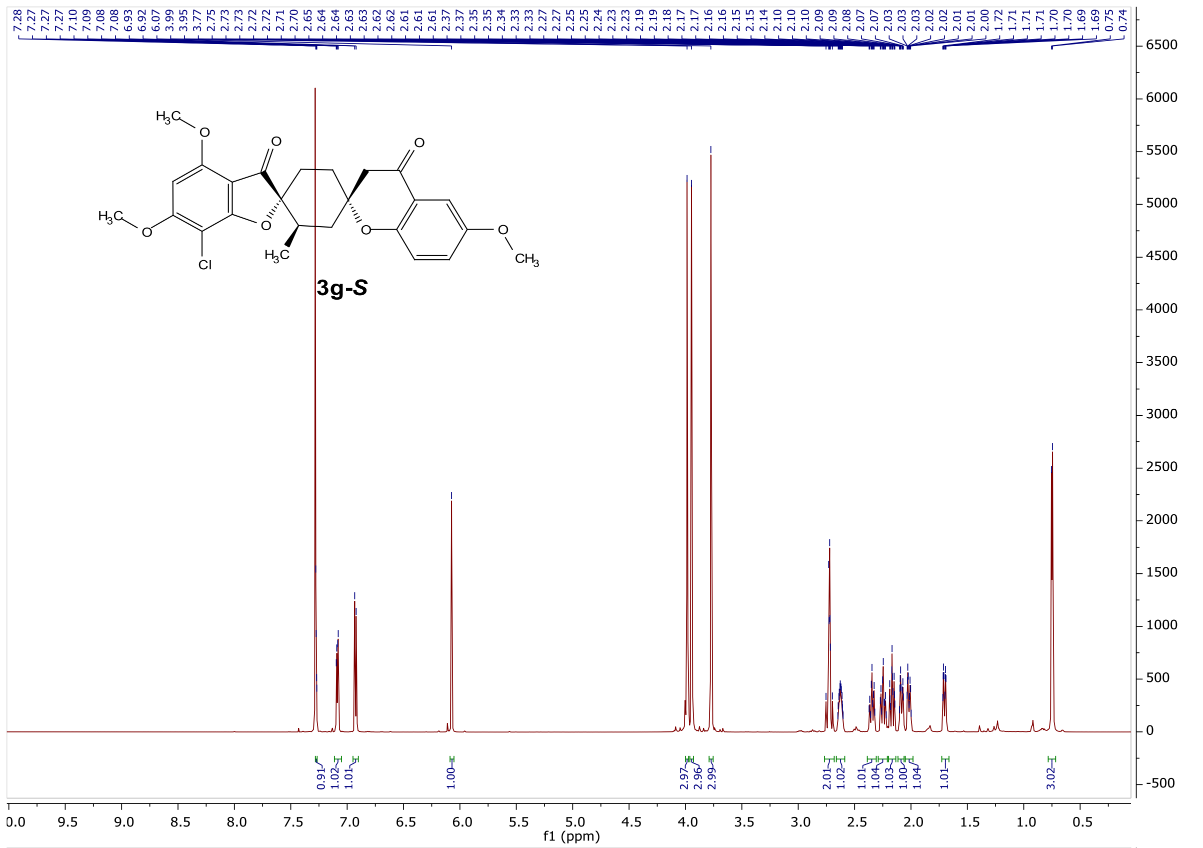

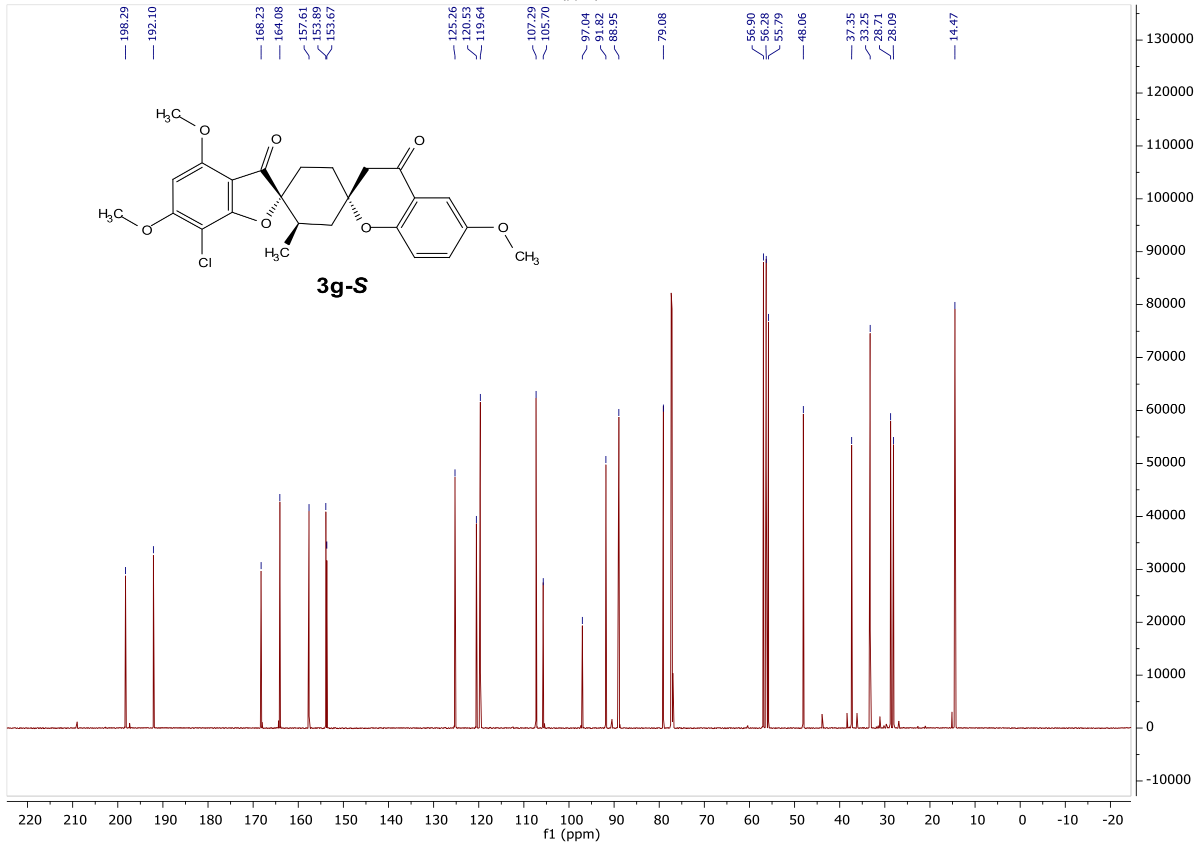

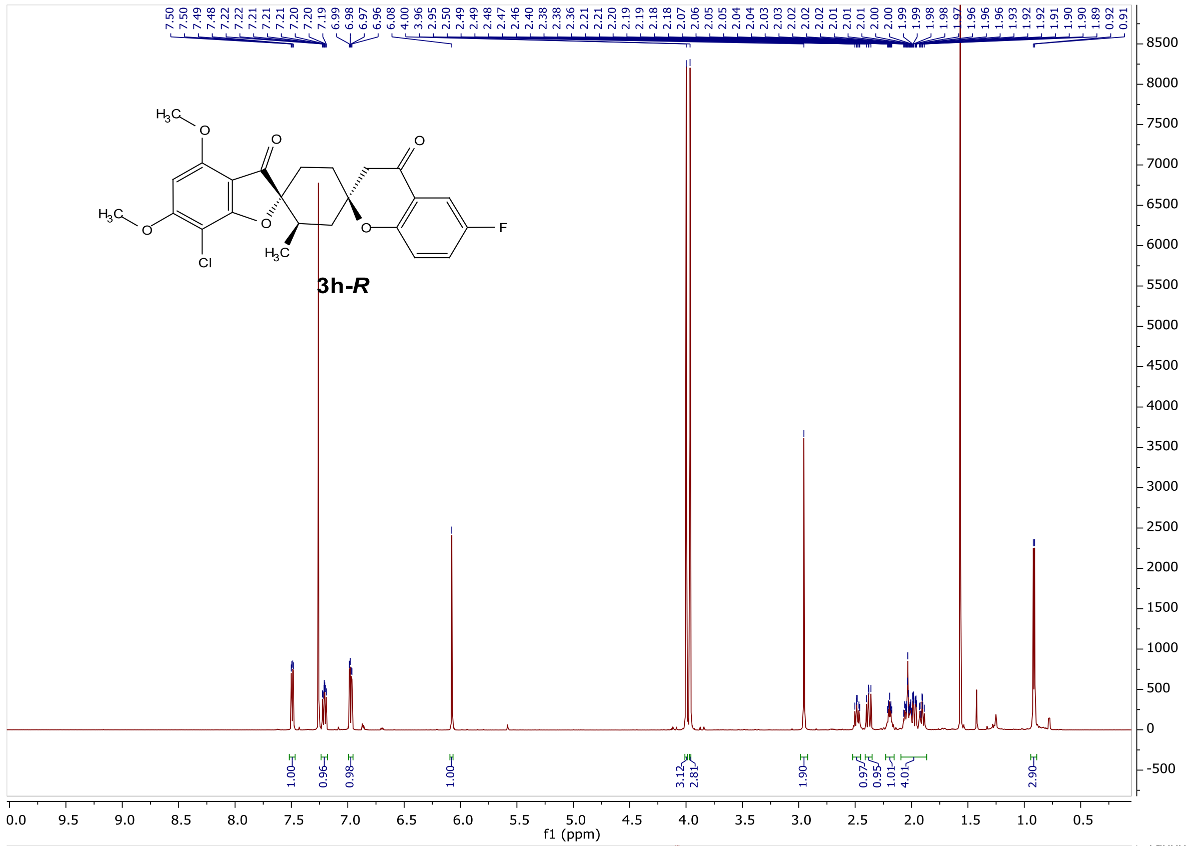

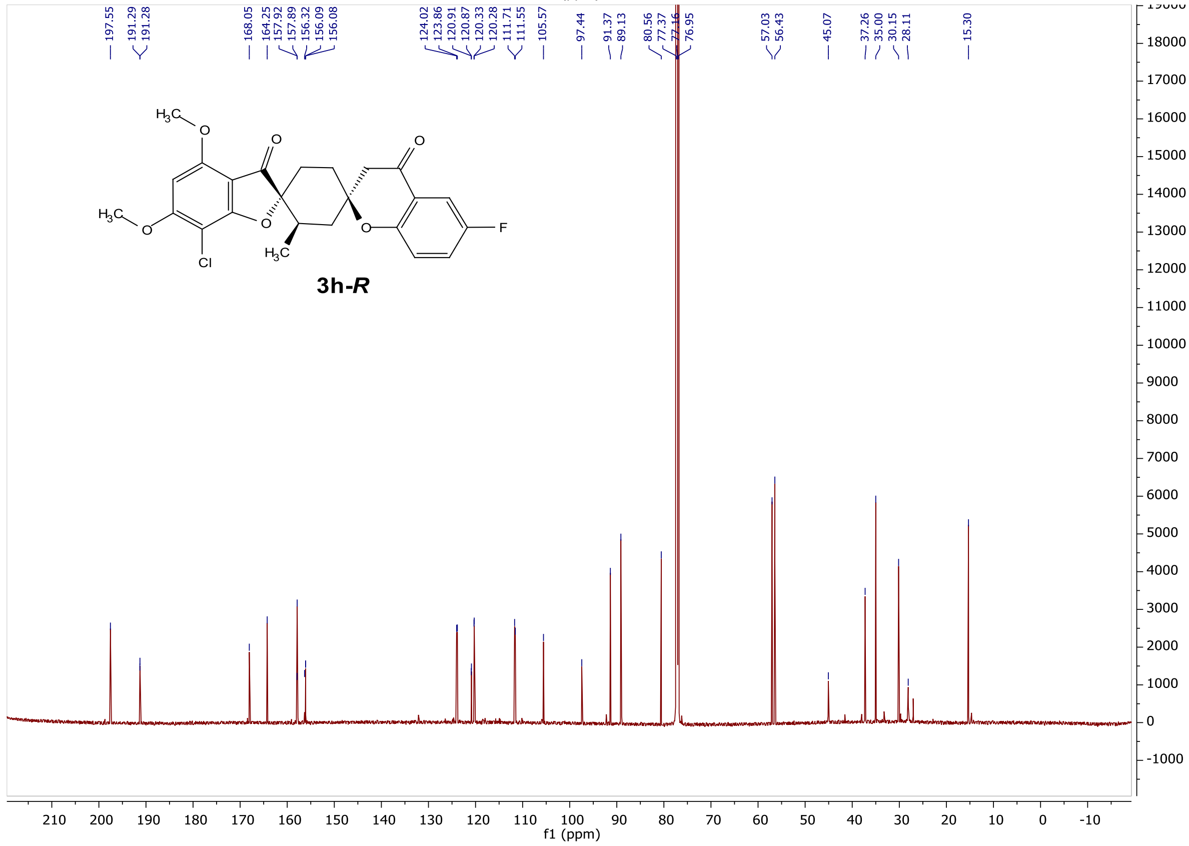

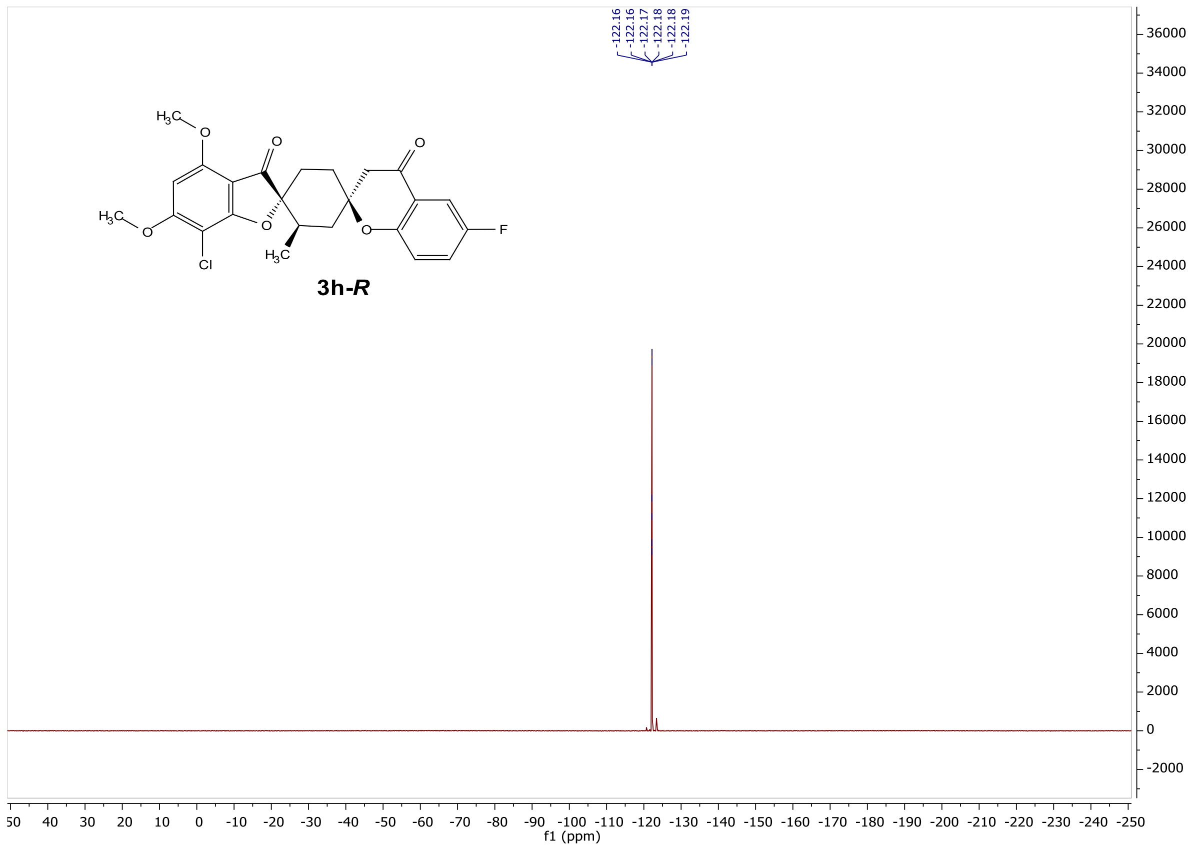
-122.16
-122.16
-122.17
-122.18
-122.18
-122.19
3h-R
36000
34000
32000
30000
28000
26000
24000
22000
20000
18000
16000
14000
12000
10000
8000
6000
4000
2000
0
-2000
50 40 30 20 10 0 -10 -20 -30 -40 -50 -60 -70 -80 -90 -100 -110 -120 -130 -140 -150 -160 -170 -180 -190 -200 -210 -220 -230 -240 -250
f1 (ppm)

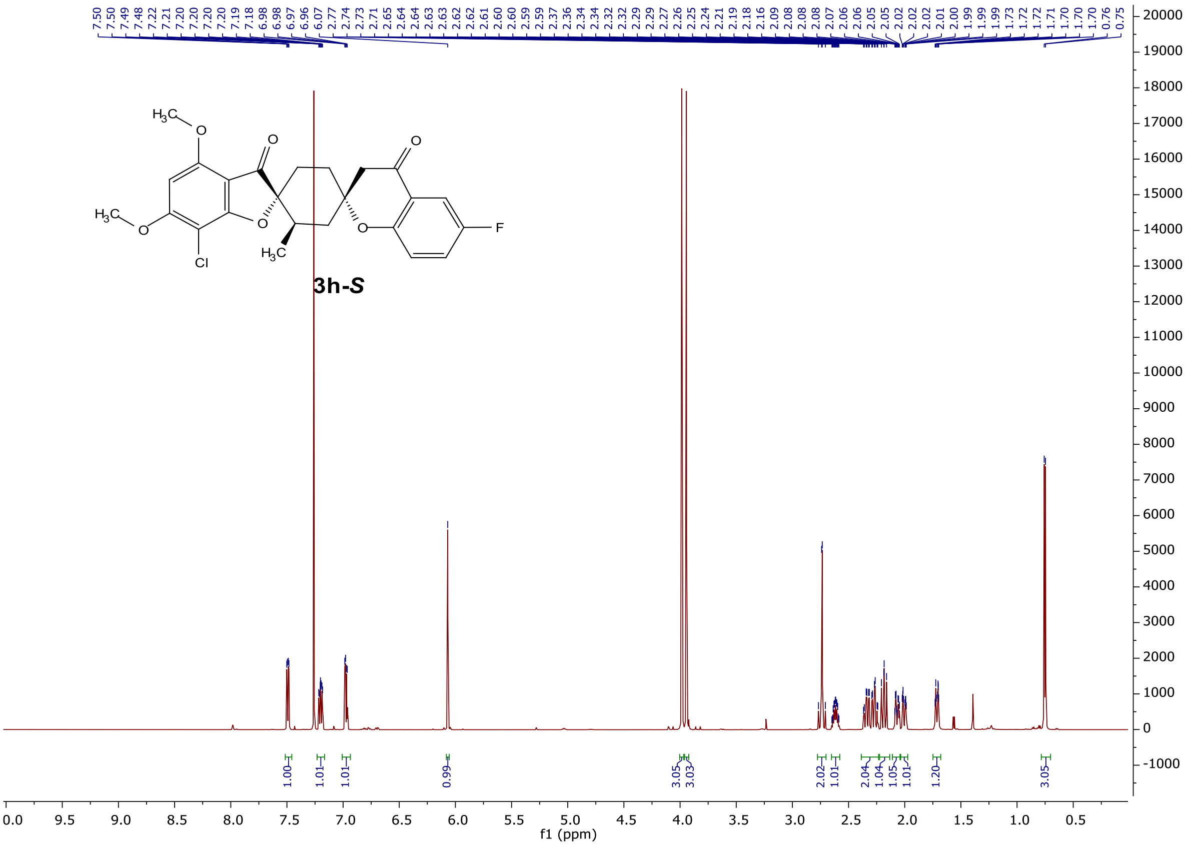
3h-S
f1 (ppm)

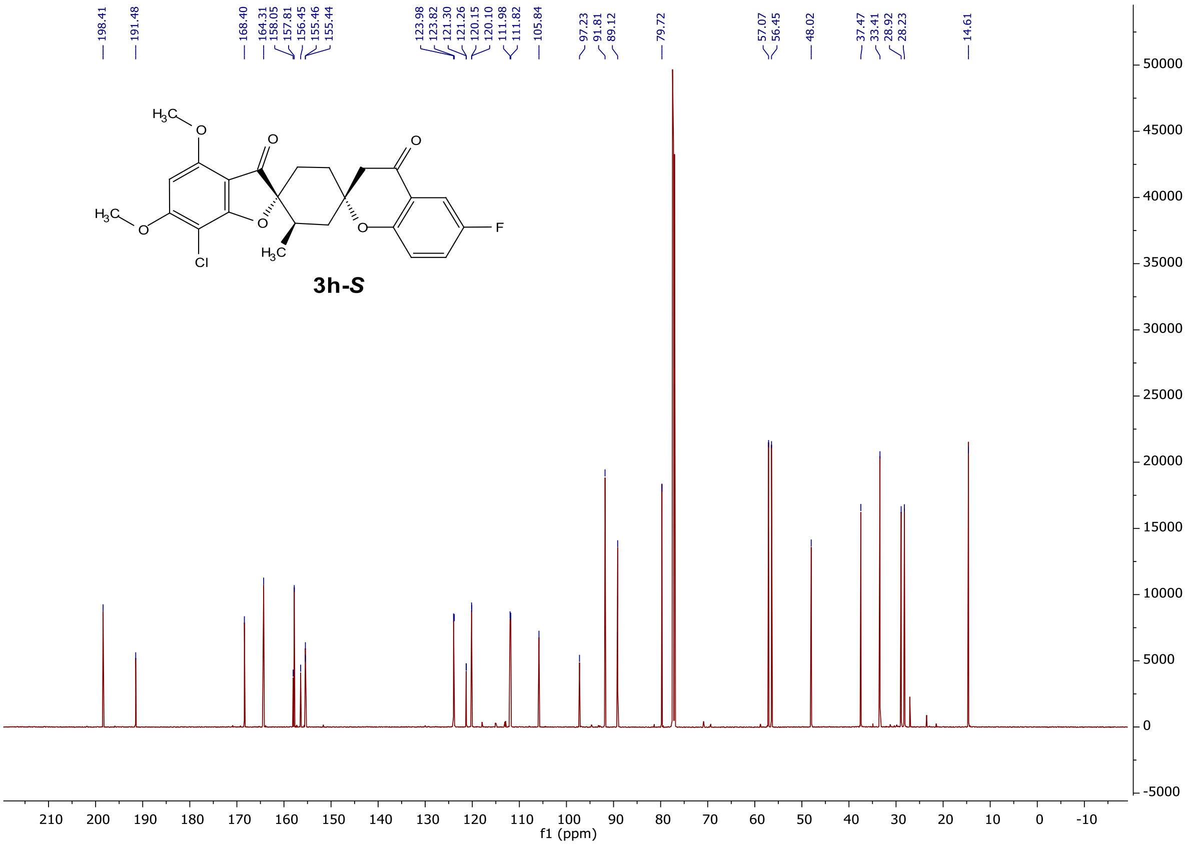
198.41
191.48
168.40
164.31
158.05
157.81
156.45
155.46
155.44
123.98
123.82
121.30
121.26
120.15
120.10
111.98
111.82
105.84
97.23
91.81
89.12
79.72
57.07
56.45
48.02
37.47
33.41
28.92
28.23
14.61
3h-S
f1 (ppm)

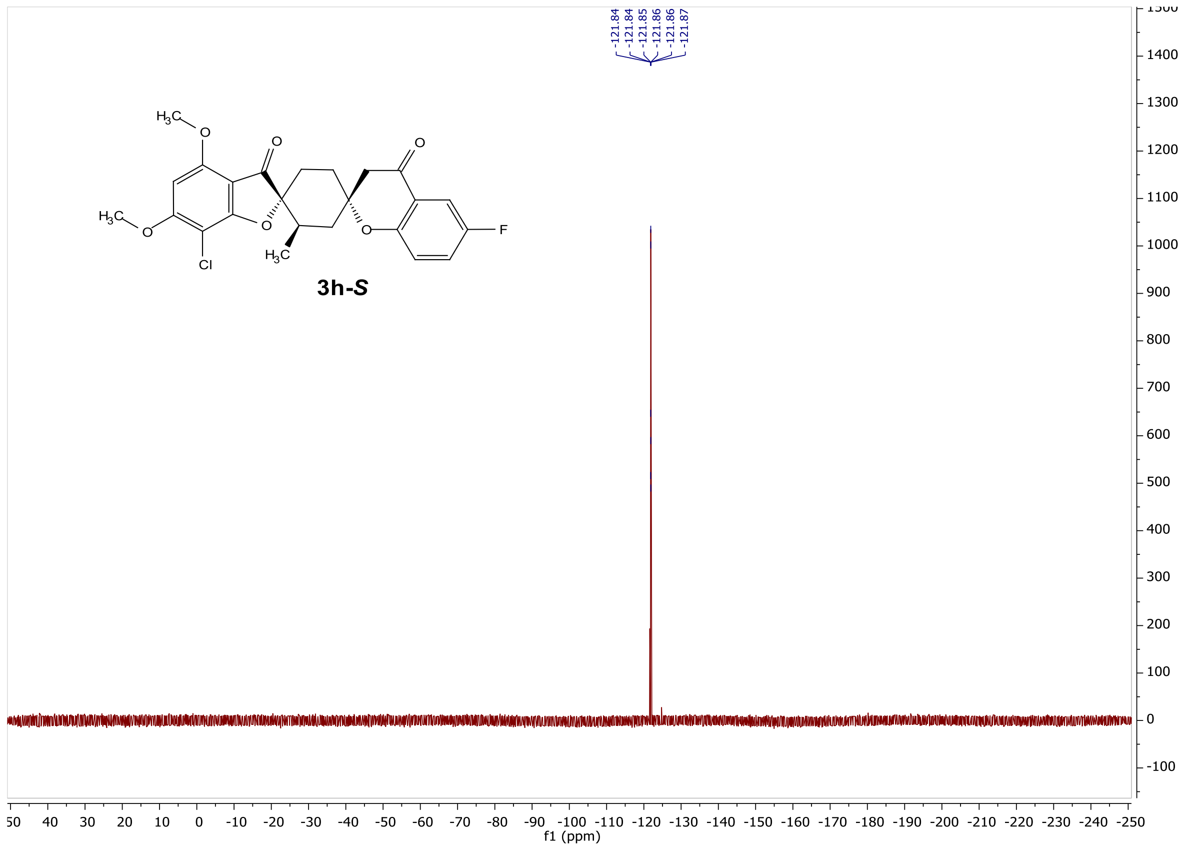
-121.84
-121.84
-121.85
-121.86
-121.86
-121.87
3h-S
f1 (ppm)

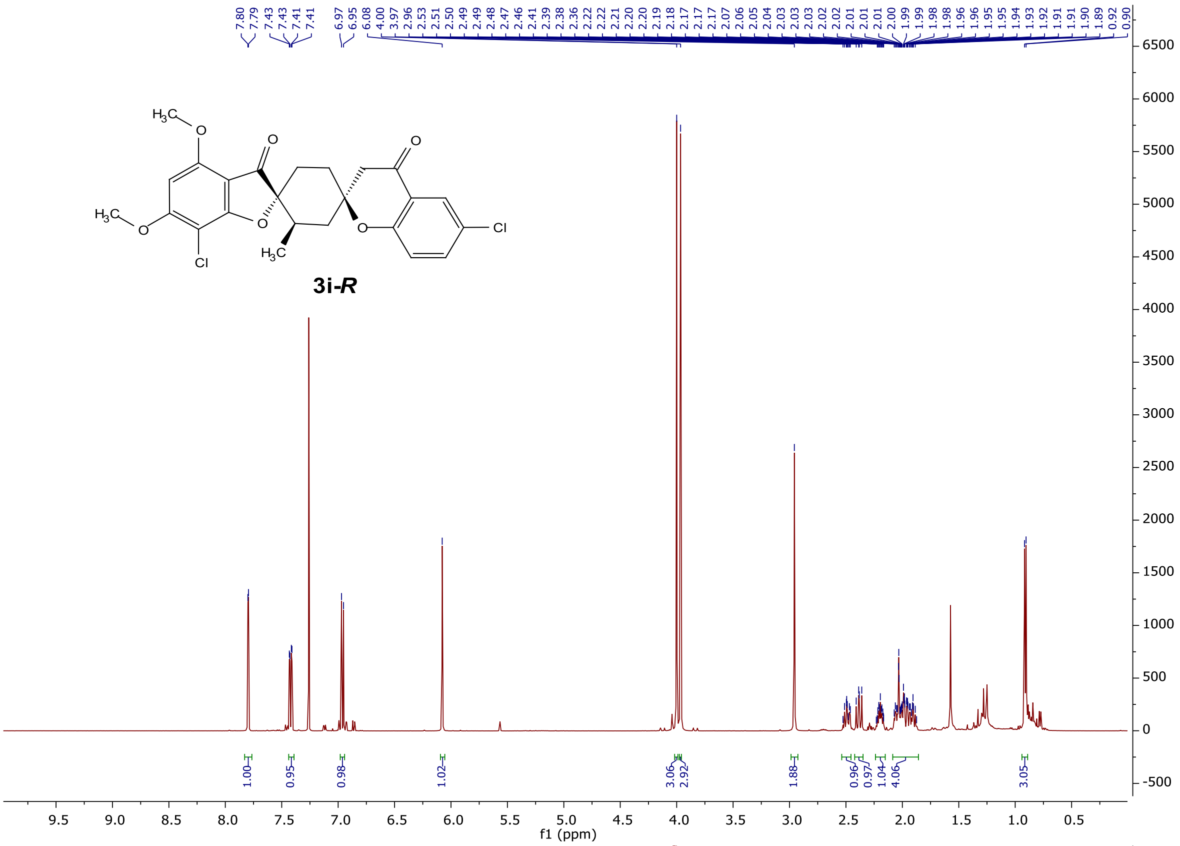
7.80 7.79 7.43 7.43 7.41 7.41 6.97 6.95 6.08 4.00 3.97 2.96 2.53 2.51 2.50 2.49 2.49 2.48 2.47 2.46 2.41 2.39 2.38 2.36 2.22 2.22 2.21 2.20 2.20 2.19 2.18 2.17 2.17 2.17 2.07 2.06 2.05 2.04 2.03 2.03 2.02 2.02 2.01 2.01 2.01 2.00 1.99 1.99 1.98 1.98 1.96 1.96 1.95 1.95 1.94 1.93 1.92 1.91 1.91 1.90 1.89 0.92 0.90
H_3C
O
O
H_3C
O
O
Cl
H_3C
O
O
Cl
3i-*R*
1.00 0.95 0.98 1.02 3.06 2.92 1.88 0.96 0.97 1.04 4.06 3.05
f1 (ppm)

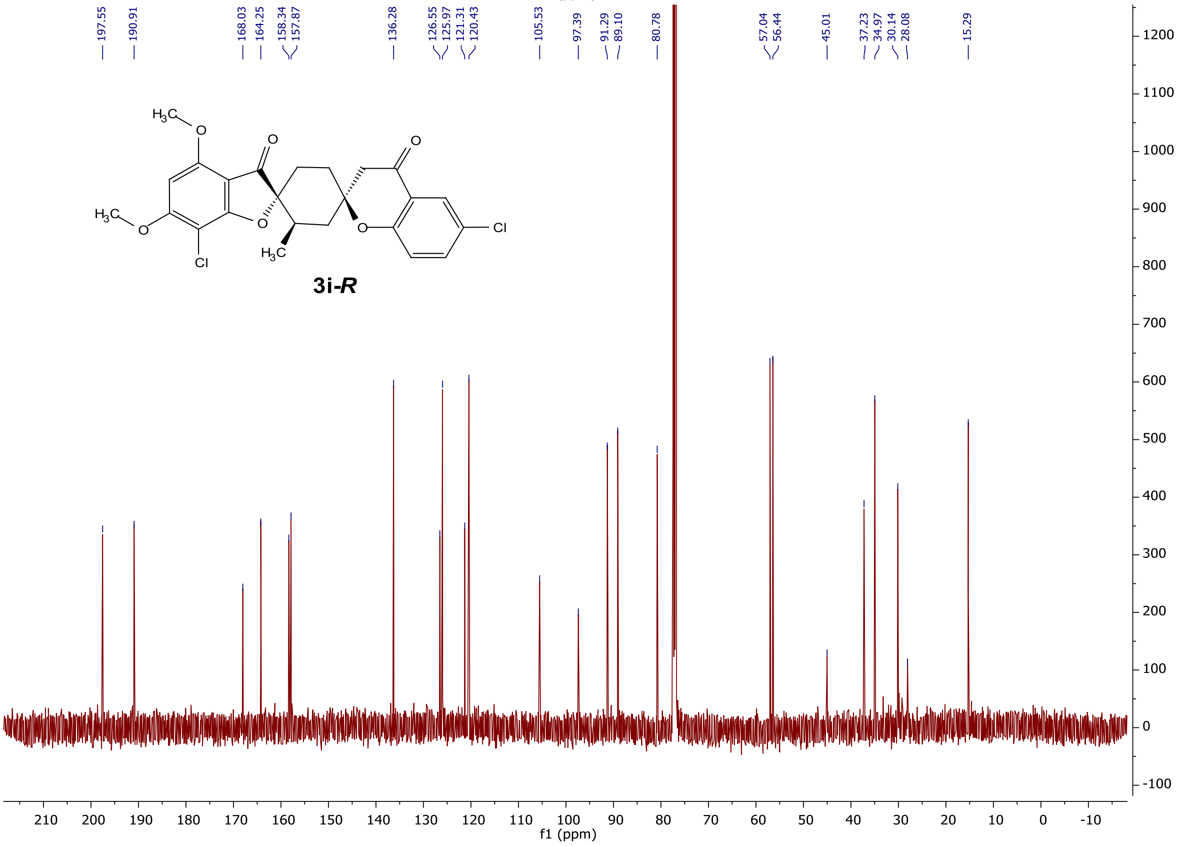
197.55 190.91 168.03 164.25 158.34 157.87 136.28 126.55 125.97 121.31 120.43 105.53 97.39 91.29 89.10 80.78 57.04 56.44 45.01 37.23 34.97 30.14 28.08 15.29
H_3C
O
O
H_3C
O
O
Cl
H_3C
O
O
Cl
3i-*R*
f1 (ppm)

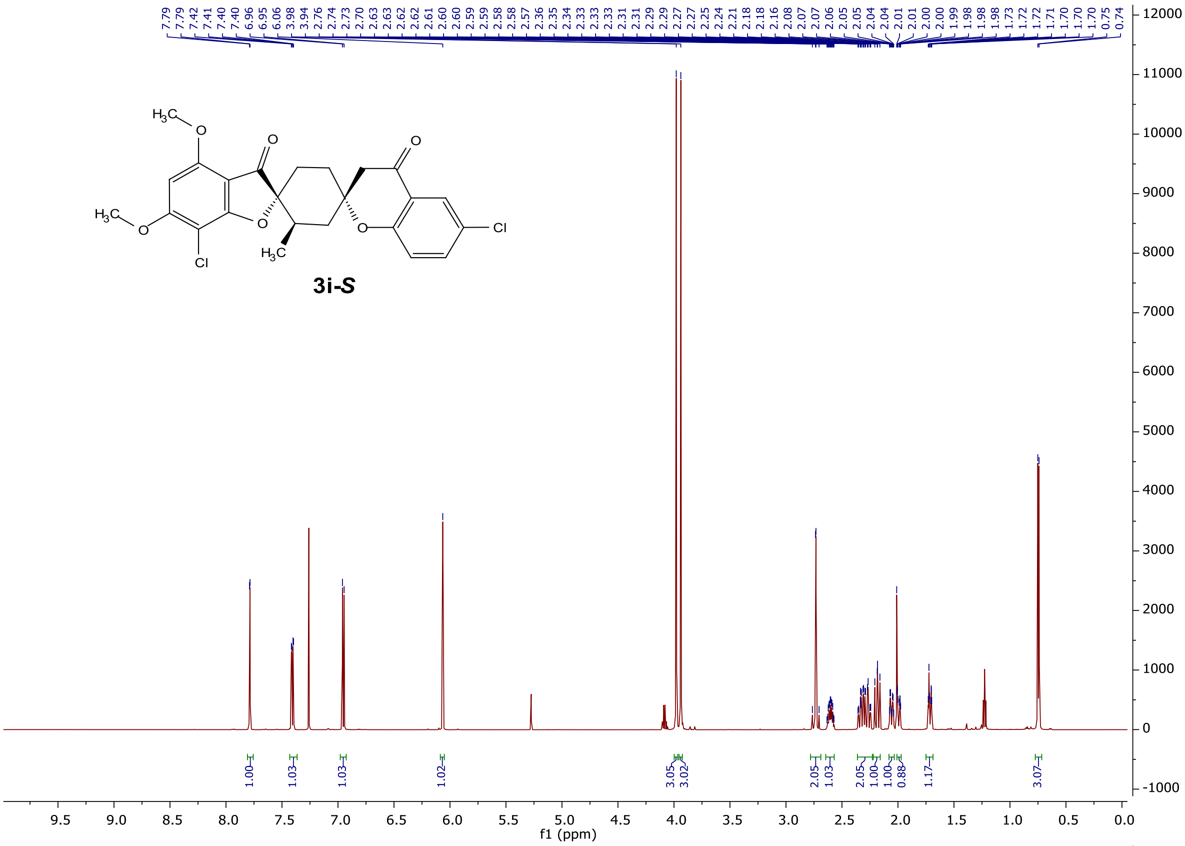
7.79 7.79 7.42 7.41 7.40 7.40 6.96 6.95 6.06 3.98 3.94 2.76 2.74 2.74 2.73 2.70 2.63 2.63 2.62 2.62 2.61 2.60 2.60 2.59 2.59 2.58 2.58 2.57 2.36 2.35 2.34 2.33 2.33 2.33 2.31 2.31 2.29 2.29 2.27 2.27 2.25 2.24 2.21 2.18 2.18 2.16 2.08 2.07 2.07 2.06 2.05 2.05 2.04 2.04 2.01 2.01 2.00 2.00 1.99 1.98 1.98 1.98 1.73 1.72 1.72 1.71 1.70 1.70 0.75 0.74
3i-S
1.00 1.03 1.03 1.02 3.05 3.02 2.05 1.03 2.05 1.00 1.00 0.88 1.17 3.07
f1 (ppm)

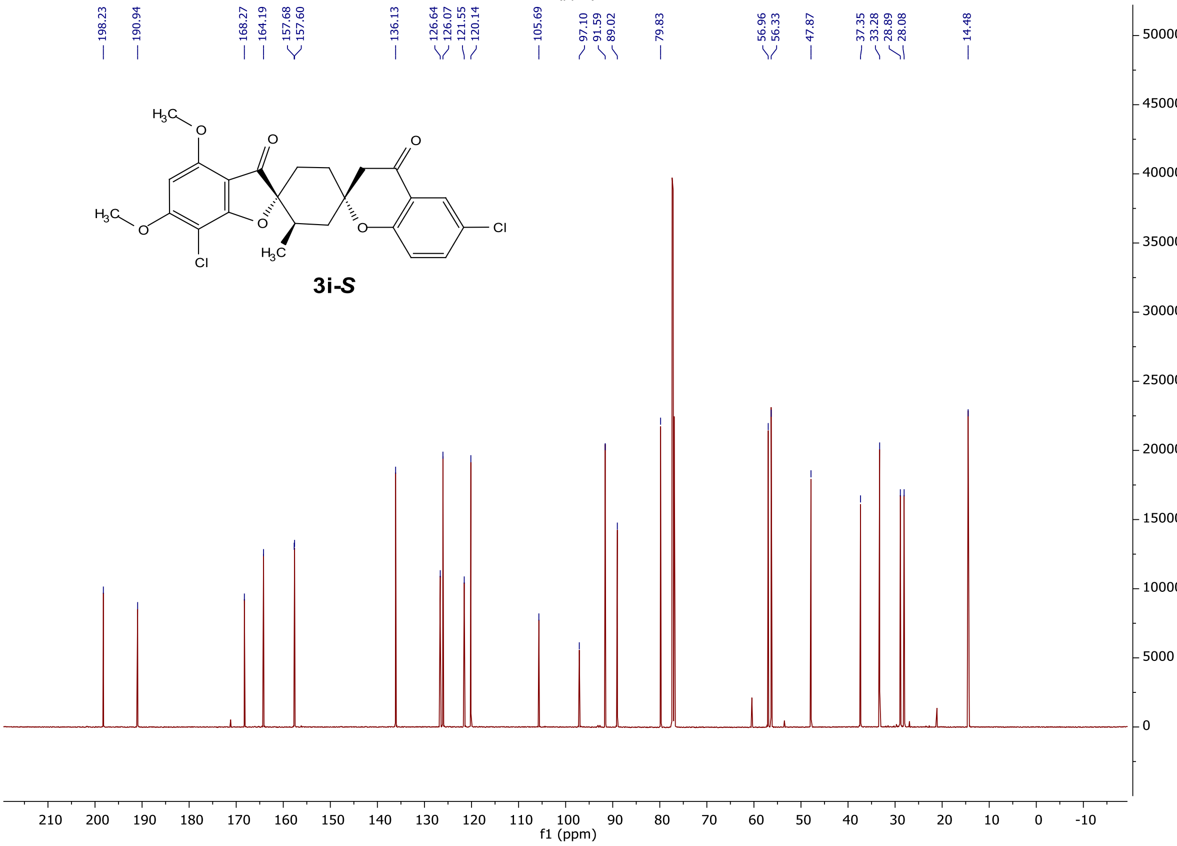
198.23 190.94 168.27 164.19 157.68 157.60 136.13 126.64 126.07 121.55 120.14 105.69 97.10 91.59 89.02 79.83 56.96 56.33 47.87 37.35 33.28 28.89 28.08 14.48
3i-S
f1 (ppm)

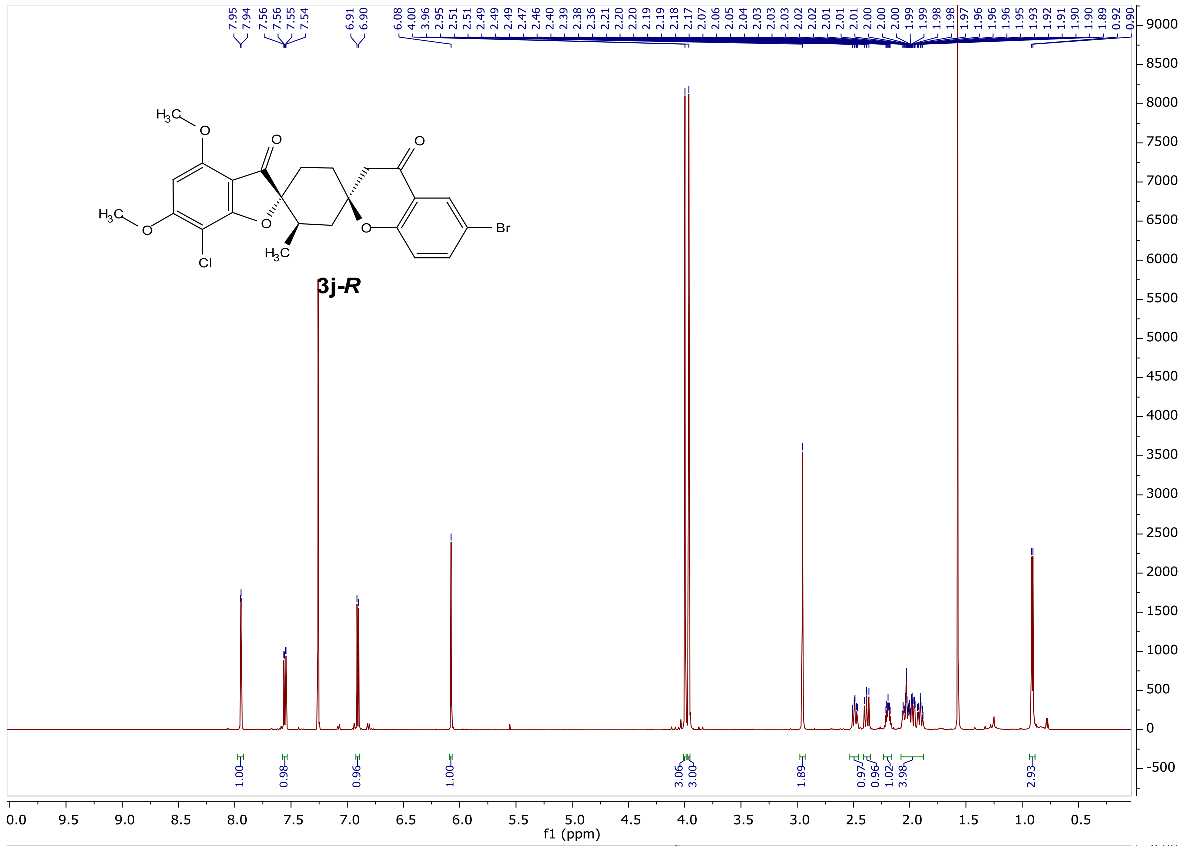
7.95
7.94
7.56
7.56
7.55
7.54
6.91
6.90
6.08
4.00
3.96
2.95
2.51
2.51
2.49
2.49
2.47
2.46
2.40
2.39
2.38
2.36
2.21
2.20
2.20
2.19
2.19
2.18
2.17
2.07
2.06
2.05
2.04
2.03
2.03
2.03
2.02
2.02
2.01
2.01
2.00
2.00
2.00
1.99
1.99
1.98
1.98
1.97
1.96
1.96
1.96
1.95
1.93
1.92
1.91
1.90
1.90
1.89
0.92
0.90
3j-*R*
1.00
0.98
0.96
1.00
3.06
3.00
1.89
0.97
0.96
1.02
3.98
2.93
f1 (ppm)

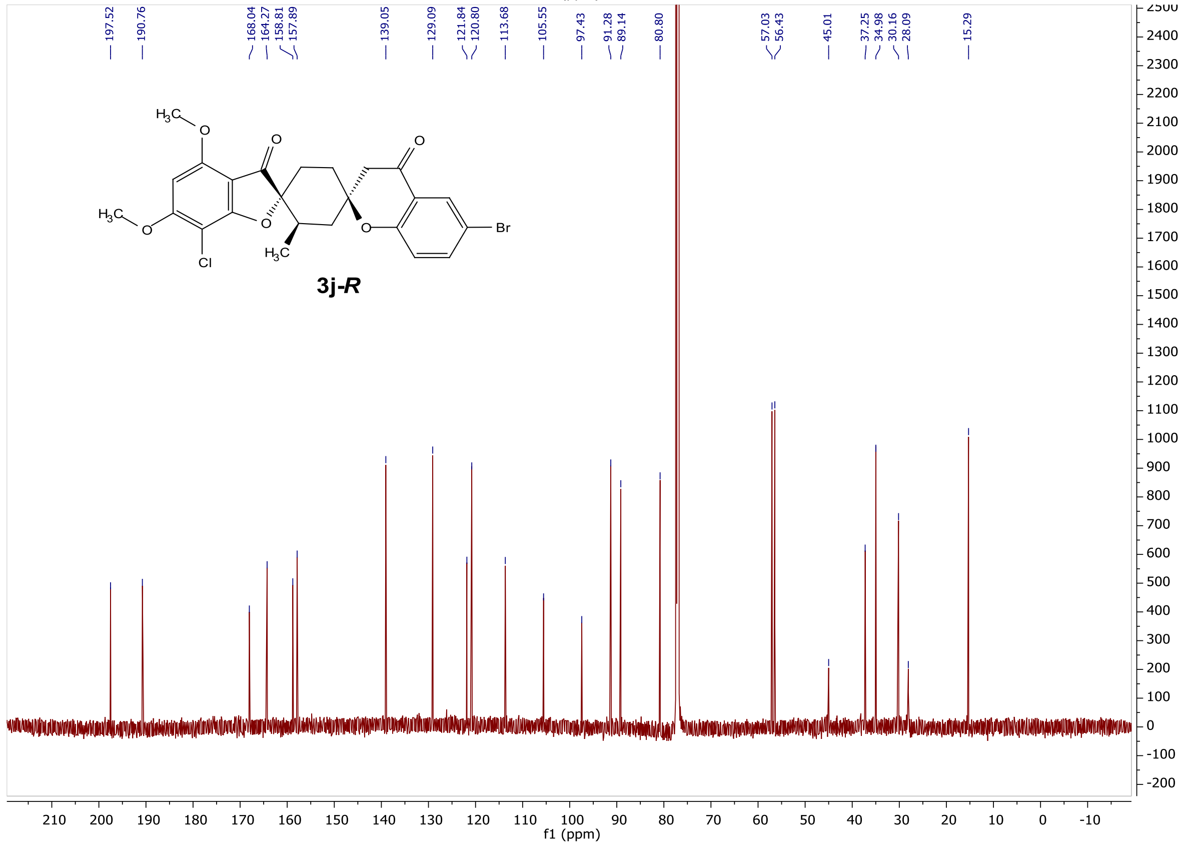
197.52
190.76
168.04
164.27
158.81
157.89
139.05
129.09
121.84
120.80
113.68
105.55
97.43
91.28
89.14
80.80
57.03
56.43
45.01
37.25
34.98
30.16
28.09
15.29
3j-*R*
f1 (ppm)

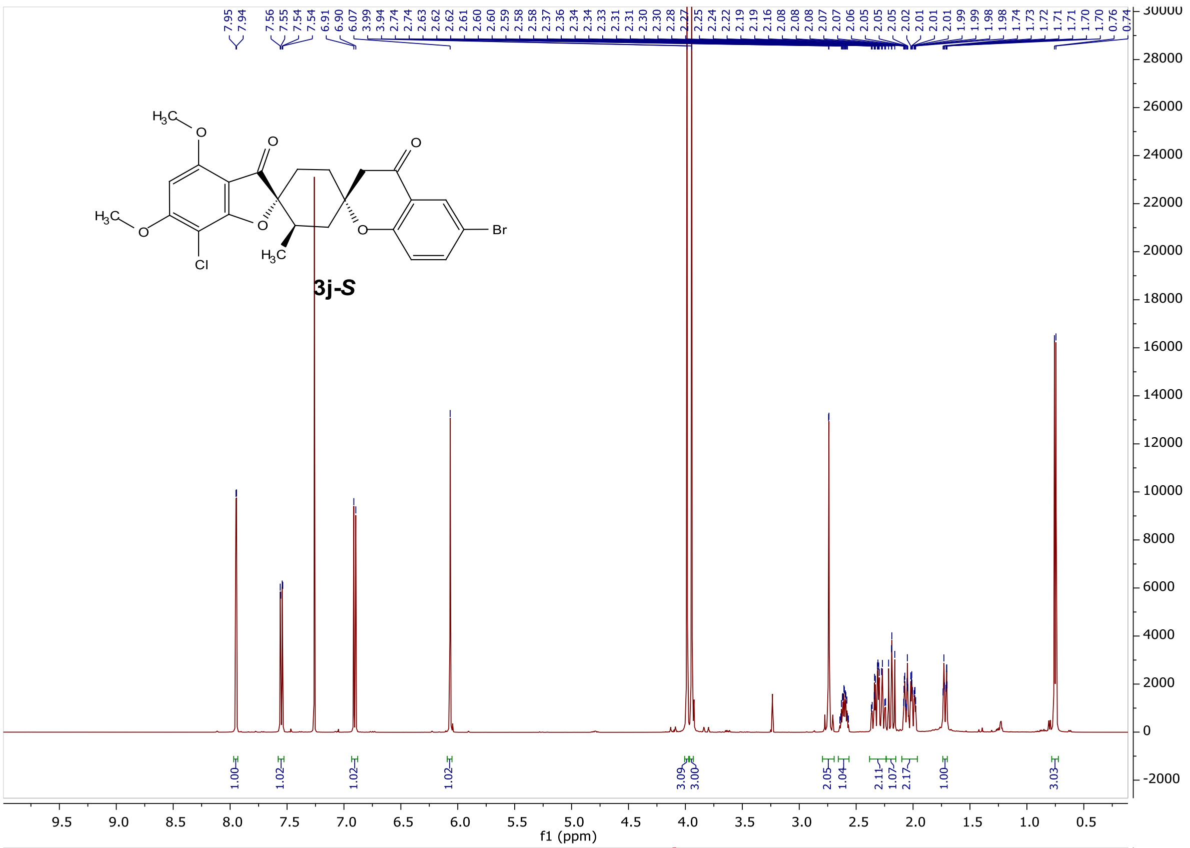
3j-S
f1 (ppm)

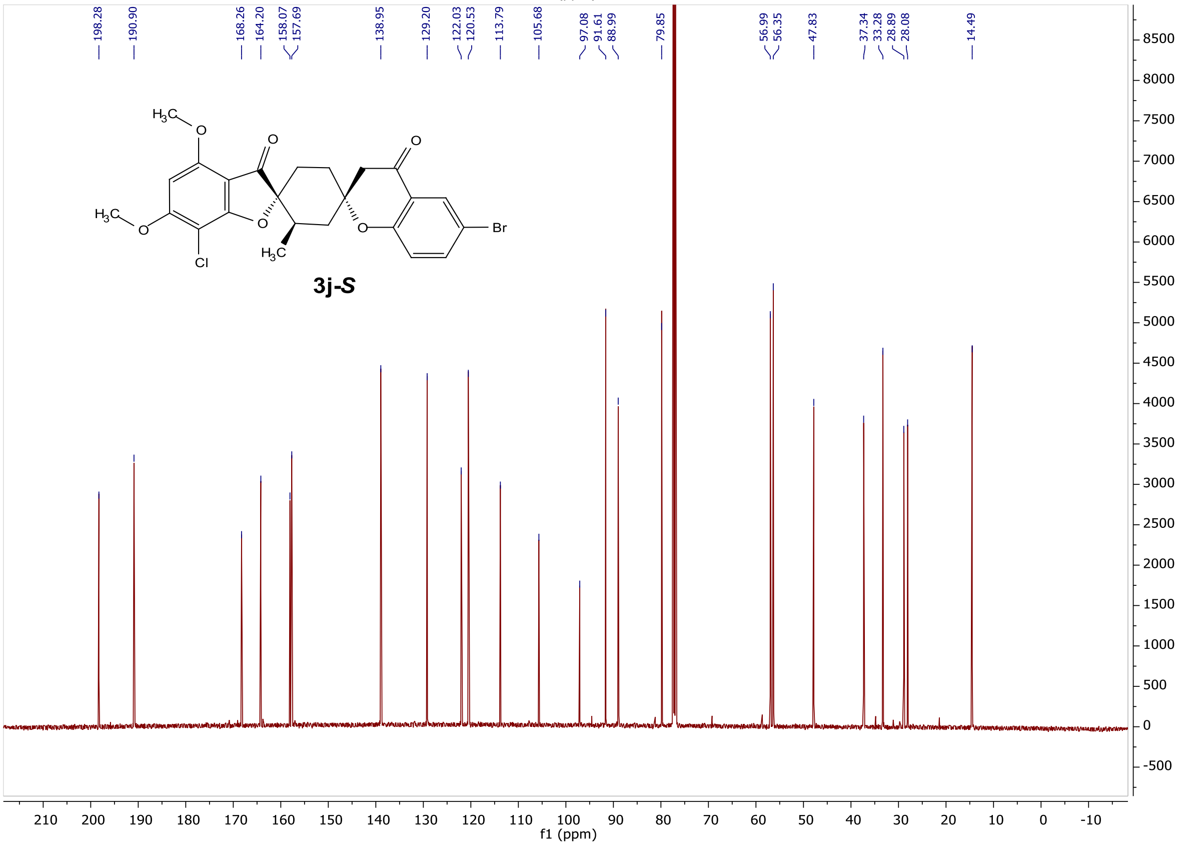
3j-S
f1 (ppm)

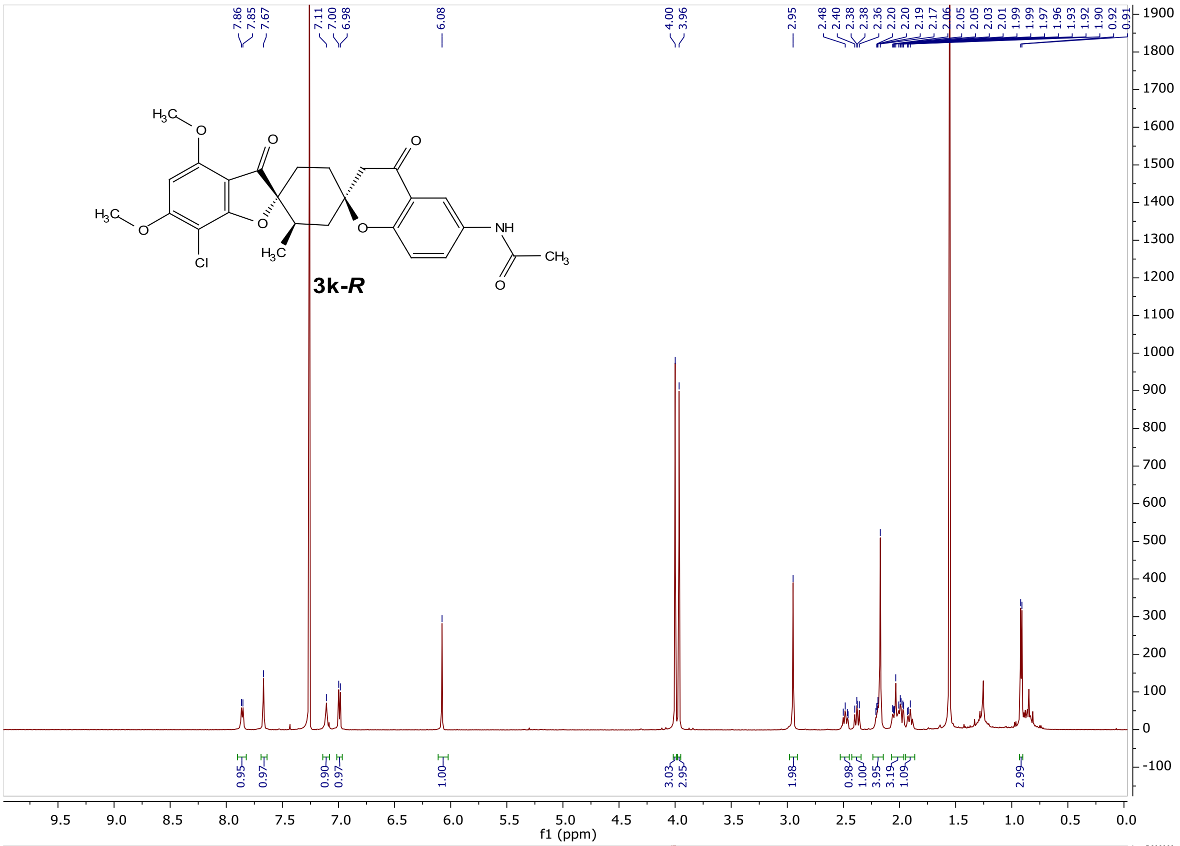
7.86
7.85
7.67
7.11
7.00
6.98
6.08
4.00
3.96
2.95
2.48
2.40
2.38
2.38
2.36
2.20
2.20
2.20
2.19
2.17
2.06
2.05
2.05
2.03
2.01
1.99
1.99
1.97
1.96
1.93
1.92
1.90
0.92
0.91
3k-R
0.95
0.97
0.90
0.97
1.00
3.03
2.95
1.98
0.98
1.00
3.95
3.19
1.09
2.99
f1 (ppm)

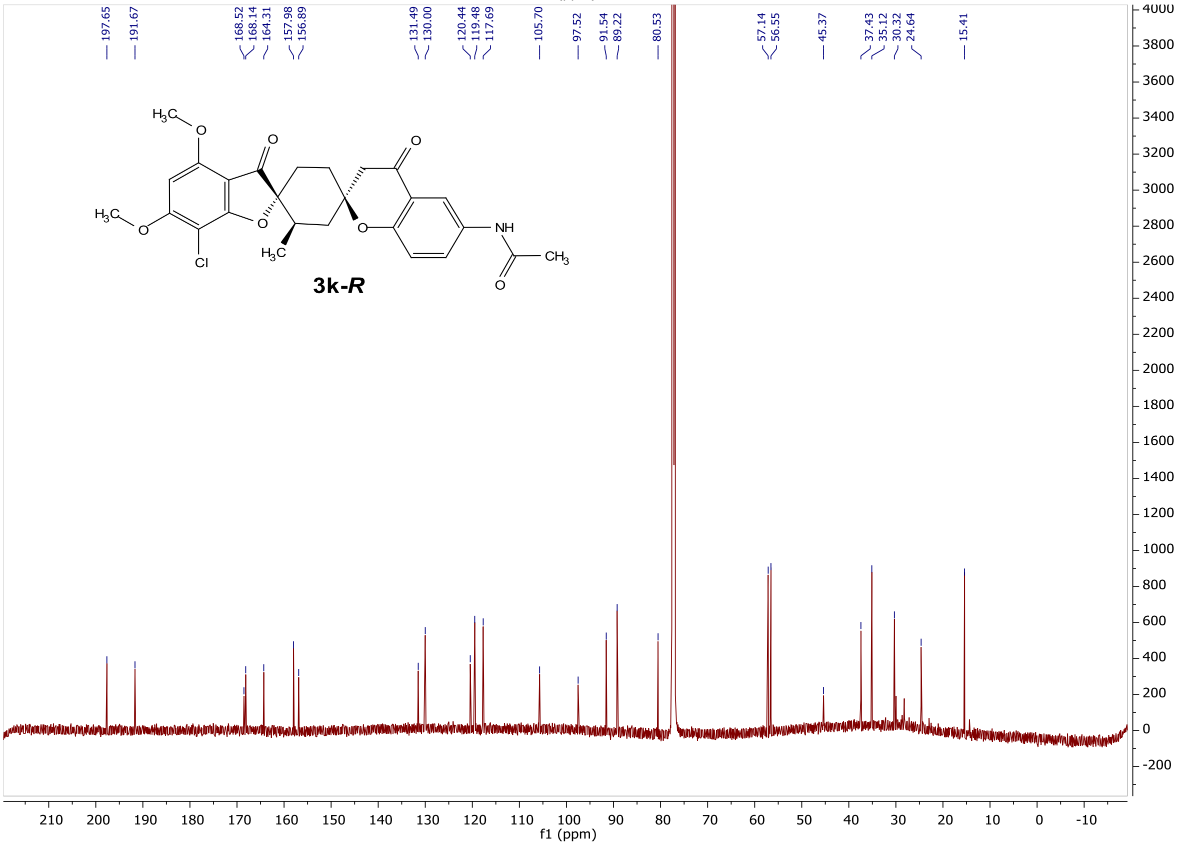
197.65
191.67
168.52
168.14
164.31
157.98
156.89
131.49
130.00
120.44
119.48
117.69
105.70
97.52
91.54
89.22
80.53
57.14
56.55
45.37
37.43
35.12
30.32
24.64
15.41
3k-R
f1 (ppm)

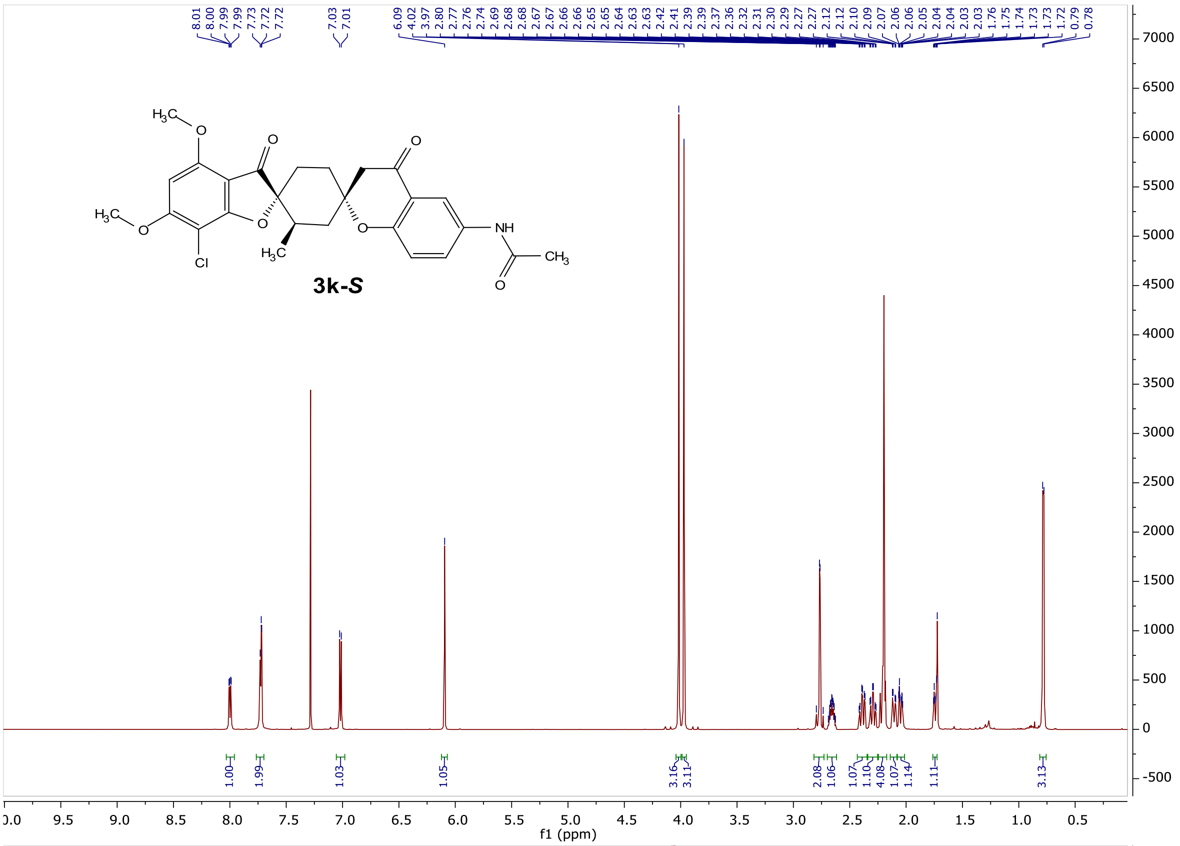
8.01 8.00 7.99 7.99 7.73 7.72 7.72 7.72 7.03 7.01 6.09 4.02 3.97 2.80 2.77 2.76 2.74 2.69 2.68 2.68 2.67 2.67 2.66 2.66 2.65 2.65 2.64 2.63 2.63 2.42 2.41 2.39 2.39 2.37 2.36 2.32 2.31 2.30 2.29 2.27 2.27 2.12 2.12 2.10 2.09 2.07 2.06 2.06 2.05 2.04 2.04 2.03 2.03 1.76 1.75 1.74 1.73 1.73 1.72 0.79 0.78
3k-S
1.00 1.99 1.03 1.05 3.16 3.11 2.08 1.06 1.07 1.10 4.08 1.07 1.14 1.11 3.13
f1 (ppm)

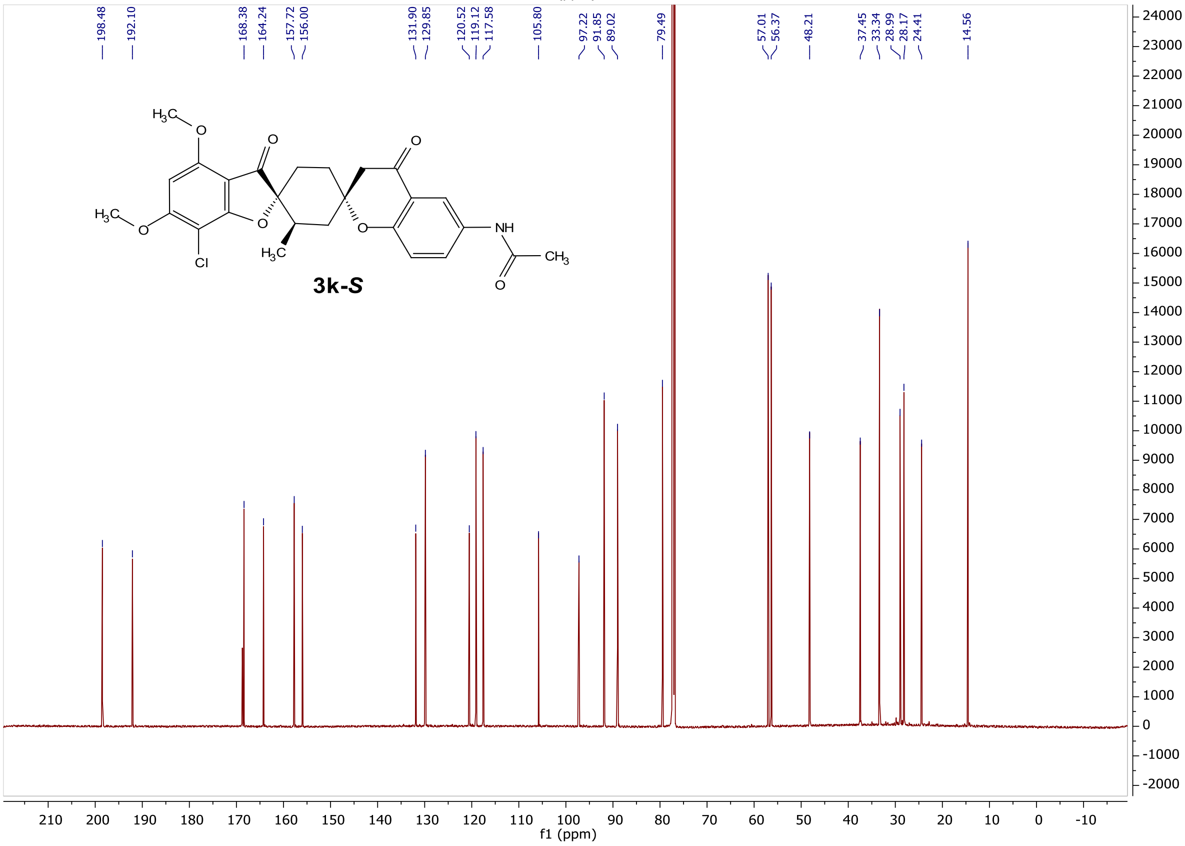
198.48 192.10 168.38 164.24 157.72 156.00 131.90 129.85 120.52 119.12 117.58 105.80 97.22 91.85 89.02 79.49 57.01 56.37 48.21 37.45 33.34 28.99 28.17 24.41 14.56
3k-S
f1 (ppm)

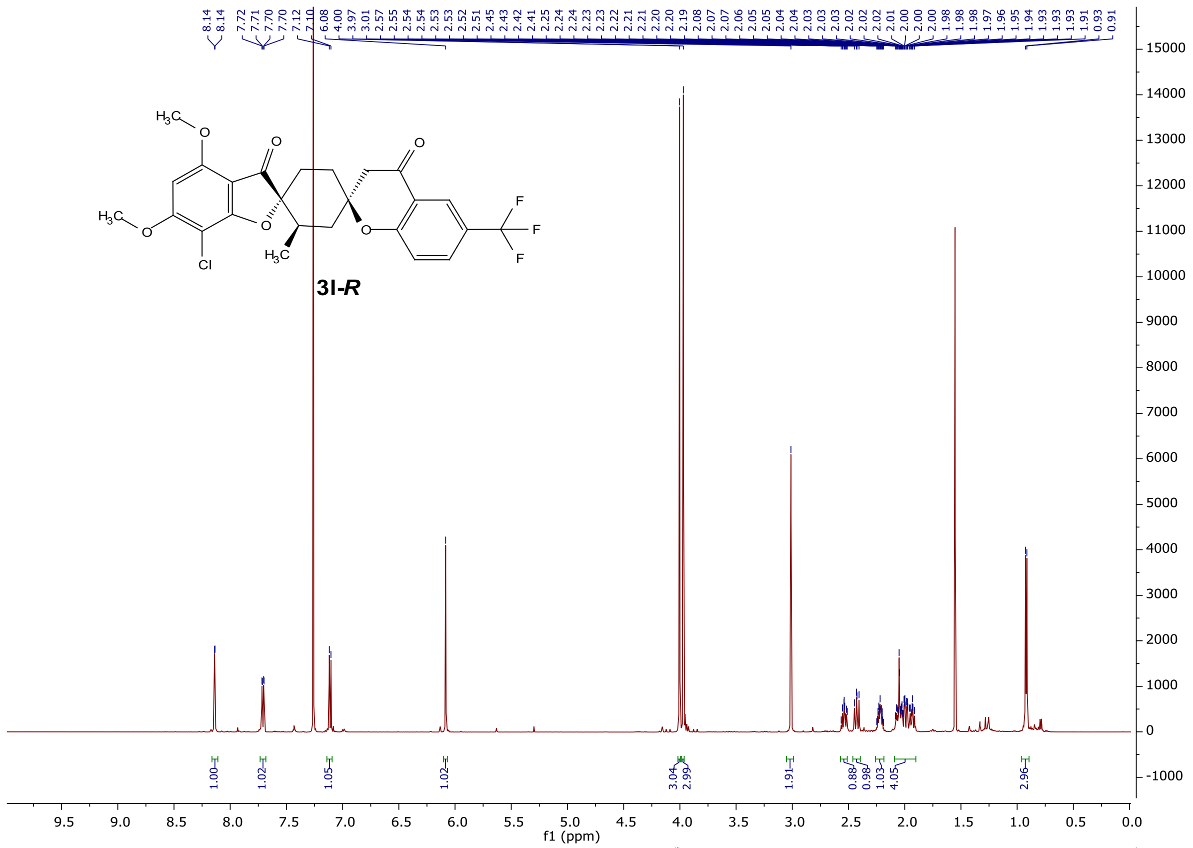
3l-*R*
f1 (ppm)

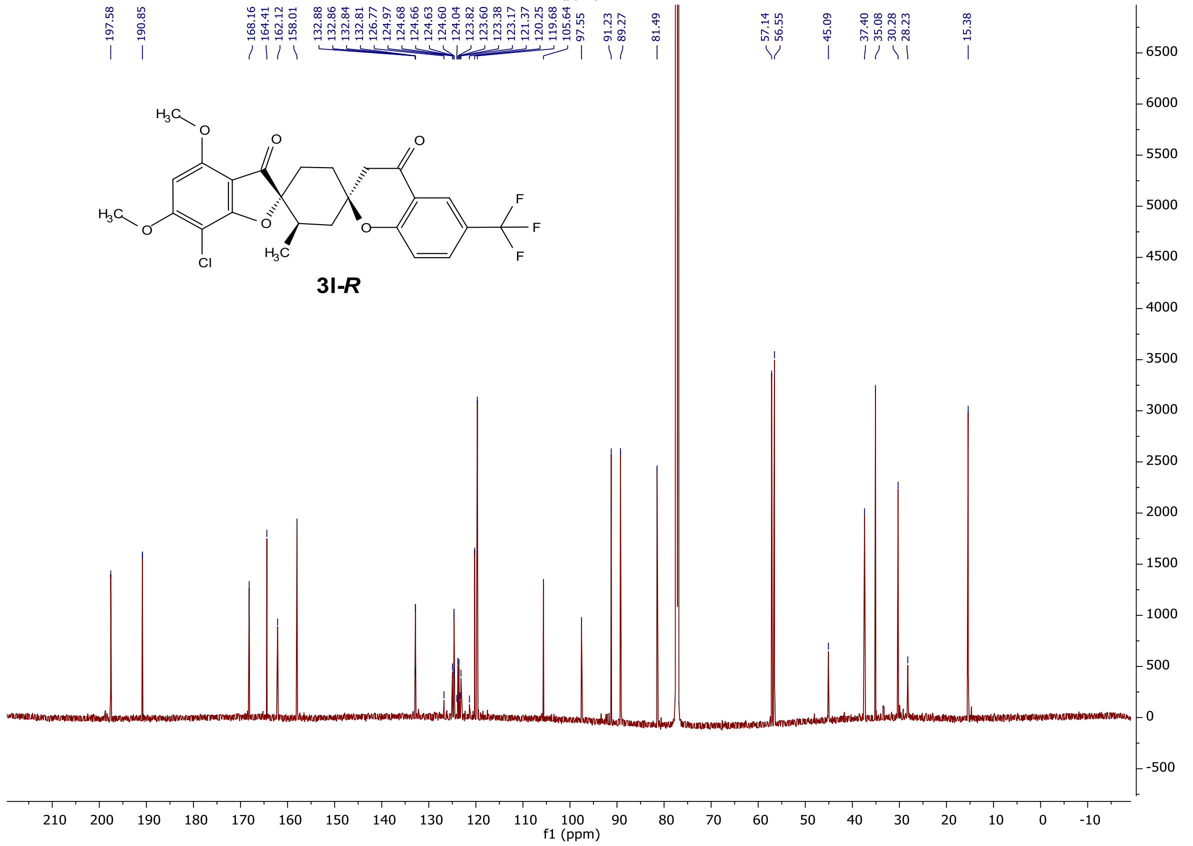
3l-*R*
f1 (ppm)

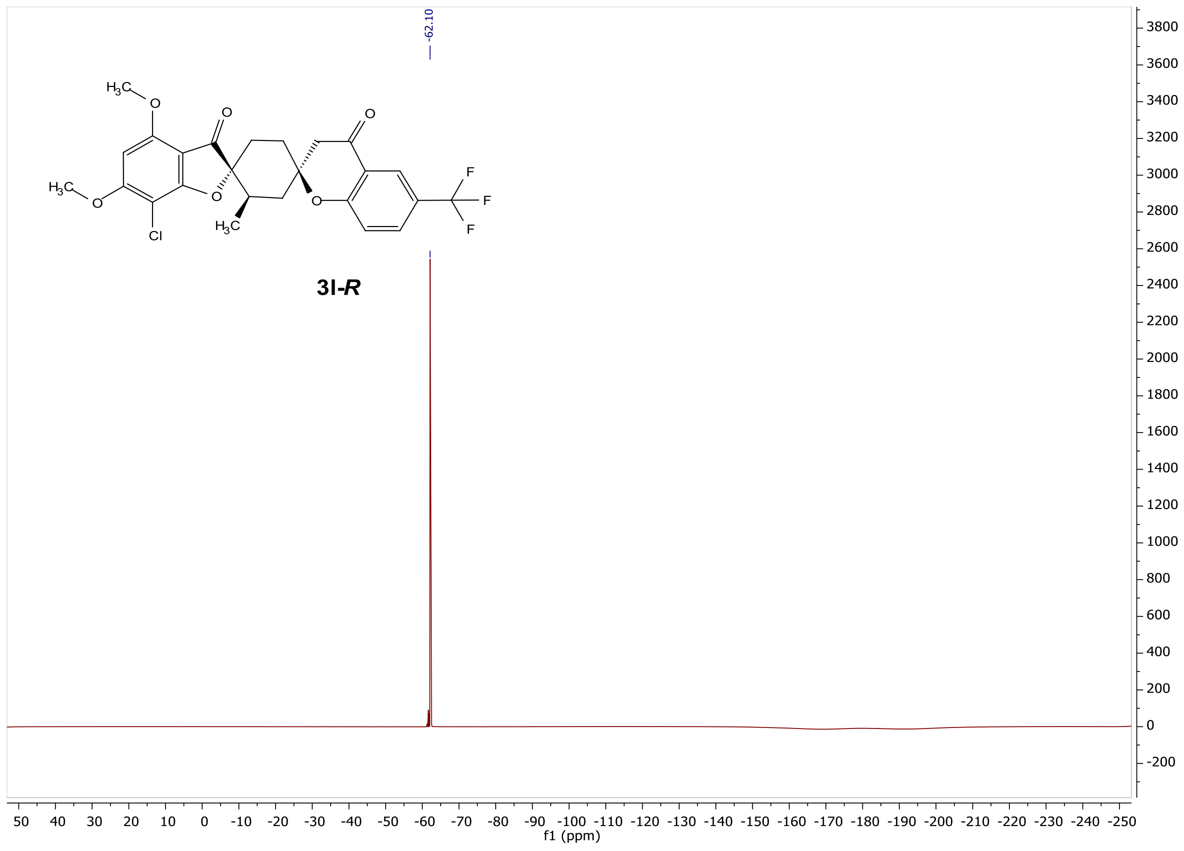

-62.10
3l-R
f1 (ppm)

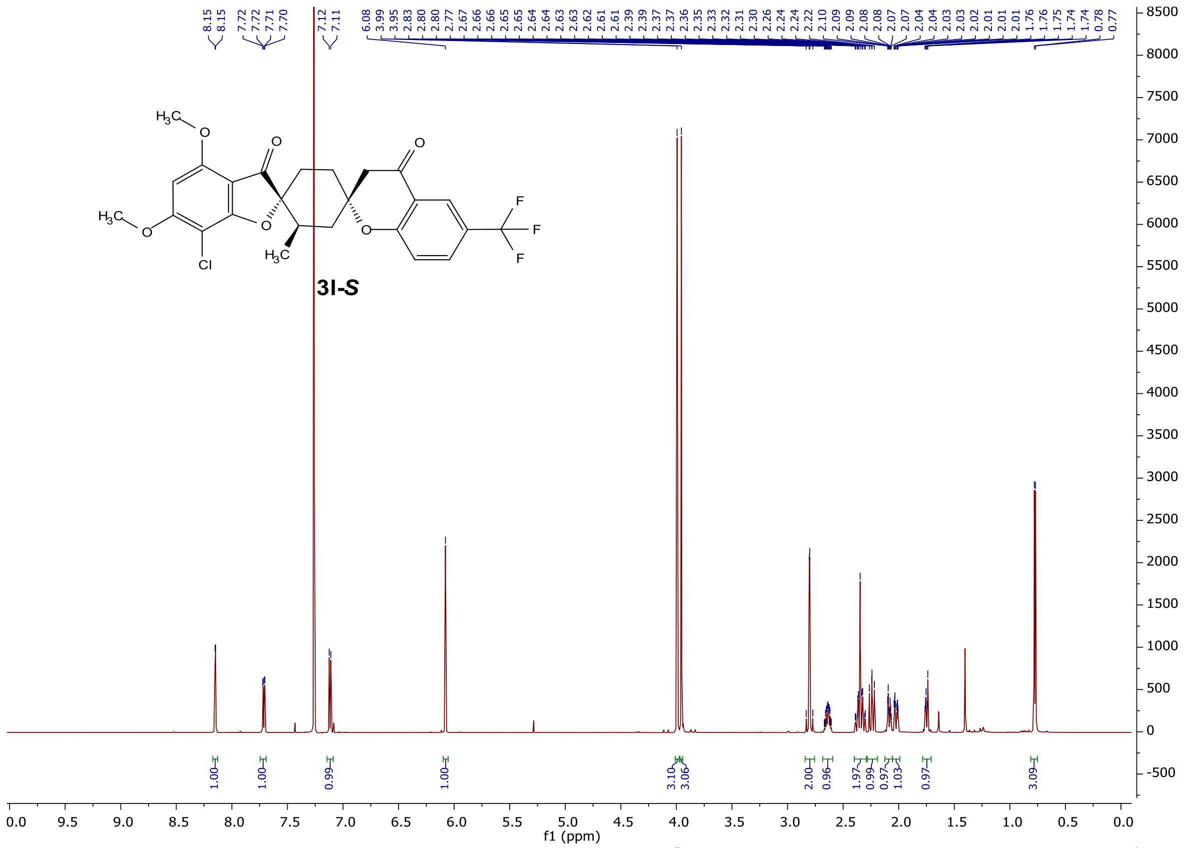

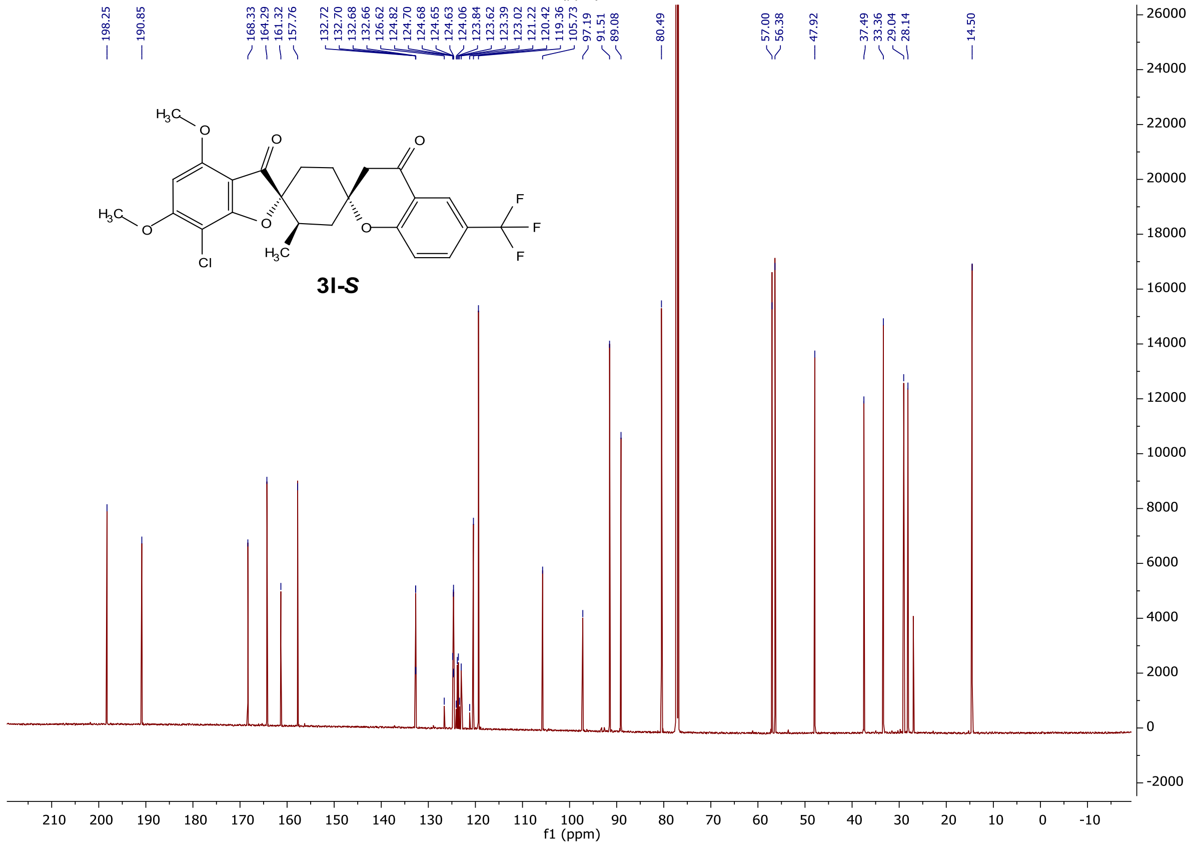

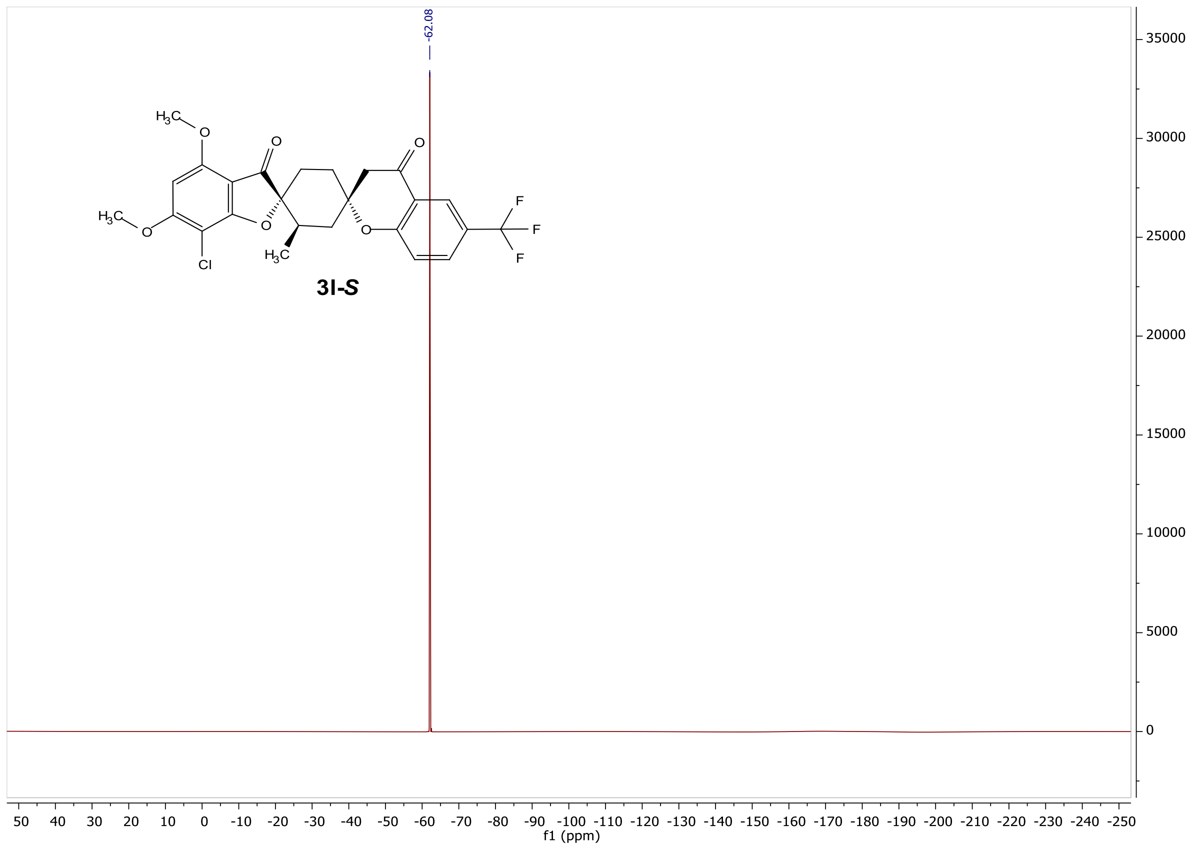

-62.08
3l-S
f1 (ppm)

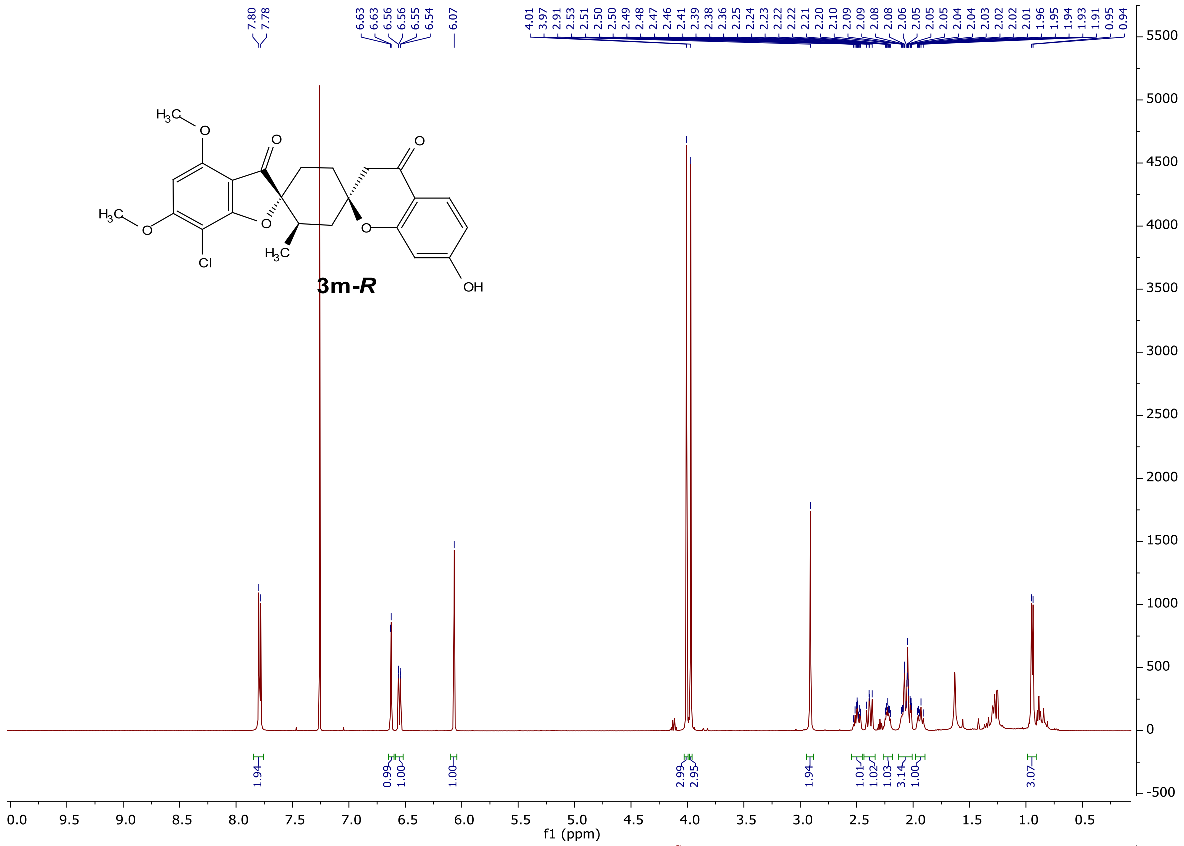
3m-R
f1 (ppm)

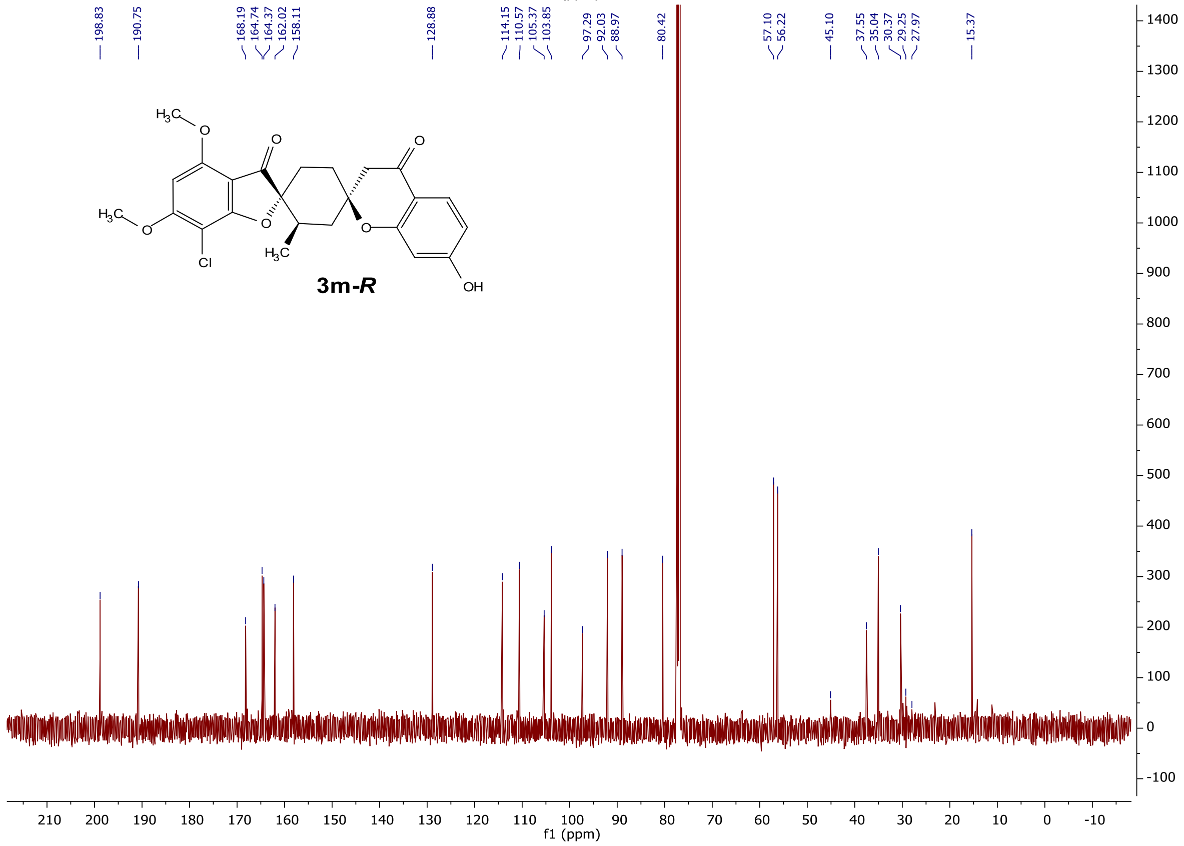
3m-R
f1 (ppm)

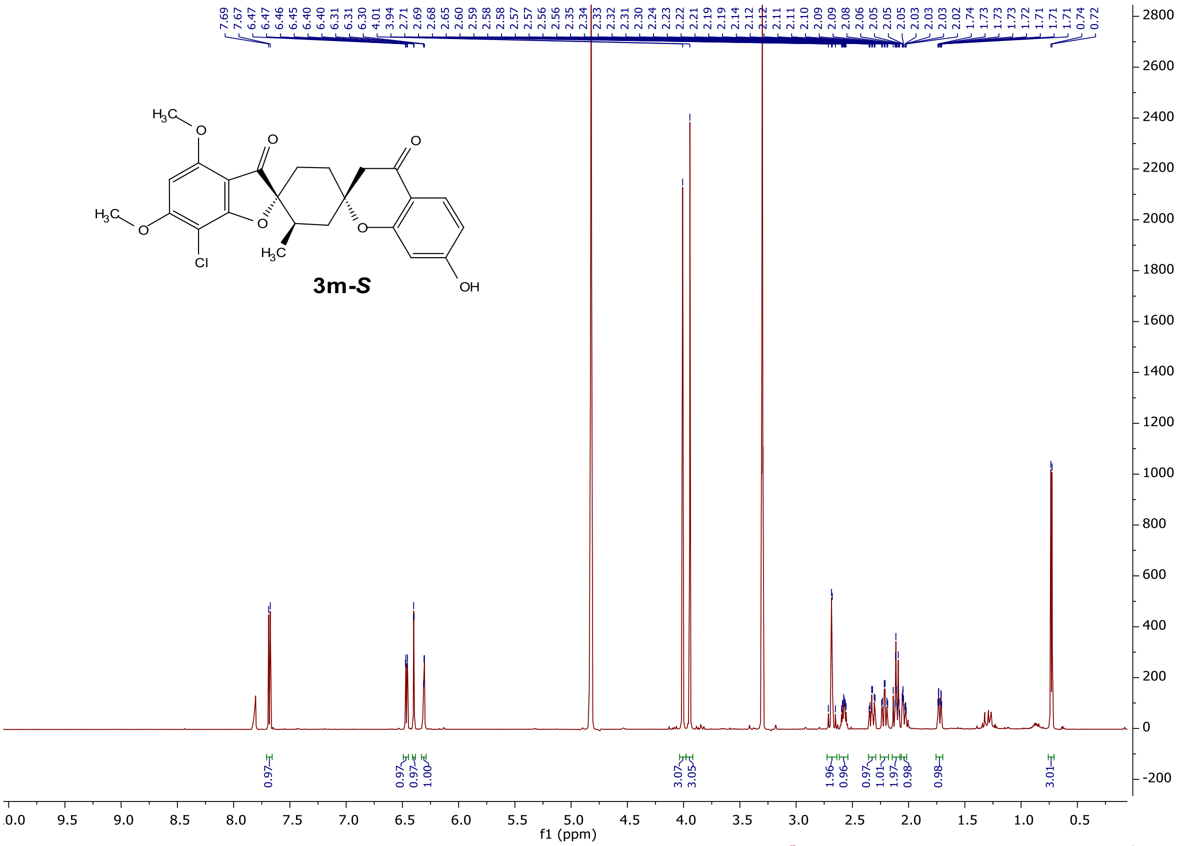
7.69 7.67 6.47 6.47 6.46 6.45 6.40 6.40 6.31 6.31 6.30 4.01 3.94 2.71 2.69 2.68 2.65 2.60 2.59 2.58 2.58 2.57 2.57 2.56 2.56 2.35 2.34 2.33 2.32 2.31 2.30 2.24 2.23 2.22 2.21 2.19 2.19 2.14 2.12 2.12 2.11 2.11 2.10 2.09 2.09 2.08 2.06 2.05 2.05 2.05 2.03 2.03 2.03 2.02 1.74 1.73 1.73 1.73 1.72 1.71 1.71 0.74 0.72
0.97 0.97 0.97 1.00 3.07 3.05 1.96 0.96 0.97 1.01 1.97 0.98 0.98 3.01
3m-S
f1 (ppm)

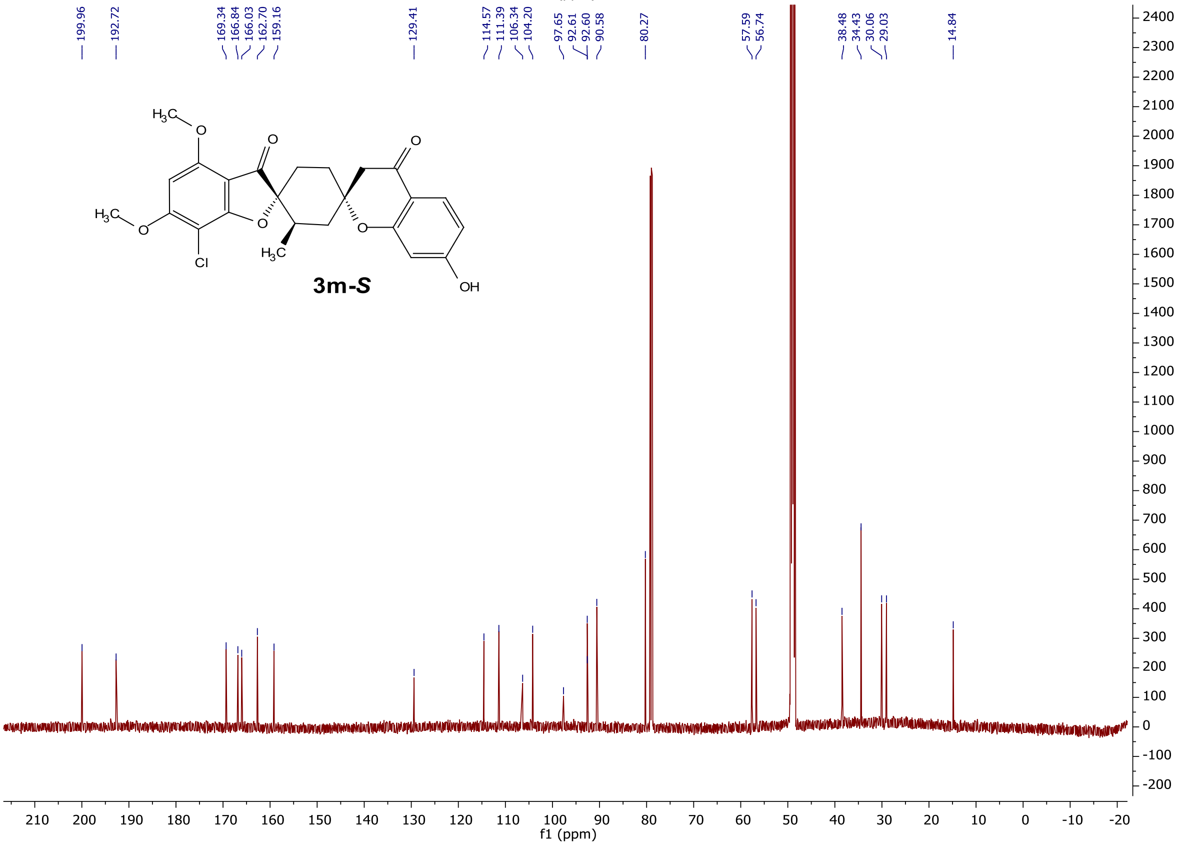
199.96 192.72 169.34 166.84 166.03 162.70 159.16 129.41 114.57 111.39 106.34 104.20 97.65 92.61 92.60 90.58 80.27 57.59 56.74 38.48 34.43 30.06 29.03 14.84
3m-S
f1 (ppm)

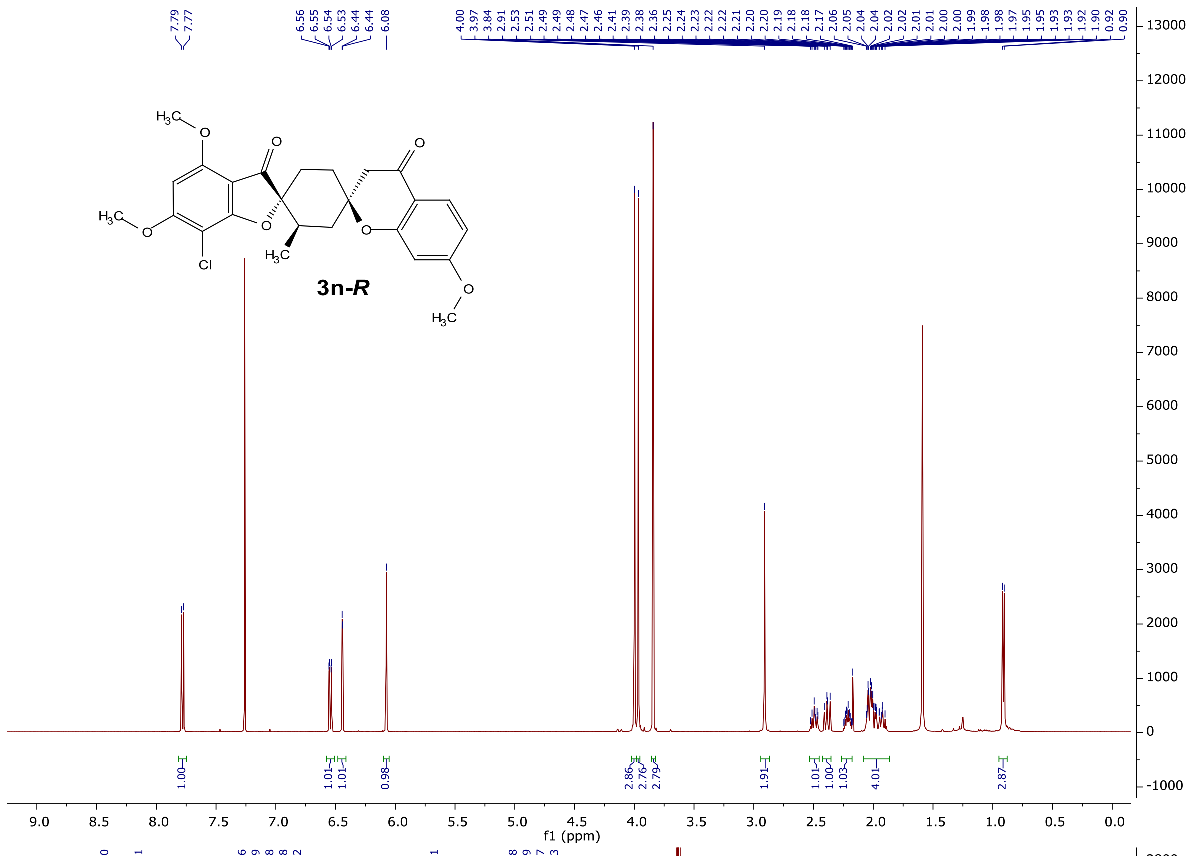

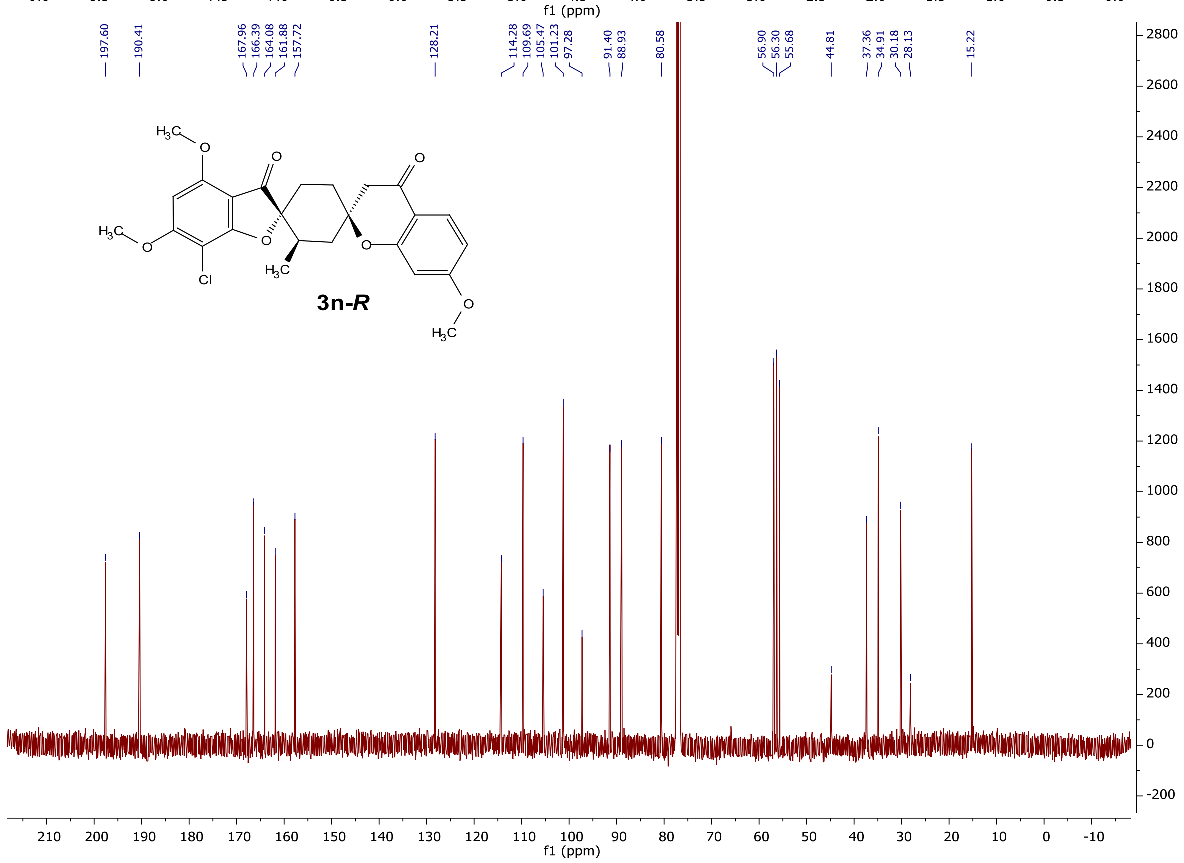

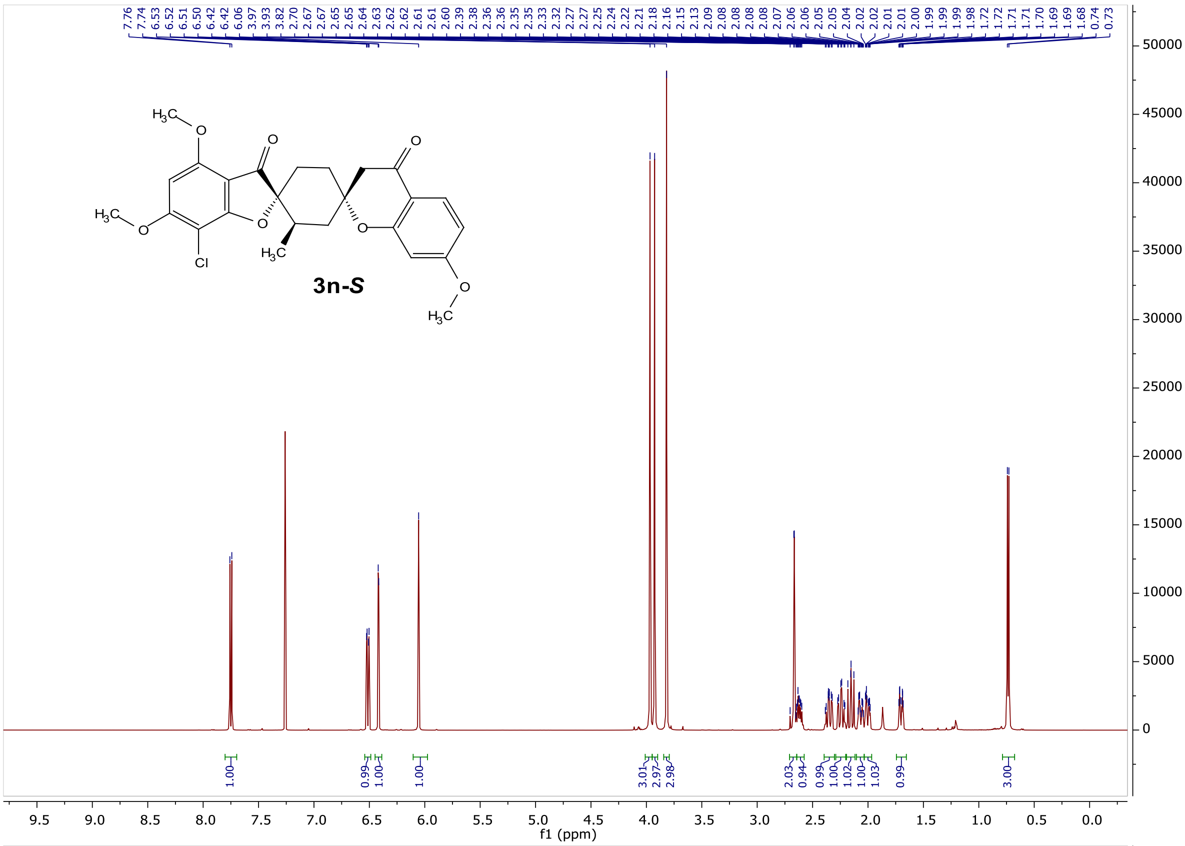
7.76 7.74 6.53 6.52 6.51 6.50 6.42 6.42 6.06 3.97 3.93 3.82 2.70 2.67 2.67 2.65 2.65 2.64 2.63 2.62 2.62 2.61 2.61 2.60 2.39 2.38 2.36 2.36 2.35 2.35 2.33 2.32 2.27 2.27 2.25 2.24 2.22 2.21 2.18 2.16 2.15 2.13 2.09 2.08 2.08 2.08 2.08 2.07 2.06 2.06 2.05 2.05 2.04 2.02 2.02 2.02 2.01 2.01 2.00 1.99 1.99 1.99 1.98 1.72 1.72 1.71 1.71 1.70 1.69 1.68 0.74 0.73
3n-S
1.00 0.99 1.00 1.00 3.01 2.97 2.98 2.03 0.94 0.99 1.00 1.02 1.00 1.03 0.99 3.00
f1 (ppm)

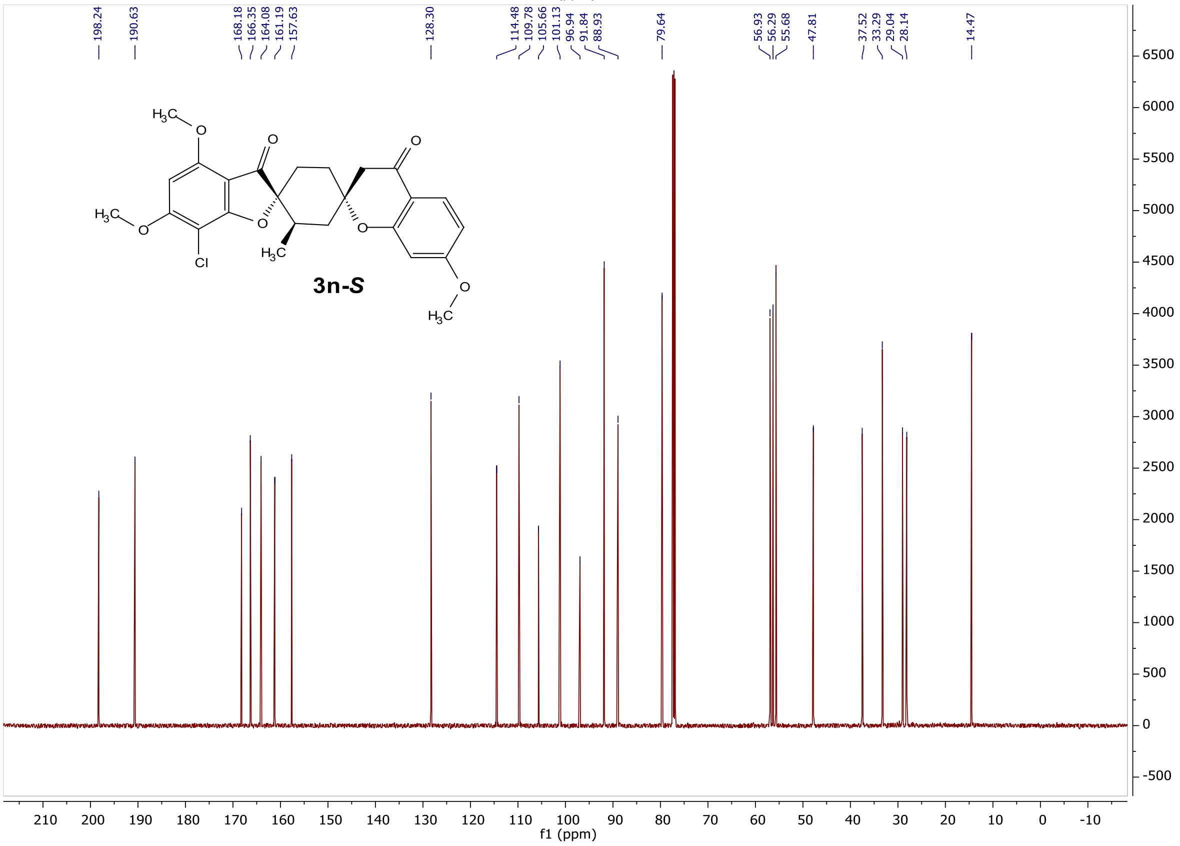
198.24 190.63 168.18 166.35 164.08 161.19 157.63 128.30 114.48 109.78 105.66 101.13 96.94 91.84 88.93 79.64 56.93 56.29 55.68 47.81 37.52 33.29 29.04 28.14 14.47
3n-S
f1 (ppm)

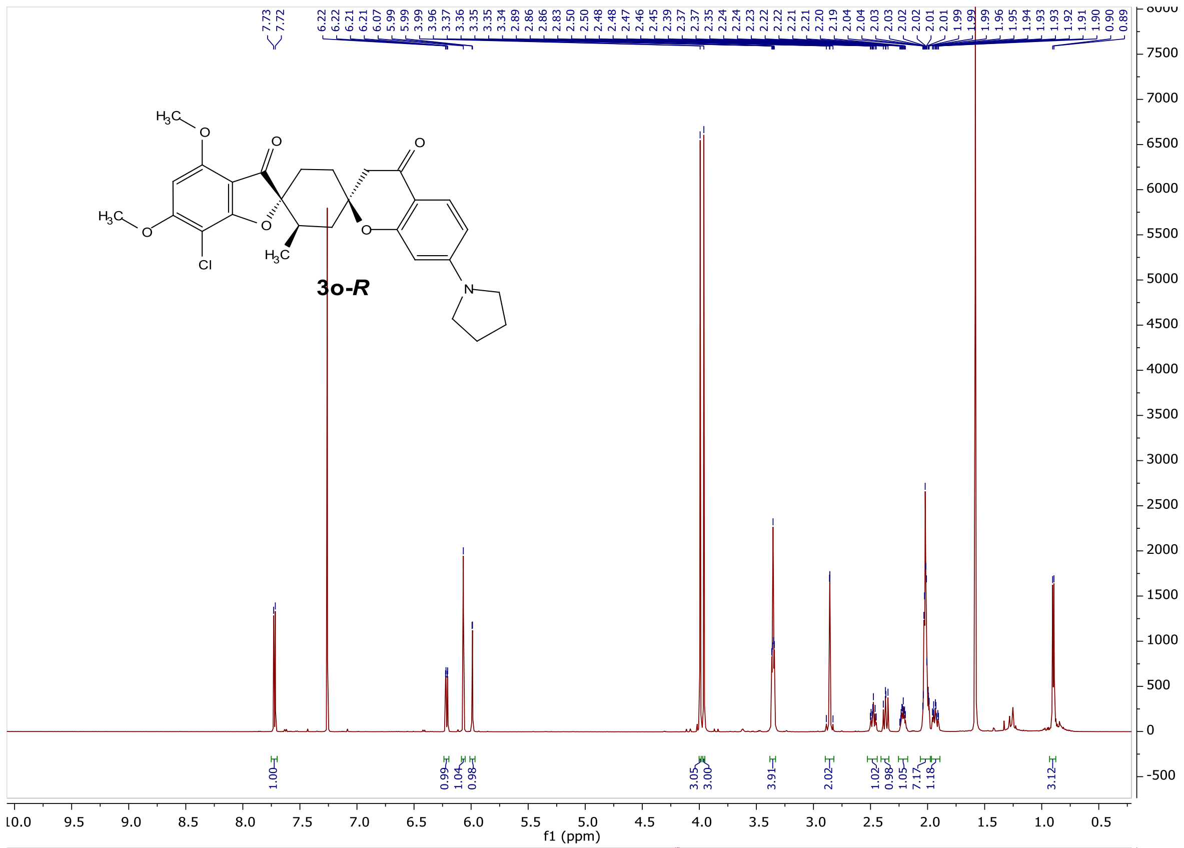
7.73
7.72
6.22
6.22
6.21
6.21
6.07
5.99
5.99
3.99
3.96
3.37
3.36
3.35
3.35
3.34
2.89
2.86
2.86
2.83
2.50
2.50
2.48
2.47
2.46
2.45
2.39
2.37
2.37
2.35
2.24
2.24
2.23
2.22
2.22
2.21
2.21
2.20
2.19
2.04
2.03
2.03
2.02
2.02
2.01
2.01
1.99
1.99
1.99
1.96
1.95
1.94
1.93
1.93
1.92
1.91
1.90
0.90
0.89
H3C
O
O
O
O
H3C
O
Cl
H3C
N
3o-R
1.00
0.99
1.04
0.98
3.05
3.00
3.91
2.02
1.02
0.98
1.05
7.17
1.18
3.12
f1 (ppm)

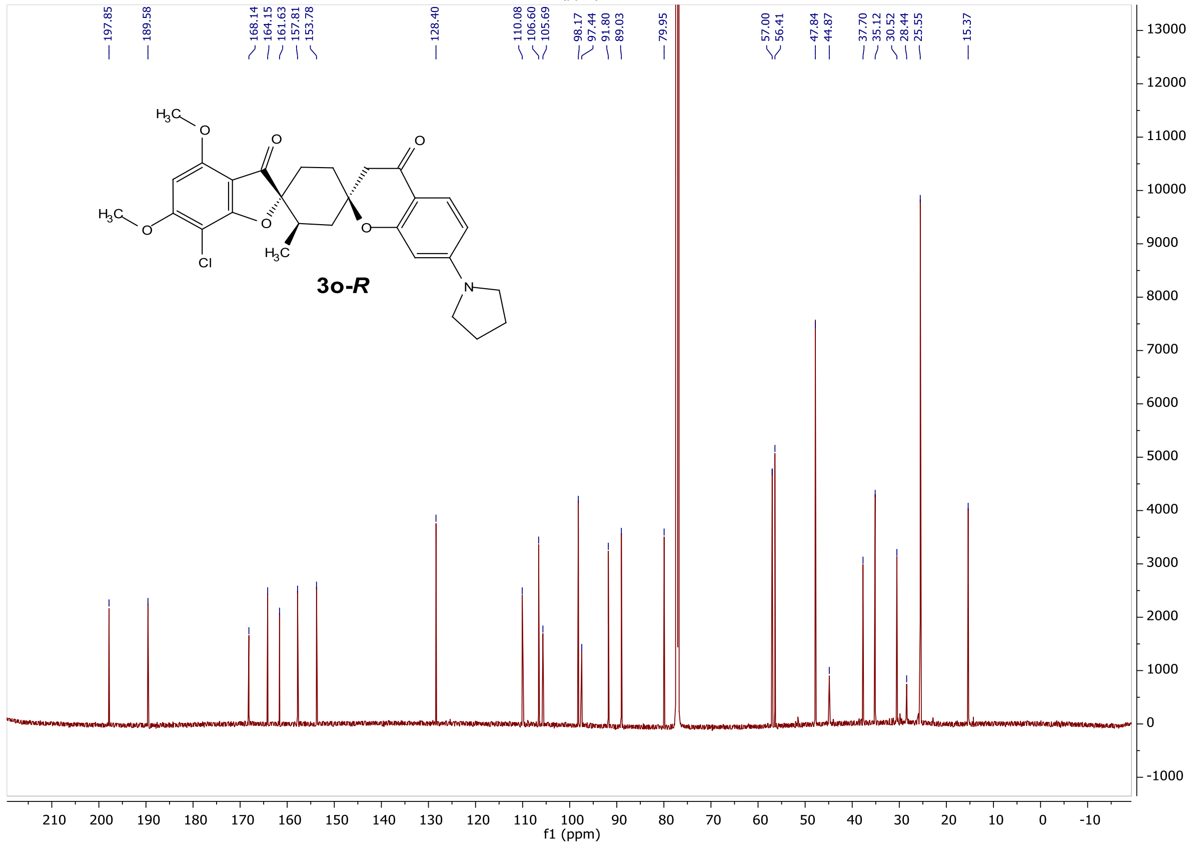
197.85
189.58
168.14
164.15
161.63
157.81
153.78
128.40
110.08
106.60
105.69
98.17
97.44
91.80
89.03
79.95
57.00
56.41
47.84
44.87
37.70
35.12
30.52
28.44
25.55
15.37
H3C
O
O
O
O
H3C
O
Cl
H3C
N
3o-R
f1 (ppm)

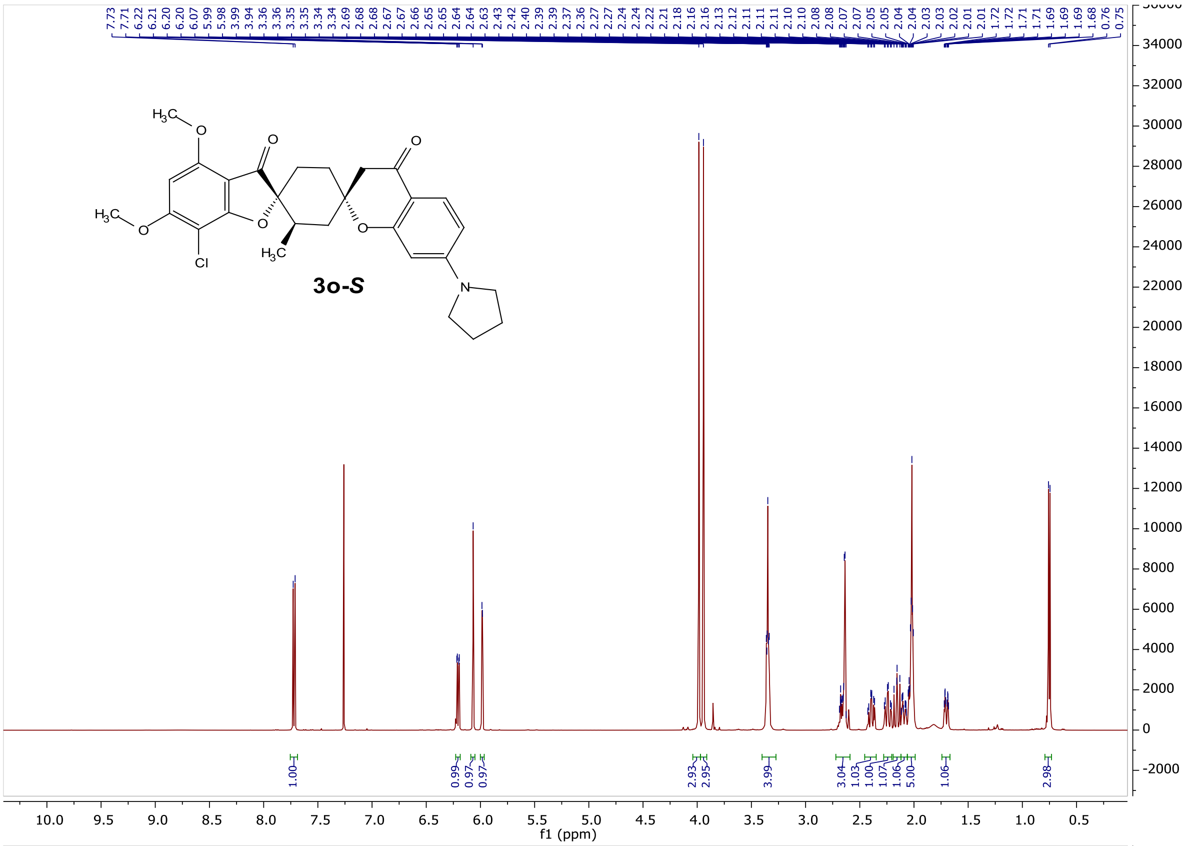
7.73 7.71 6.22 6.21 6.20 6.20 6.07 5.99 5.98 3.99 3.94 3.36 3.36 3.35 3.35 3.34 3.34 2.69 2.68 2.68 2.67 2.67 2.66 2.65 2.65 2.64 2.64 2.63 2.43 2.42 2.40 2.39 2.39 2.37 2.36 2.27 2.27 2.24 2.24 2.22 2.21 2.18 2.16 2.16 2.13 2.12 2.11 2.11 2.11 2.10 2.10 2.08 2.08 2.07 2.07 2.05 2.05 2.04 2.04 2.03 2.03 2.02 2.02 2.01 2.01 1.72 1.72 1.71 1.71 1.69 1.69 1.69 1.68 0.76 0.75
3o-S
1.00 0.99 0.97 0.97 2.93 2.95 3.99 3.04 1.03 1.00 1.07 1.06 5.00 1.06 2.98
f1 (ppm)

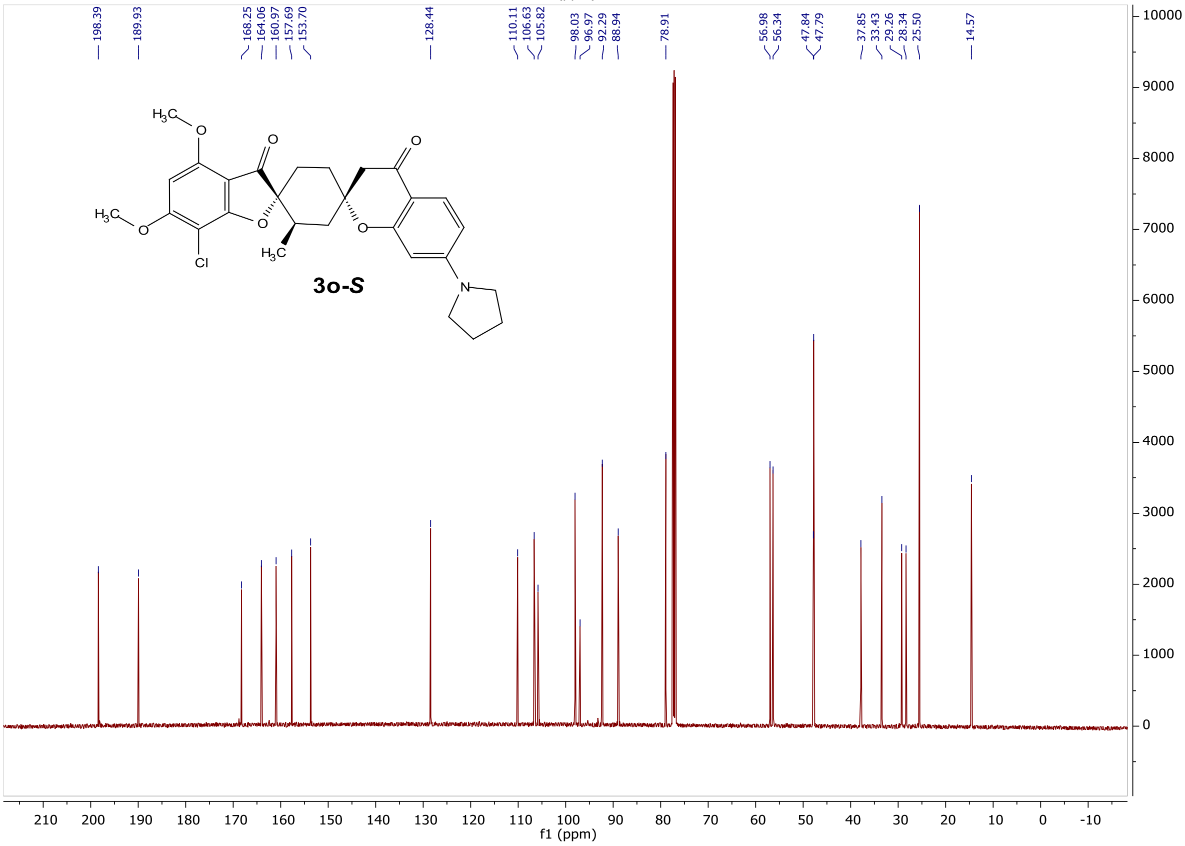
198.39 189.93 168.25 164.06 160.97 157.69 153.70 128.44 110.11 106.63 105.82 98.03 96.97 92.29 88.94 78.91 56.98 56.34 47.84 47.79 37.85 33.43 29.26 28.34 25.50 14.57
3o-S
f1 (ppm)

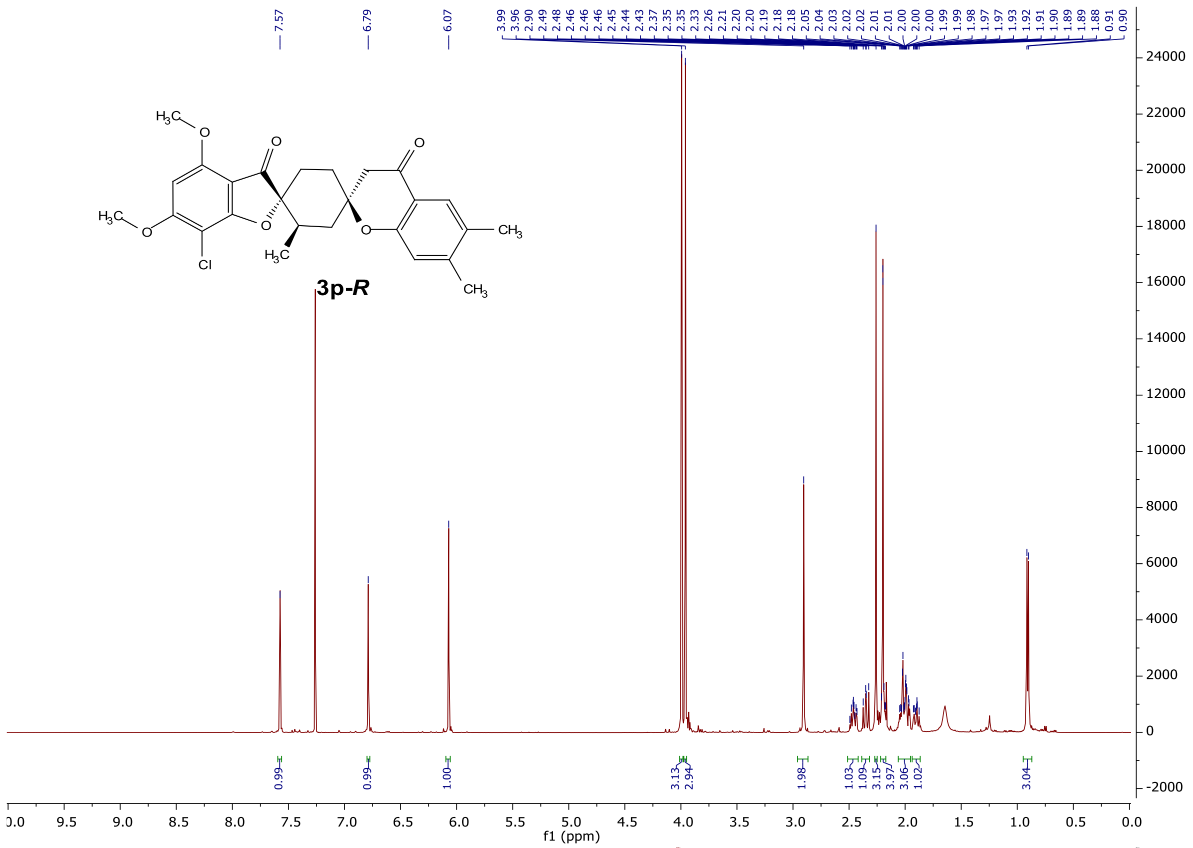
3p-R
f1 (ppm)

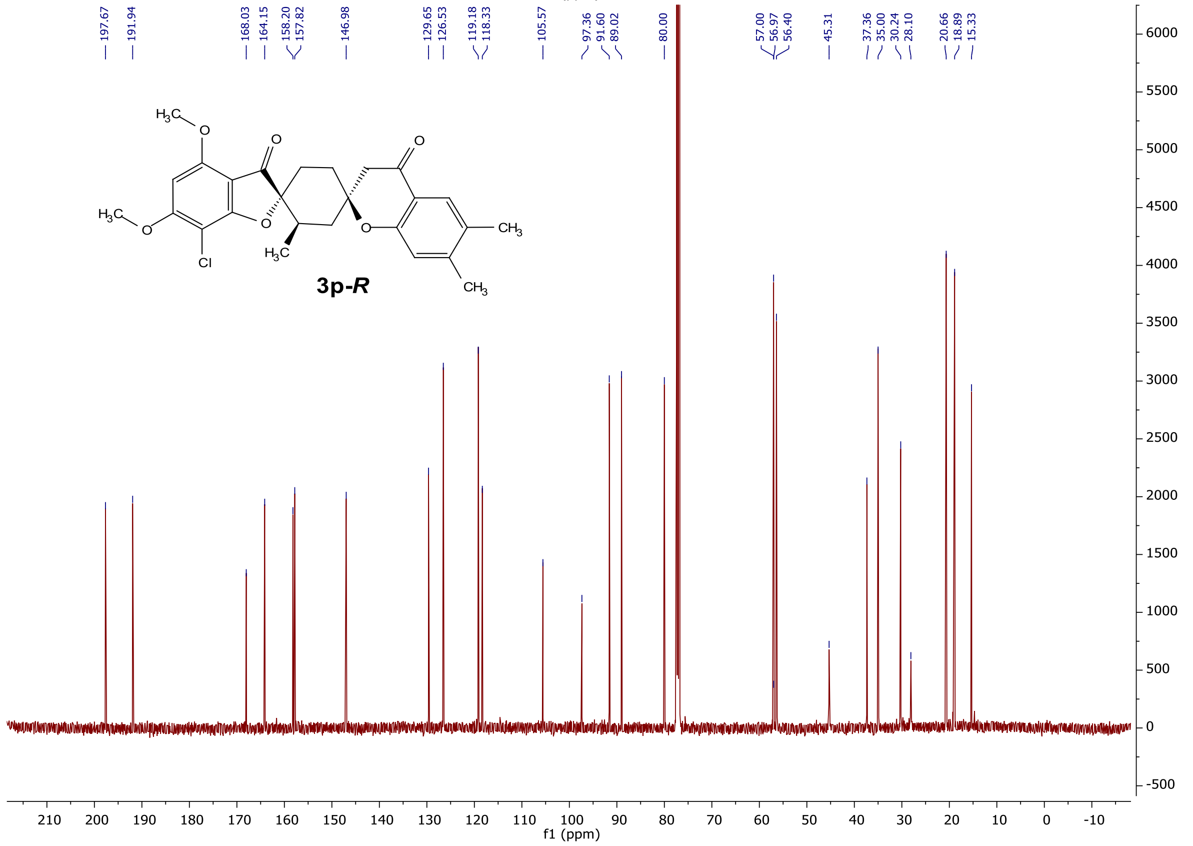
3p-R
f1 (ppm)

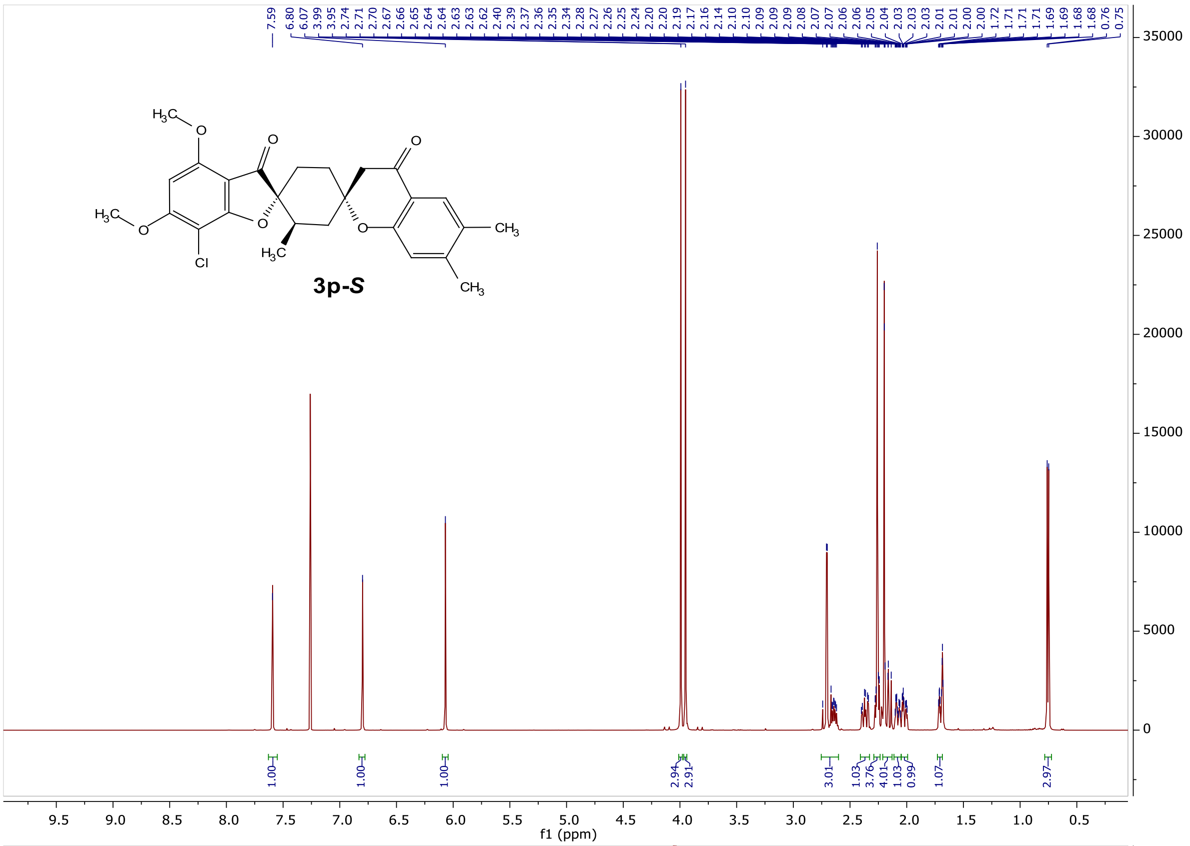

3p-S
7.59
6.80
6.07
3.99
3.95
1.00
1.00
1.00
2.94
2.91
3.01
1.03
3.76
4.01
1.03
0.99
1.07
2.97
f1 (ppm)

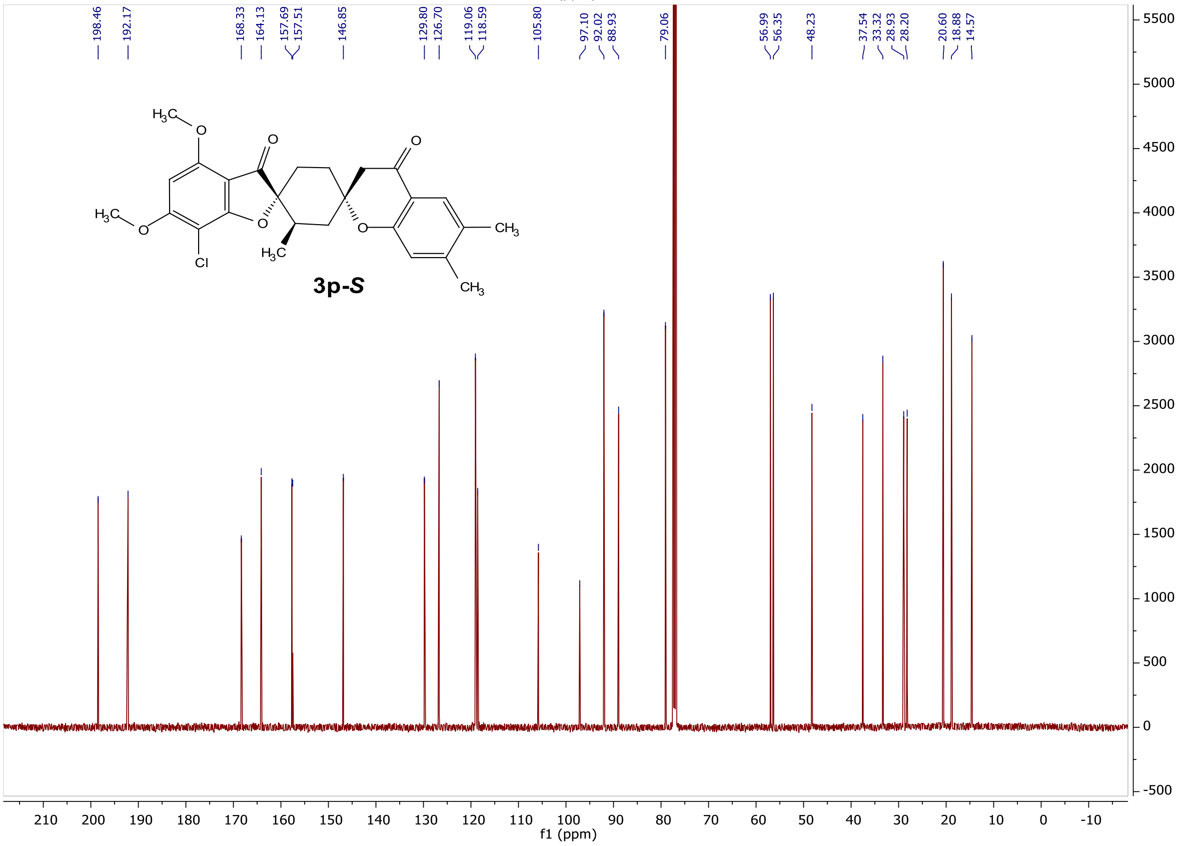

3p-S
198.46
192.17
168.33
164.13
157.69
157.51
146.85
129.80
126.70
119.06
118.59
105.80
97.10
92.02
88.93
79.06
56.99
56.35
48.23
37.54
33.32
28.93
28.20
20.60
18.88
14.57
f1 (ppm)

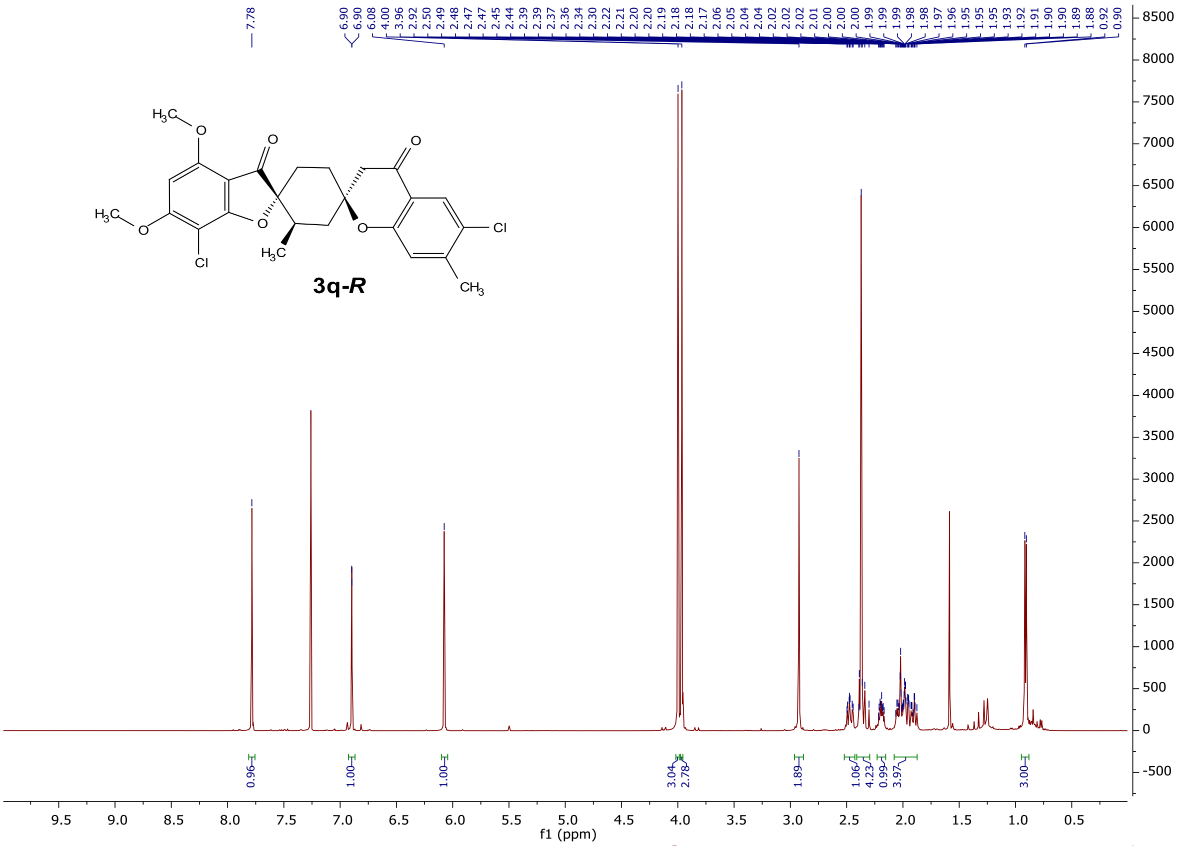

7.78
6.90 6.90 6.08 4.00 3.96 2.92 2.50 2.49 2.48 2.47 2.47 2.45 2.44 2.39 2.39 2.37 2.36 2.34 2.30 2.22 2.21 2.20 2.20 2.20 2.19 2.18 2.18 2.17 2.06 2.05 2.04 2.04 2.02 2.02 2.02 2.01 2.00 2.00 2.00 1.99 1.99 1.99 1.98 1.98 1.97 1.96 1.95 1.95 1.95 1.93 1.92 1.91 1.90 1.90 1.89 1.88 0.92 0.90
3q-R
0.96 1.00 1.00 3.04 2.78 1.89 1.06 4.23 0.99 3.97 3.00
f1 (ppm)

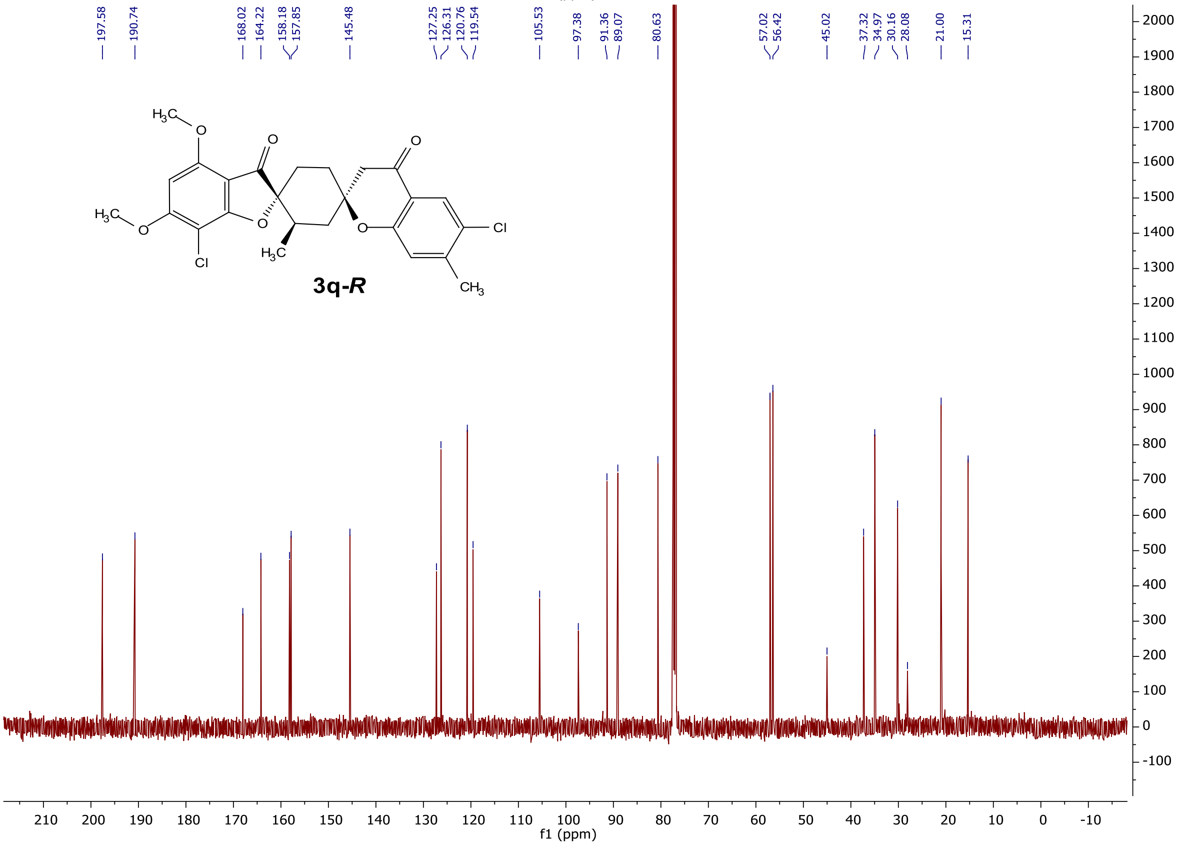

197.58 190.74 168.02 164.22 158.18 157.85 145.48 127.25 126.31 120.76 119.54 105.53 97.38 91.36 89.07 80.63 57.02 56.42 45.02 37.32 34.97 30.16 28.08 21.00 15.31
3q-R
f1 (ppm)

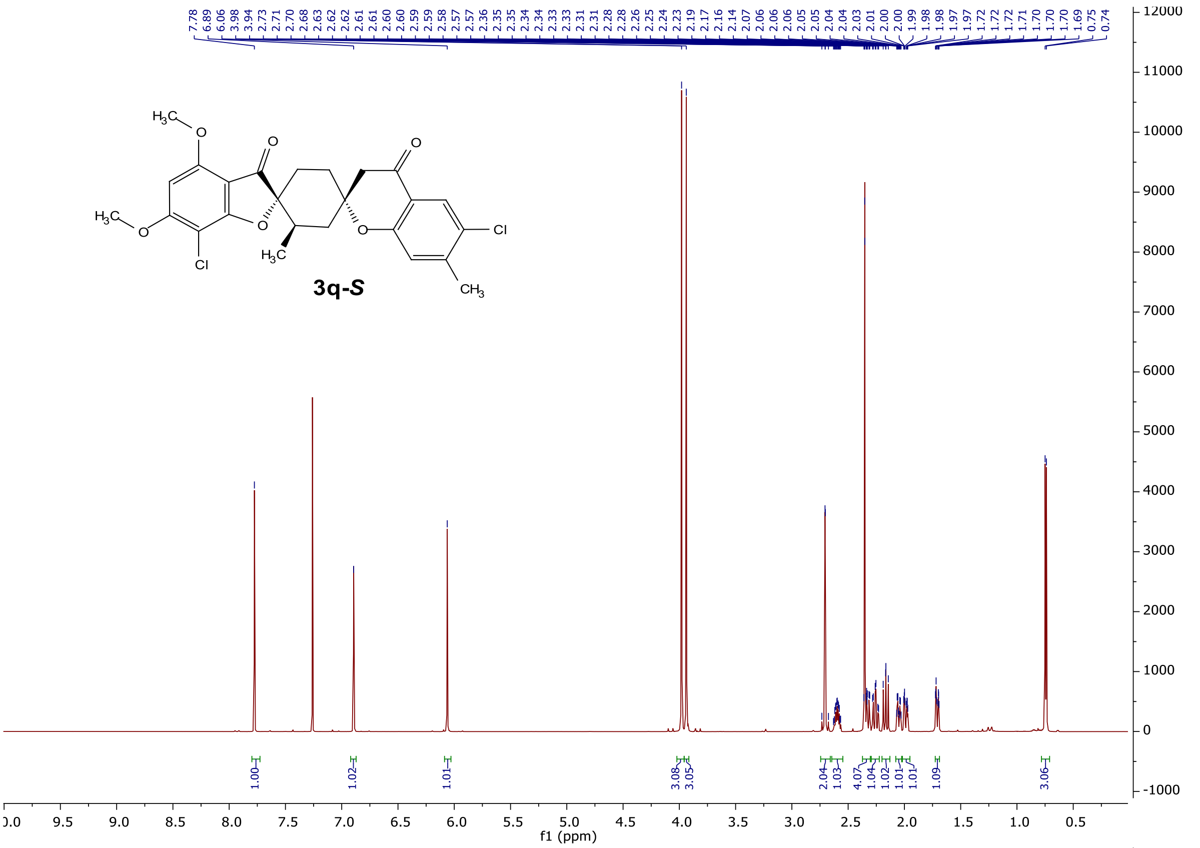
3q-S

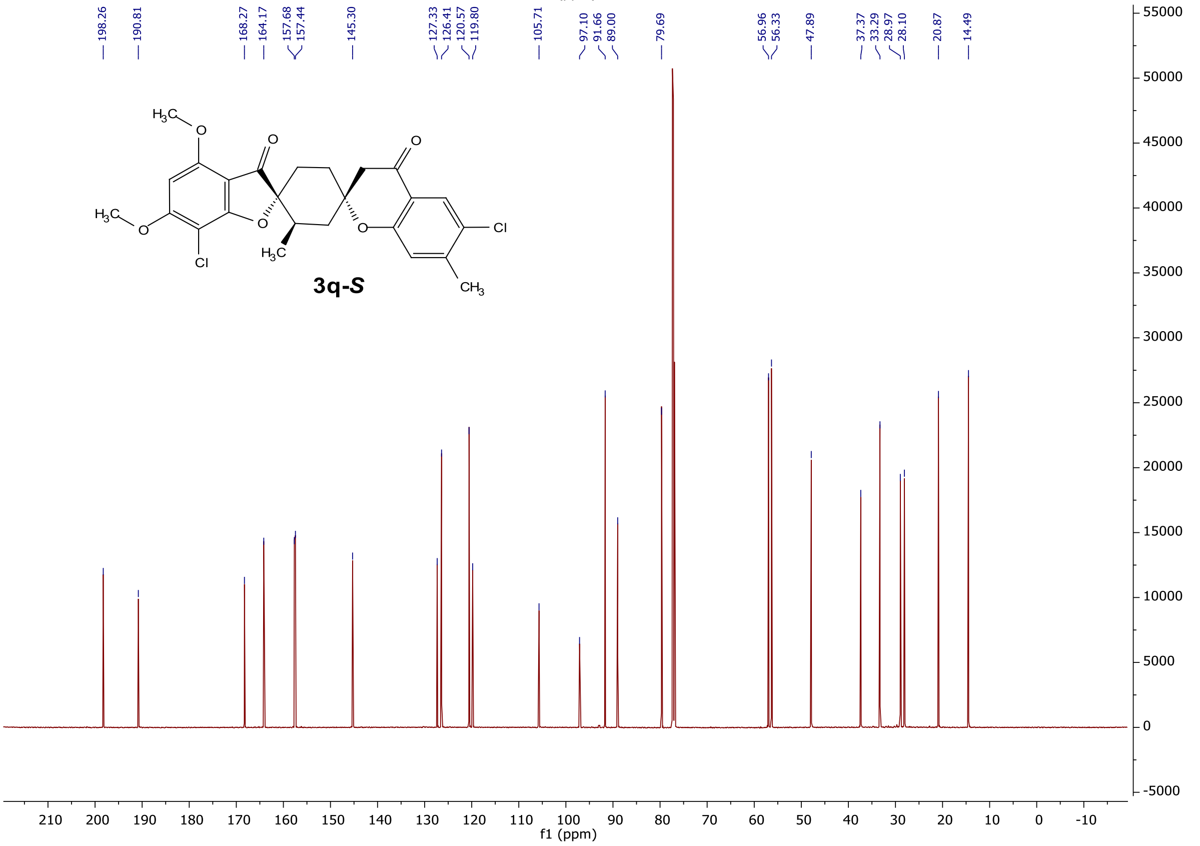
3q-S

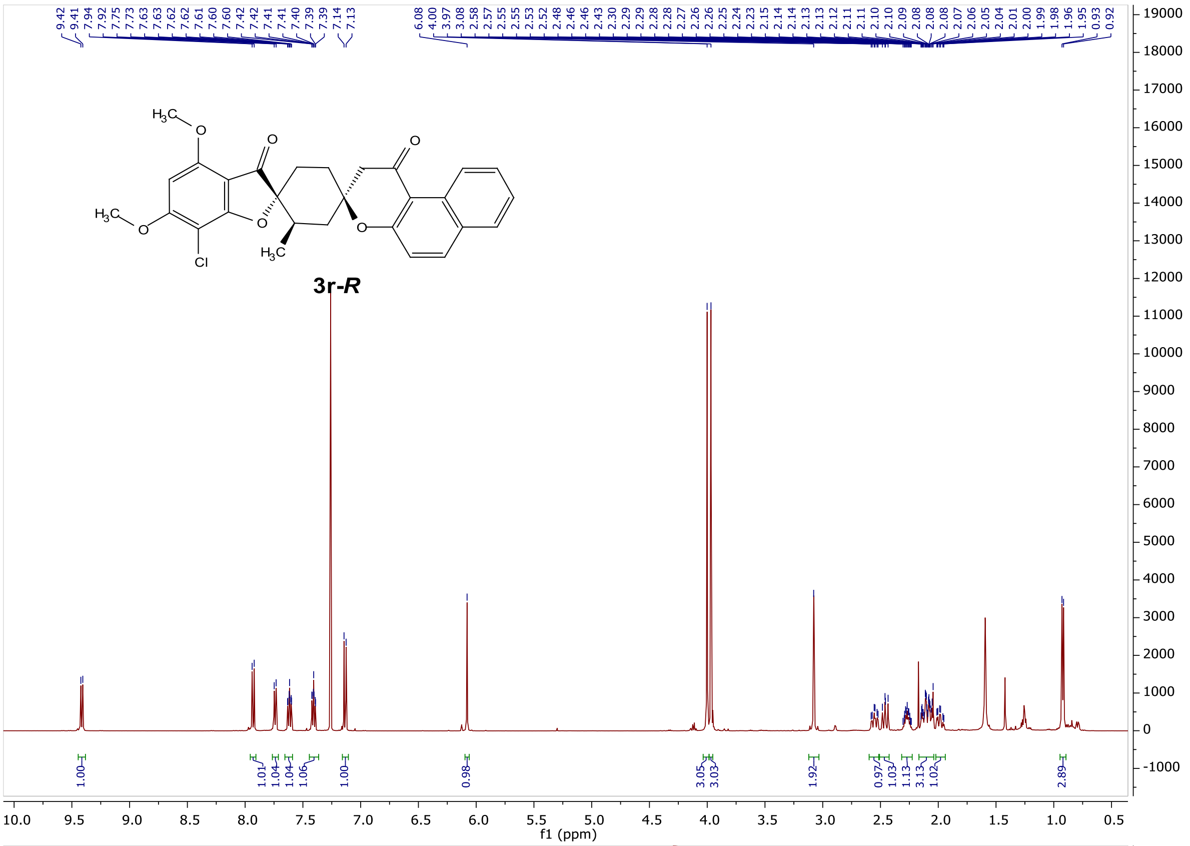
3r-*R*
f1 (ppm)

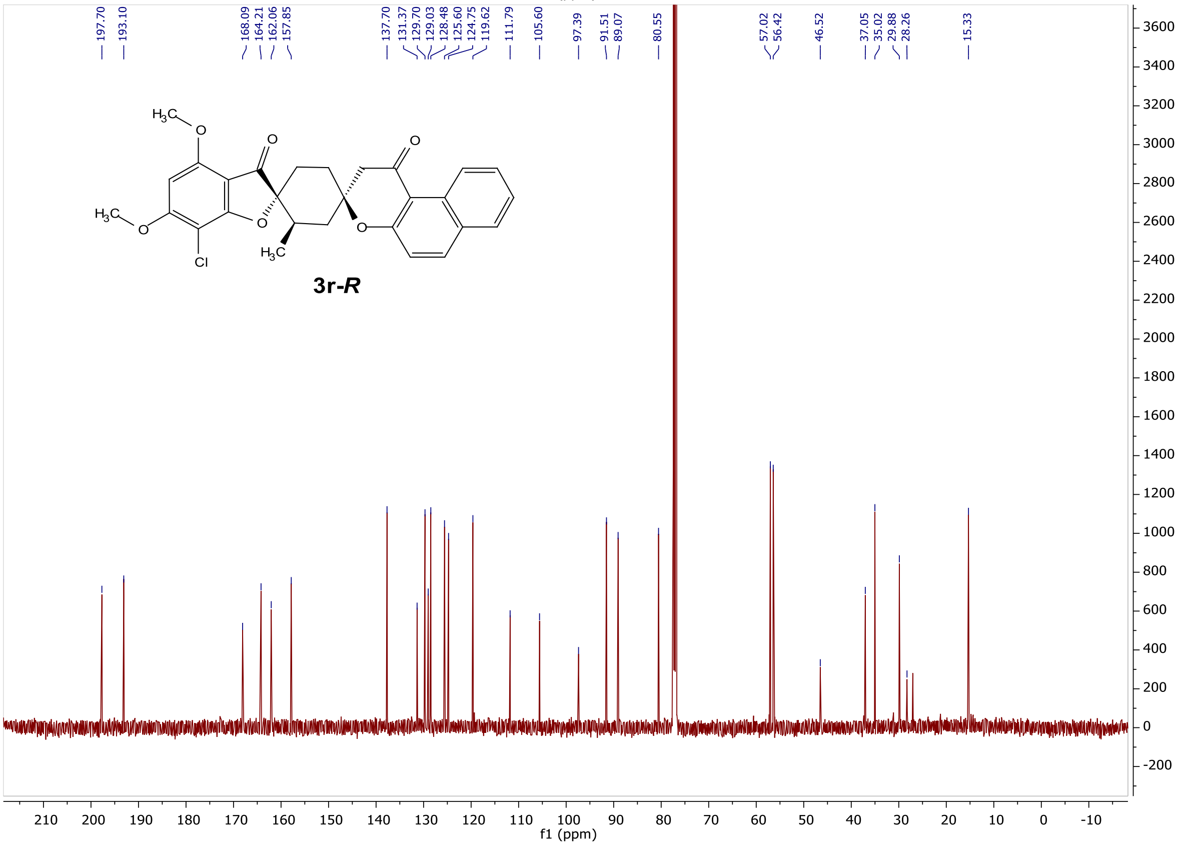
3r-*R*
f1 (ppm)

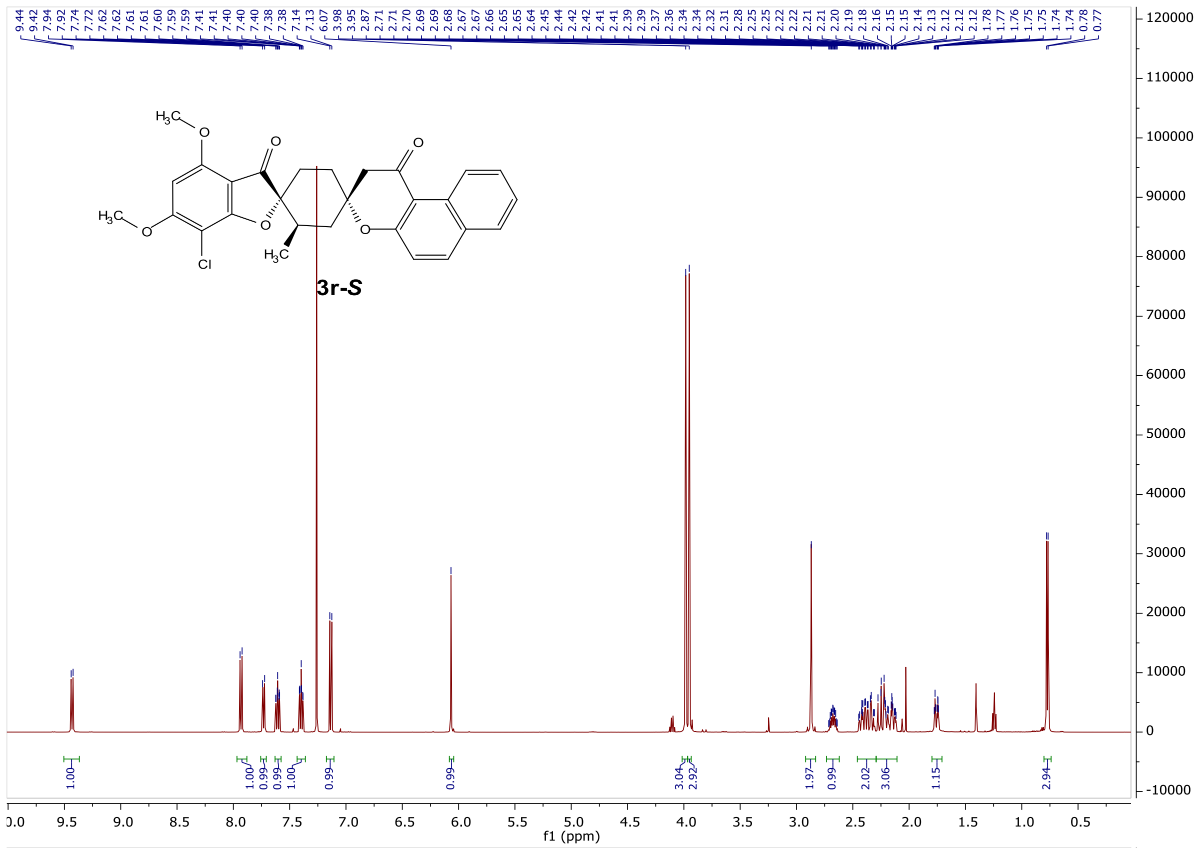

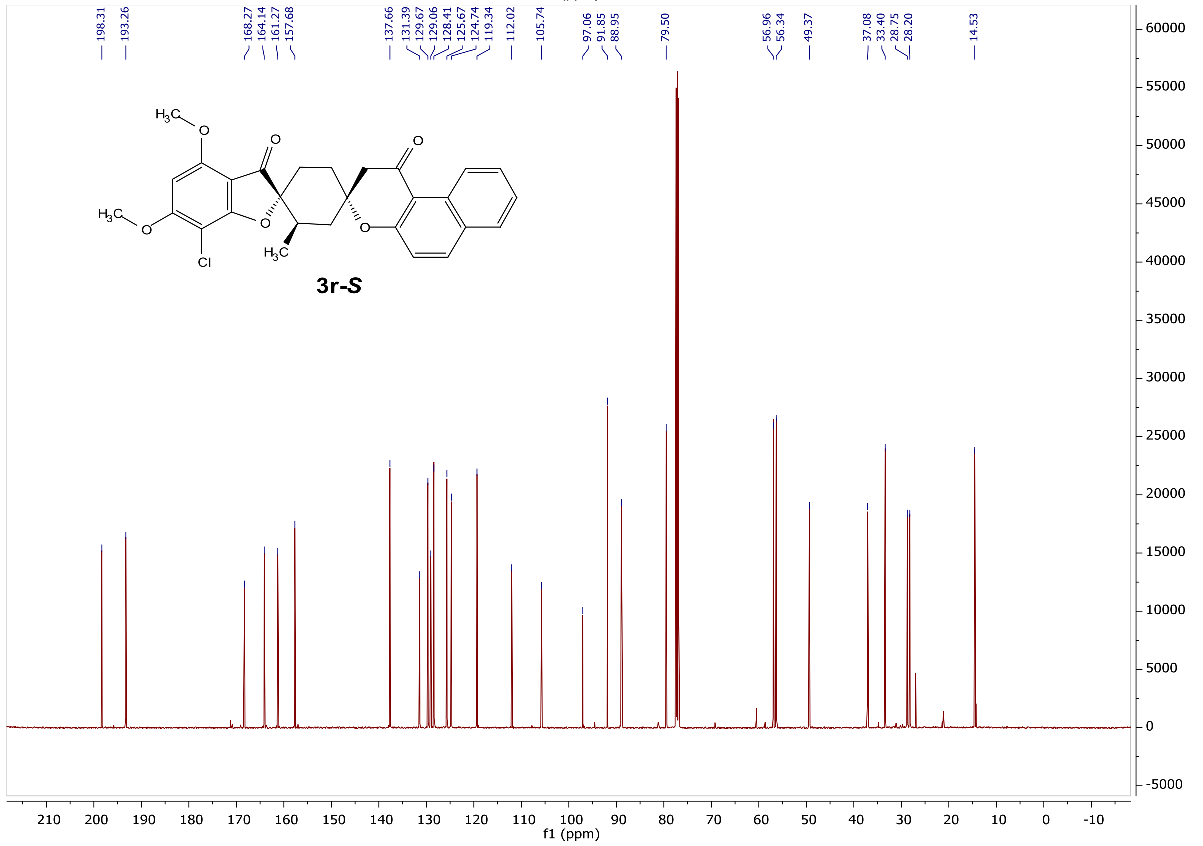

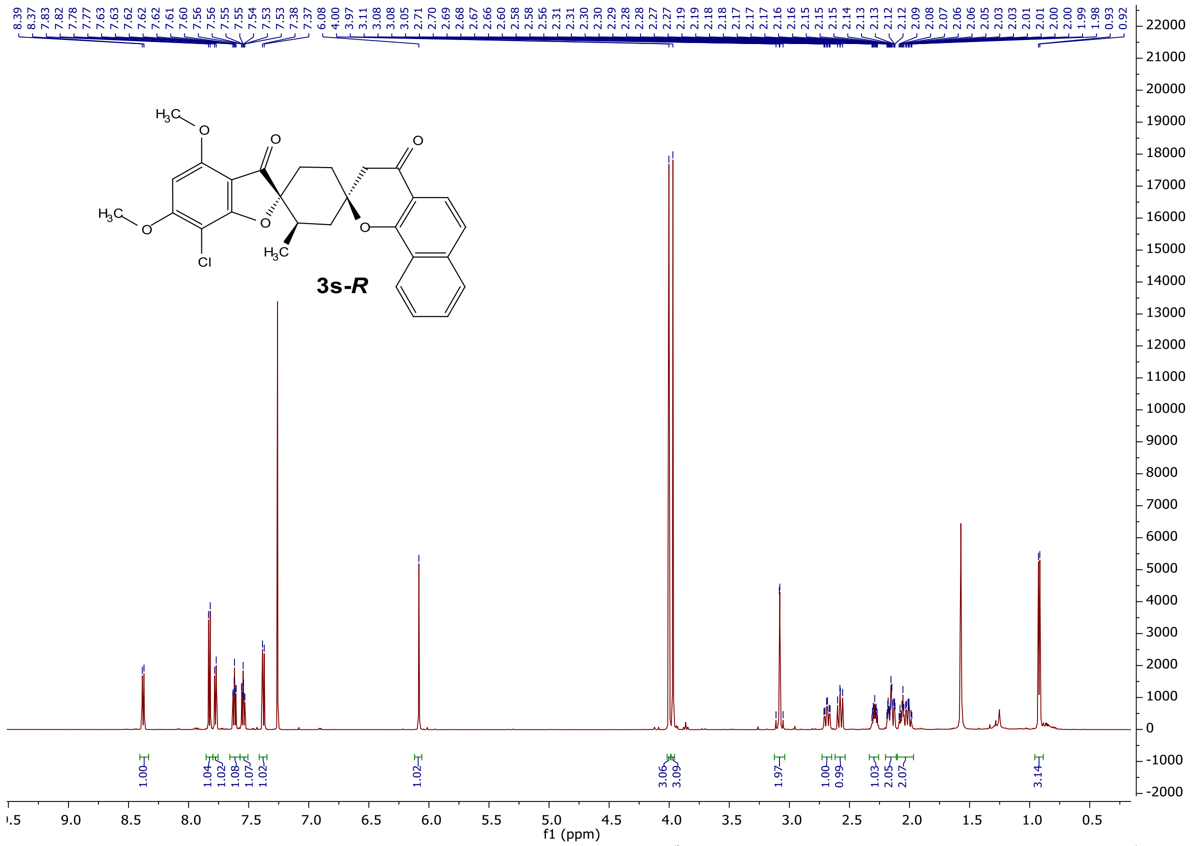

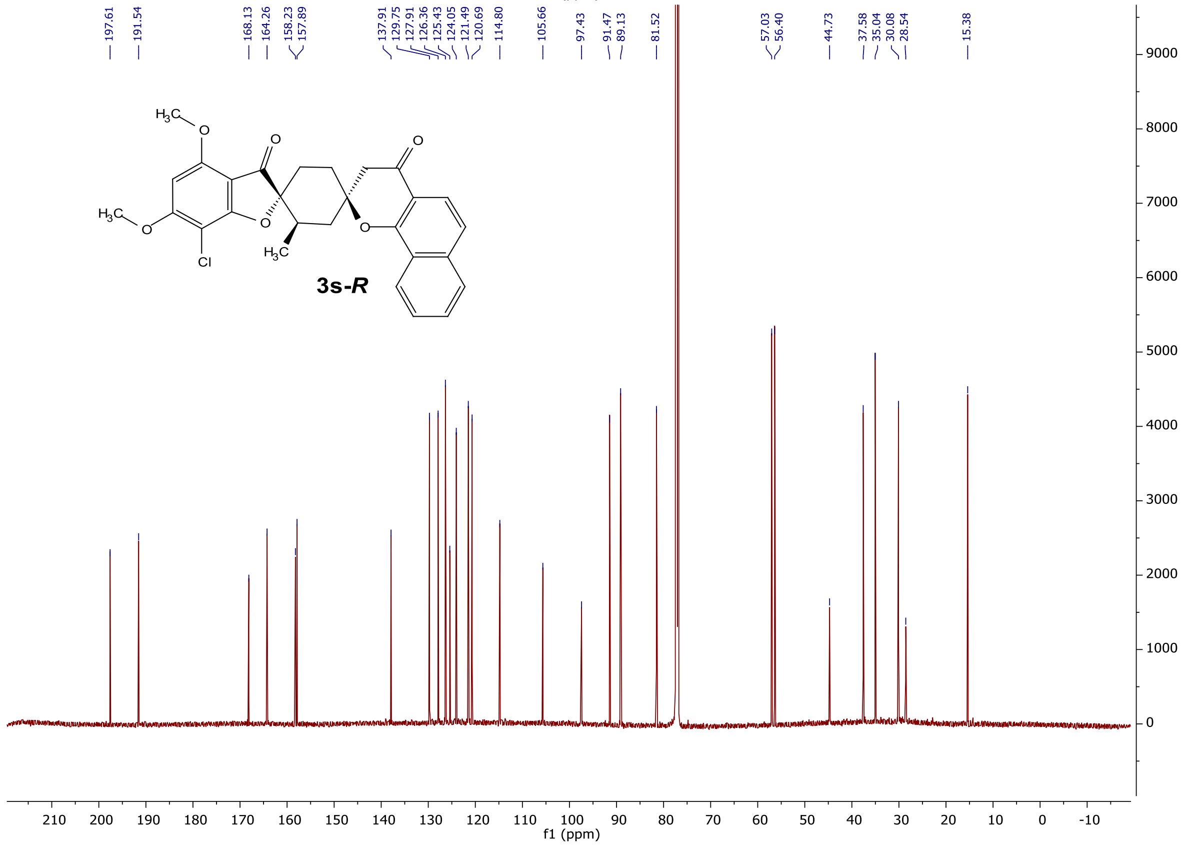

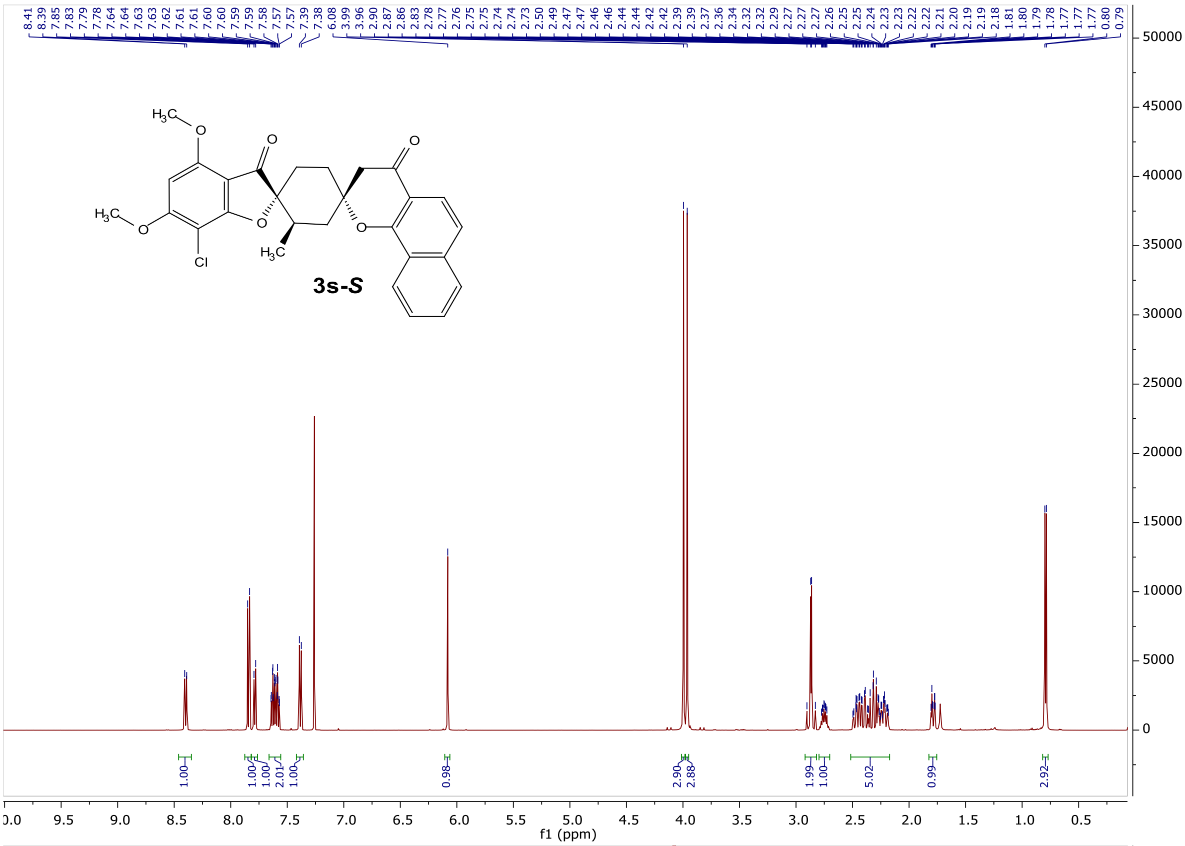
8.41 8.39 7.85 7.83 7.79 7.78 7.64 7.64 7.63 7.62 7.61 7.61 7.60 7.60 7.59 7.59 7.58 7.57 7.57 7.39 7.38 6.08 3.99 3.96 2.90 2.87 2.86 2.83 2.78 2.77 2.76 2.75 2.75 2.74 2.74 2.73 2.50 2.49 2.47 2.47 2.46 2.44 2.44 2.42 2.42 2.39 2.39 2.37 2.36 2.34 2.32 2.32 2.29 2.27 2.27 2.27 2.26 2.25 2.25 2.24 2.23 2.23 2.22 2.22 2.21 2.20 2.19 2.19 2.18 1.81 1.80 1.79 1.78 1.77 1.77 0.80 0.79
1.00 1.00 1.00 2.01 1.00 0.98 2.90 2.88 1.99 1.00 5.02 0.99 2.92
3s-S
f1 (ppm)

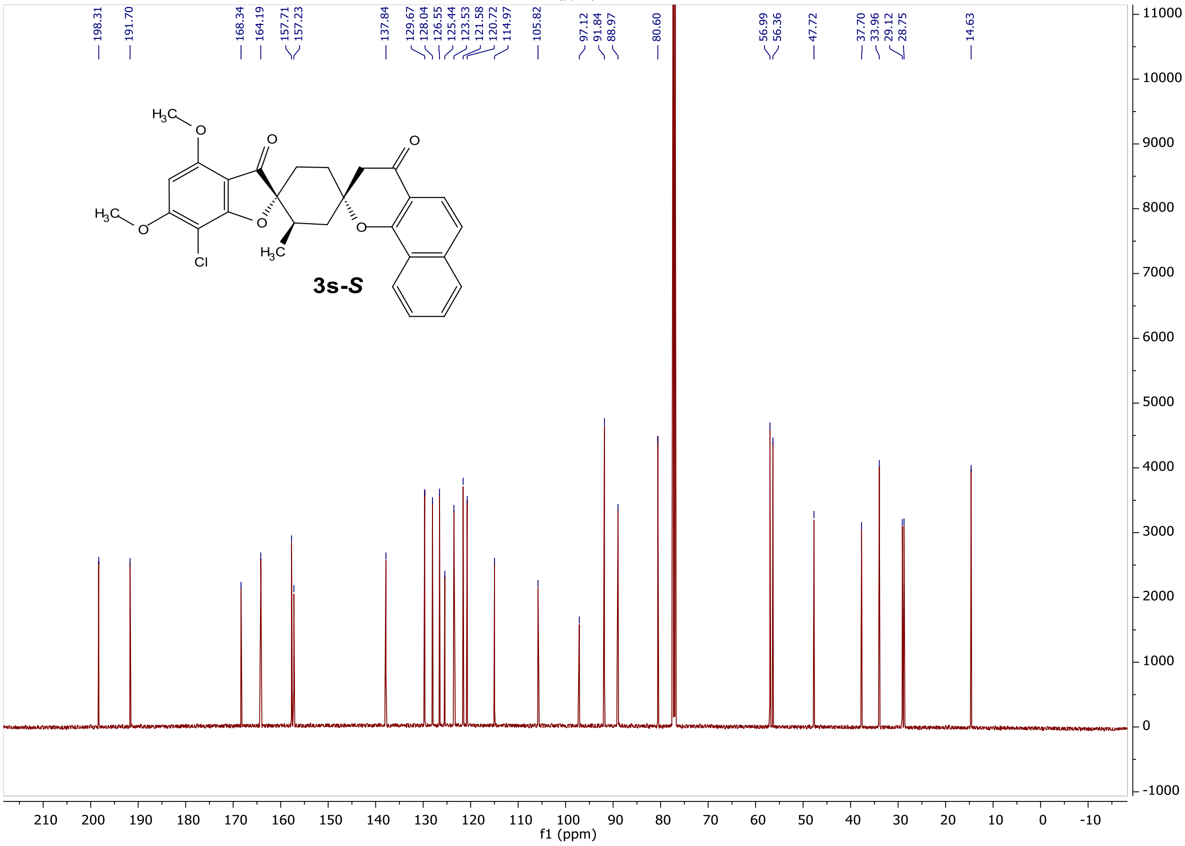
198.31 191.70 168.34 164.19 157.71 157.23 137.84 129.67 128.04 126.55 125.44 123.53 121.58 120.72 114.97 105.82 97.12 91.84 88.97 80.60 56.99 56.36 47.72 37.70 33.96 29.12 28.75 14.63
3s-S
f1 (ppm)

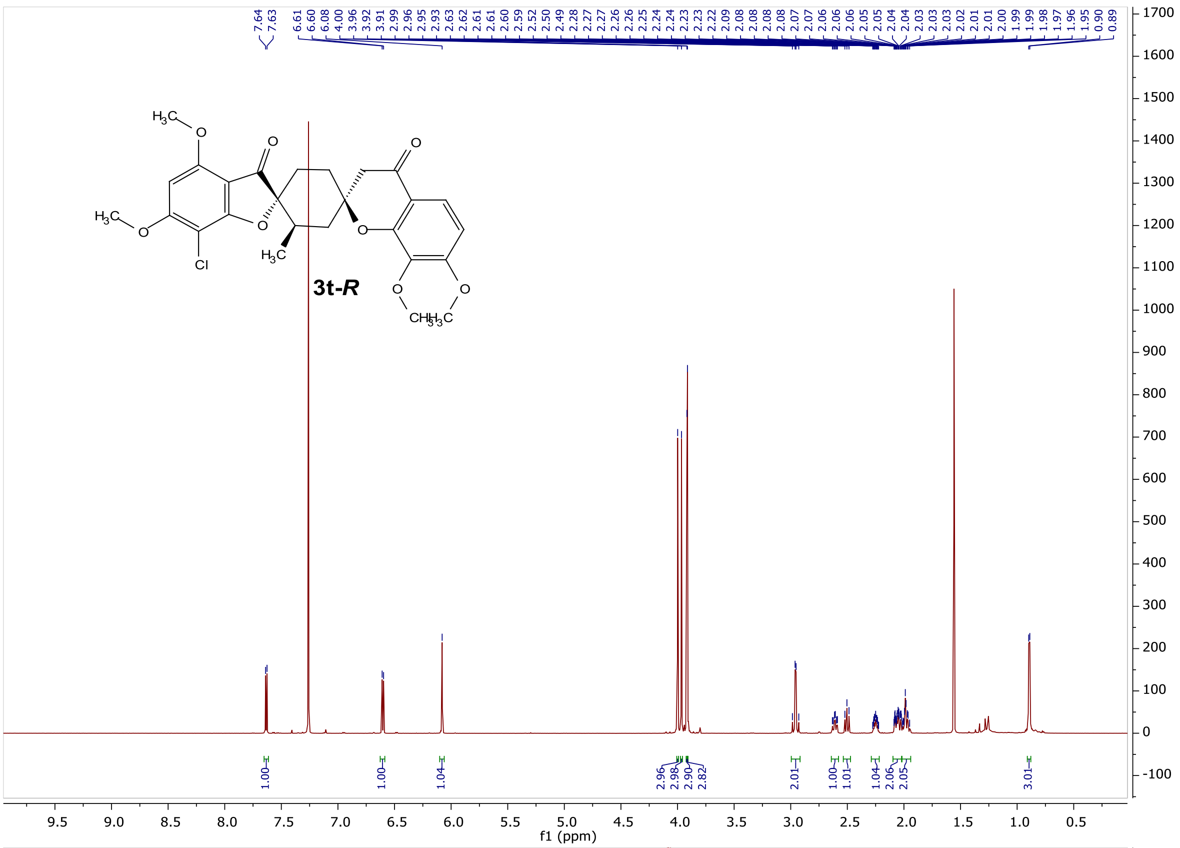
3t-*R*
f1 (ppm)

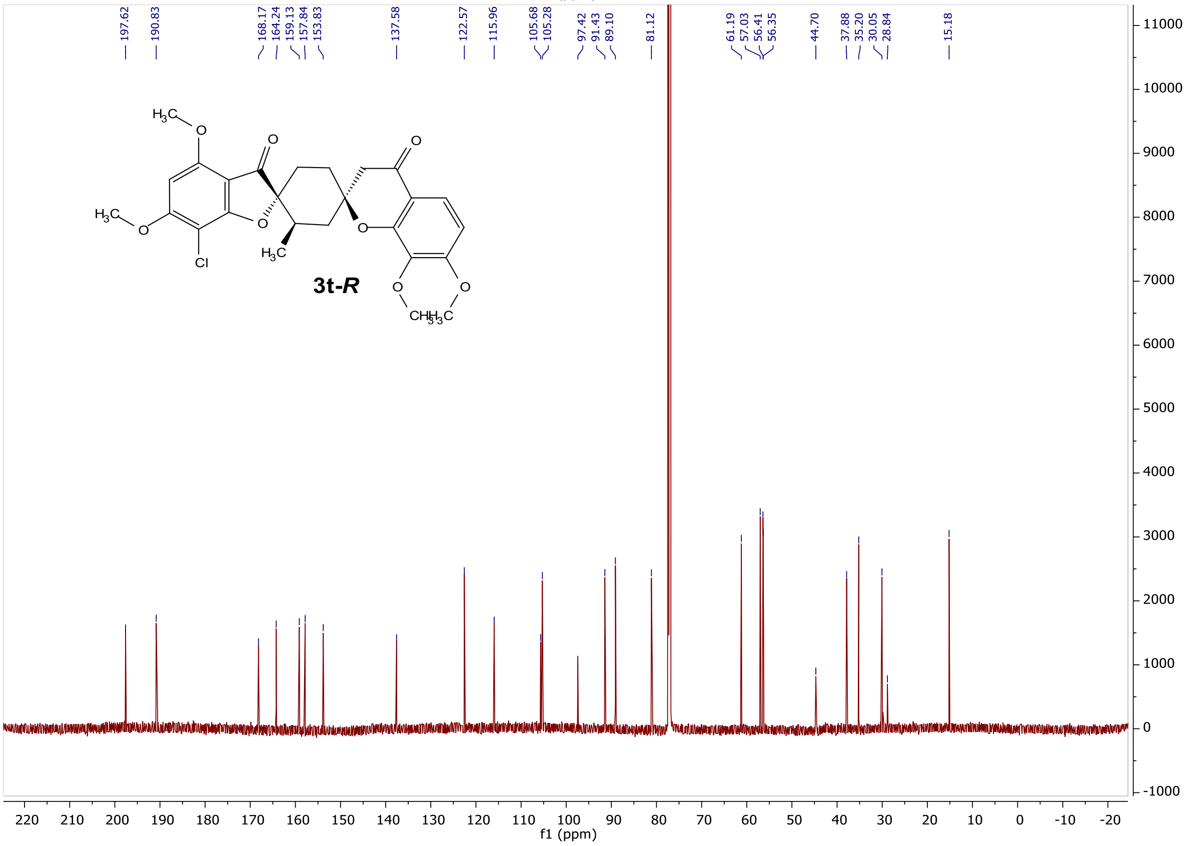
3t-*R*
f1 (ppm)

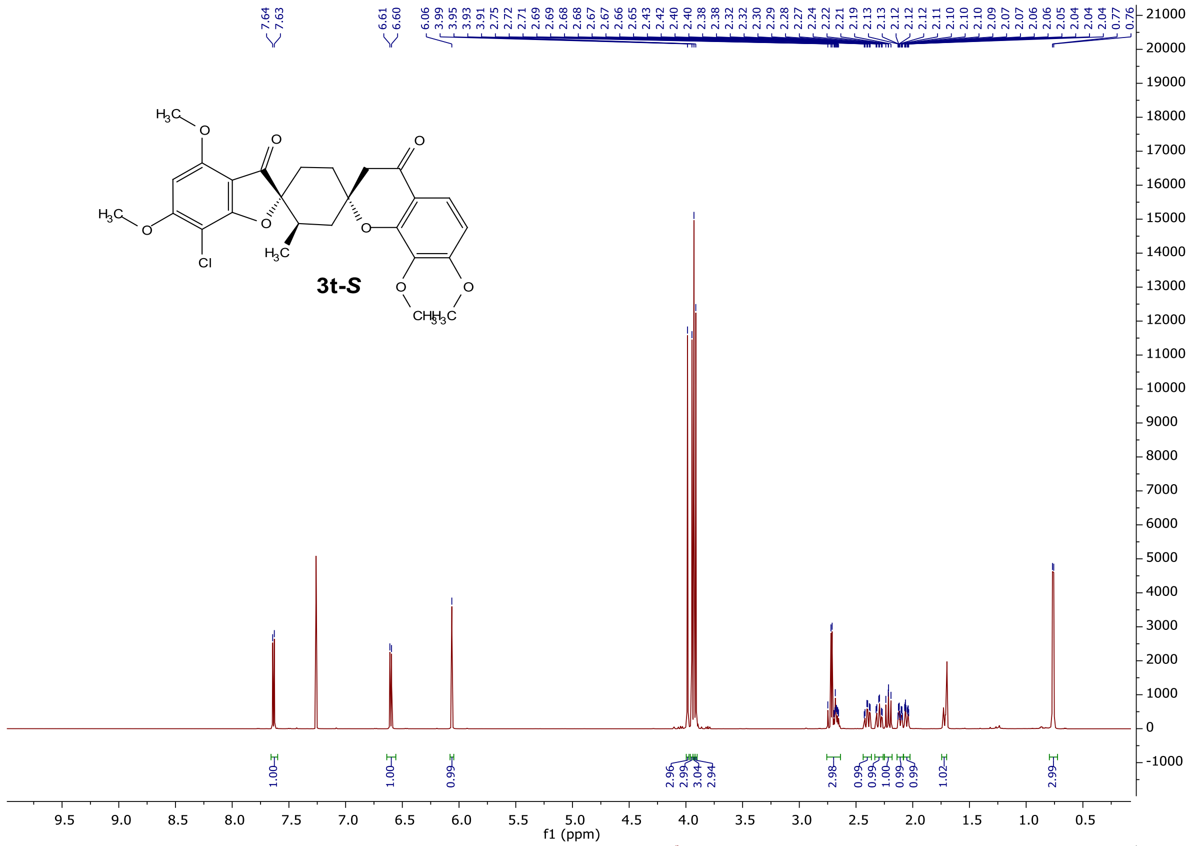
3t-S
f1 (ppm)

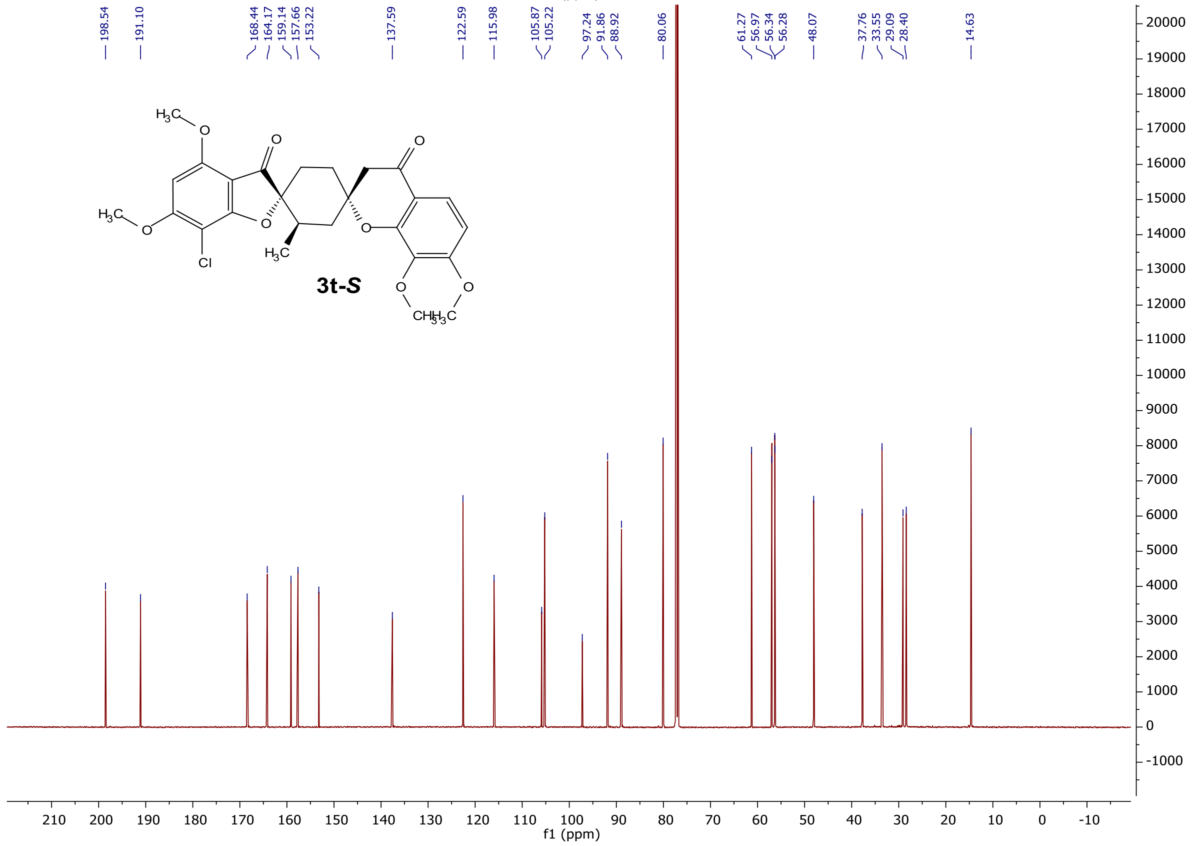
3t-S
f1 (ppm)

Purity Spectra of Griseofulvin-Chromanones

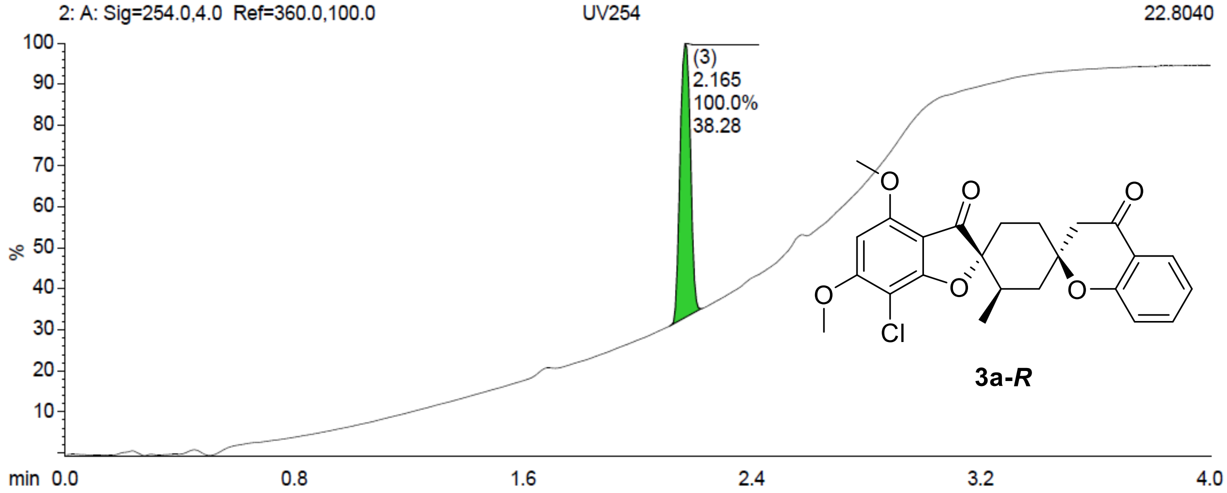

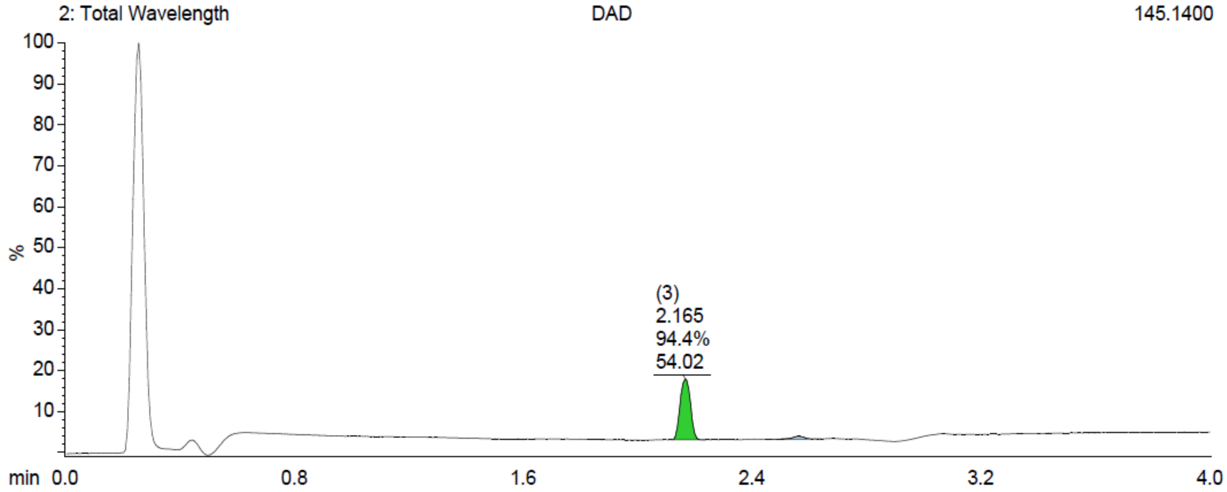

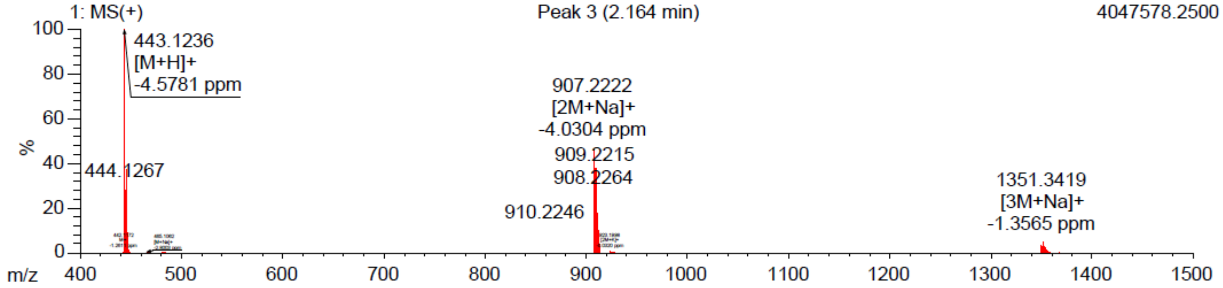

BPM	Error PPM	Error mDa	Target
443.1236	9.8294	13.4399	442.1…

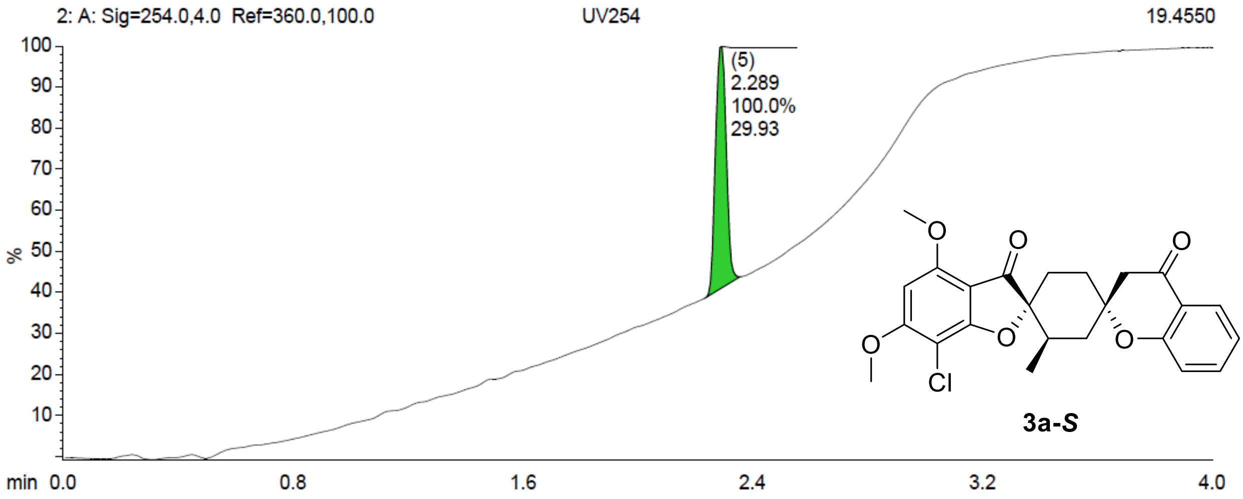

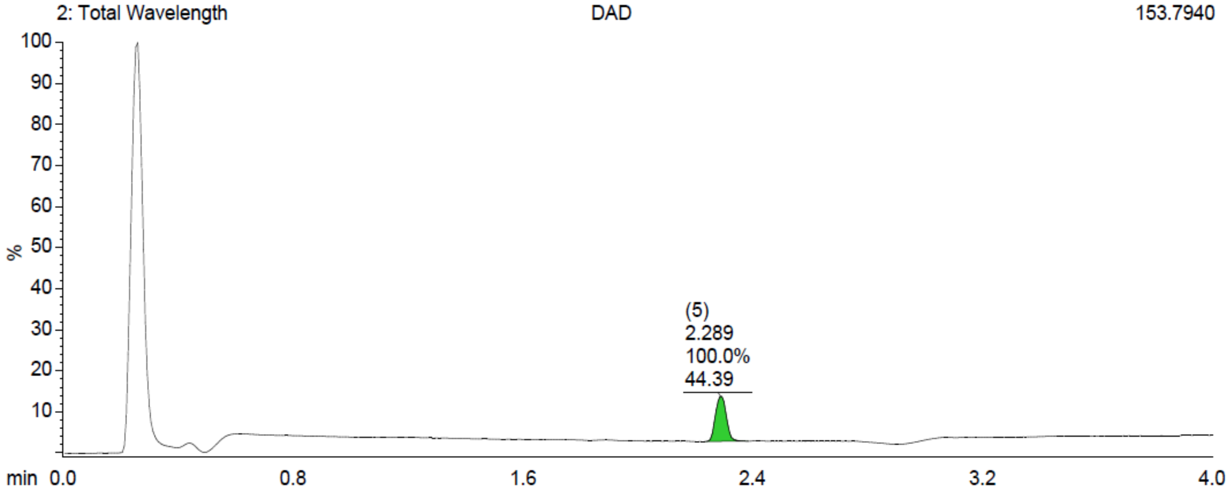

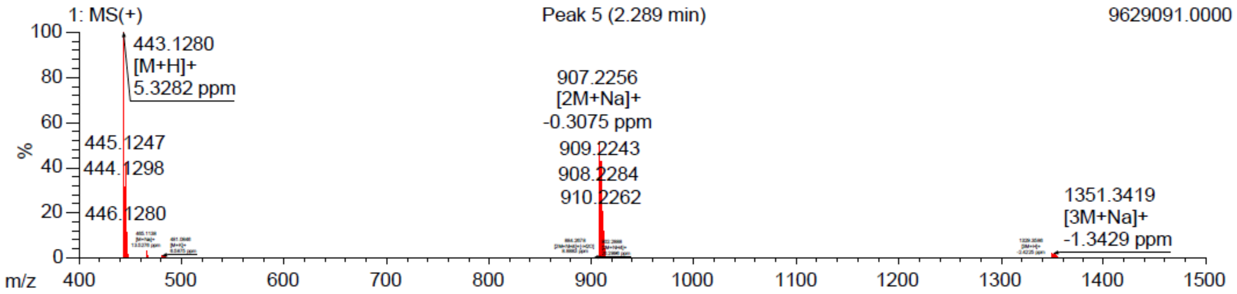

BPM	Error PPM	Error mDa	Target
443.1280	-1.3429	-1.8148	443.1…

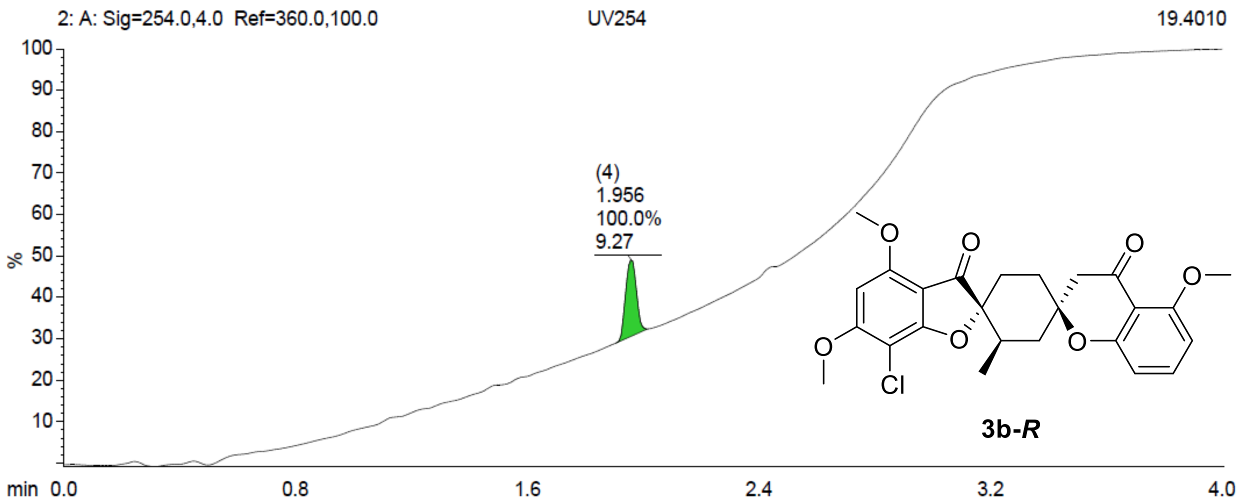

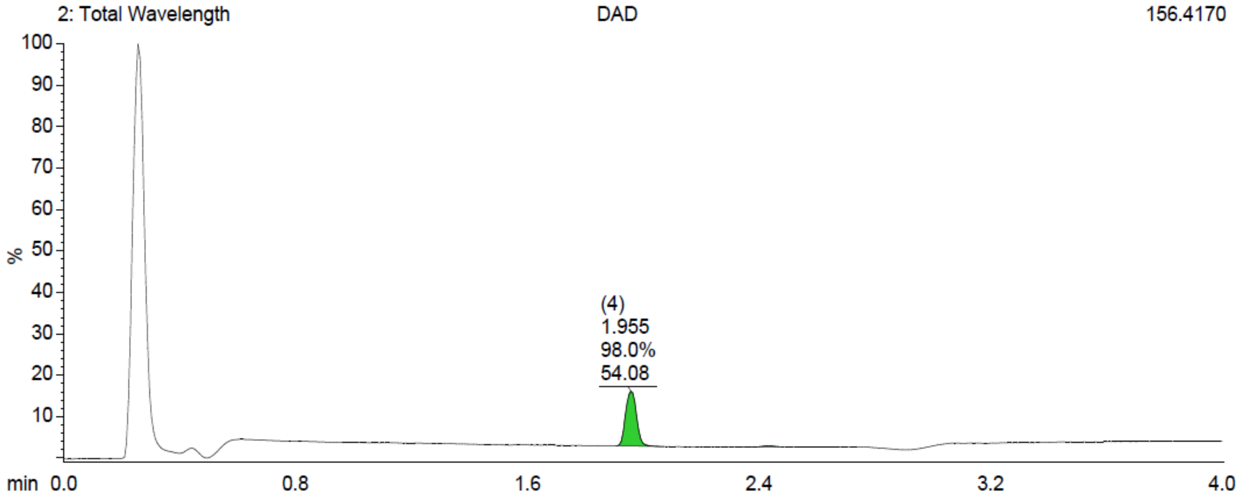

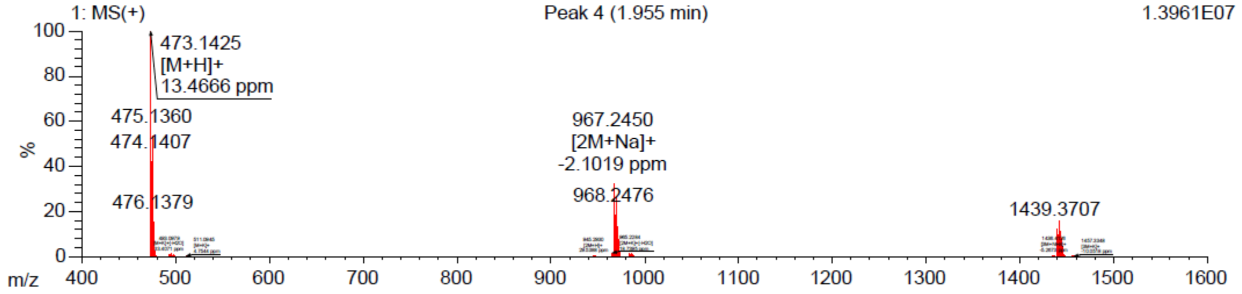

BPM	Error PPM	Error mDa	Target
473.1425	-10.0518	-14.6491	473.1…

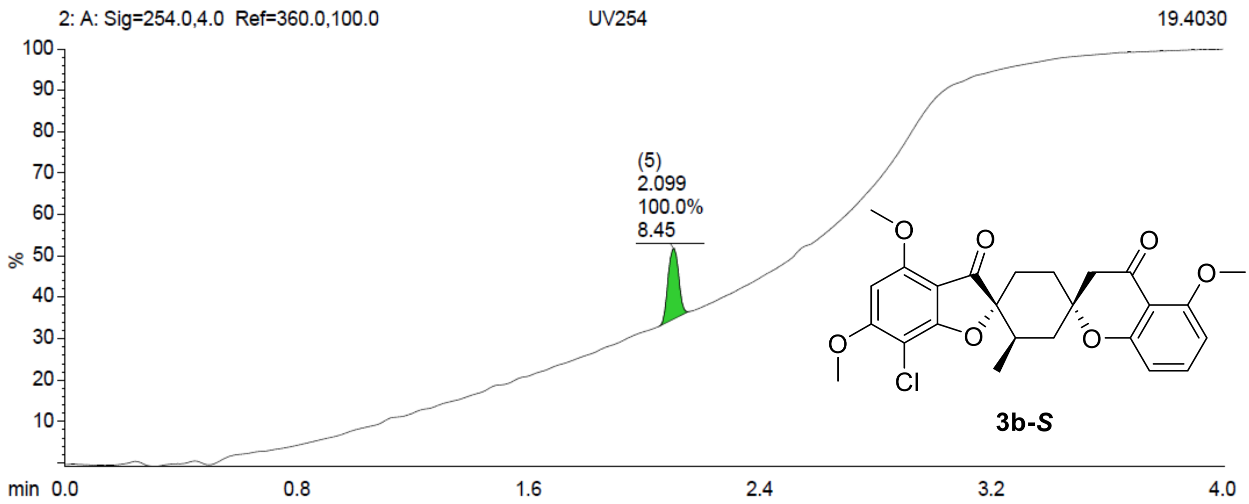

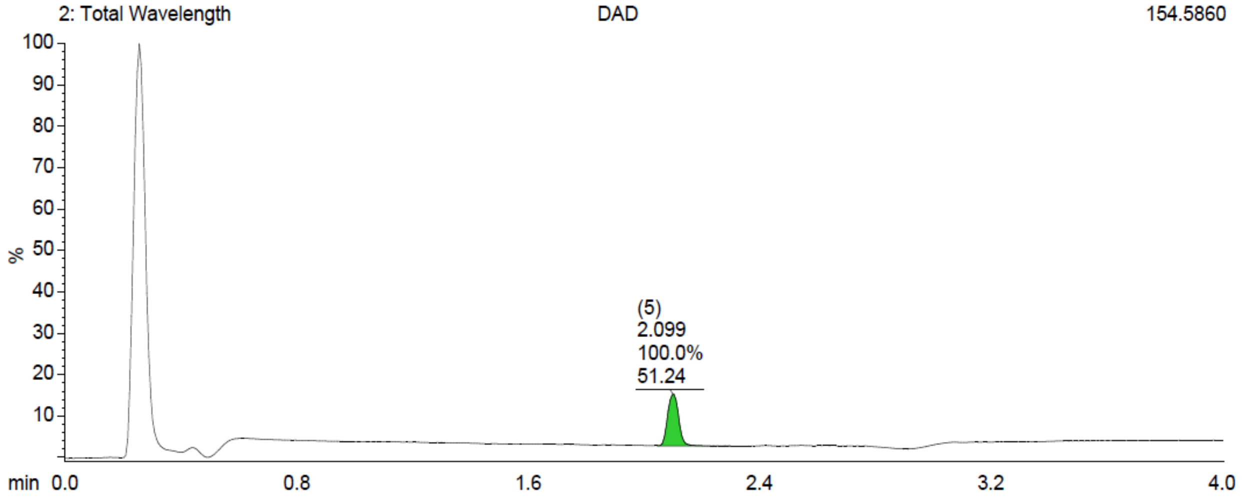

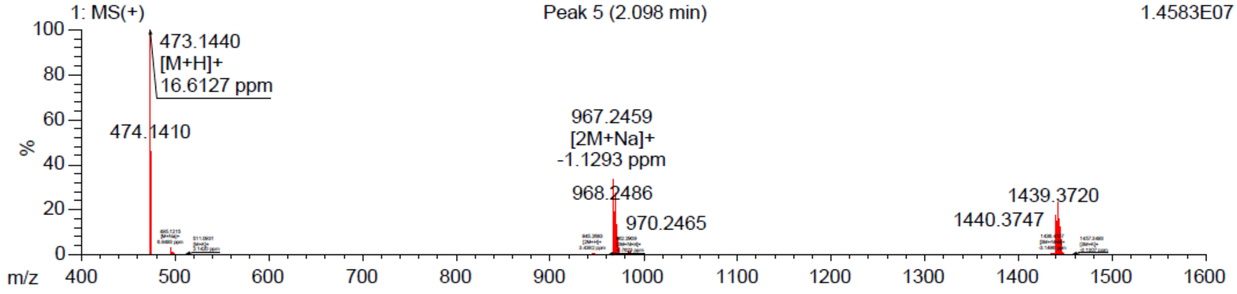

BPM	Error PPM	Error mDa	Target
473.1440	-0.1307	-0.1904	473.1…

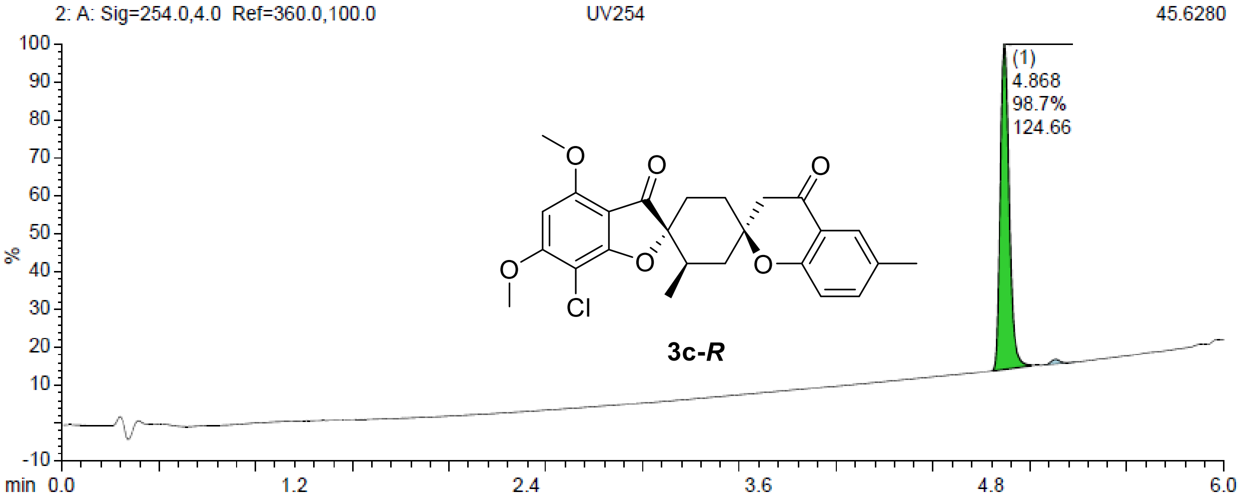

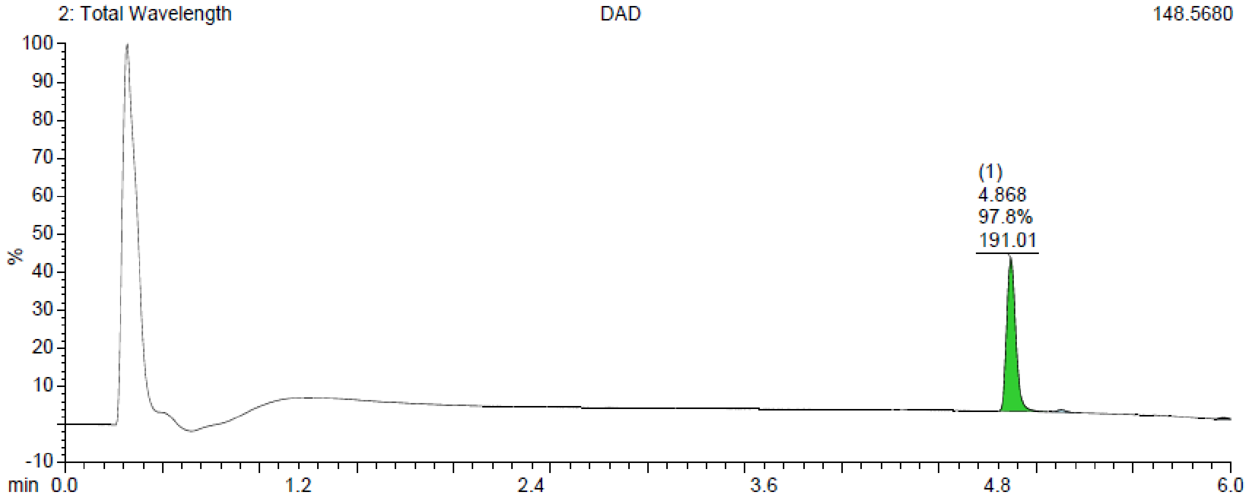

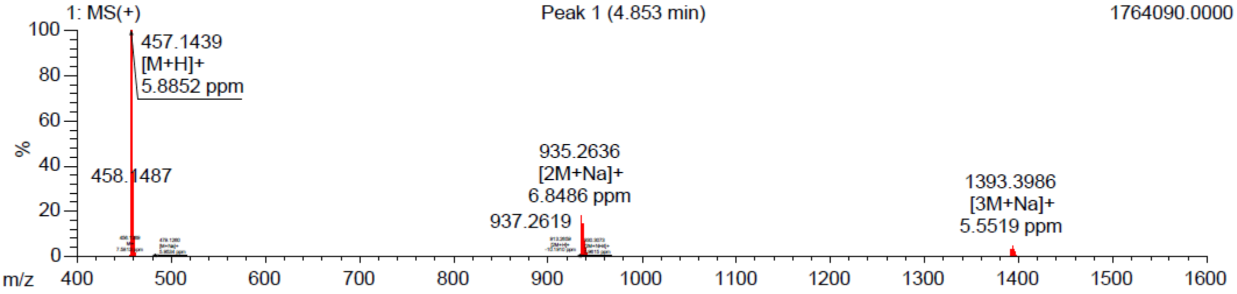

BPM	Error PPM	Error mDa	Target
457.1439	-1.7182	-2.4216	456.1…

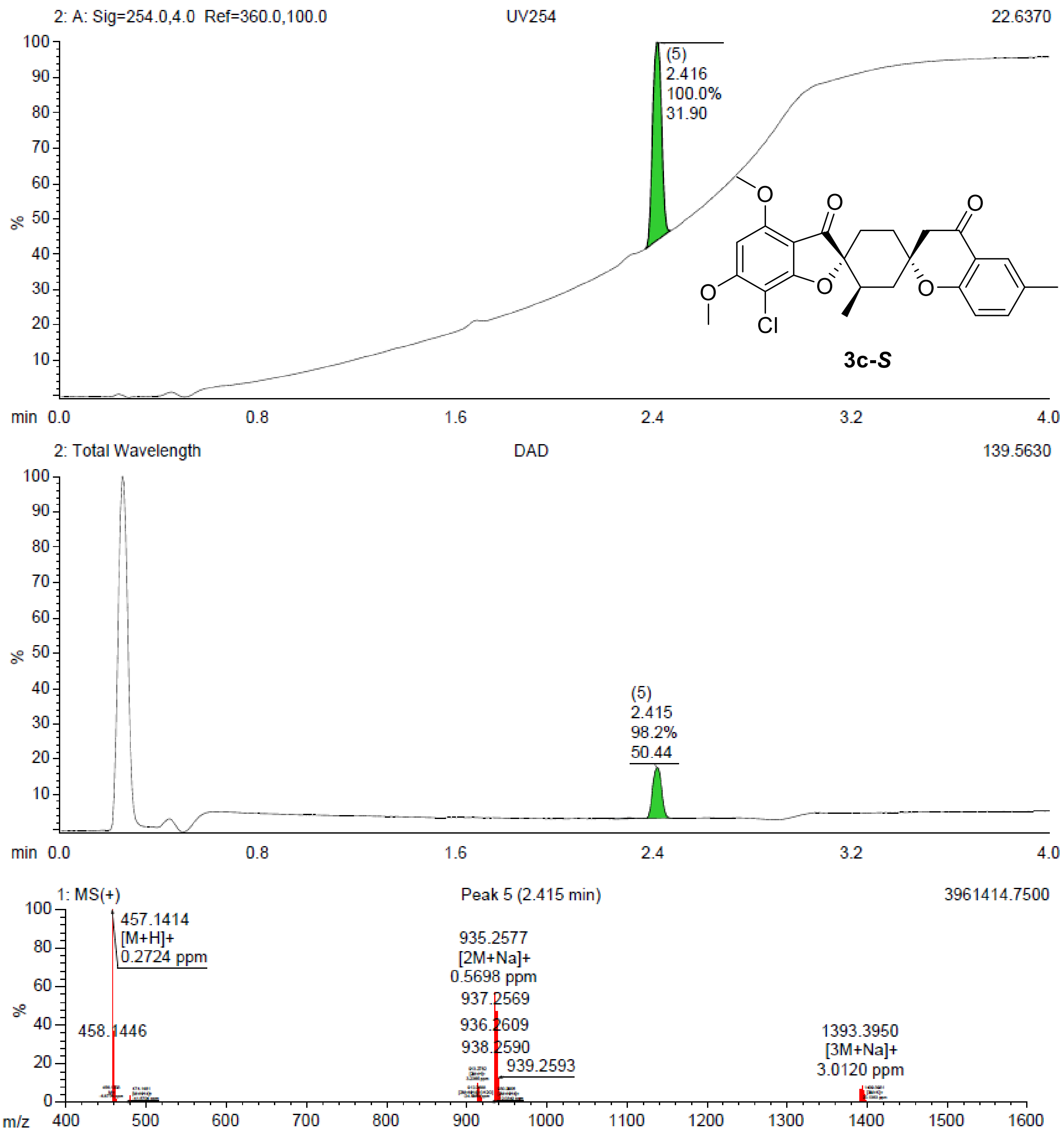

BPM	Error PPM	Error mDa	Target
457.1414	20.1363	28.3794	456.1…

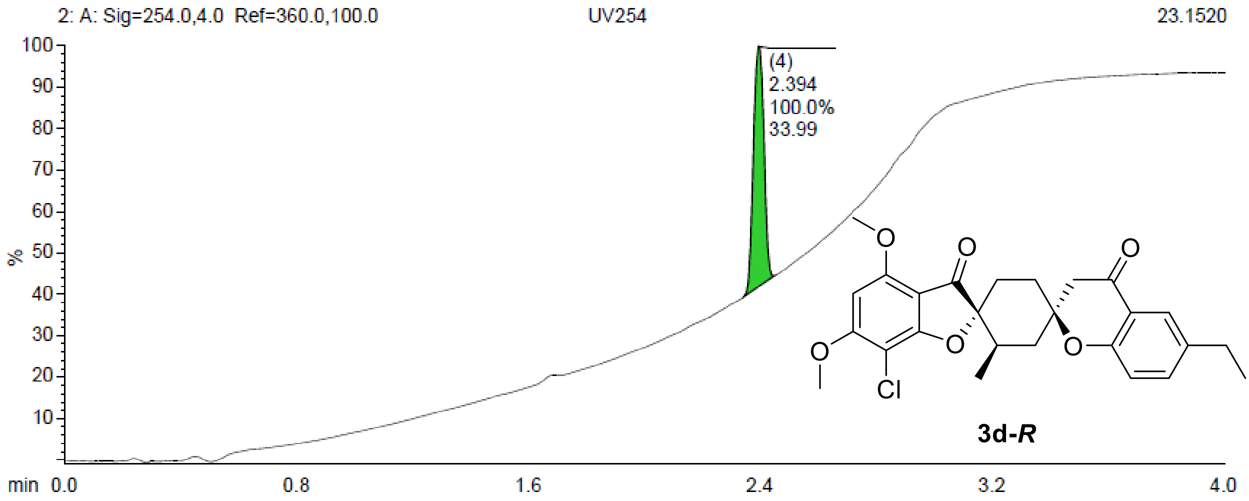

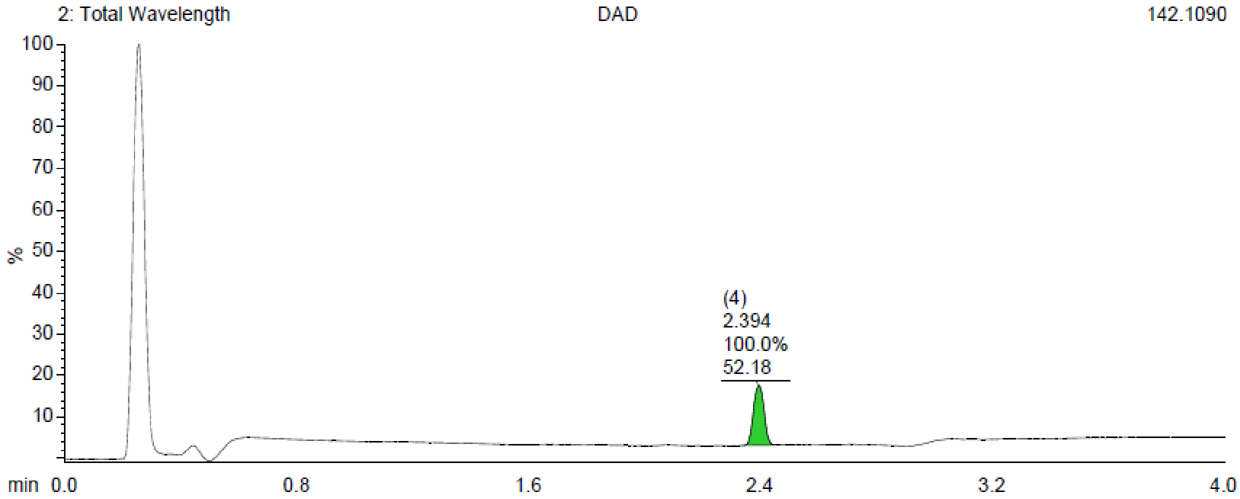

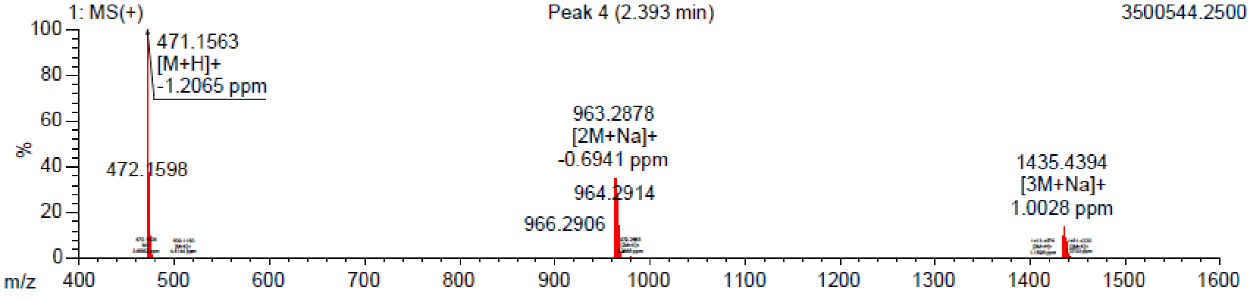

BPM	Error PPM	Error mDa	Target
471.1563	7.0733	10.2662	470.1…

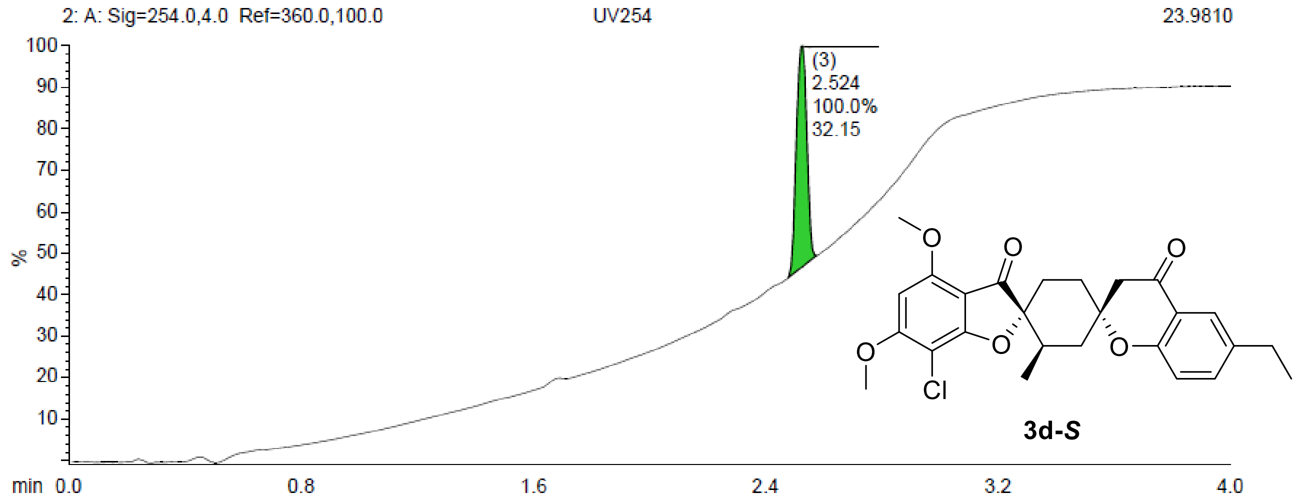

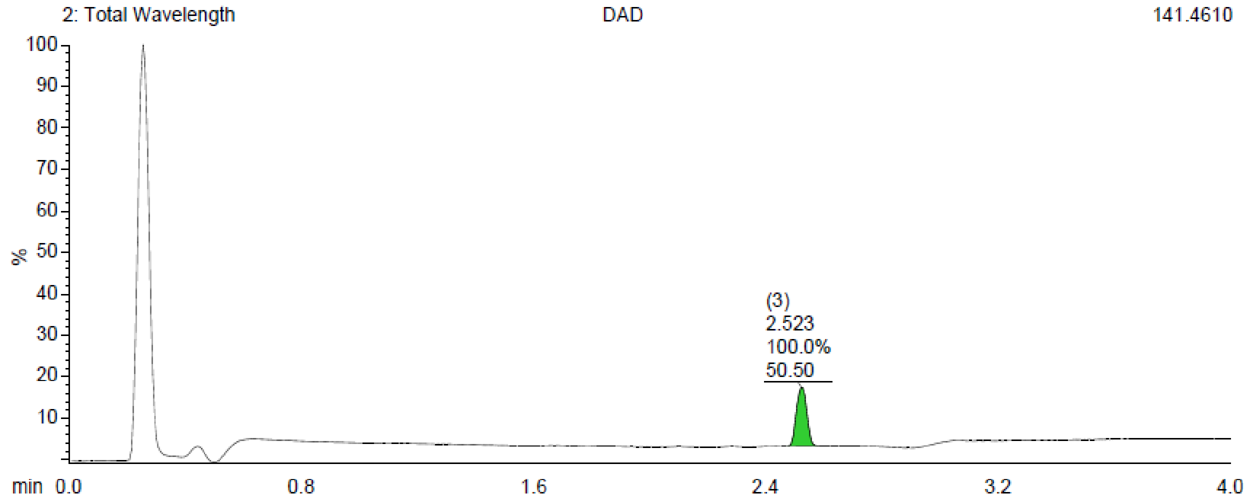

BPM	Error PPM	Error mDa	Target
304.2999			

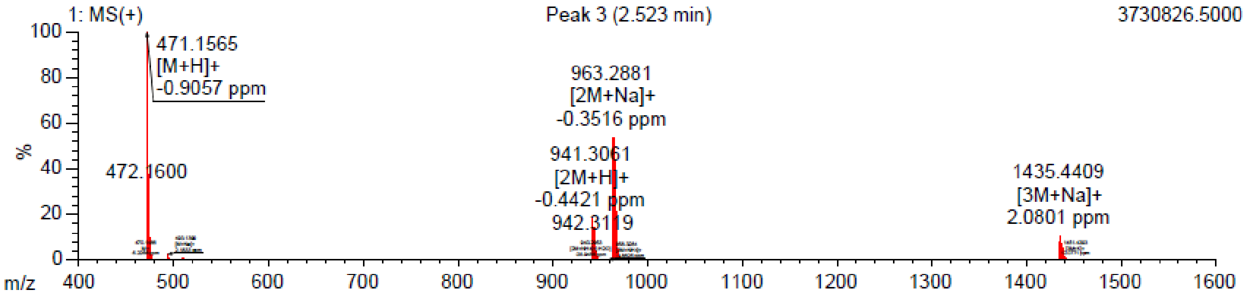

BPM	Error PPM	Error mDa	Target
471.1565	12.0771	17.5289	470.1…

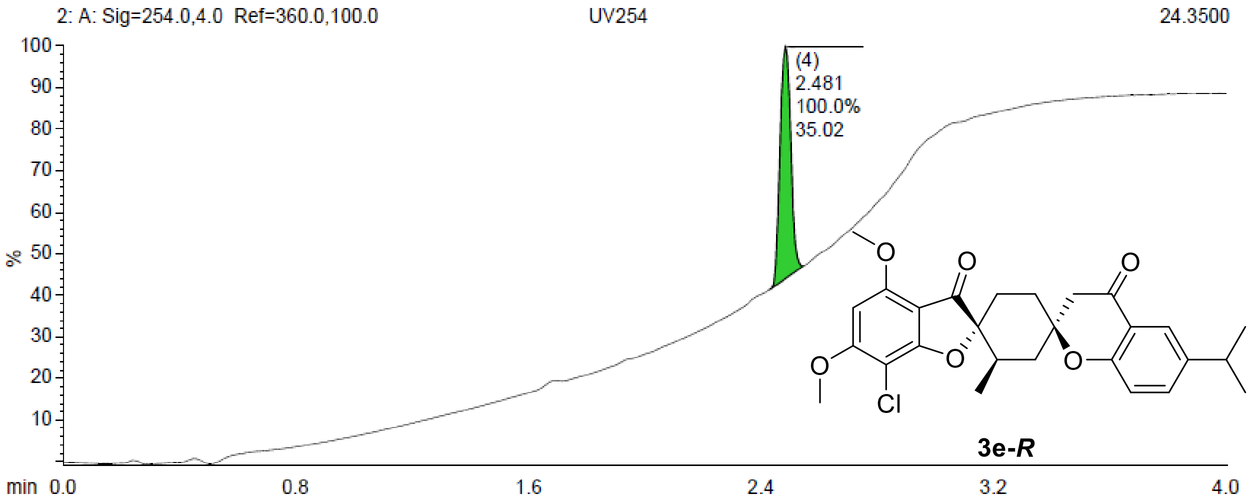

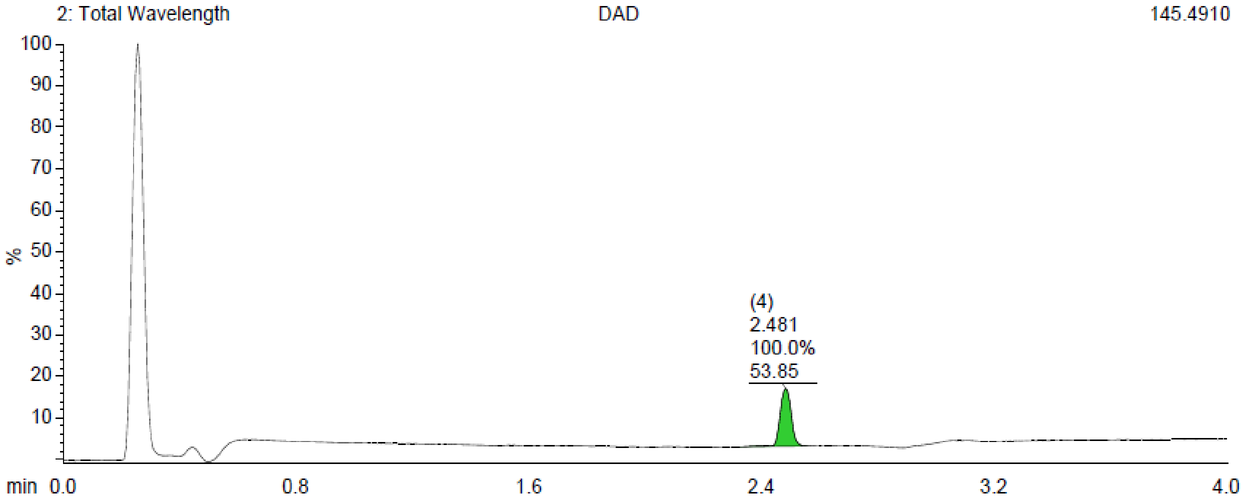

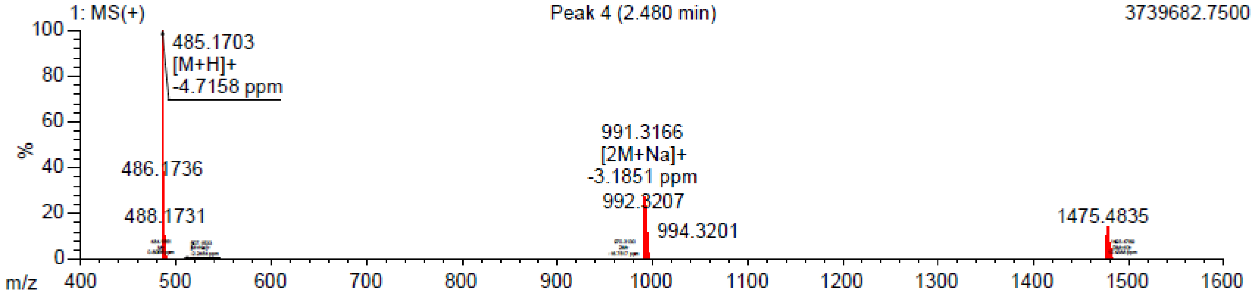

BPM	Error PPM	Error mDa	Target
485.1703	13.4388	20.0702	484.1...

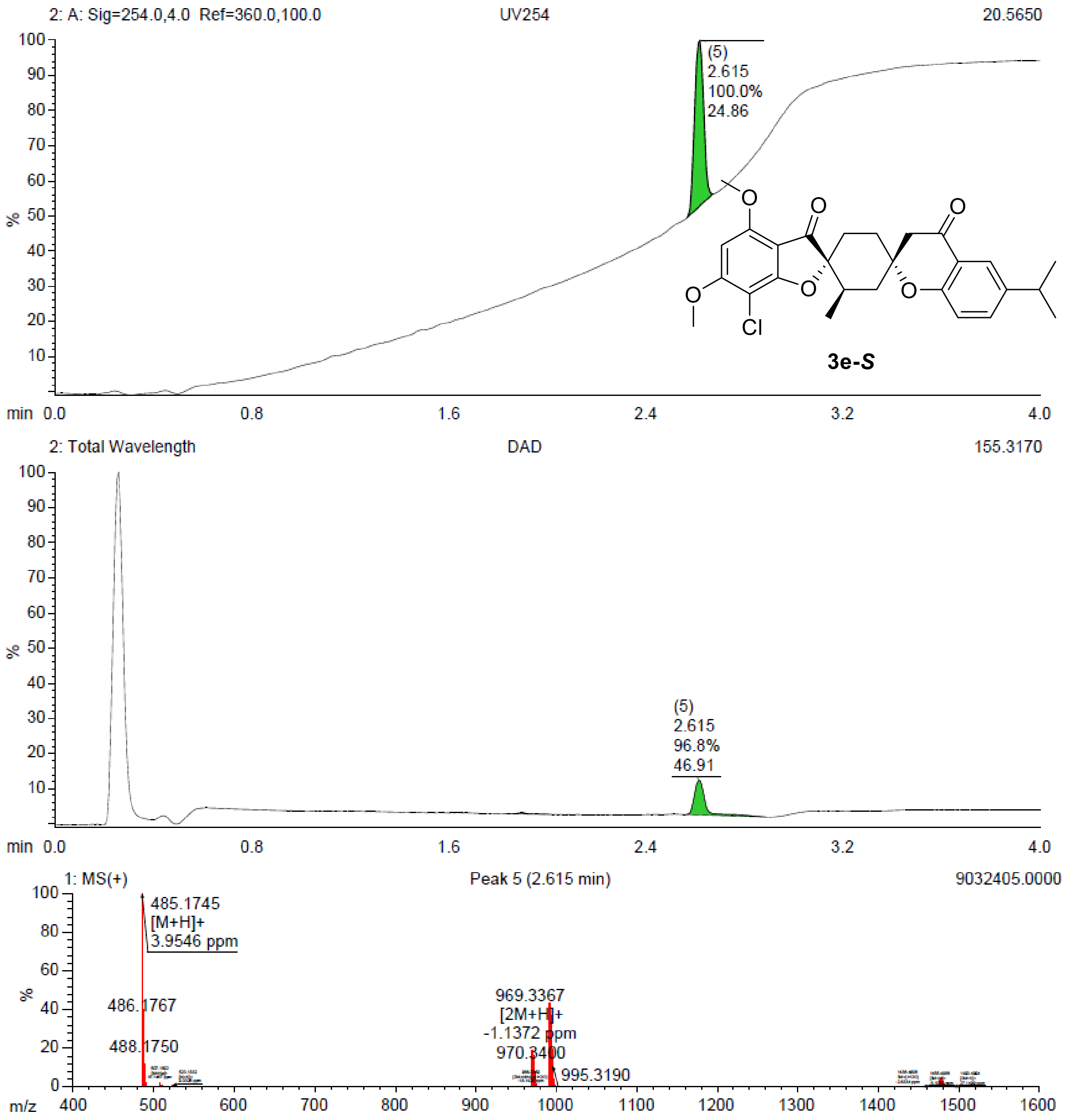

BPM	Error PPM	Error mDa	Target
485.1745	27.1499	40.5473	485.1…

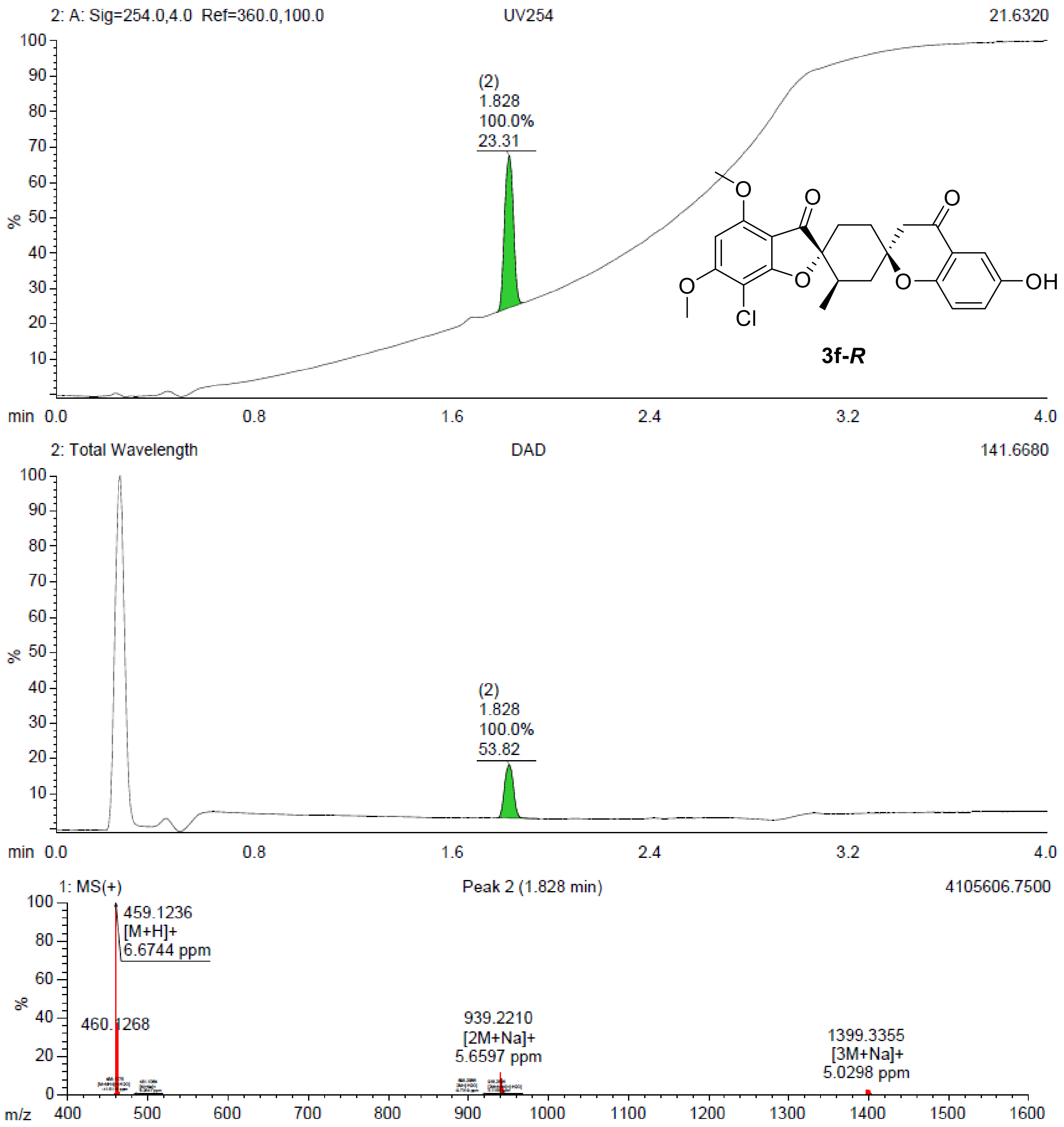

BPM	Error PPM	Error mDa	Target
459.1236	1.9733	2.7928	458.1…

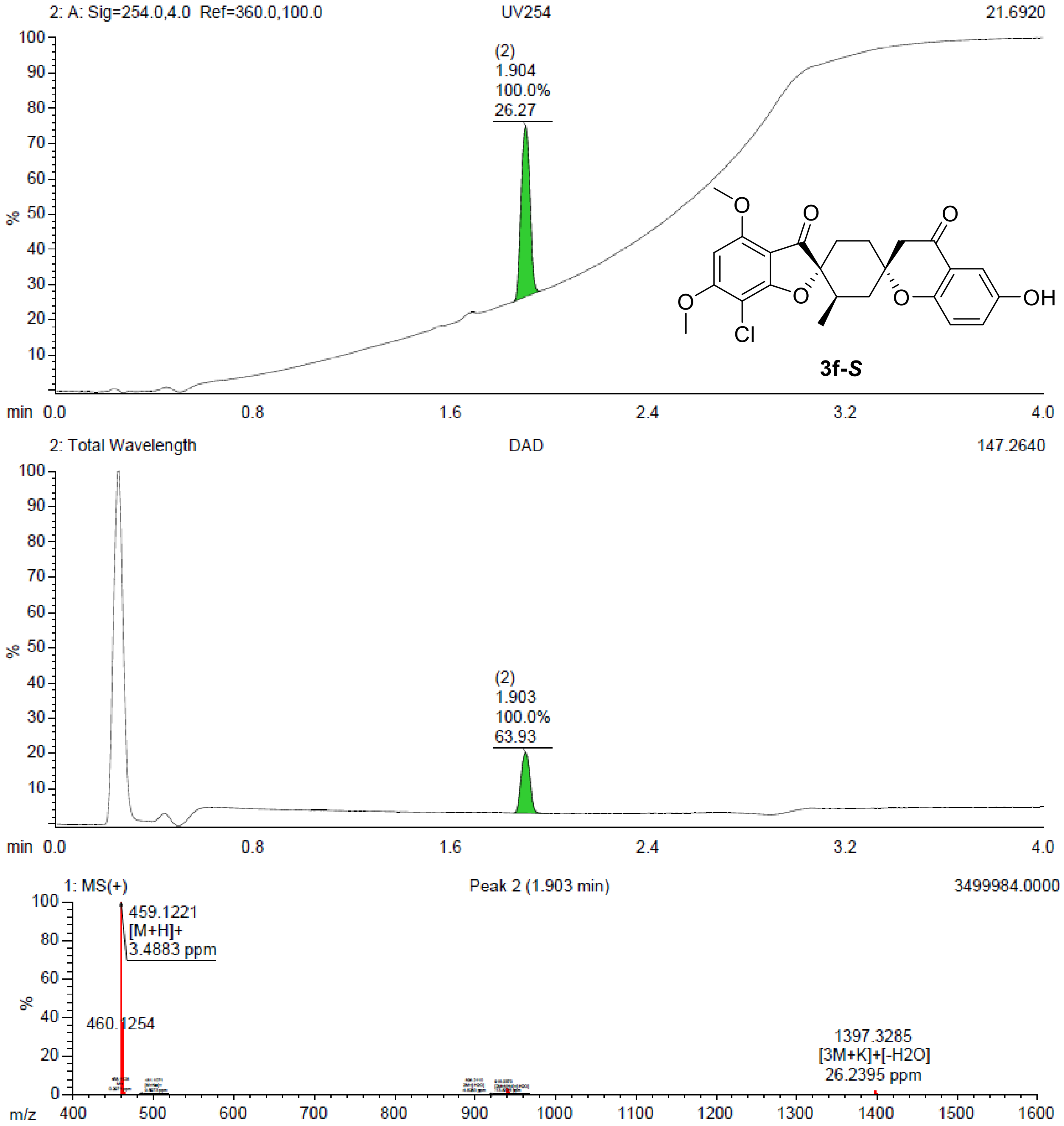

BPM	Error PPM	Error mDa	Target
459.1221	12.1587	17.2082	458.1…

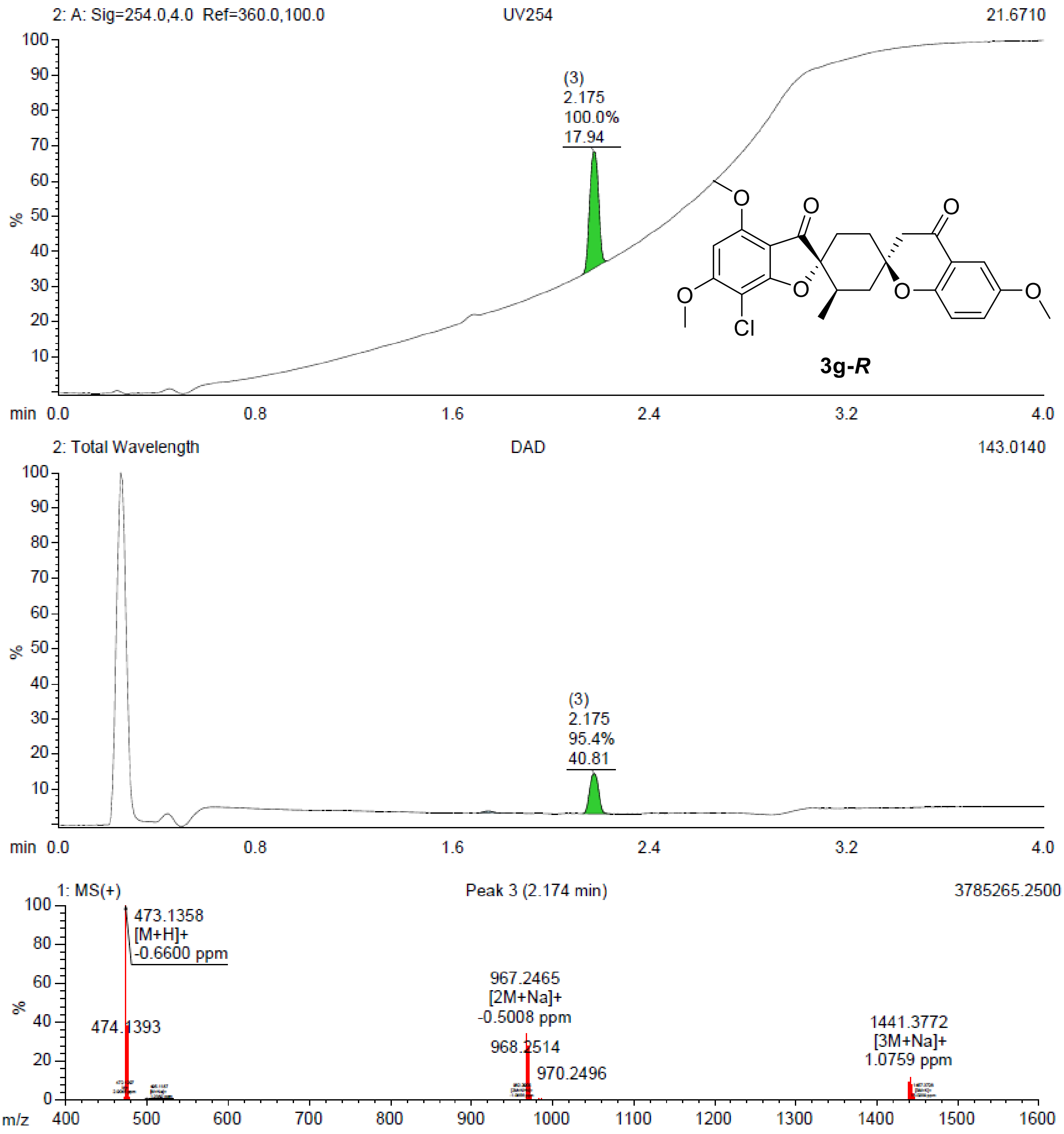

BPM	Error PPM	Error mDa	Target
473.1358	16.0209	23.3480	472.1...

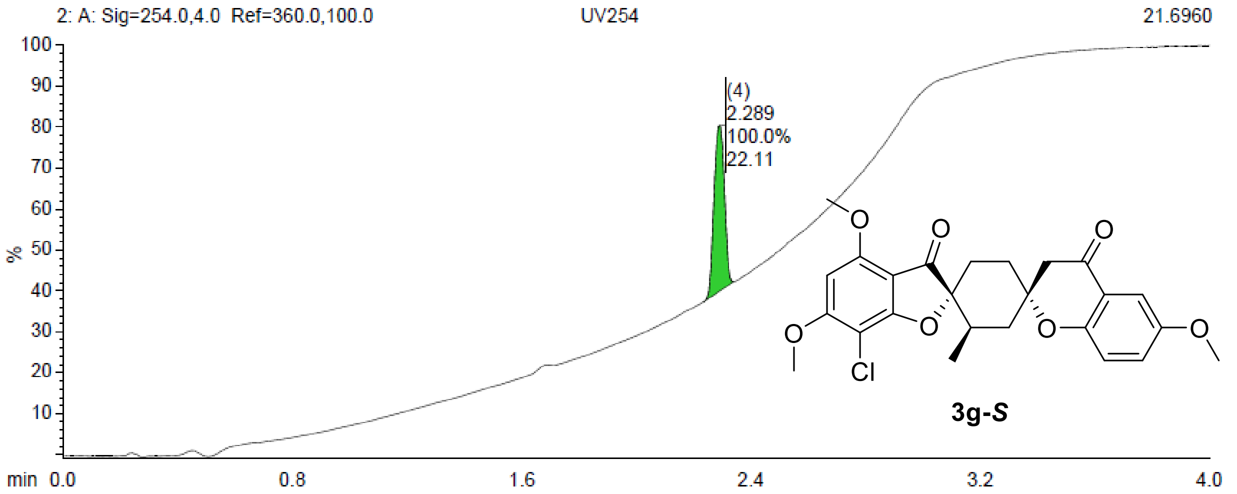

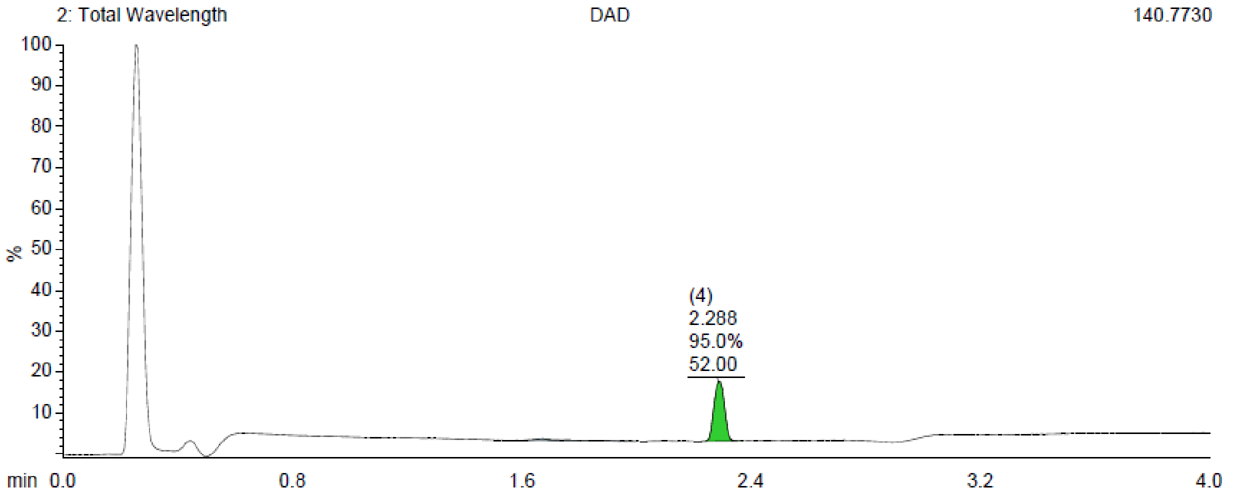

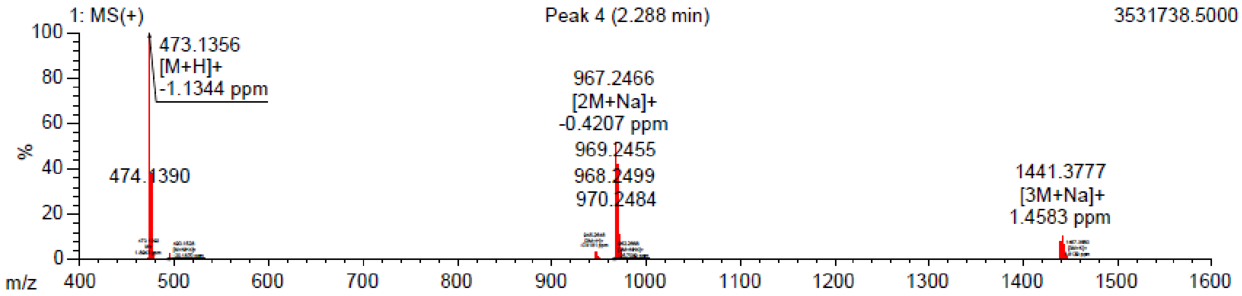

BPM	Error PPM	Error mDa	Target
473.1356	3.8129	5.5568	472.1…

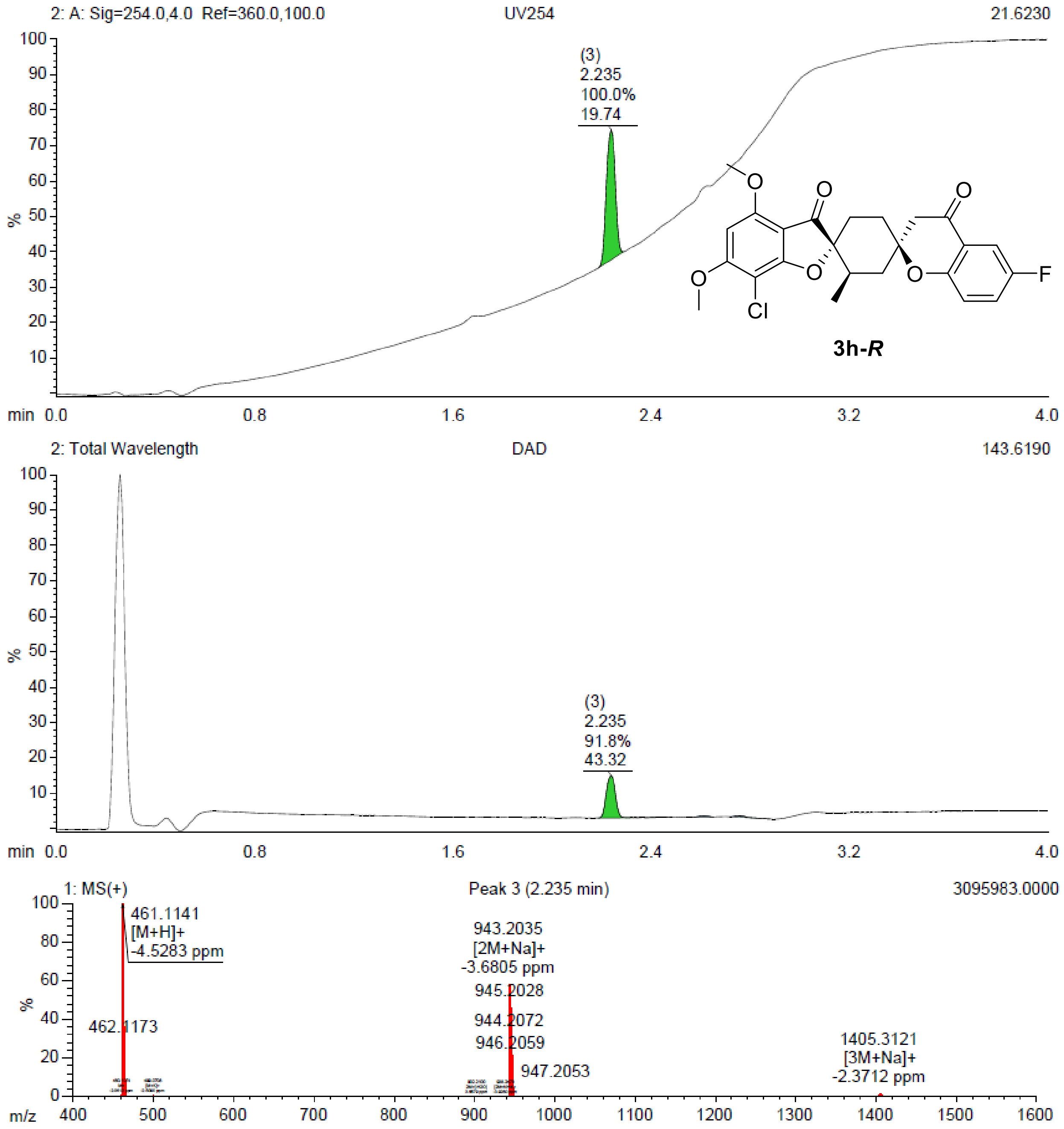
2: A: Sig=254.0,4.0 Ref=360.0,100.0
UV254
21.6230
(3)
2.235
100.0%
19.74
%
min
0.0
0.8
1.6
2.4
3.2
4.0
O
O
O
O
O
O
F
Cl
3h-R
2: Total Wavelength
DAD
143.6190
(3)
2.235
91.8%
43.32
1: MS(+)
Peak 3 (2.235 min)
3095983.0000
461.1141
[M+H]+
-4.5283 ppm
462.1173
943.2035
[2M+Na]+
-3.6805 ppm
945.2028
944.2072
946.2059
947.2053
1405.3121
[3M+Na]+
-2.3712 ppm
m/z

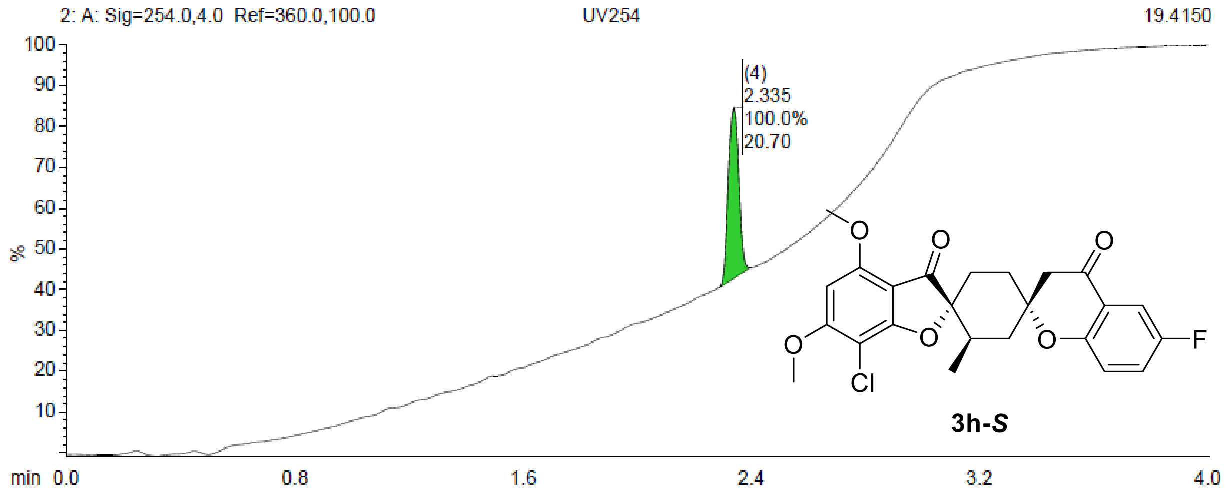

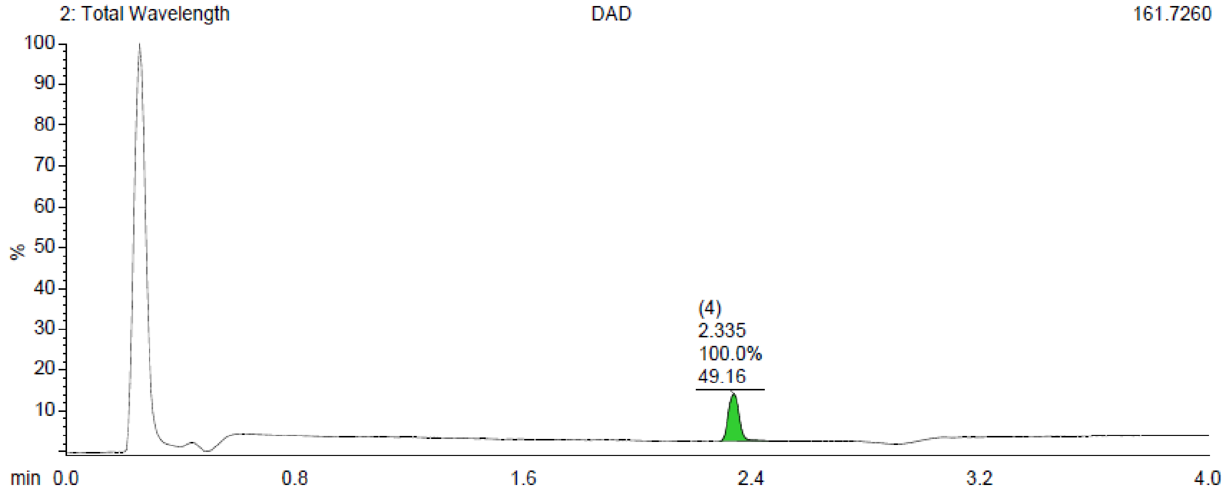

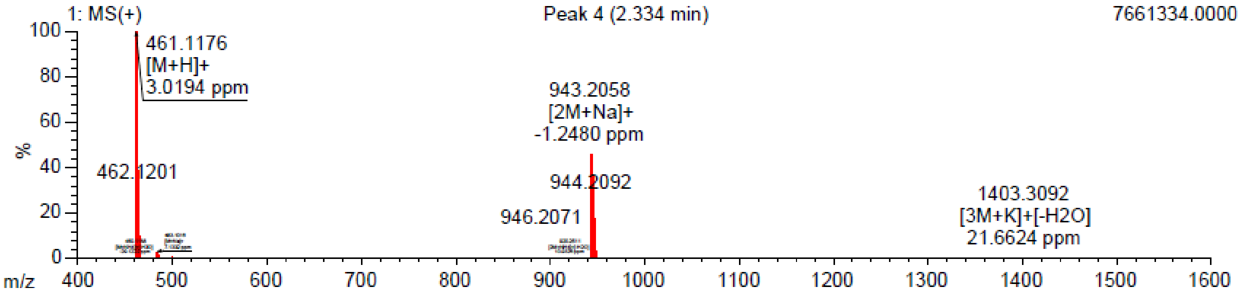

BPM	Error PPM	Error mDa	Target
461.1176	21.6624	30.3983	460.1…

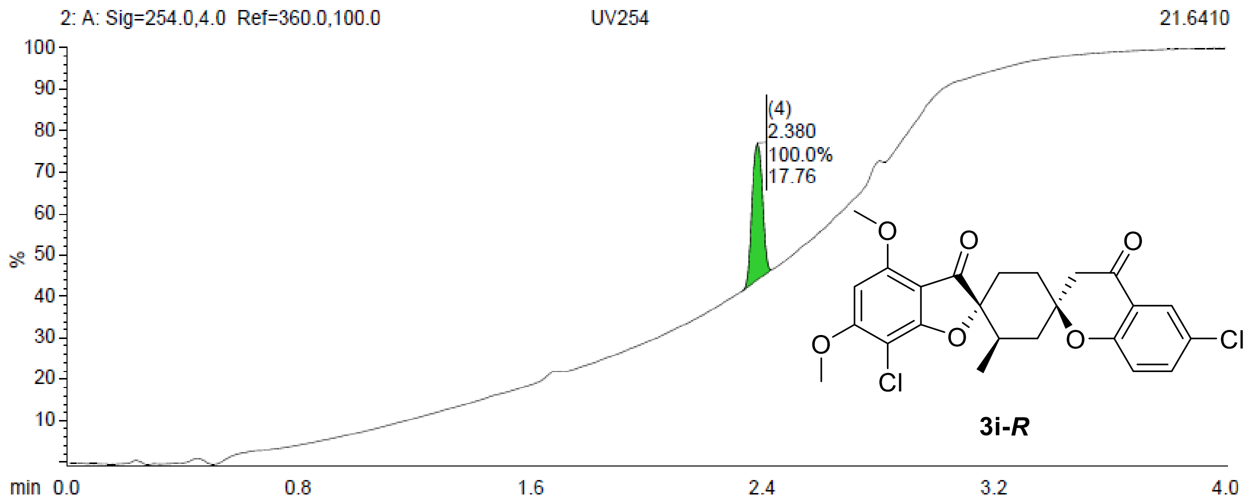

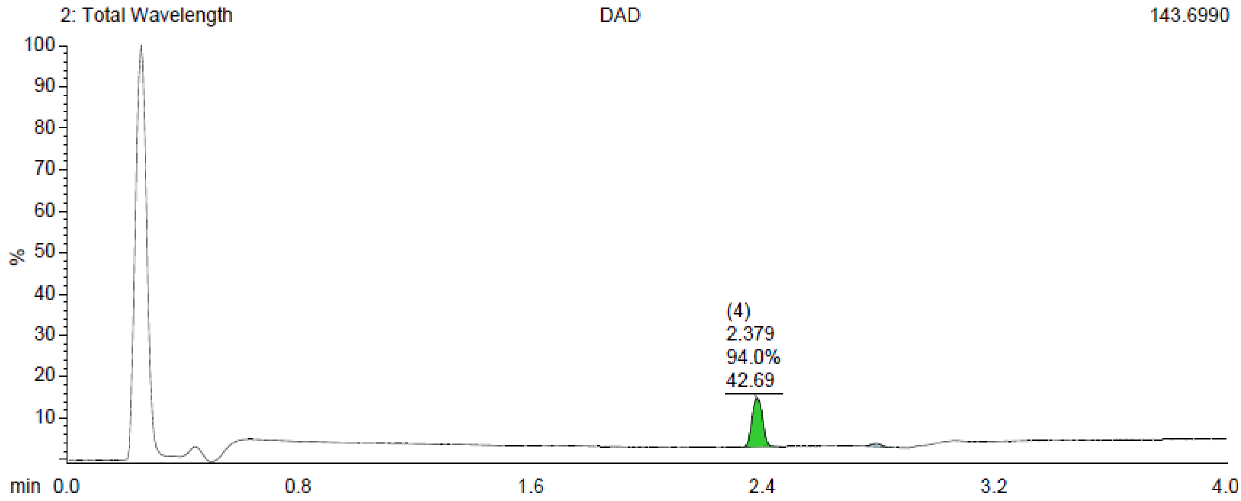

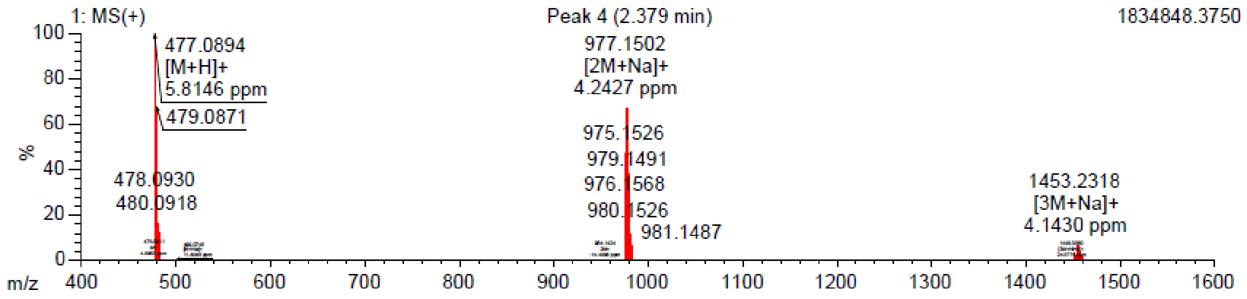

BPM	Error PPM	Error mDa	Target
477.0894	4.1430	6.0208	476.0...

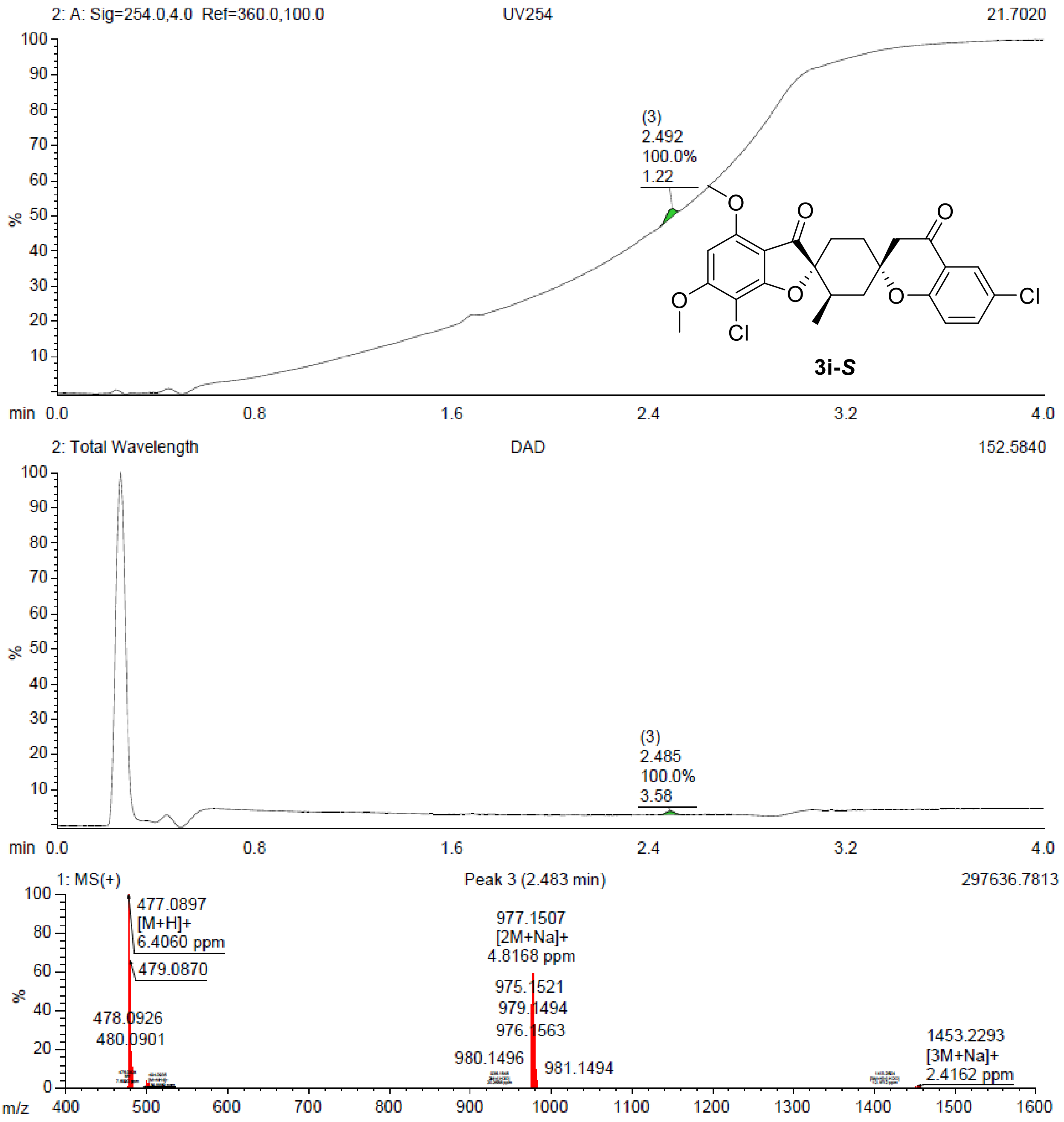

BPM	Error PPM	Error mDa	Target
477.0897	2.4162	3.5112	476.0...

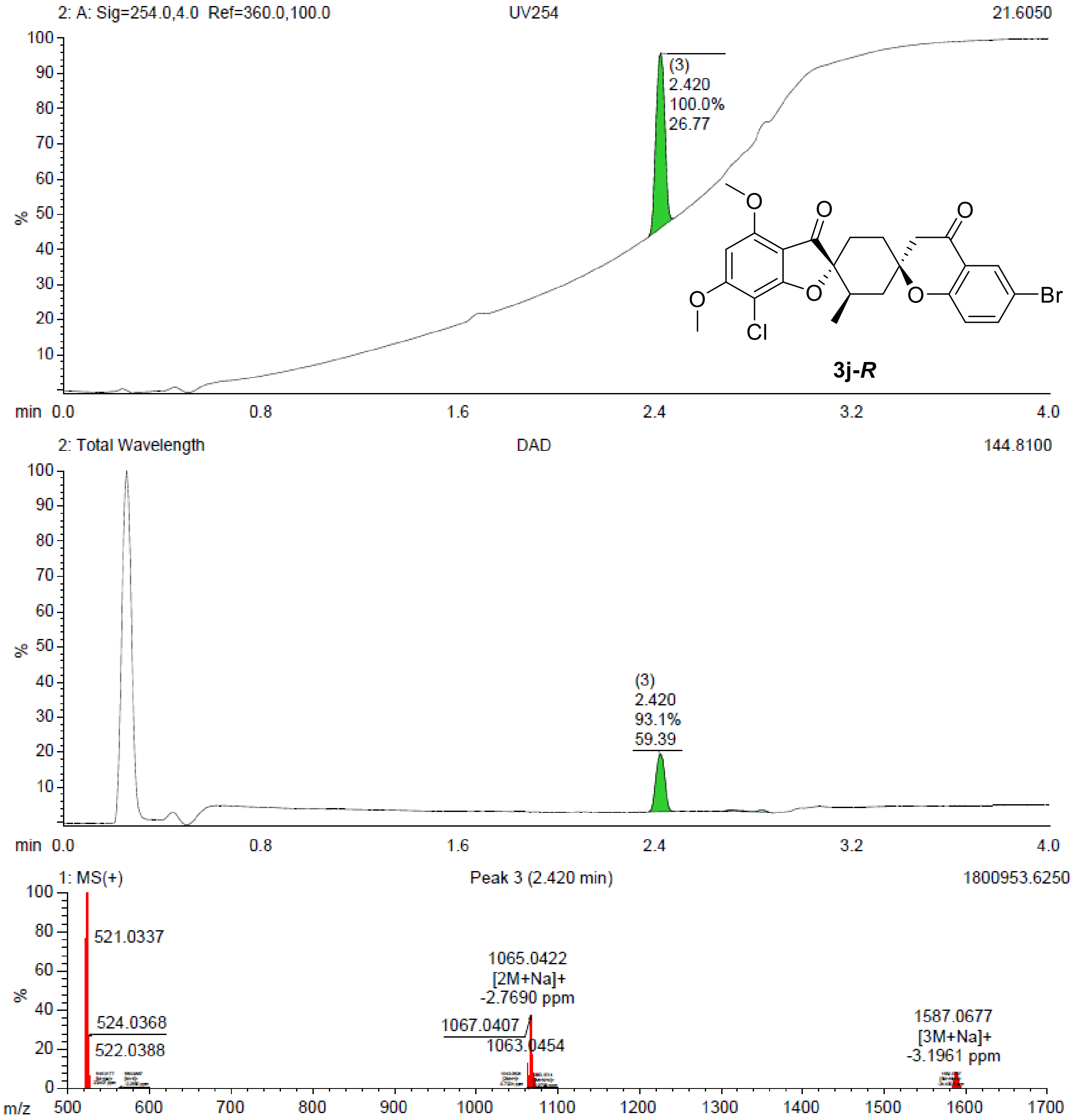

BPM	Error PPM	Error mDa	Target
523.0317	-3.1961	-5.0724	523.0…

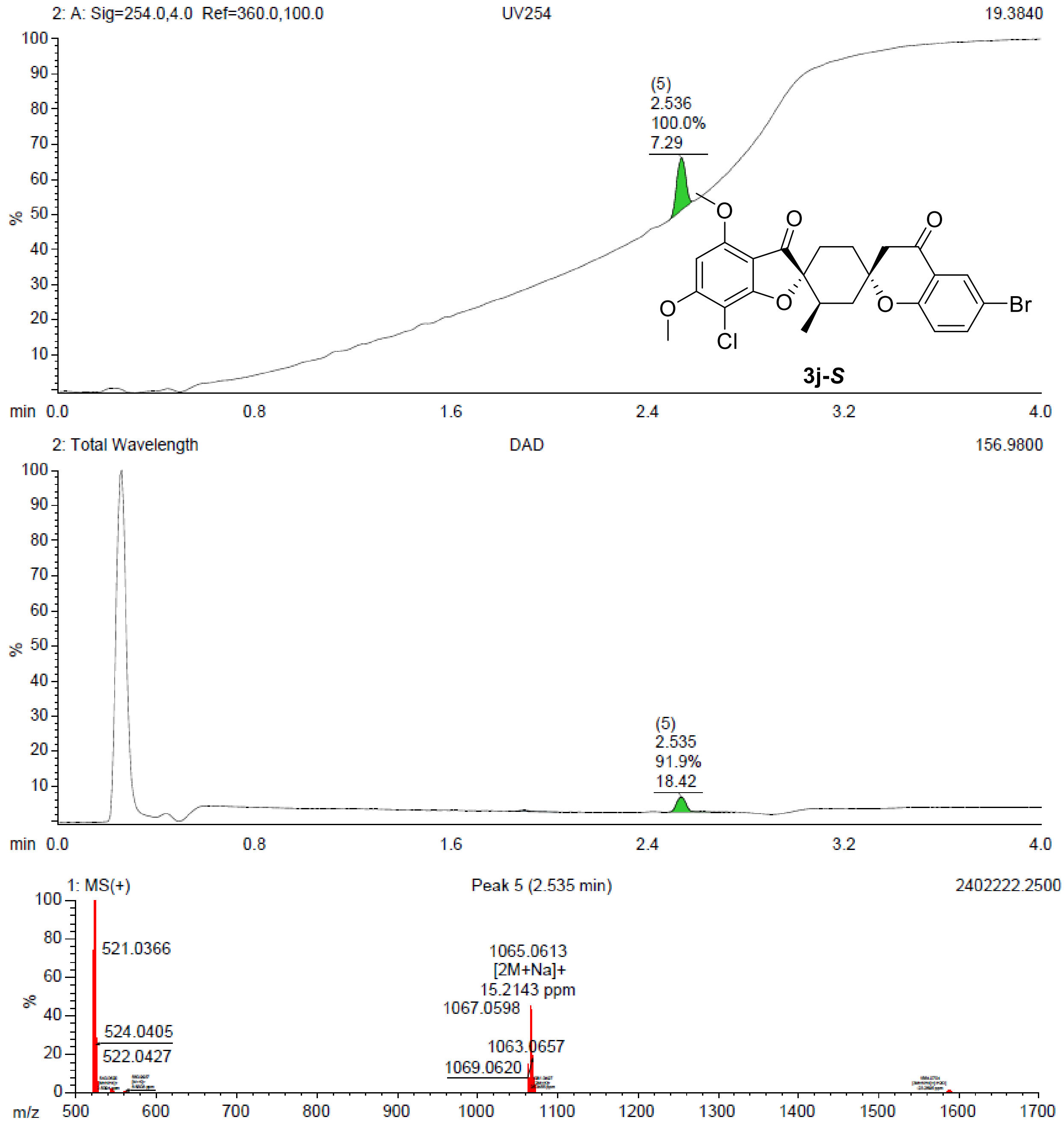

BPM	Error PPM	Error mDa	Target
523.0347	22.2255	35.3178	523.0…

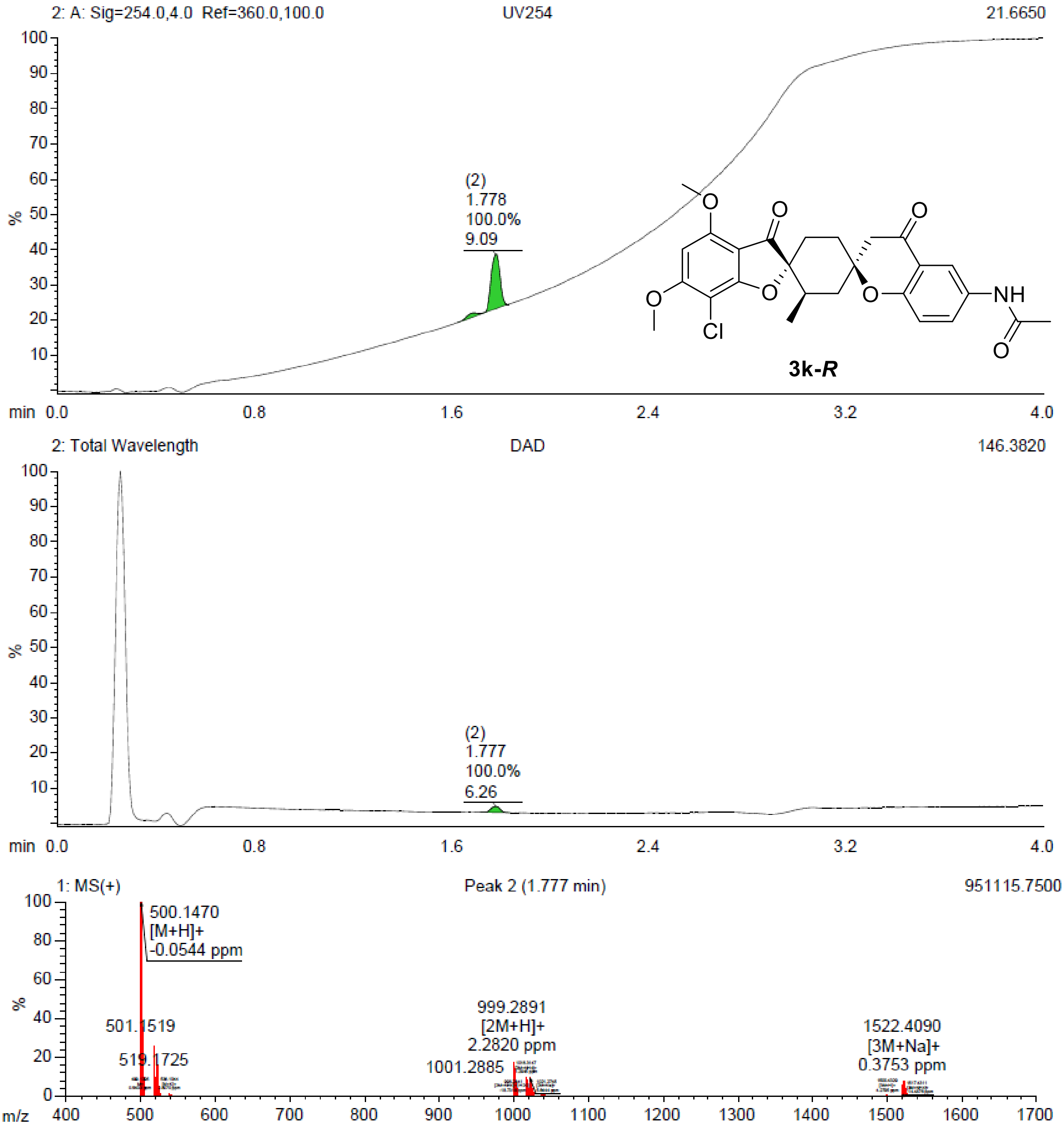

BPM	Error PPM	Error mDa	Target
500.1470	-22.8934	-35.2188	499.1…

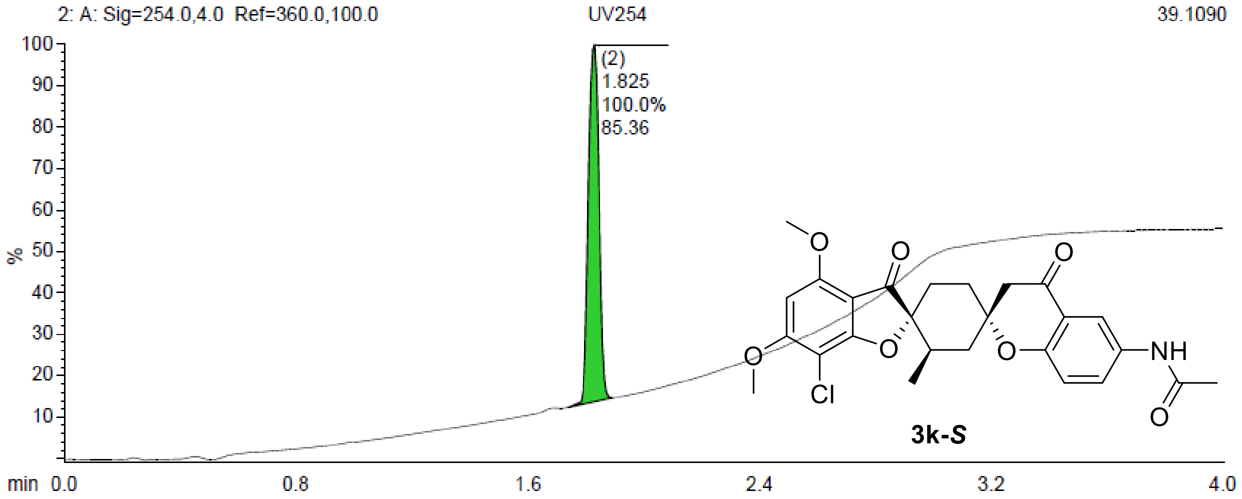

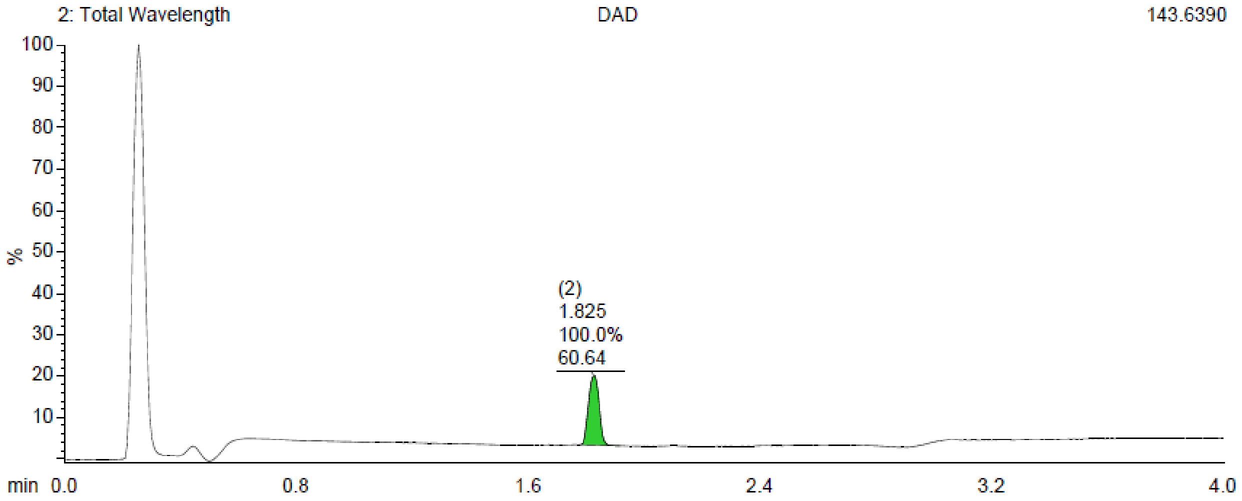

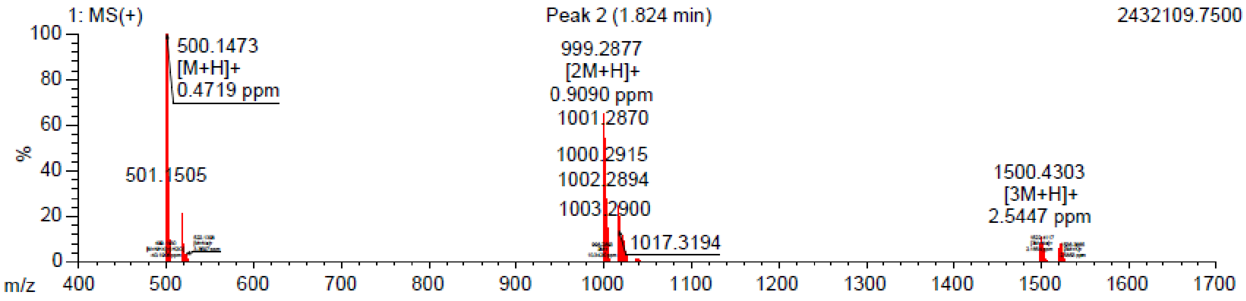

BPM	Error PPM	Error mDa	Target
500.1473	2.5853	3.9721	499.1...

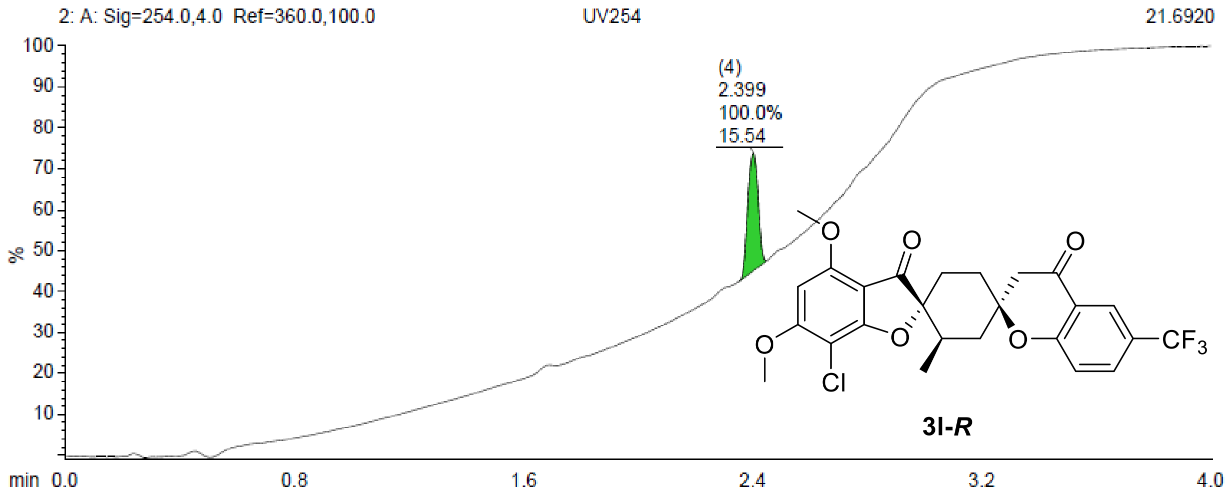

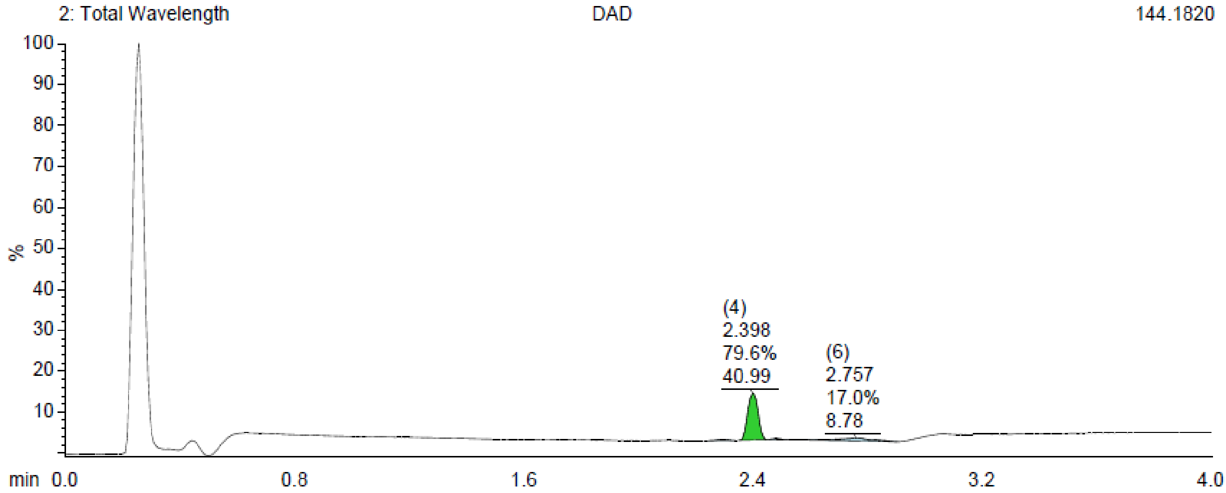

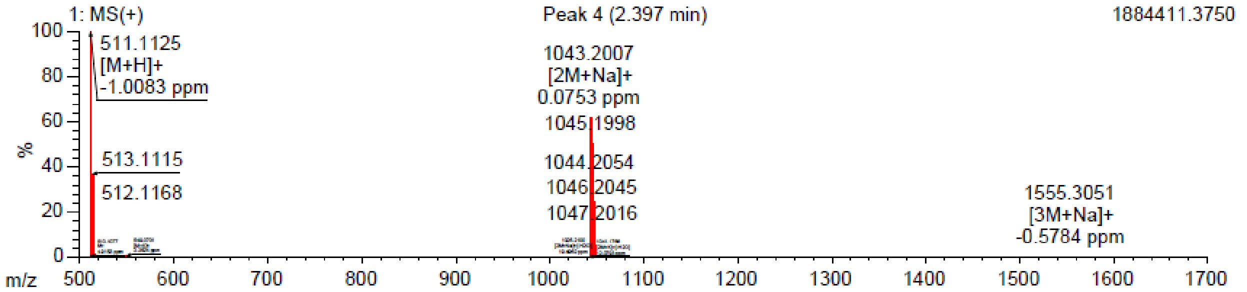

BPM	Error PPM	Error mDa	Target
511.1125	-0.5784	-0.8995	510.1…

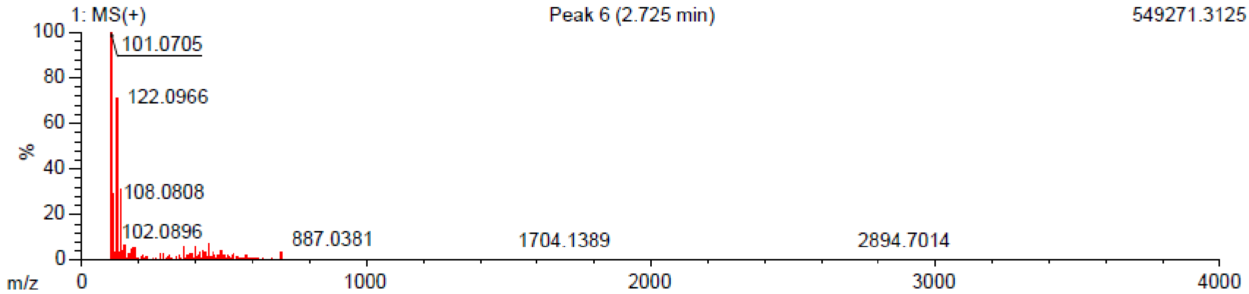

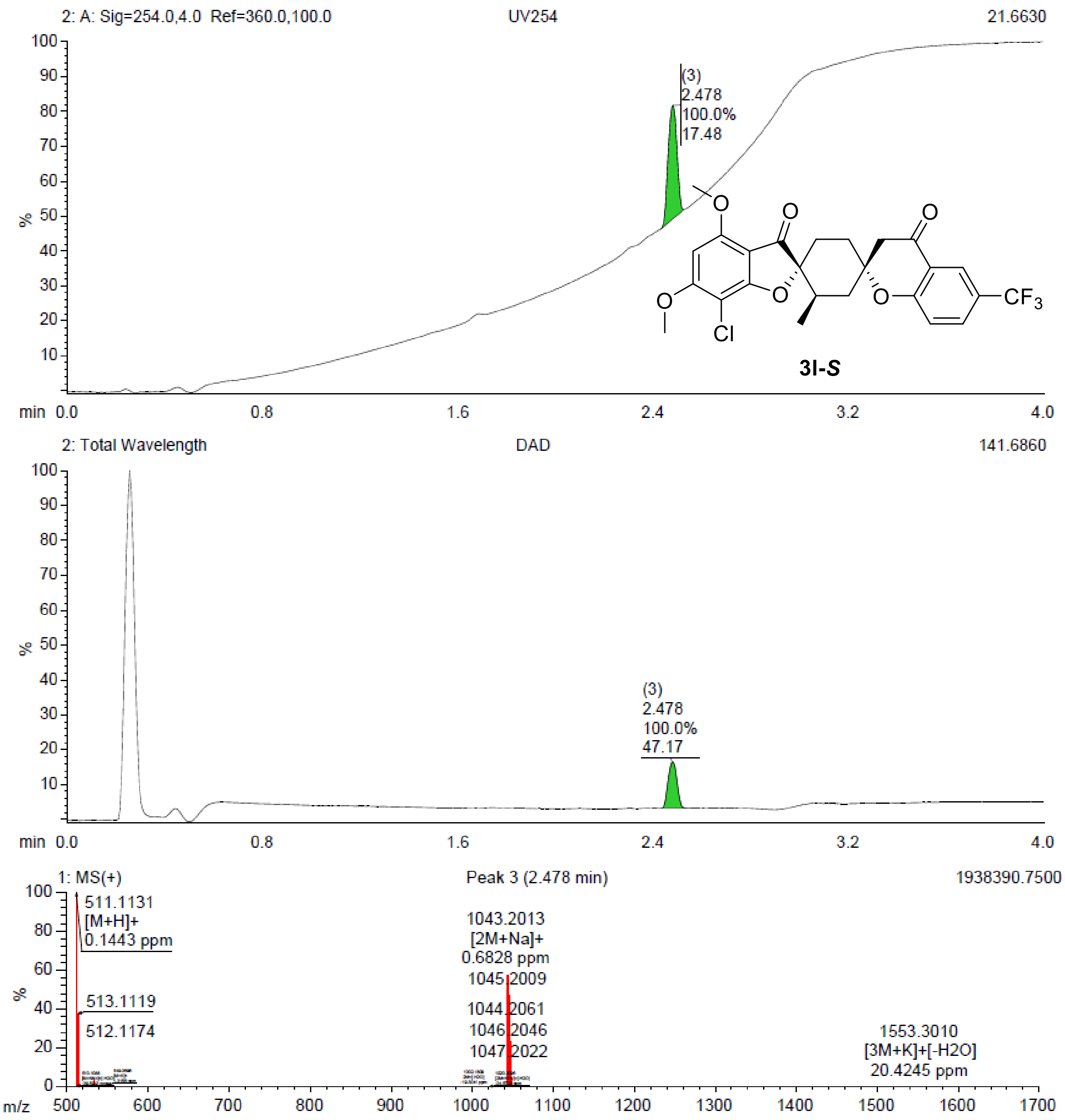

BPM	Error PPM	Error mDa	Target
511.1131	7.3611	11.4488	510.1...

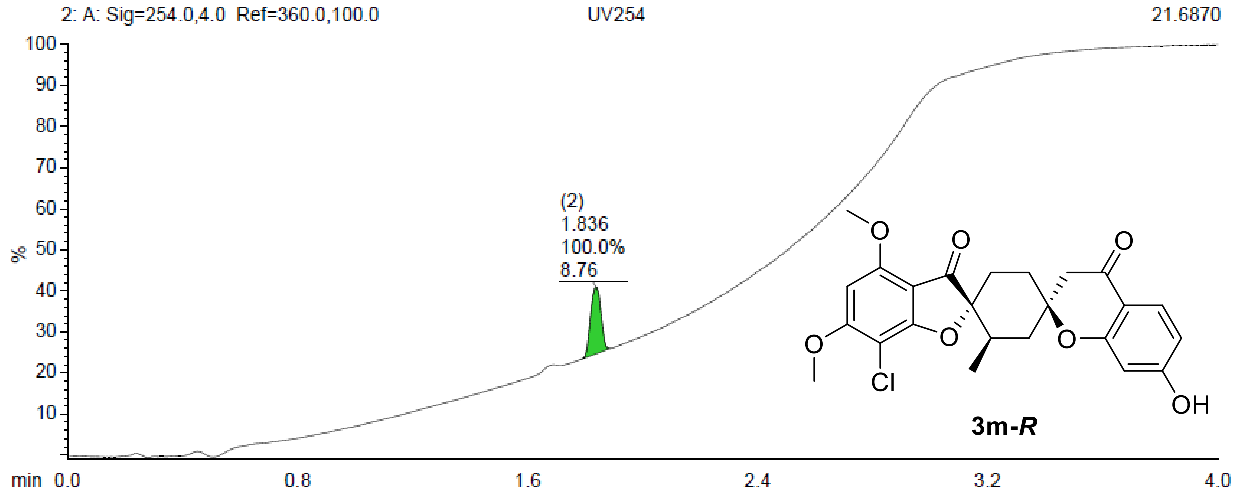

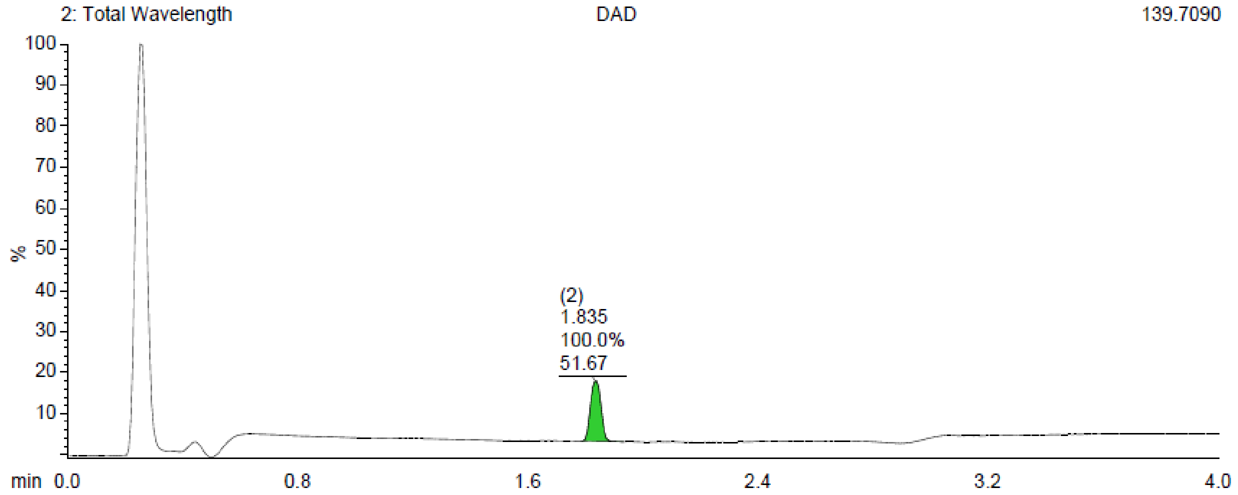

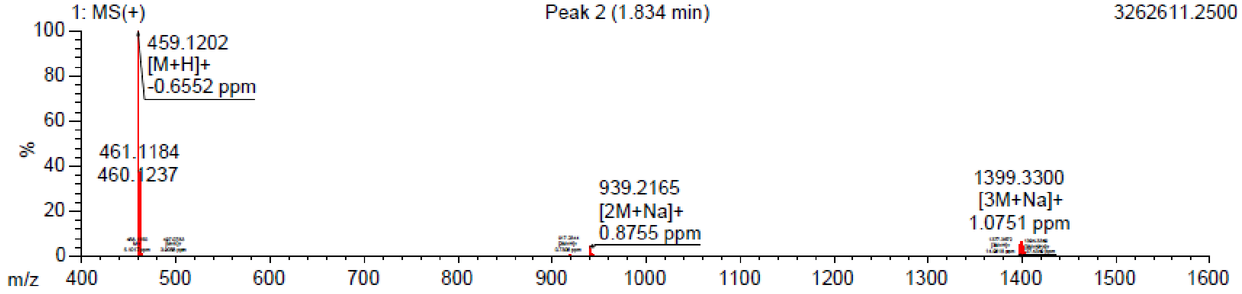

BPM	Error PPM	Error mDa	Target
459.1202	-3.6882	-5.2199	458.1...

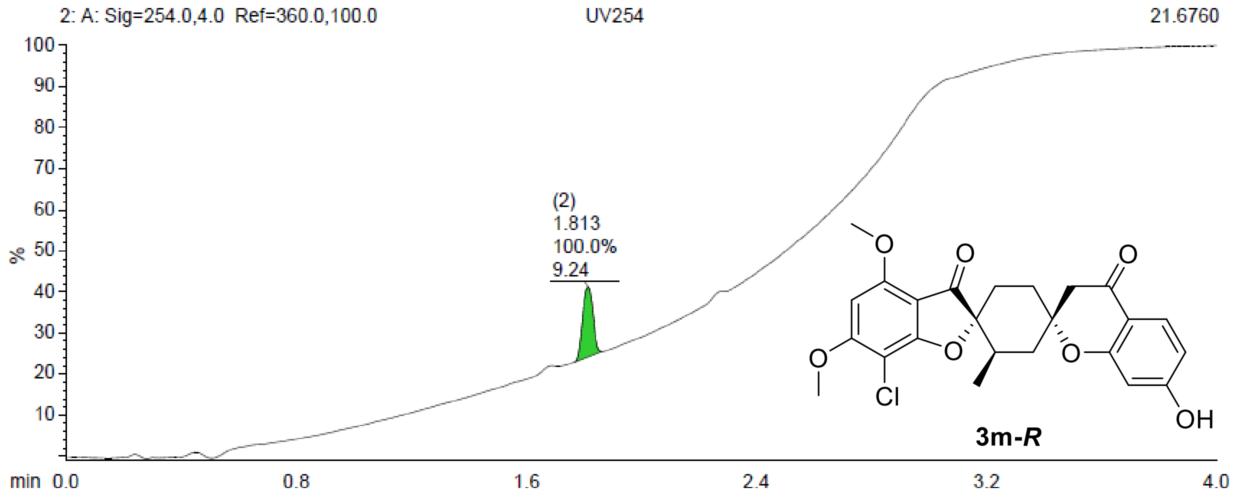

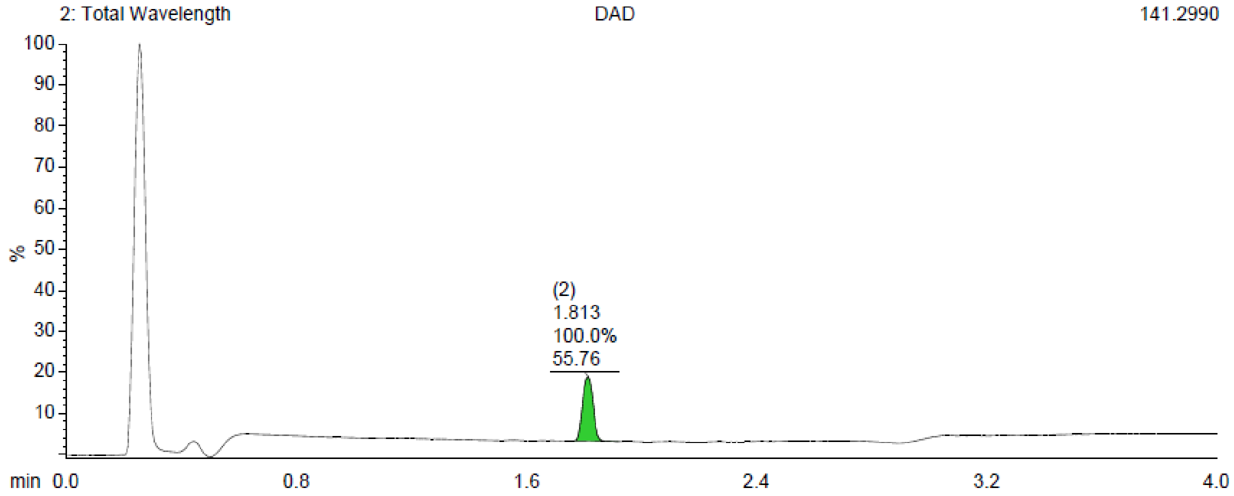

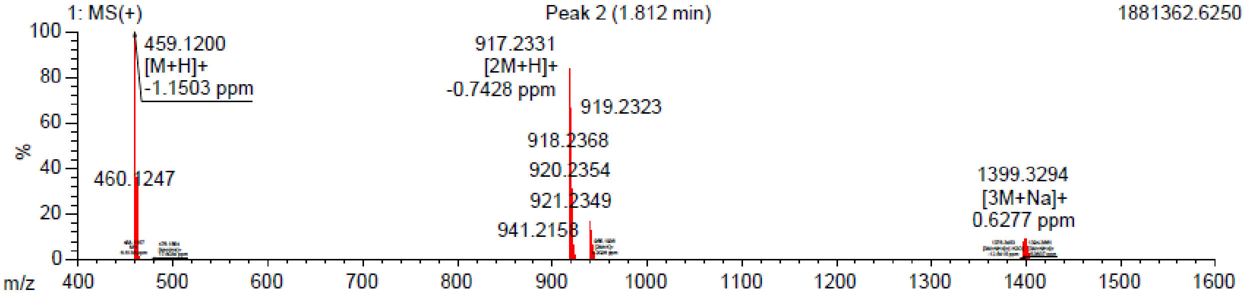

BPM	Error PPM	Error mDa	Target
459.1200	-1.6072	-2.2747	458.1…

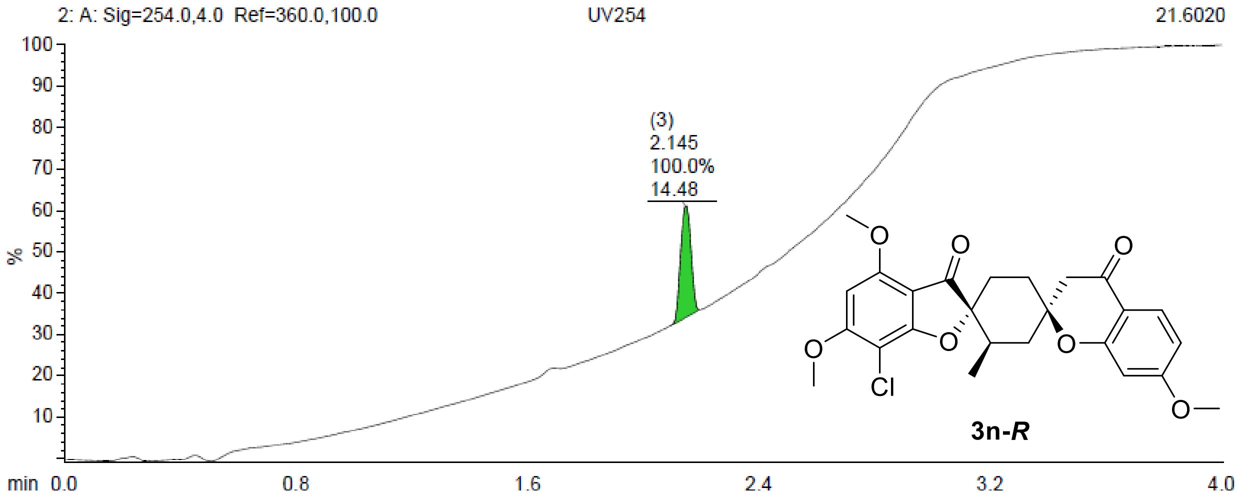

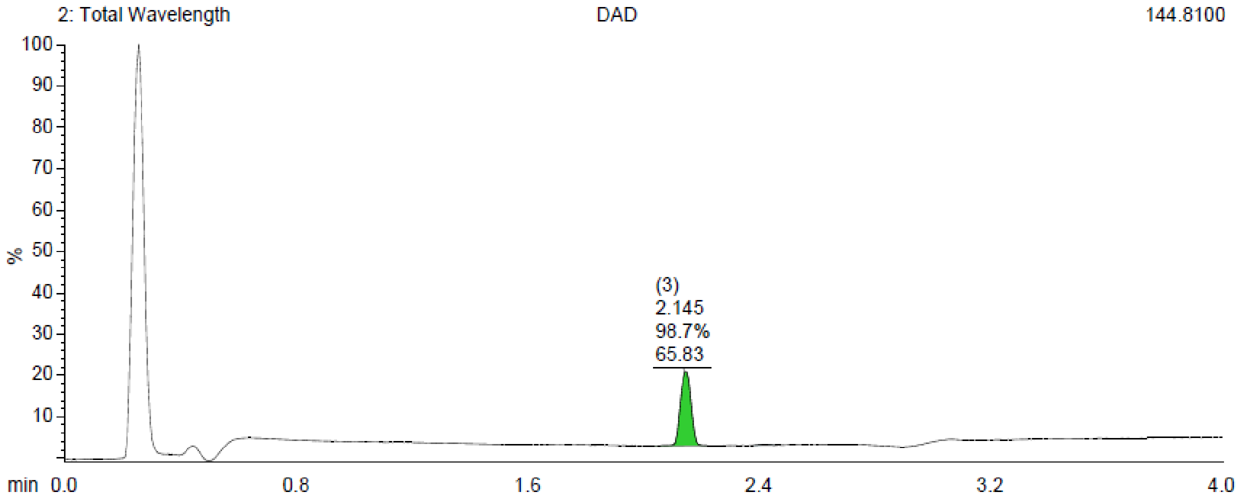

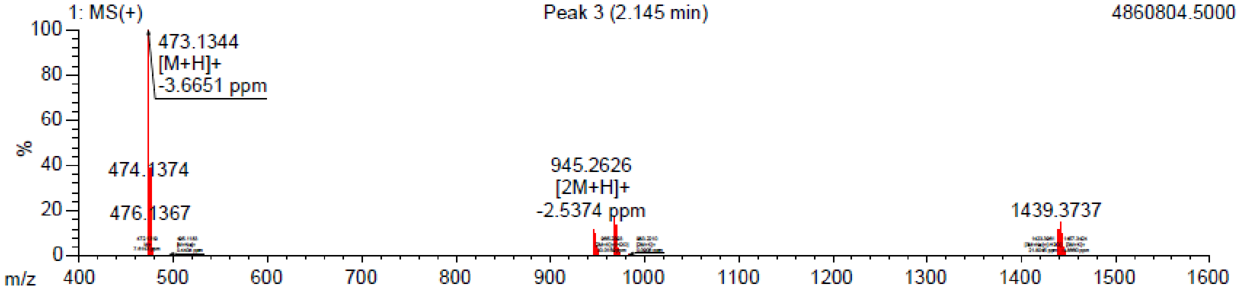

BPM	Error PPM	Error mDa	Target
473.1344	-4.8660	-7.0915	472.1…

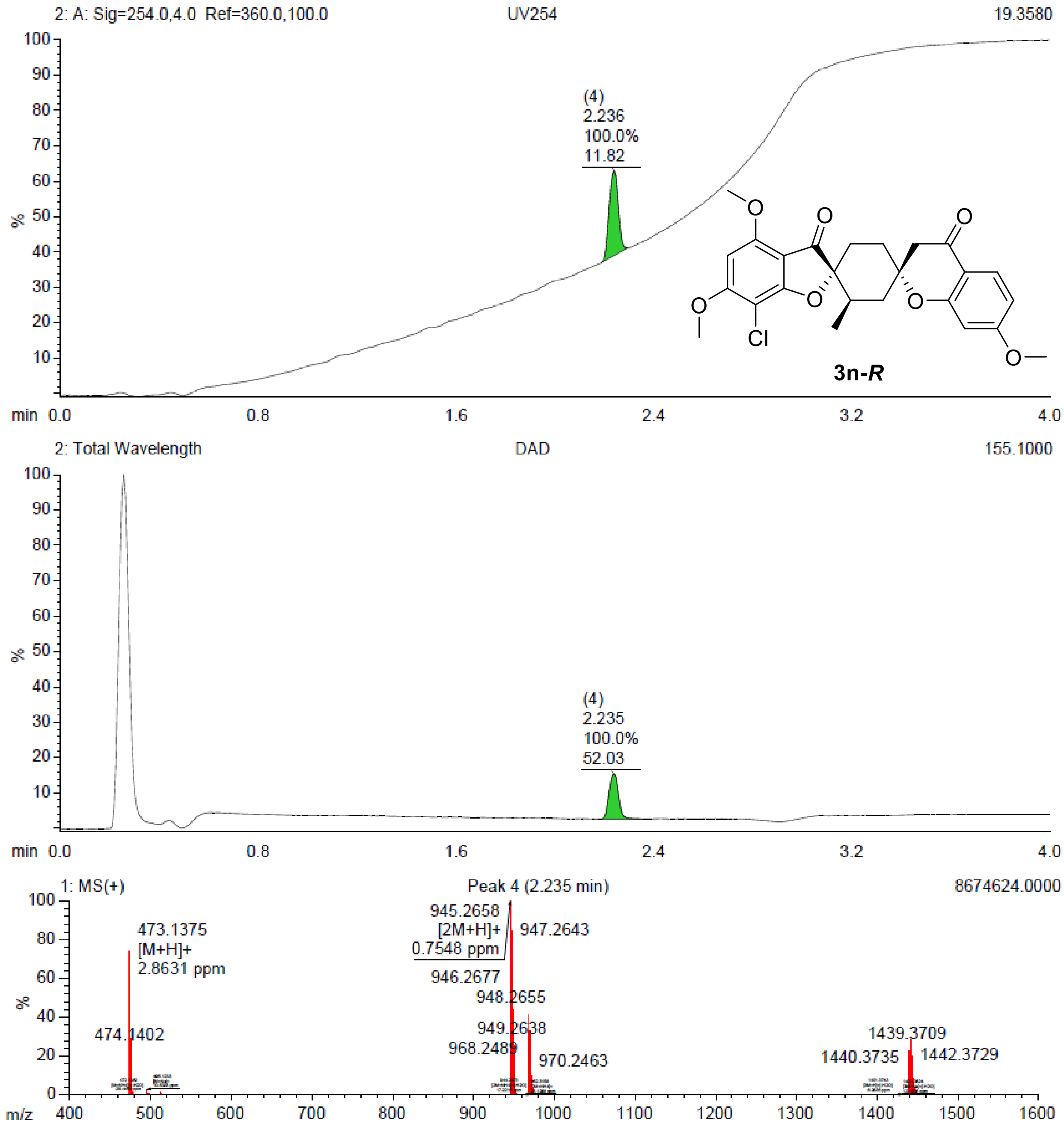

BPM	Error PPM	Error mDa	Target
945.2658	-9.0673	-13.2142	472.1...

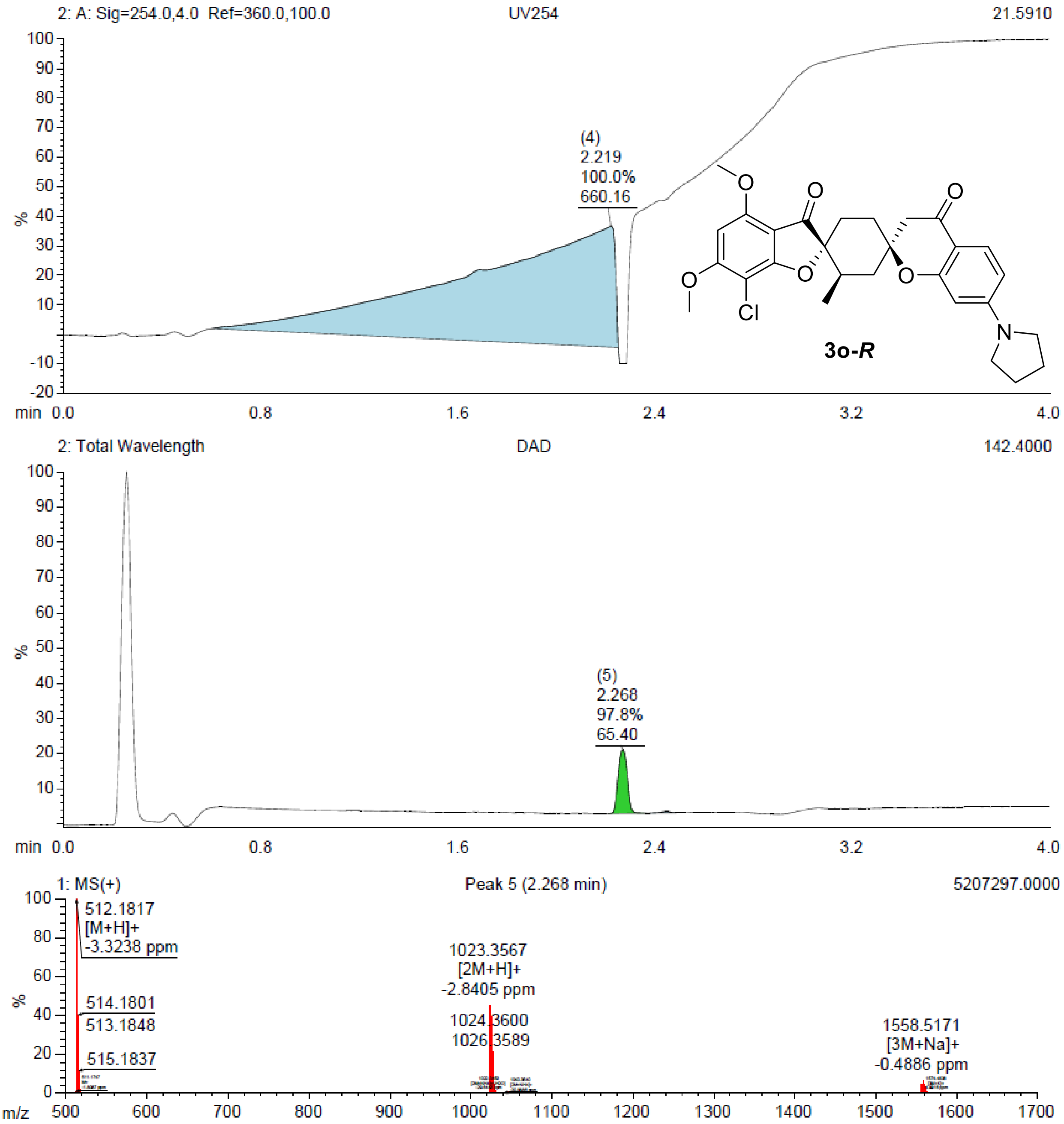

BPM	Error PPM	Error mDa	Target
512.1817	-4.9916	-7.8592	511.1…

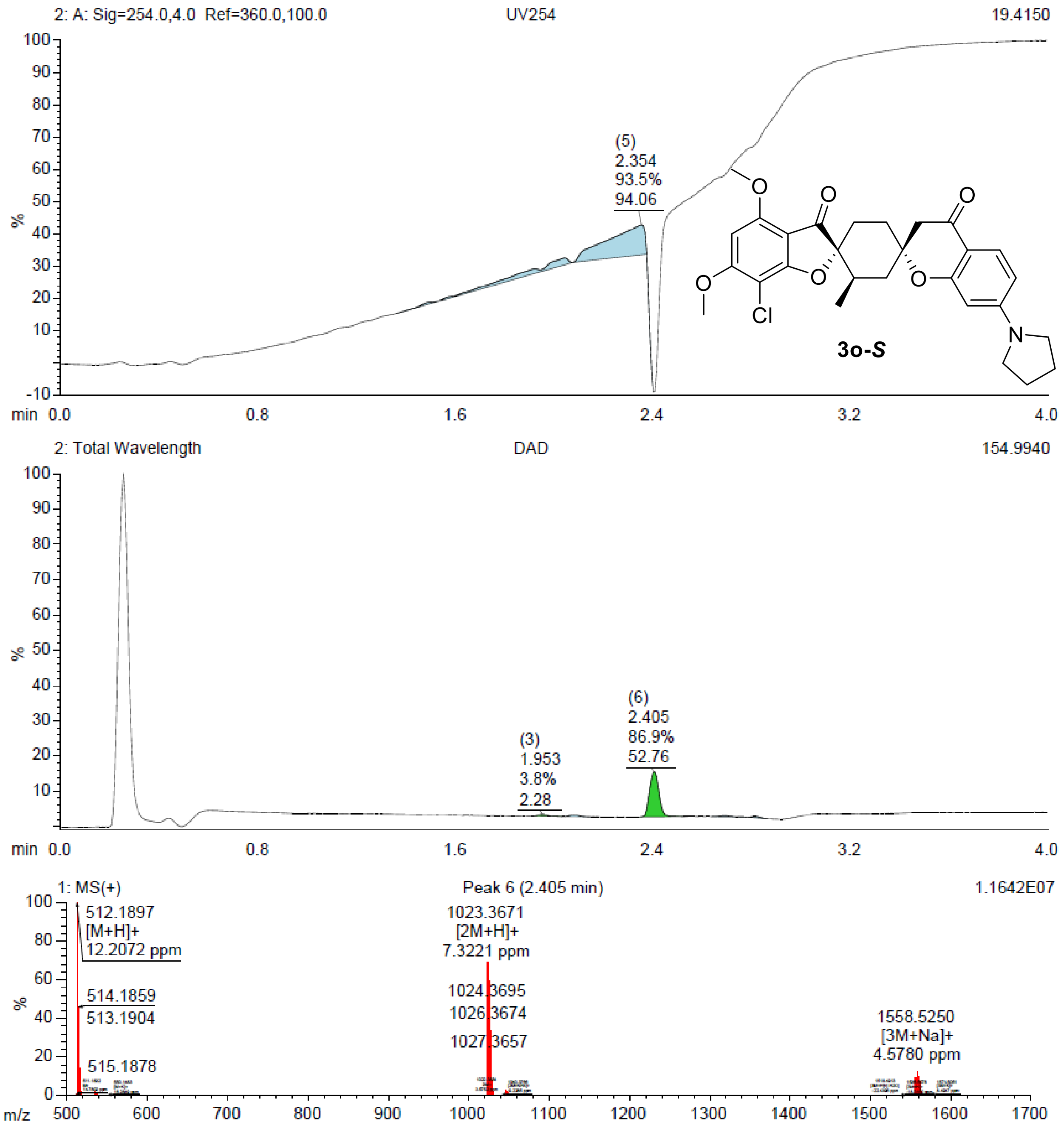

BPM	Error PPM	Error mDa	Target
512.1897	8.4947	13.3748	511.1…

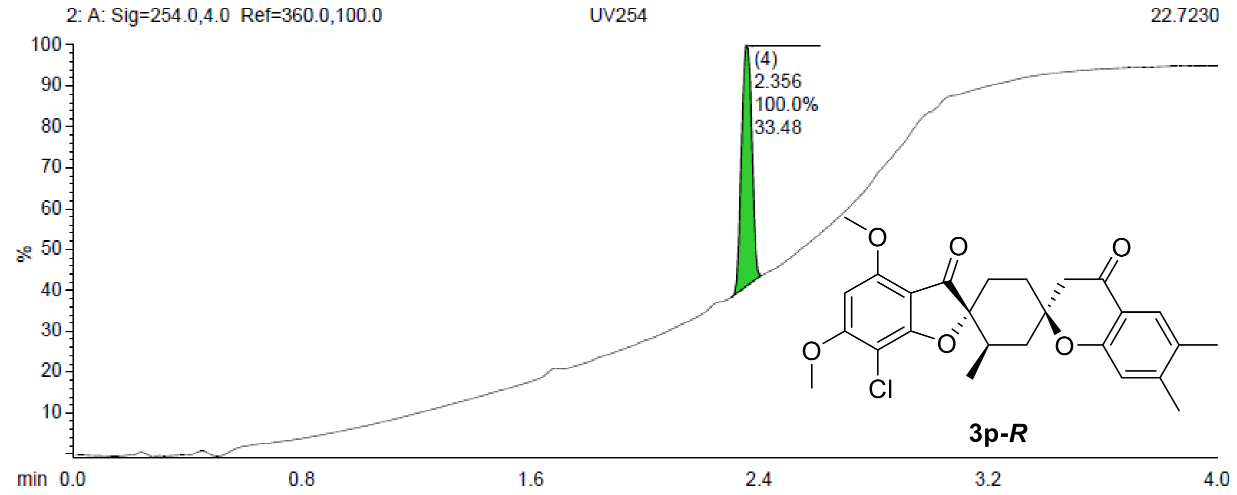

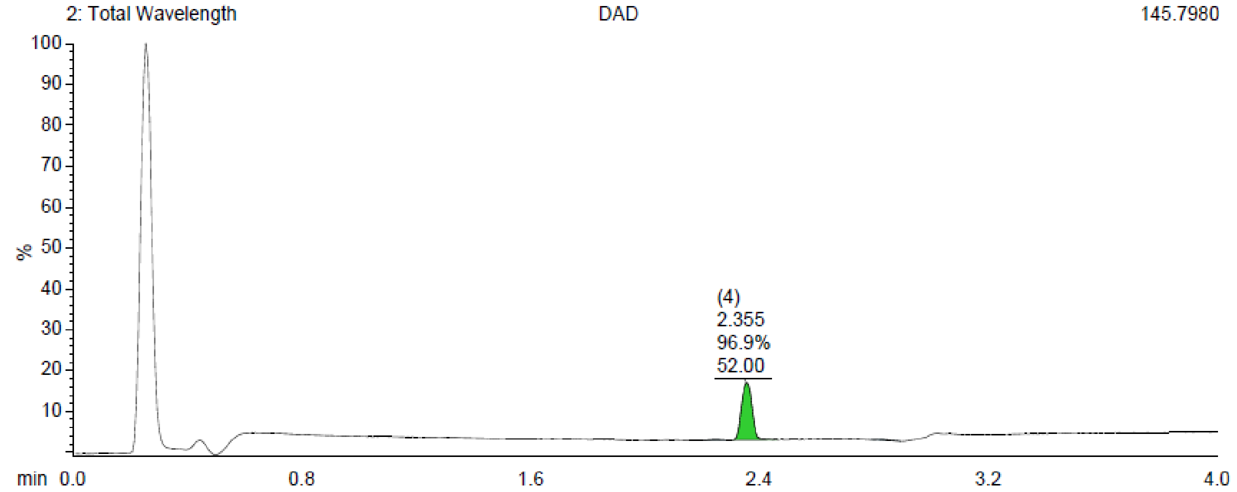

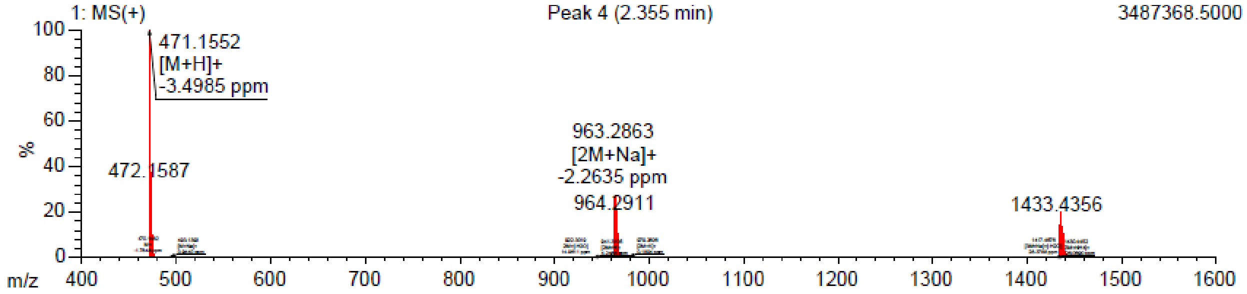

BPM	Error PPM	Error mDa	Target
471.1552	14.5578	21.1293	470.1…

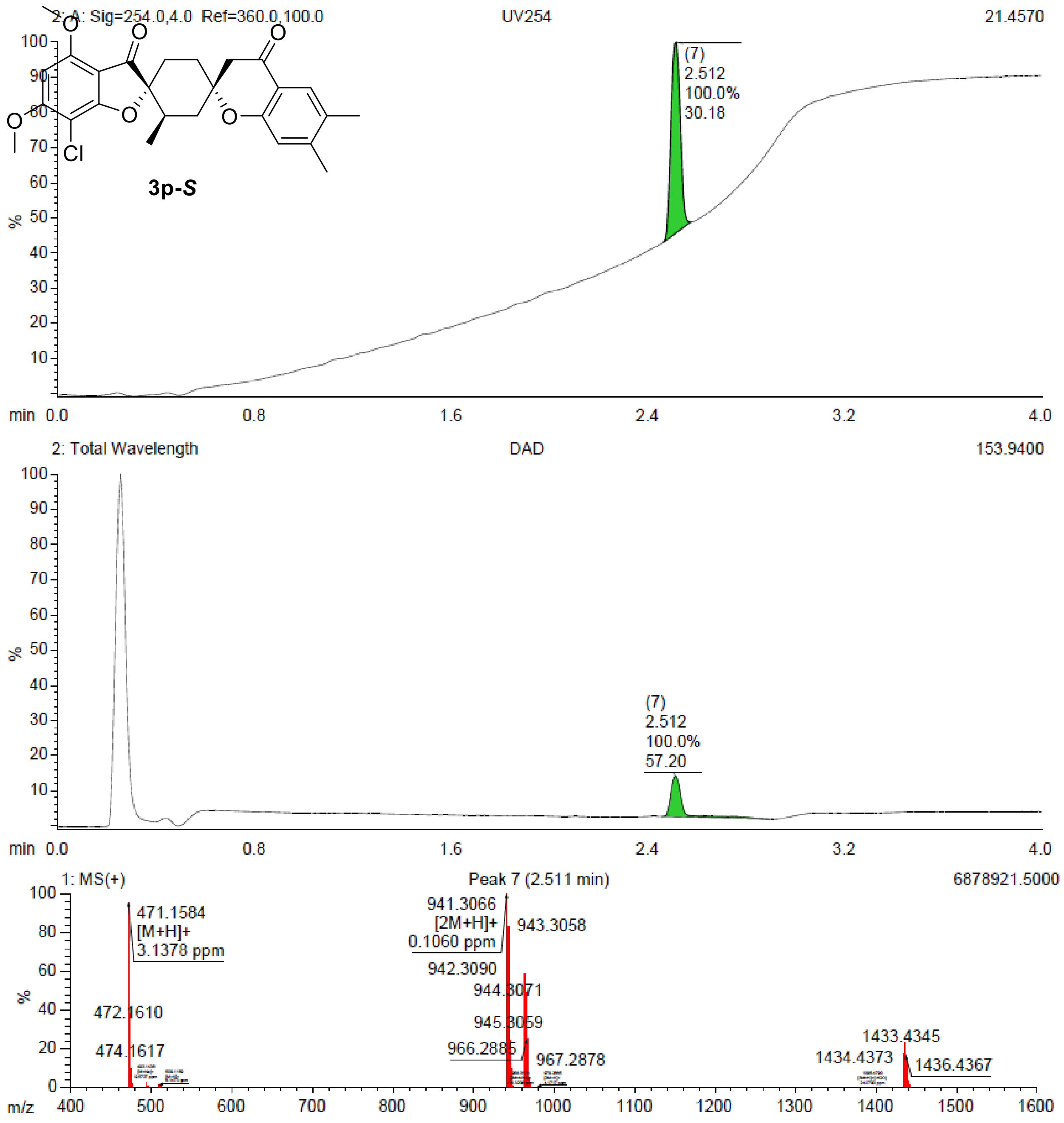

BPM	Error PPM	Error mDa	Target
941.3066	8.3641	12.1397	471.1…

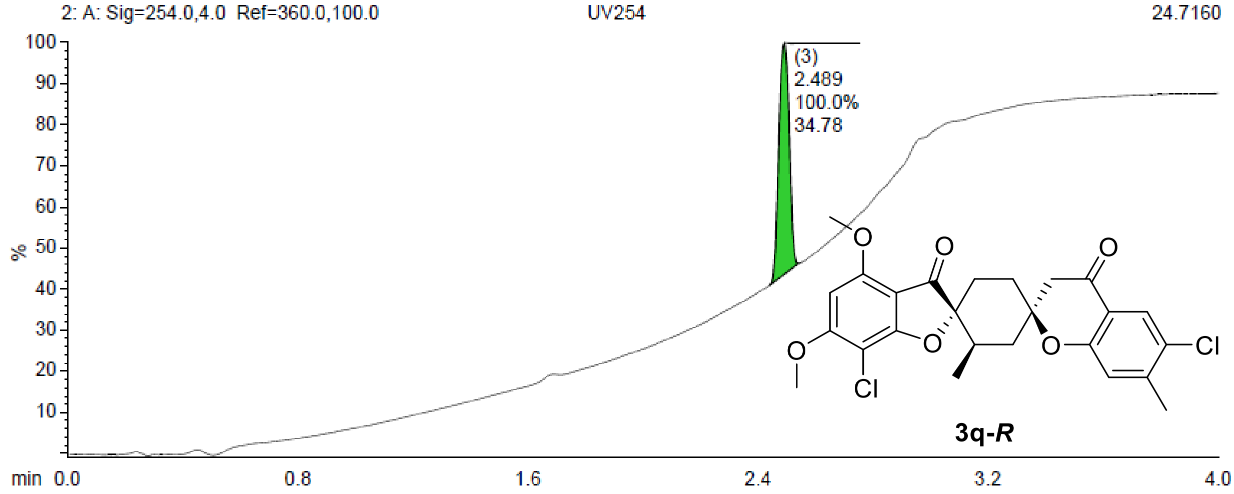

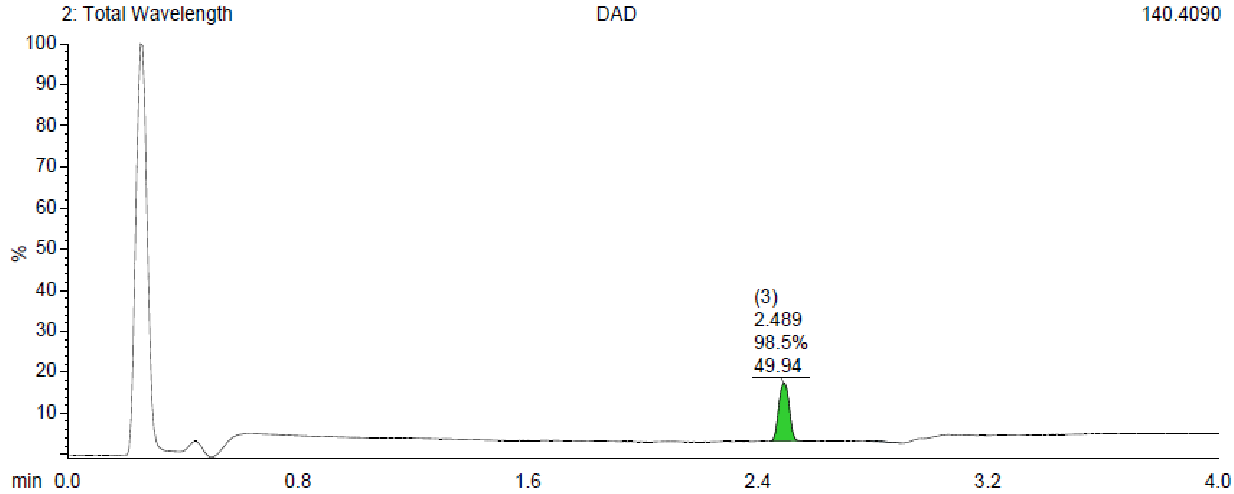

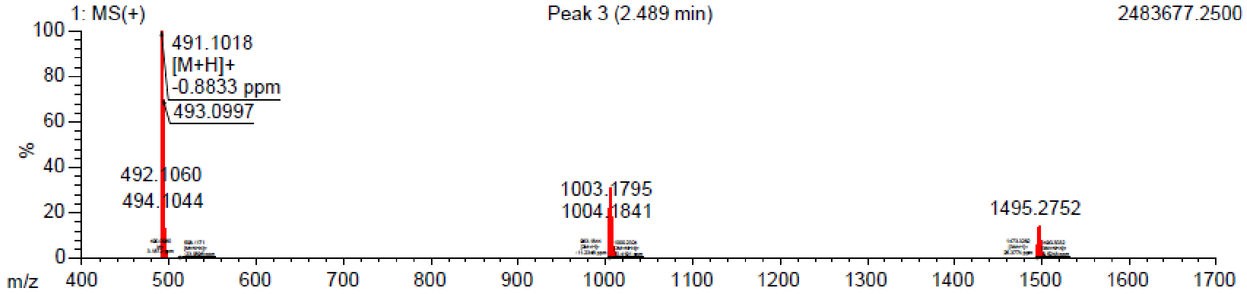

BPM	Error PPM	Error mDa	Target
491.1018	14.3024	21.6430	490.0...

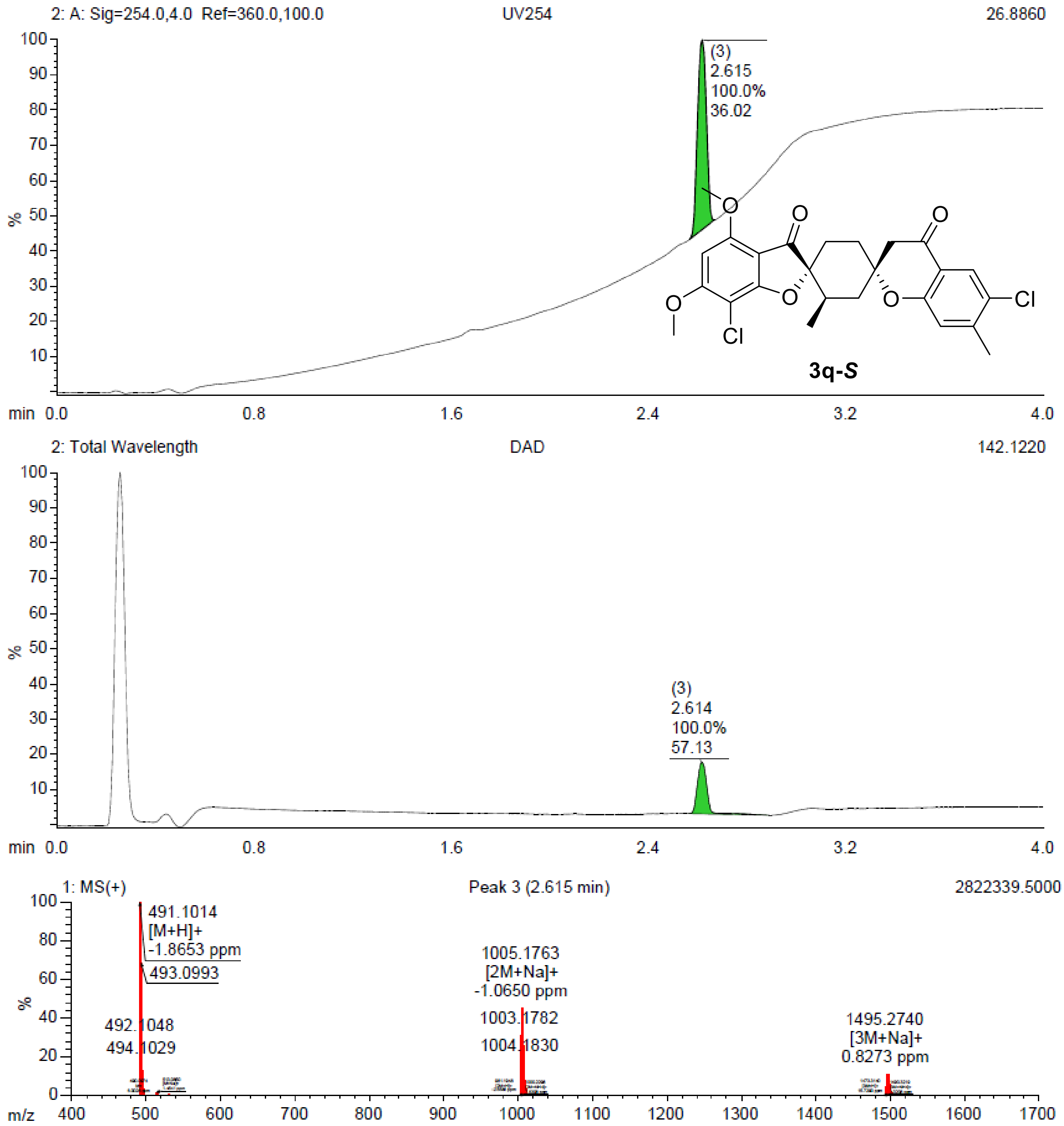

BPM	Error PPM	Error mDa	Target
491.1014	-10.5627	-15.9839	490.0…

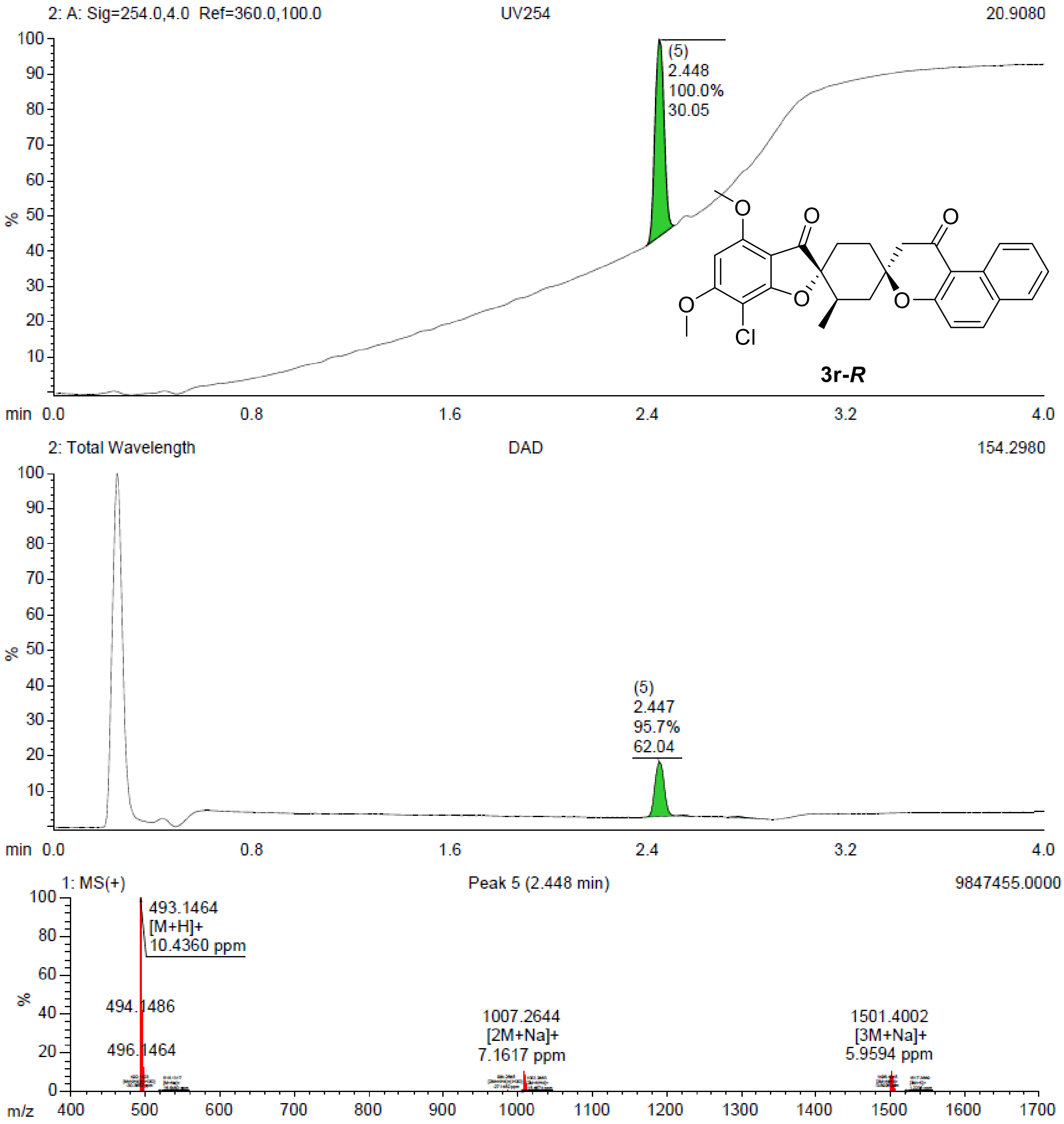

BPM	Error PPM	Error mDa	Target
493.1464	1.2238	1.8569	492.1…

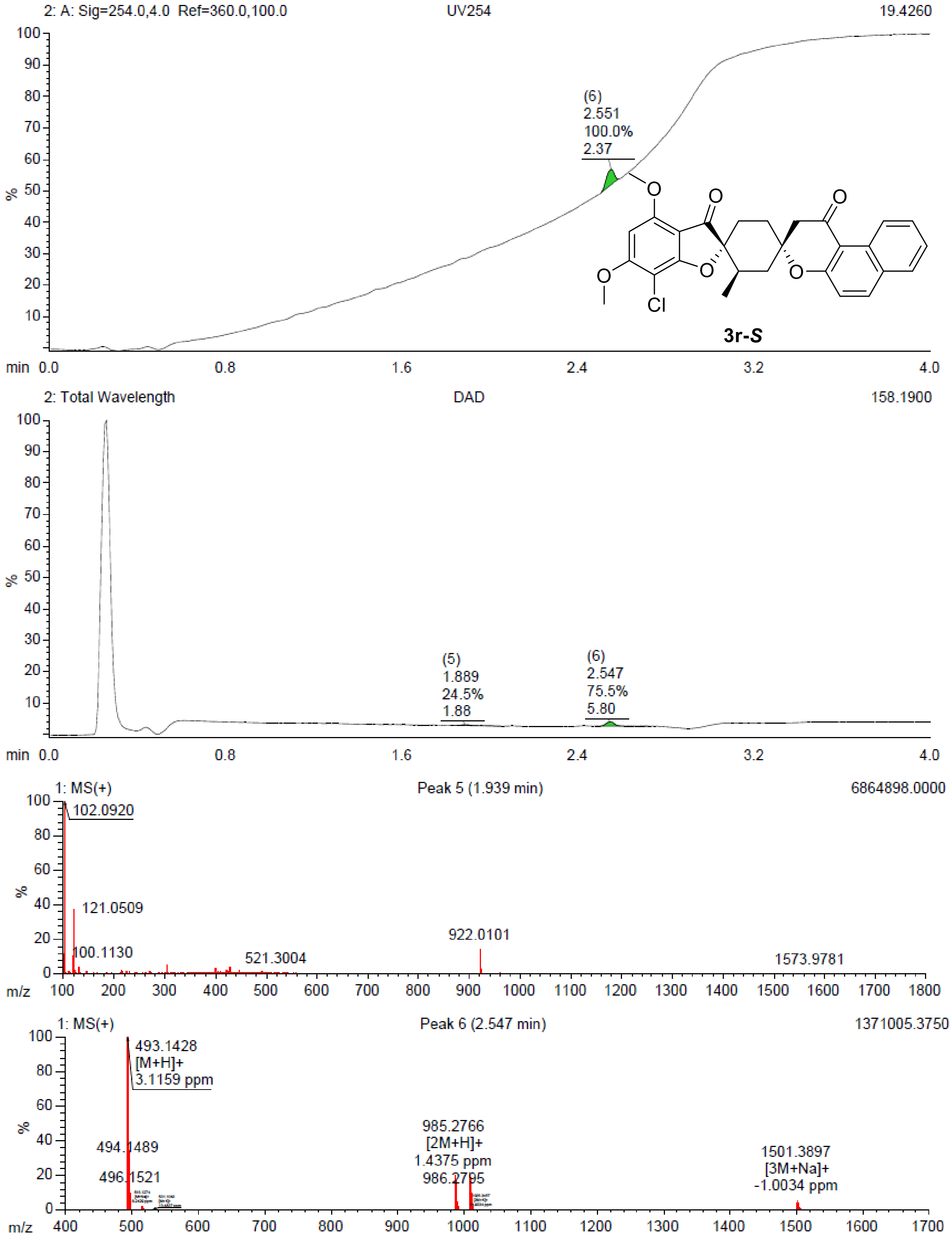

BPM	Error PPM	Error mDa	Target
493.1428	-1.0034	-1.5065	493.1…

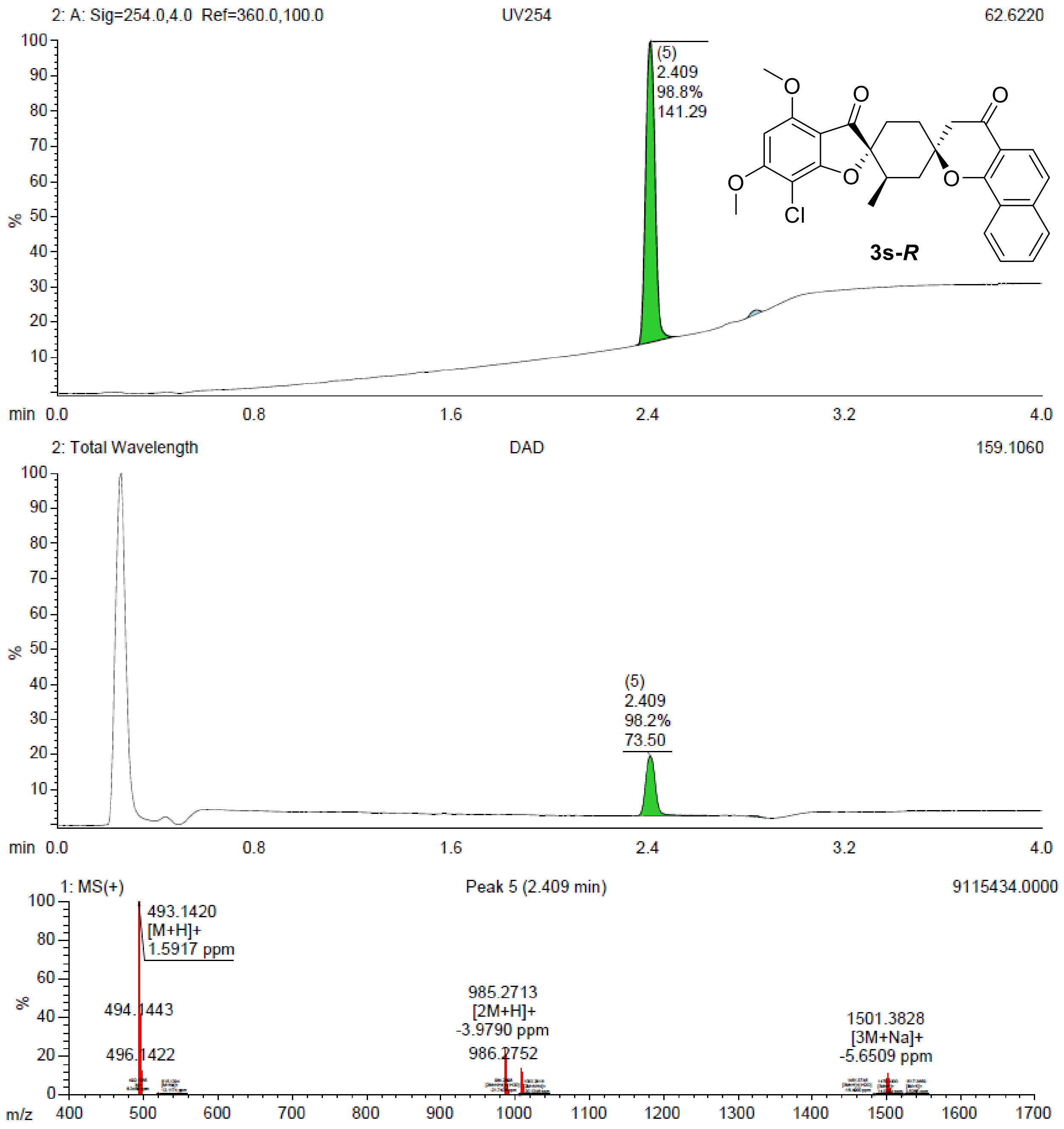

BPM	Error PPM	Error mDa	Target
493.1420	0.5265	0.7988	492.1…

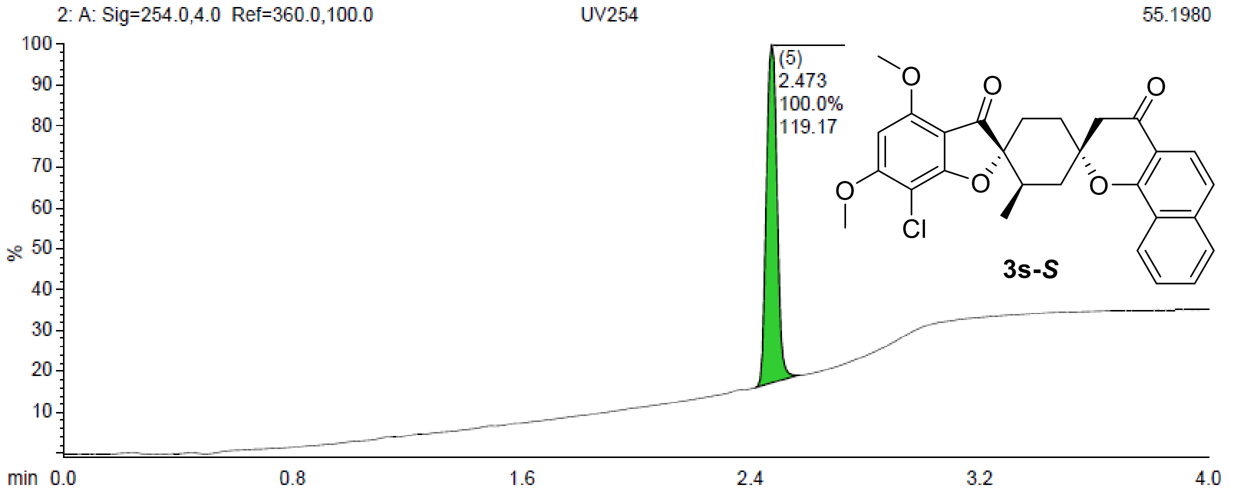

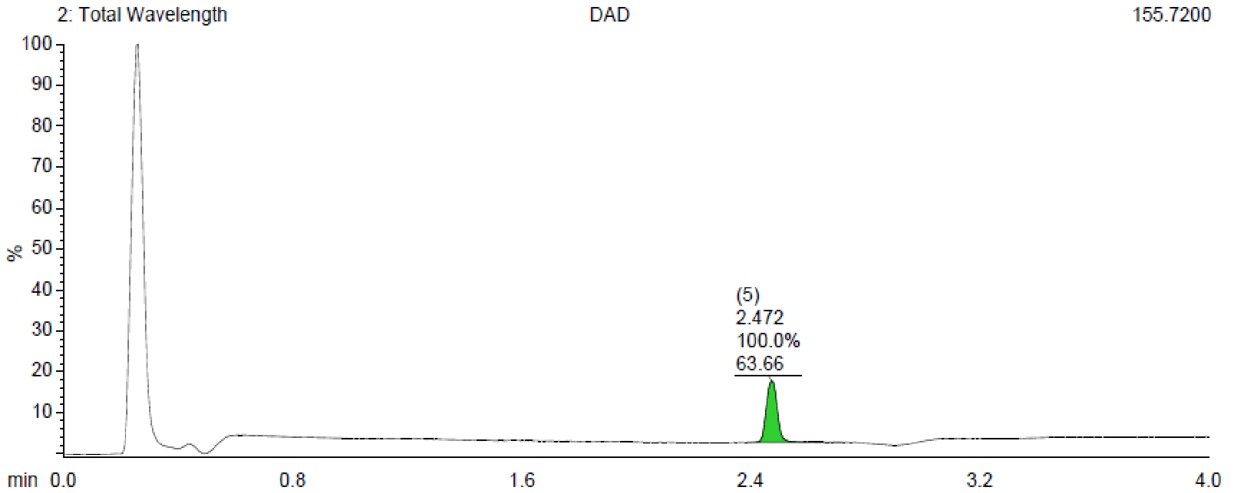

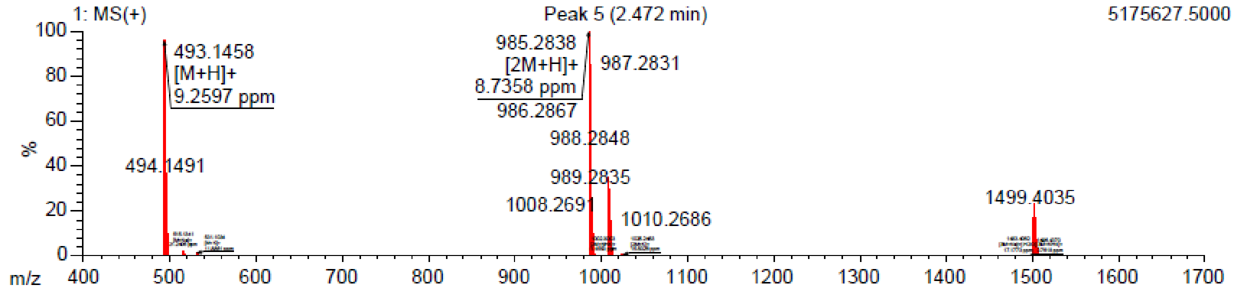

BPM	Error PPM	Error mDa	Target
985.2838	27.1604	41.2122	493.1…

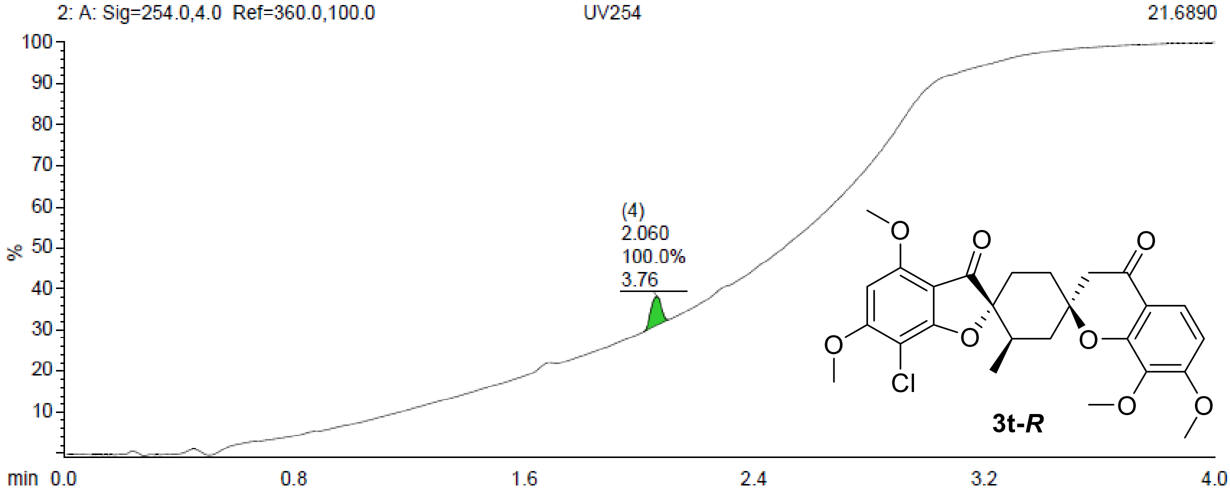

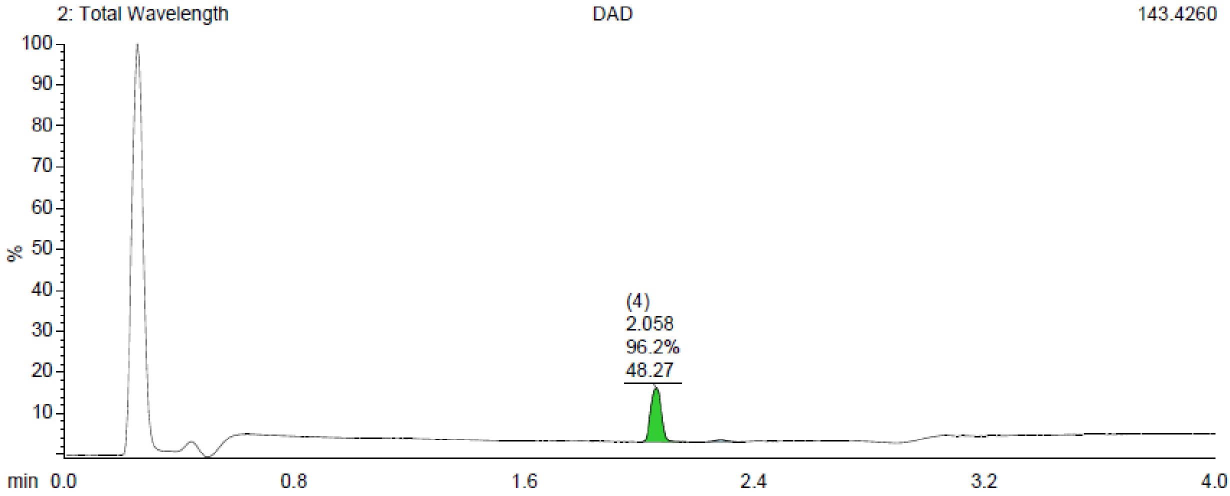

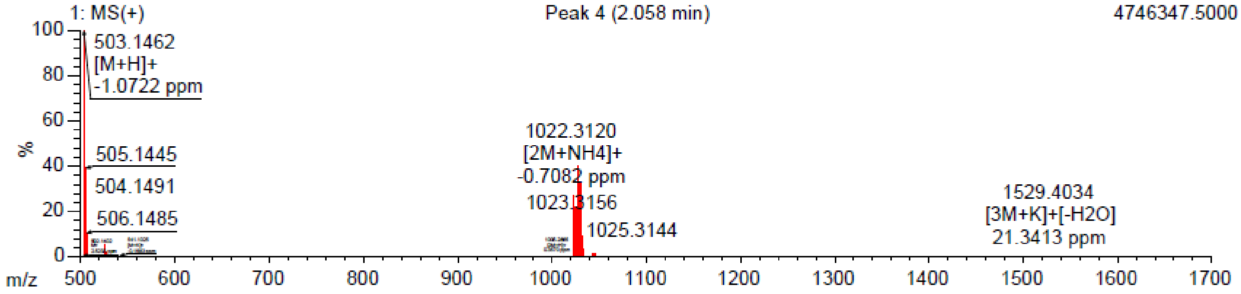

BPM	Error PPM	Error mDa	Target
503.1462	21.3413	32.6388	502.1…

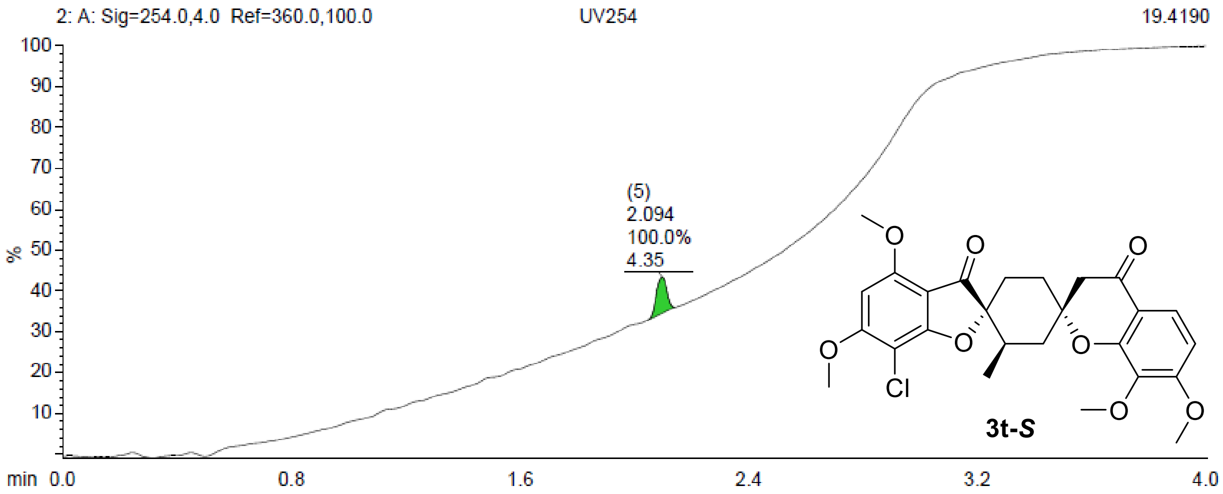

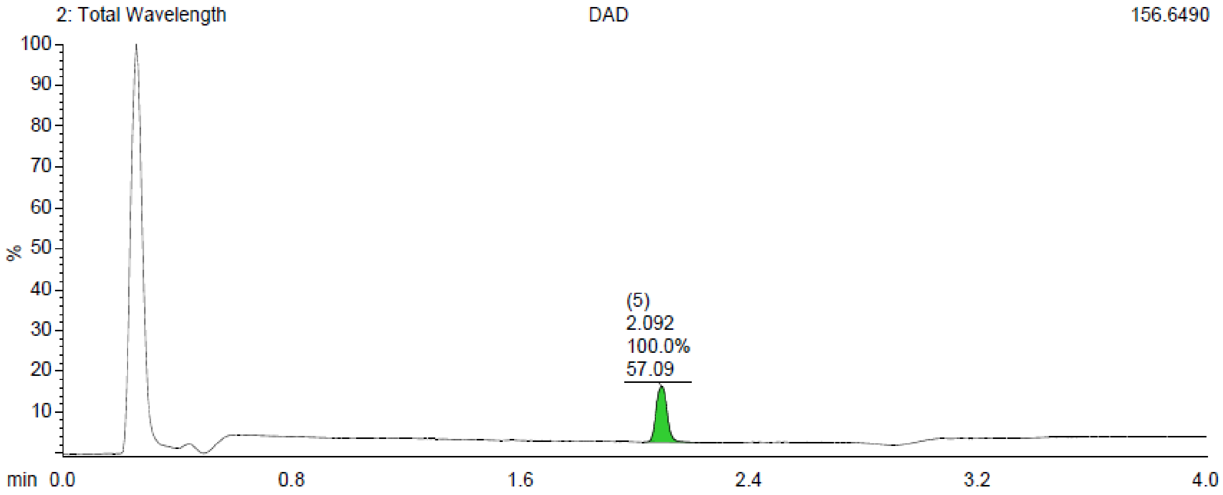

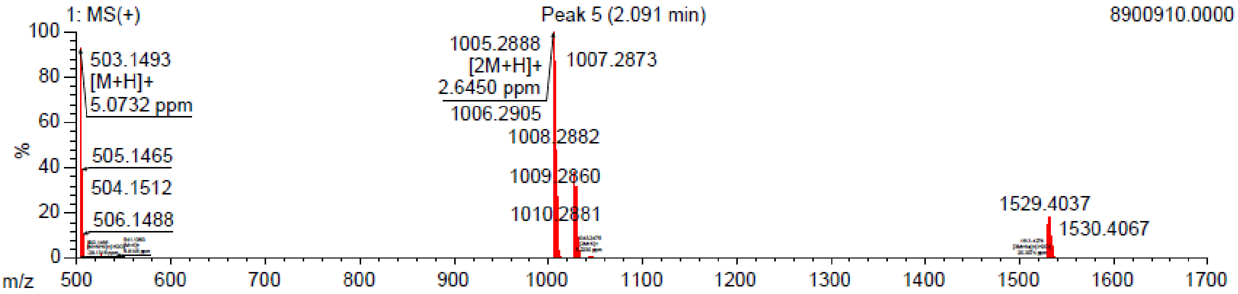

BPM	Error PPM	Error mDa	Target
1005.2888	-1.5293	-2.3420	502.1…
